# Global and regional spending on dementia care from 2000–2019 and expected future health spending scenarios from 2020–2050: An economic modelling exercise

**DOI:** 10.1016/j.eclinm.2022.101337

**Published:** 2022-03-13

**Authors:** Paola Pedroza Velandia, Molly K Miller-Petrie, Carina Chen, Suman Chakrabarti, Abigail Chapin, Simon Hay, Golsum Tsakalos, Anders Wimo, Joseph L Dieleman

**Affiliations:** a Institute for Health Metrics and Evaluation, University of Washington, 3980 15th Ave NE, Seattle, WA 98195, USA; b Center for Alzheimer Research, Karolinska Institutet, Stockholm 17177, Sweden

**Keywords:** Dementia, Alzheimer's disease, Health care spending

## Abstract

**Background:**

The global burden of dementia is increasing. As diagnosis and treatment rates increase and populations grow and age, additional diagnosed cases will present a challenge to healthcare systems globally. Even modelled estimates of the current and future healthcare spending attributable to dementia are valuable for decision makers and advocates to prepare for growing demand.

**Methods:**

We modelled healthcare spending attributable to dementia from 2000 to 2019 and expected estimated future spending from 2020 to 2050 under multiple scenarios. Data were from the Global Burden of Diseases 2019 study and from two systematic literature reviews. We used meta-regression to estimate the fraction of dementia spending that is attributable to dementia for those receiving nursing home-based care and for those receiving community-based care. We used spatiotemporal Gaussian process regression to account for data missingness and model diagnosis and treatment rates, nursing home-based care and community-based care rates, and unit costs for the many countries without their own underlying estimates. Projections of future spending estimate a baseline scenario from 2020 to 2050 based on ongoing growth. Alternative scenarios assessed faster growth rates for dementia diagnosis and treatment rates, nursing home-based care, and healthcare costs. All spending is reported in 2019 United States dollars or 2019 purchasing-power parity-adjusted dollars.

**Findings:**

Based on observed and modelled inputs, we estimated that global spending on dementia increased by 4.5% (95% uncertainty interval: 3.4–5.4%) annually from 2000 to 2019, reaching $263 billion (95% uncertainty interval [UI] $199– $333) attributable to dementia in 2019. We estimated total healthcare spending on patients with dementia was $594 billion (95% UI $457–$843). Under the baseline scenario, we estimated that attributable dementia spending will reach $1.6 trillion (95% UI $0.9–$2.6) by 2050. We project it will represent 11% (95% UI 6–18%) of all expected health spending, although it could be as high as 17% (95% UI 10–26%) under alternative scenarios.

**Interpretation:**

Health systems will experience increases in the burden of dementia in the near future. These modelled direct cost estimates, built from a relatively small set of data and linear time trends, highlight the magnitude of health system resources expected to be used to provide care and ensure sufficient and adequate resources for aging populations and their caretakers. More data are needed to corroborate these important trends.

**Research in context**

**Evidence before this study**

We searched PubMed with the terms dementia [MH], underdiagnosis, and dementia diagnosis [MH]; and with the terms Alzheimer Disease [MH] AND Dementia AND Costs and Cost Analysis [MH] OR Health Spending [MH] OR Cost of Illness [MH] AND 1990:2021 [DP] for data to populate our models to estimate diagnosis and treatment rates, nursing home-based care and community-based care rates, and unit of care costs. We extracted data from 295 publications in multiple languages in an effort to minimize bias and capture data from as many geographies as possible. Previous estimates capture the healthcare costs of caring for patients with dementia, but not the healthcare costs attributable to dementia. Our estimates report modelled healthcare costs attributable specifically to dementia.

**Added value of this study**

This study provides modelled estimates of healthcare spending attributed to dementia globally and in 195 countries and territories from 2000 to 2019 as well as modelled future health spending scenarios from 2020 to 2050. A key innovation of our study is the estimation of the fraction of spending that is attributable to dementia, rather than the estimation of healthcare spending on patients with dementia.

**Implications of all the available evidence**

Global spending on dementia care is substantial and will continue to grow in coming decades; the financial implications of this growth is expected to have the largest impacts in low- and middle-income countries, where improved diagnosis and treatment rates coupled with growing and aging populations will drive a great expansion in the number of cases requiring care. Planning for healthcare system design and services to treat these future cases and the policy choice of how to treat them will determine the total costs incurred in these countries.

Alt-text: Unlabelled box

## Introduction

More than 57 million people are currently estimated to be living with dementia, including people with Alzheimer's disease.^1^ By 2050, it has been predicted that dementia prevalence will increase by nearly 300%, most rapidly in low- and middle-income countries.^2^ There is no cure or alleviating treatment for dementia, and treatment largely consists of community or nursing home-based care for daily needs.^3^^,^^4^ As global populations age and methods to improve diagnosis for dementia are developed and scaled up, it is likely that rates of dementia, related treatment needs, and the burden on healthcare systems and community carers will continue to increase.

The provision of quality healthcare for dementia patients is generally quite expensive. In high-income countries, total societal spending was previously estimated to exceed US$660 billion in 2015.^2^^,^^5^ These costs are driven by healthcare provided to patients receiving nursing home-based care, as well as healthcare provided for those with dementia receiving community-based care. While this study only focuses on the direct costs associated with these types of care, it highlights the need to better understand the societal and economic costs of dementia, which are immense. Understanding the economic burden of dementia to patients, caregivers, the broader economy, and healthcare systems is critical for policymakers to effectively budget, prioritise, and plan for future healthcare needs.

Projected increases in the healthcare cost for rising rates of dementia are of critical concern not just in high income countries – where costs associated with care are currently greatest^2^^,^^5^ – but also in low- and middle-income countries, where data on costs of dementia care are much sparser. Efforts to prepare for this future burden are hampered by the lack of comparable spending estimates across locations and years. Further, to our knowledge, current estimates of the future costs of dementia care have only been produced at the global level or for specific high and upper middle-income countries,^6^, ^7^, ^8^, ^9^, ^10^ despite the growing burden in low- and middle-income countries.

Prior estimates of the cost of dementia have primarily been produced in the World Alzheimer's Report by Prince and Wimo through 2015,^11^, ^12^, ^13^, ^14^ but are not available at the national-level for all countries since 2006. Further, these and other existing estimates of the costs of dementia care have not distinguished between the spending attributable specifically to dementia and the other costs of healthcare incurred by individuals with dementia (i.e. spending associated with health conditions other than dementia). Given the growing size of the elderly population in many countries, growing rates of chronic disease prevalence in this population, and the associated costs of providing them with appropriate care, it is increasingly important to distinguish between the additional burden that dementia places on healthcare systems, patients, and caregivers, and to differentiate this burden from other chronic diseases.

This study aims to collect all available information about the direct costs of healthcare for dementia, model the many gaps, and make estimates of global dementia spending for 195 countries and territories from 2000 to 2019, as well as potential future dementia spending from 2020 to 2050 under four different scenarios. While underlying data is sparse, especially in low-income countries, these build from what is available and the modelled estimates incorporate changing country-specific age-specific populations, dementia prevalence, diagnosis and treatment rates, as well as community-based care and nursing home-based care rates, and direct cost of care attributable to dementia. These estimates do not include a quantification of the cost of care provided to dementia patients outside of the formal health sector, nor does it include lost economic productivity because of dementia. This manuscript was produced in accordance with GBD Protocols.

## Methods

### Overview

We extracted data from the Global Burden of Disease (GBD) 2019 study and completed literature reviews to identify additional data, employed spatiotemporal Gaussian process regression modelling (ST-GPR), and adjusted estimates using a mixed-effects meta-regression to produce estimates of total annual healthcare spending attributable to dementia globally and for 195 countries and territories from 2000 to 2019, and estimated multiple future health spending scenarios for dementia from 2020 to 2050. When sample size was available we incorporated it into our ST-GPR models to contribute to the uncertainty in our estimates.^15^ We propagated uncertainty throughout the modelling process by using 1000 draws from the distribution of each modelled element (e.g., dementia prevalence, diagnosis and treatment rates, nursing home-based care rates among patients diagnosed and undiagnosed with dementia, and unit costs), and by performing all calculations at the draw level. We estimated 95% uncertainty intervals using the 2.5 and 97.5 percentile of the sample distribution. Modelled inputs used to make these estimates included: (1) the age-sex-specific population and dementia prevalence rates for each country and year, (2) diagnosis and treatment rates among prevalent cases, (3) the rate of nursing home-based care versus community-based care among diagnosed and treated cases, (4) the rate of nursing home-based care among undiagnosed cases, and (5) the cost per patient of dementia care for patients receiving nursing home-based care and patients receiving community-based care. A flow diagram of this process is presented in the appendix (Appendix Fig. S1) and described in detail below.

### Data

*Disease prevalence:* We extracted dementia prevalence counts and rates from the GBD 2019 study which provides estimates at the national level by year.^1^ Although, GBD 2019 quantifies the proportion of dementia that is due to stroke, Down syndrome, Parkinson's disease, and traumatic brain injury^16^ we utilize all dementia prevalent cases irrespective of cause as costs attributable to dementia and do not vary by dementia cause. While dementia prevalence rates are expected to increase as life expectancy increases around the world, there is evidence that modifiable risk factors might contribute to lowering dementia prevalence rates^17^ or at least delaying its onset. Delaying the onset of dementia will greatly benefit older adults.^18^

*Diagnosis and treatment rates:* Data reporting diagnosis and treatment rates (per prevalent case) came from a systematic literature review conducted on May 25th, 2021 on PubMed with the terms dementia[MH], underdiagnosis, and a dementia diagnosis[MH]. We identified a total of 726 results for title and abstract screening, of which 41 proceeded to full article review and extraction and standardization. Further detail can be found in the appendix. Due to the limited data on diagnosis and treatment rates available in much of the world, particularly in low- and middle-income countries, we modelled missingness data using ST-GPR modelling. In the presence of data ST-GPR models give significant weight to observed data. On the other hand, in the absence of data ST-GPR borrows strength across time and geographies to generate a complete set of estimates, and the Gaussian process generates uncertainty intervals for the estimates. This three-step Bayesian modelling process has been described in detail previously.^19^ Due to the lack of data on dementia treatment among undiagnosed patients, we assumed that those not diagnosed only have healthcare spending attributable to dementia if they receive nursing home-based care.

*Nursing home-based care rates among patients diagnosed with dementia:* Data on the fraction of diagnosed and treated dementia cases receiving nursing home-based care were extracted from a second literature review, and then a complete set of estimates were modelled using ST-GPR. The second literature review used the terms Alzheimer Disease[MH] AND Dementia AND Costs and Cost Analysis[MH] OR Health Spending[MH] OR Cost of Illness[MH] AND 1990:2021[DP]. It was conducted on May 25th, 2021 on PubMed. We identified a total of 1362 results for title and abstract screening, of which 254 proceeded to full article review and extraction (see appendix for details). Those not receiving nursing home-based care were considered to be receiving community-based care.

*Nursing home-based care rates among undiagnosed dementia patients:* Due to the high rates of dementia under-diagnosis around the world, we expect that a fraction of undiagnosed patients living in nursing homes have dementia, and some healthcare spending for their care should be attributed to dementia. As part of our first literature review we extracted diagnosis and treatment rates for patients with dementia in community, nursing home, and not reported care settings. Using a fixed-effects model we estimated dementia diagnosis and treatment rates by care setting. We incorporated uncertainty into our estimation process by capturing sample size of our inputs and including it into our ST-GPR models. These estimates in addition to nursing home-based care rates were used to estimate the fraction of undiagnosed dementia patients who are cared for in nursing home settings. More details can be found in the appendix.

*Unit costs:* Data on annual healthcare costs for a person living with dementia were extracted from the second systematic literature review, described above. For this process, the unit cost of dementia care was calculated as the average annual cost for treating a dementia patient, stratified by patients receiving nursing home-based care vs community-based care. Outliers were identified by performing Cook's distance analysis.^20^ A list of the outliers excluded from the analysis can be found in the appendix. Again, due to limited data, we used ST-GPR to model dementia unit costs across all countries and years, and to generate uncertainty. All unit costs were converted to 2019 USD, with purchasing-power parity-adjusted estimates included in the appendix.

Each of the ST-GPR models included total health spending per person (THE) and the Socio-demographic Index (SDI) as covariates. The SDI is a proxy for development that extends beyond just economic indicators, and has been shown to be well correlated with health outcomes.^21^^,^^22^ It is calculated as a composite of total fertility in women under 25, income, and education. While specific data on dementia spending was sparse, we had a full set of inputs for SDI and THE helping our models accurately fill in the blanks in the absence of observed dementia spending data.

### Attributable costs

We used a mixed-effects meta-regression^23^ conducted on a subset of the unit cost data that contained information on attribution to estimate the amount of spending that is directly attributable to care for dementia, rather than to care for other conditions, stratified by care setting. Our analysis included four care settings: nursing home, community, mixed, or not reported. To estimate uncertainty around our estimates we ran a simulation based on the results of our meta-analysis. A more detailed explanation can be found in the appendix. Due to the lack of data on attributable costs of dementia on undiagnosed patients, we assumed that healthcare spending attributable to undiagnosed persons with dementia was half the estimated attribution rate for persons diagnosed with dementia. To test this assumption we performed a sensitivity analysis where we assumed attributable rates were identical for diagnosed and undiagnosed dementia patients. A more detailed account of the methods and a plot comparing retrospective to sensitivity analysis estimates can be found in the appendix (Fig. S13).The final unit cost attributable to dementia was calculated by multiplying the estimated unit cost in each care setting by the attributable spending rate. Unless otherwise specified, all estimates presented in this paper reflect healthcare spending attributable to dementia, rather than healthcare spending on patients with dementia. The attributable rates applied were not country- or year-specific because of insufficient data to capture heterogeneity across time or country.

### Modelling retrospective dementia spending (2000–2019)

Attributable healthcare spending on dementia patients receiving nursing home-based care was estimated for each location and year as the total population multiplied by the prevalence, the diagnosis and treatment rate, the nursing home-based care rate, and the unit cost attributed to dementia for patients cared for in nursing homes. Similarly, attributable spending on undiagnosed dementia patients receiving nursing home-based care was estimated as the total population multiplied by the prevalence, one minus the diagnosis and treatment rate, inpatient treatment rate for undiagnosed patients, and half the unit cost attributed to dementia for nursing home residents. Lastly, attributable spending on dementia patients receiving community-based care was estimated as the total population multiplied by the prevalence, the diagnosis and treatment rate, one minus nursing home-based care rate, and the unit cost attributed to dementia for patients cared for in the community. Total annual spending attributable to dementia was estimated as the summed totals of nursing home-based care and community-based care costs attributable to dementia in each country aggregated to the global level for each year. Because of the relative magnitude of The United States health spending, around 30% of total health spending in the world, we extracted dementia US spending estimates from another study focused on measuring healthcare spending by disease in the US.^24^ Their estimates are informed by a robust set of microdata using a consistent definition with the one used in this study.

### Projecting future dementia spending (2020–2050)

#### Future healthcare spending scenarios: baseline scenario

We modelled global- and country-specific dementia costs for 2020–2050 under a baseline scenario that assumed the country-specific annualized rates of change (AROC) for diagnosis and treatment rates, nursing home-based care rates, and nursing home-based care and community-based care unit costs for 2000–2019 hold for 2020–2050. To estimate future dementia prevalence, we used age-sex-specific projections for populations by country from GBD 2019^17^ and multiplied these by age-sex-specific dementia prevalence rate projections from GBD 2019.^25^ All future health spending estimates were adjusted to represent only costs attributable to dementia, as with the retrospective estimates.

#### Future health spending scenarios: alternative scenarios

We modelled attributable dementia healthcare spending for the period 2020–2050 for four alternative scenarios. For these alternative scenarios, we assumed that dementia prevalence was unchanged from our baseline scenario. For the first three alternative scenarios we assumed that one of the key drivers – diagnosis and treatment rates, nursing home-based care rates, or unit costs – increased at an accelerated pace. To estimate the accelerated pace, we selected the 85th percentile of the AROCs observed across countries for 2000 to 2019. If a country-specific AROC was lower than the 85th percentile across all locations in the baseline scenario, we used the 85th percentile AROC to approximate a realistic but accelerated rate of increase. If the country-specific AROC was already at or above the 85th percentile, it was unchanged. (Use of the 85th percentile to highlight a more extreme scenario has been a convention elsewhere.)^26^

#### Sensitivity analyses

We assessed the sensitivity of our results to inputs disaggregated by severity. We used identical methods as in the main analysis, but stratified all estimated quantities, including costs, diagnosis and treatment rates, and nursing home-based care rates, by severity – mild, moderate, or severe. To account for substantial data missingness, we used ST-GPR to model each of the inputs. A more detailed account of the methods and a plot comparing retrospective to sensitivity analysis estimates can be found in the appendix (Fig. S12). We conducted an additional sensitivity analysis where we assessed if the difference between our estimates with and without country specific input data was statistically significant. (See appendix Table S13.)

### Role of the funding source

This research was supported by Gates Ventures. The sponsor of the study had no role in the study design, data collection, data analysis, data interpretation, or writing of the report. The first and corresponding authors, Paola Pedroza Velandia and Joseph Dieleman, had full access to all the data in the study and had final responsibility for the decision to submit for publication.

## Results

**Figure 1:**
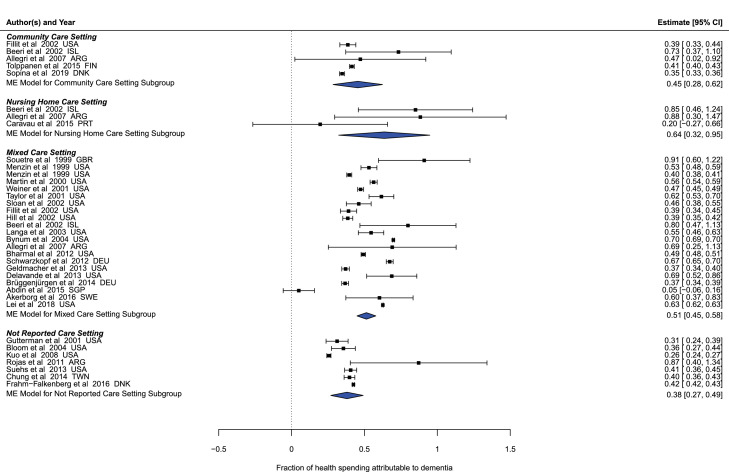
Fraction of total health spending on dementia patients attributable to dementia care, by care setting.

Our meta-regression estimated that 45% (95% uncertainty interval [UI] 29–62%) of the healthcare spending on patients receiving community-based care who are diagnosed with dementia can be attributed to dementia care, with 55% (95% UI 38–72%) attributable to other health conditions (Figure 1). For patients receiving nursing home-based care who are diagnosed with dementia, we estimated that 64% (95% UI 32–95%) of total spending was attributable to dementia, and 36% (95% UI 5–68%) of spending was attributable to other health conditions. For patients with dementia who receive nursing home-based care, but who lack a dementia diagnosis (but who have dementia) we estimated that 32% (95% UI 16–48%) of spending was attributable to dementia while the remainder 68% (95% UI 53–84%) was attributable to other health conditions. Unless otherwise noted, all estimates herein have been adjusted to reflect these attribution rates.

Black squares highlight extracted estimates from previous research, while the blue diamonds highlight our meta-regression estimates. Whiskers show 95% confidence interval.

We estimated that in 2019, healthcare spending on people with dementia was $594 billion (95% UI $457–$843) (not shown). After removing the spending estimated to be on health conditions other than dementia, modelled total global spending attributable to dementia was $263 billion (95% UI $199–$333) in 2019. Our models estimated an annual increase of 4.5% (95% UI 3.4–5.4%) from 2000, when it was $114 billion (95% UI $94.2–$134) (not shown). Per person, we estimated global spending attributable to dementia to have increased from $19 (95% UI $15–$22) to $34 (95% UI $26–$43) over this period.

Geographically, estimated attributable dementia spending per person was highest in the GBD High-income region $206 [95% UI 157–260]), followed by Central Europe, Eastern Europe, and Central Asia ($23 [95% UI 12–36]), and was lowest in Sub-Saharan Africa ($0.5 [95% UI 0.3–0.8]), followed by South Asia ($0.6 [95% UI 0.3–1.0]) (Table 1). A complete set of modelled spending estimates reported at the country level are included in the appendix (Appendix Fig. S5*,* Table S8).

**Table 1:** Estimated attributable dementia spending by World Bank income groups and Global Burden of Disease super regions, 2019.

	Spending attributable to dementia in 2019 (In millions of USD)	Spending per person (USD)	Spending per person (PPP)	Dementia spending as a fraction of the total health spending (%)	Annualized rate of change 2000 to 2019
Global	$262 788 ($198 589–$332 873)	$33.98 ($25.68–$43.05)	$41.32 ($30.12–$53.16)	3.0% (2.3–3.9%)	4.5% (3.4–5.4%)
**World Bank Income Group**					
High income	$228 418 ($173 134–$287 719)	$189.22 ($143.42–$238.34)	$207.29 ($155.07–$262.38)	3.3% (2.5–4.2%)	4.1% (3.0–5.1%)
Upper middle income	$30 809 ($17 669–$47 567)	$11.43 ($6.55–$17.64)	$21.41 ($12.37–$32.25)	2.1% (1.2–3.2%)	9.1% (7.1–11.1%)
Lower middle income	$3349 ($1958–$5046)	$1.08 ($0.63–$1.63)	$3.50 ($2.02–$5.29)	1.2% (0.7–1.8%)	7.2% (5.7–8.8%)
Low income	$211 ($133–$300)	$0.29 ($0.18–$0.41)	$1.03 ($0.65–$1.53)	0.8% (0.5–1.2%)	5.5% (4.2–6.7%)
**Global Burden of Disease Super Regions**					
Central Europe, Eastern Europe, and Central Asia	$9770 ($5149–$15 082)	$23.39 ($12.33–$36.11)	$55.96 ($29.26–$86.65)	4.0% (2.1–6.1%)	5.7% (3.9–7.5%)
High-income	$223 515 ($170 595–$281 456)	$206.29 ($157.45–$259.76)	$222.10 ($167.40–$282.95)	3.3% (2.6–4.2%)	4.0% (2.9–5.0%)
Latin America and Caribbean	$3740 ($2138–$5725)	$6.42 ($3.67–$9.82)	$12.25 ($7.04–$18.59)	1.1% (0.6–1.7%)	6.7% (4.6–8.7%)
North Africa and Middle East	$2101 ($1303–$3066)	$3.45 ($2.14–$5.04)	$9.52 ($5.75–$13.98)	0.9% (0.6–1.3%)	8.2% (6.5–9.9%)
South Asia	$1014 ($543–$1735)	$0.56 ($0.30–$0.96)	$1.85 ($0.98–$3.16)	0.8% (0.4–1.4%)	9.5% (6.1–12.5%)
Southeast Asia, East Asia, and Oceania	$22 097 ($11 512–$36 651)	$10.24 ($5.34–$16.99)	$17.60 ($9.39–$28.44)	2.4% (1.2–3.9%)	11.3% (8.4–14.2%)
Sub-Saharan Africa	$552 ($325–$816)	$0.51 ($0.30–$0.76)	$1.20 ($0.72–$1.75)	0.7% (0.4–1.0%)	5.7% (3.9–7.6%)

Table one reports attributable dementia spending by World Bank income groups and GBD super regions in millions and per person in 2019 USD. It also reports the fraction of dementia spending over total health spending in 2019 and the annualized rate of change from 2000 to 2019. The values in parenthesis represent the 95% uncertainty interval. Country specific estimates reported in Table S8 of the appendix.

Modelled spending increased the fastest in areas that did not have the most global spending in 2019. Southeast Asia, East Asia, and Oceania and South Asia were the GBD regions with the greatest annualized percent change in total spending, at 11.3% (95% UI 8.4–14.2%) and 9.6% (95% UI 6.1–12.5%), respectively (Table 1).

When considering total dementia spending as a ratio of total healthcare spending, ratios followed a similar geographic pattern as per person spending, with higher health spending ratios in the Central Europe, Eastern Europe, and Central Asia and GBD High-income regions and the lowest ratios in Sub-Saharan Africa followed by South Asia (Table 1).

**Figure 2:**
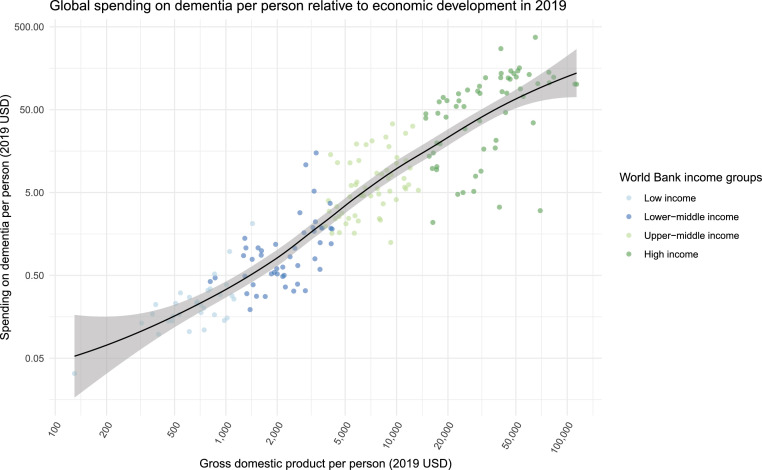
Estimated global dementia spending per person relative to economic development, 2019.

**Figure 3:**
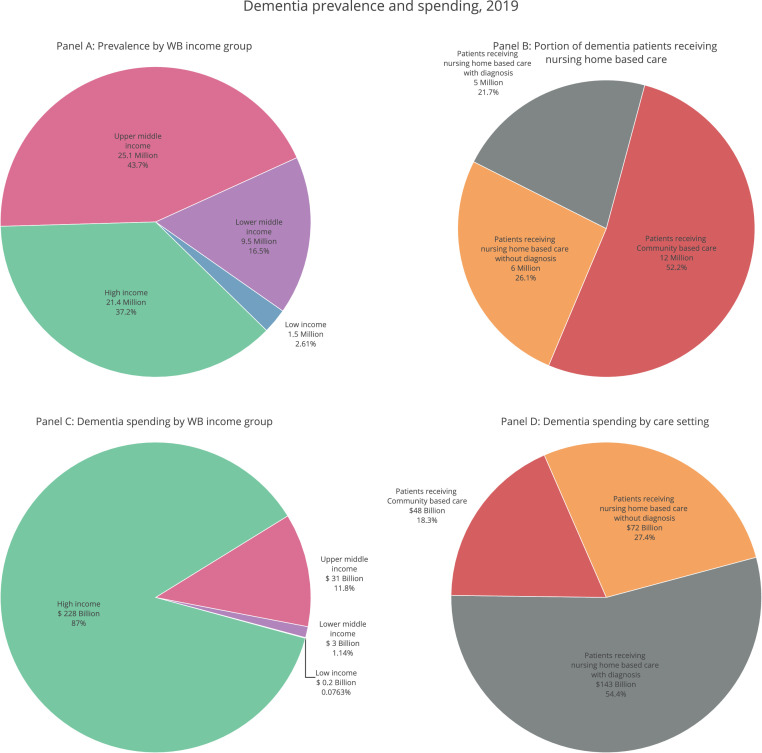
Dementia prevalence and estimated spending by World Bank income group, 2019 Panel A illustrates the distribution of dementia prevalent cases by 2019 World Bank income groups. Panel C illustrates dementia spending by 2019 World Bank income groups. (2019 USD) Panel B Illustrates the location where people with dementia are cared for. Panel D Illustrates dementia spending by care setting (2019 USD).

Estimated spending on dementia per person (USD 2019) was broadly associated with gross domestic product per person in 2019 (USD 2019), with World Bank defined high-income countries spending the most on dementia per person ($189 [95%UI 143–238]) and low-income countries spending the least per person ($0.3 [95%UI 0.2–0.4]) (Figure 2*,* Table 1). A similar relationship held for dementia spending per person and total healthcare spending (health spending) per person (2019 USD) (Appendix Fig. S9), with higher healthcare spending overall correlated with higher spending on dementia care. While high-income countries accounted for 87% of total spending (95%UI 82–91%), they accounted for just 37% of total prevalence (95%UI 36–39%) in 2019 (Figure 3).

Gross domestic product per person is plotted in the *x*-axis while spending on dementia per person is plotted in the *y*-axis. The points are colored according to the income group classification by the World Bank in 2019. Both variables are presented in 2019 USD.

Although more than half of dementia care worldwide is community-based care (Figure 3), nursing home-based care contributes to over 80% of global dementia spending. Nursing home-based care for those with a diagnosis contributed to 54% (95% UI 49–59%) of global spending on dementia while nursing home-based care for those without a diagnosis contributed to 27% (95% UI 22–33%) of global spending on dementia care (Figure 3), as the unit cost of nursing home-based care was higher than community-based care in all locations (Table s6).

Modelled estimates showed that global per person spending on dementia will increase annually by 5.1% (95% UI 3.7–6.6%) (not shown) from 2019 to 2050 under our baseline future health spending scenario, from $34 (95% UI $26–$43) per person to $163 (95% UI $89–$272) per person (Tables 1*,* 2, Fig. s5). Under this scenario, by 2050, we estimated that 57% (95% UI 37–78%) of total global spending will occur in the WB high-income group, where only 26% (95% UI 24–28%) of global prevalence will be concentrated (Appendix Fig. S11). While dementia spending is expected to increase in every region, in relative terms it is estimated it will increase by the largest annual percentage in North Africa and Middle East, with a 11.2% increase (95% UI 9.5–12.9%) (not shown), from $2 billion (95% UI $1–$3) to $58 billion (95% UI $27–$104) (Appendix Table S9).

**Table 2:** Estimated attributable dementia spending, by World Bank income groups and GBD super regions, 2050 - baseline and alternative scenarios.

	Spending attributable to dementia 2050 (in millions)	Spending per person attributable to dementia 2050 – *baseline*	Spending per person attributable to dementia 2050 – *accelerated diagnosis and treatment rates*	Spending per person attributable to dementia 2050 – *accelerated nursing homed based care rates*	Spending per person attributable to dementia 2050 – *accelerated unit costs*
Global	$1 557 735($851 288–$2 596 169)	$163.64($89.34–$272.12)	$208.90($117.66–$334.26)	$207.35($117.66–$331.48)	$254.82($153.61–$382.05)
**World Bank Income Group**					
High income	$864 303($495 089–$1 358 980)	$696.52($396.86–$1090.64)	$951.17($527.09–$1570.79)	$1009.67($559.06–$1597.29)	$1335.42($794.94–$1901.76)
Upper middle income	$641 321($194 791–$1 493 211)	$229.73($69.09–$529.33)	$266.29($111.26–$553.75)	$238.02($74.87–$542.85)	$252.13($85.27–$550.89)
Lower middle income	$49 339($22 280-$89 326)	$12.14($5.63–$22.02)	$15.13($7.78–$25.70)	$13.15($6.16–$23.62)	$15.01($7.21–$25.96)
Low income	$2771($1414–$4545)	$1.95($0.99–$3.22)	$2.51($1.37–$3.89)	$2.14($1.06–$3.48)	$2.78($1.50–$4.41)
**Global Burden of Disease Super Regions**					
Central Europe, Eastern Europe, and Central Asia	$62 432($27 716–$113 638)	$156.46($68.88–$286.59)	$199.45($92.91–$353.69)	$181.30($79.03–$321.27)	$233.37($105.52–$396.30)
High-income	$817 849($459 513–$1 301 505)	$726.71($407.62–$1149.76)	$994.24($534.68–$1668.68)	$1067.11($590.64–$1712.25)	$1410.58($836.67–$2028.98)
Latin America and Caribbean	$38 345($16 183–$72 212)	$53.94($22.82–$100.50)	$74.43($36.04–$129.22)	$64.15($26.66–$119.56)	$77.77($34.11–$138.45)
North Africa and Middle East	$57 887($27 347–$103 574)	$64.48($30.37–$116.00)	$85.14($42.39–$143.70)	$65.35($30.51–$117.43)	$83.40($40.27–$142.75)
South Asia	$18 730($4185–$48 69)	$8.85($2.00–$23.25)	$11.00($3.96–$25.70)	$9.10($2.08–$23.34)	$10.15($2.65–$24.46)
Southeast Asia, East Asia, and Oceania	$555 535($131 335–$1 402 283)	$261.65($61.35–$647.54)	$296.00($106.24–$662.29)	$267.92($63.68–$659.68)	$274.97($70.84–$668.37)
Sub-Saharan Africa	$6955($3401–$12 001)	$3.24($1.61–$5.61)	$4.28($2.26–$7.05)	$3.83($1.84–$6.71)	$4.61($2.43–$7.52)

Table two reports spending attributable to dementia in 2050 in billions and per person for the reference scenario as well as dementia spending per person for each of the forecasted scenarios for World Bank income groups and GBD super regions. All spending is measured in 2019 US dollars. The values in parenthesis represent the 95% uncertainty interval. Country specific estimates reported in Table S10 of the appendix.

Under the baseline scenario global spending attributable to dementia is estimated to reach $164 (95% UI $89–$272) per person in 2050 (Table 3). The scenario with accelerated increases in diagnosis and treatment rates led to estimated dementia spending $209 (95% UI $118–$334) globally. The scenario with accelerated nursing home-based care rates yielded spending estimates of $207 (95% UI $118–$332) per person, while the accelerated unit costs scenario estimated $255 (95% UI $154–$382) per person (Table 3).

**Table 3:** Estimated diagnosis and treatment rates, inpatient rates, and unit costs.

	Diagnosis and treatment rates 2019	Diagnosis and treatment rates 2050 (Baseline)	Diagnosis and treatment rates 2050 (Accelerated)	Nursing home-based care rates 2019	Nursing home-based care rates 2050 (Baseline)	Nursing home-based care rates 2050 (Accelerated)	Community-based care unit cost 2019	Community-based care unit cost 2050 (Baseline)	Community-based care unit cost2050 (Accelerated)	Nursing home-based care unit cost 2019	Nursing home-based care unit cost 2050 (Baseline)	Nursing home-based care unit cost 2050 (Accelerated)
Global	29% (25% - 33%)	43% (30% - 57%)	56% (46% - 67%)	23% (21% - 26%)	39% (28% - 52%)	43% (35% - 55%)	$3992 ($2787 - $5317)	$6443 ($3331 - $13 659)	$8389 ($4820 - $15 498)	$30 835 ($22 294 - $39 833)	$41 642 ($22 001 - $68 727)	$67 758 ($39 207 - $102 478)
World Bank Income Group												
High income	43% (37% - 51%)	57% (43% - 71%)	80% (72% - 86%)	36% (34% - 39%)	46% (37% - 57%)	74% (68% - 78%)	$5949 ($4336 - $7768)	$6560 ($3860 - $10 088)	$11 909 ($8464 - $15 860)	$40 858 ($29 541 - $53 003)	$64 381 ($34 731 - $107 267)	$121 758 ($79 861 - $168 171)
Upper middle income	24% (18% - 31%)	45% (22% - 70%)	55% (38% - 76%)	17% (15% - 19%)	38% (27% - 54%)	40% (32% - 54%)	$2406 ($1369 - $3735)	$7726 ($2840 - $19 031)	$8104 ($3296 - $19 335)	$7941 ($4056 - $12 344)	$28 058 ($11 067 - $61 067)	$31 207 ($13 580 - $63 586)
Lower middle income	14% (12% - 17%)	24% (15% - 37%)	32% (25% - 43%)	14% (12% - 15%)	28% (22% - 37%)	30% (25% - 38%)	$970 ($566 - $1399)	$1964 ($966 - $3611)	$2320 ($1287 - $3910)	$4151 ($2091 - $6327)	$10 685 ($5010 - $19 202)	$13 456 ($6758 - $22 259)
Low income	13% (11% - 14%)	19% (14% - 28%)	28% (23% - 35%)	8% (7% - 9%)	14% (11% - 17%)	16% (13% - 18%)	$577 ($337 - $831)	$783 ($427 - $1300)	$1151 ($658 - $1747)	$2514 ($1291 - $3773)	$5993 ($2723 - $10 052)	$8181 ($4011 - $13 153)
Global Burden of Disease Super Regions												
Central Europe, Eastern Europe, and Central Asia	25% (21% - 30%)	39% (25% - 61%)	54% (42% - 69%)	46% (41% - 51%)	69% (54% - 78%)	79% (78% - 80%)	$1929 ($1131 - $2773)	$2971 ($1530 - $5005)	$3943 ($2271 - $6069)	$7718 ($3870 - $11 790)	$15 833 ($7160 - $26 421)	$23 639 ($12 590 - $37 307)
High-income	44% (37% - 52%)	58% (42% - 74%)	82% (73% - 88%)	36% (34% - 39%)	46% (36% - 57%)	74% (68% - 79%)	$6078 ($4408 - $7924)	$6665 ($3836 - $10 375)	$12 222 ($8713 - $16 229)	$42 556 ($30 790 - $55 412)	$69 341 ($37 360 - $115 173)	$132 210 ($88 469 - $182 821)
Latin America and Caribbean	23% (18% - 28%)	30% (17% - 54%)	48% (38% - 64%)	15% (14% - 17%)	26% (20% - 35%)	32% (29% - 37%)	$1778 ($1019 - $2625)	$2232 ($1050 - $3997)	$3631 ($2077 - $5427)	$9523 ($4887 - $14 653)	$22 088 ($9389 - $39 412)	$30 366 ($15 248 - $47 744)
North Africa and Middle East	21% (17% - 25%)	32% (21% - 47%)	47% (37% - 58%)	13% (12% - 14%)	34% (28% - 43%)	35% (29% - 43%)	$1660 ($987 - $2390)	$3071 ($1617 - $5349)	$3775 ($2160 - $6130)	$7948 ($3929 - $12 222)	$21 787 ($9502 - $38 986)	$28 365 ($13 733 - $45 687)
South Asia	11% (8% - 15%)	18% (7% - 43%)	25% (16% - 44%)	13% (11% - 14%)	29% (21% - 39%)	29% (24% - 39%)	$685 ($379 - $1096)	$1137 ($409 - $2422)	$1456 ($761 - $2643)	$3343 ($1570 - $5549)	$10 395 ($3638 - $24 392)	$11 719 ($4994 - $24 532)
Southeast Asia, East Asia, and Oceania	23% (16% - 33%)	48% (18% - 80%)	55% (33% - 81%)	17% (15% - 19%)	38% (26% - 54%)	39% (31% - 54%)	$2597 ($1446 - $4209)	$8990 ($3001 - $22 127)	$9098 ($3353 - $22 223)	$8452 ($4258 - $13 736)	$31 209 ($10 702 - $72 656)	$33 377 ($13 560 - $73 829)
Sub-Saharan Africa	15% (13% - 17%)	22% (15% - 32%)	32% (27% - 40%)	12% (11% - 14%)	20% (16% - 26%)	23% (19% - 29%)	$791 ($460 - $1145)	$936 ($491 - $1646)	$1483 ($818 - $2312)	$5028 ($2409 - $7689)	$9435 ($4340 - $16 984)	$13 103 ($6752 - $21 355)

## Discussion

We modeled a broad set of input data and estimated direct global spending attributable to dementia was over $275 billion in 2019, at which time roughly a fourth of global cases were diagnosed and treated. Although more than 50% of dementia patients were cared for in the community, community-based care costs represent just 20% of global spending. Dementia spending is substantially higher in high-income countries, where modelled spending per person was over 550 times that of low-income countries, highlighting enormous disparities in diagnosis and treatment rates, nursing home-based care rates, and healthcare costs.

Our modelled estimates indicate that direct global cost of dementia care will reach $1.6 trillion (95% UI $0.9–$2.6) by 2050 under the baseline scenario, but total spending could be as high as $2.4 trillion (95% UI $1.5–$3.6)(not shown) under our most accelerated alternative scenario. This considerable variation in healthcare spending highlights the cost implications of policy choices and design of dementia care. In addition, escalating unit costs is the largest of the factors leading to these divergent amounts. It is important to emphasize that nursing home-based care and community-based care dementia costs are often funded out-of-pocket. Moreover, a great deal of care is provided by family members or part of the informal sector, and historically has not been included in national health budgets as the true extent of societal costs are hidden. As the number of people with dementia increases, governments need to design policies to maximize health, seek efficient care options, and cover the costs of providing care to this vulnerable population. Policy choices will determine what fraction of the expected health sector budget will be needed for dementia care. Policy makers should prioritize the health and financial wellbeing of dementia patients and caregivers, and while not included in this study, should also consider the broader societal costs of providing care for dementia patients. A key feature of this study is also the underlying increase in dementia cases, which, more than anything is driven by an aging global population. As life expectancy extends globally, broader consideration for how to fund the healthcare for an increasingly elderly population will be critical.

Our estimates of direct dementia spending as a portion of total health spending globally in 2019 (3% [95% UI 2–4%]) align quite well with previous estimates of spending, once adjusted for differences in the studies. Prince and Wimo,^2^ estimated $487.1 billion in spending globally in 2015, but their estimates are for healthcare spending on people with dementia, not healthcare spending attributable to dementia, which our study focused. Our estimates of total healthcare spending on people with dementia in 2015 is 7% (95% UI 5–10%) of global total health spending, or $508 billion (95% UI $389–$726) (not shown). Because dementia generally affects older adults, a large portion of dementia patients also likely suffer from other chronic health conditions that require care. We exclude from our primary estimates ‘excess’ spending on dementia patients attributable for other health conditions, which explains much of the difference between our and Prince and Wimo's estimates. One other important difference in our study and that of Prince and Wimo is that we do not assume that all prevalent cases were diagnosed and treated, while Prince and Wimo do. In our study, the estimated country-specific diagnosis and treatment rates ranged from above 50% in Finland, Norway and United Kingdom, to below 10% in countries such as Somalia, Pakistan and Bangladesh. Despite these differences, we find very similar spending estimates at the global level, and reach many of the same important qualitative conclusions.

The estimates reported in this work only represent direct spending attributable to dementia. Therefore, they should be interpreted as a lower bound of global dementia spending. As previously mentioned dementia care often consists informal care provided by family members, and falls outside of the measured costs.

The World Health Organization (WHO) Action Plan on the Public Health Response to Dementia 2017–2025 recommends that people with dementia be treated in the community, rather than in nursing home settings, in order to receive care that coincides with their preferences and to protect their rights and dignity across the continuum of dementia care.^27^ In order to provide the quality care that dementia patients deserve, policies, healthcare providers, and support systems are needed for caregivers and patients alike. Indeed, among the WHO Action Plan's seven action areas and targets is area 5: support for dementia carers.^27^ It is crucial to support caregivers develop skills to cope with the challenging behaviours often associated with dementia to improve patients’ quality of life and to prevent their own stress and health problems.^27^ Developing or strengthening policies to protect carers is a fundamental aspect of providing care for patients with dementia in the community, policy makers need to prioritize social and disability benefits for caregivers as well as policies and legislation against discrimination.^21^ The WHO and World Bank have estimated that 40 million new health and social care jobs will be needed by 2030 alongside 18 million additional health workers needed to serve this population.^27^ Given the expected increase in dementia prevalence in the near future it is critical to emphasize training clinicians and healthcare workers to identify patients suffering from dementia and provide them with adequate care to help them stay in the community as long as possible. Investing in the necessary training and support for caretakers can enable governments to ensure quality care is provided in the community, where our results show costs are lower. Some countries have successfully begun to embark on plans in line with WHO suggestions: Israel, Italy, Malta, Norway, Finland, and Wales have national dementia strategies in which care for dementia patients in the community is supported by multi-disciplinary teams.^28^ This strategy supports patients and caregivers, allowing them to live at home and incurring lower costs. By 2050, the expected financial burden on healthcare systems in low- and middle-income countries is likely to be substantial, particularly in regions like Southeast Asia, East Asia, and Oceania, where the growth rates in dementia spending have historically been highest. The financial and human resources requirements of the increasing dementia burden in low- and middle-income countries are large, and these countries will need to monitor cost growth over time, given the potential for exponential increases. All countries need to prepare to provide adequate care for dementia patients and ensure they have the necessary resources budgeted.

This study is subject to several limitations. First and foremost, this study is fundamentally a modelling analysis, as we have a relatively small dataset that is not randomly distributed globally, as there is very little data available from low-and middle-income countries on diagnosis and treatment rates, nursing home-based care versus community-based care rates, or unit costs. Although ST-GPR modelling addresses data sparsity, increased data collection from these countries would improve the accuracy of our estimates. We attempted to capture variability among countries by incorporating country specific information related to population, dementia prevalence, socio-demographic level, and total health spending from the GBD into our model. While our estimates of dementia spending are highly modelled and should be interpreted as such, they were produced with all of the data available and serve to highlight the expected increases in resources for dementia, as well as the urgent need to collect more data on this very relevant topic. Another limitation is the difficulty of distinguishing costs strictly attributable to dementia from those caused by other comorbidities associated with older populations. Our estimates of the fraction of health spending on dementia patients attributable to dementia do not vary across countries or time because of a lack of data, even though it is likely that in reality there might be some heterogeneity (see Fig. 1), and our estimates of the attributed spending rate for undiagnosed dementia patients in nursing facilities is merely assumed to be half of that of the diagnosed population. While this assumption makes intuitive sense (and sensitivity analyses have been reported in the appendix) there is no evidence that this ratio is correct. The lack of data necessitated the assumption that there is no direct spending attributable to dementia for patients with undiagnosed dementia who live in the community. We are aware this might not be entirely accurate, but were unable to capture spending for this population due to the lack of data on this topic, although we do not expect this spending to be large relative to spending on diagnosed patients and spending on those in nursing facilities. This work also combines costs associated with treating Alzheimer's disease with those associated with other dementias because the underling literature and distinction between the two in the underlying studies was not robust. An additional limitation of our work is that it only includes direct healthcare cost attributable to dementia without quantifying the economic burden of informal care or the cost of care for other diseases that may have been caused by dementia. Given that a large fraction of the care provided to patients with dementia is informal, especially in countries that do not rely heavily on nursing care facilities, it is important in the future to develop innovative methods to quantify indirect costs to truly understand the economic burden of dementia around the world. This is especially important in the context of the WHO Action Plan on the Public Health Response to Dementia 2017–2025 where community-based care is encouraged to provide care aligned with the wishes and preferences of people with dementia in addition to helping them preserve their autonomy and rights.^21^ A limitation of our future dementia spending modelling approach is that none of the future scenarios considers the development of new dementia treatments and prevention strategies that could lower the direct cost of caring for someone with dementia in the future. Our future scenarios reflect the trends in dementia prevalence, care, and costs observed in the literature over the last several decades, and we opted to not in include a “better” scenario that illustrated potential reductions in spending, as the majority of countries saw increases in spending between 2000 and 2019. Finally, although we included data from sources in multiple languages into our sample the search terms for the systematic reviews were completed in English alone.

As life expectancy continues to improve and dementia diagnosis and treatment rates increase, the financial cost associated with dementia is likely to increase dramatically, particularly in low- and middle-income countries. Meeting the healthcare needs of this growing population will have major implications in these countries. While costs of dementia care are woefully recorded, especially in low-income countries, dementia will increasing cost a great deal in all countries. Policymakers can use the modelled estimates presented here when advocating for financially sustainable and appropriate care for all who are affected by Alzheimer's and other dementias. Dementia must become a policy priority in line with the WHO Action Plan; aiming to preserve the quality of life of individuals with dementia and their caregivers.

### Funding

Gates Ventures.

### Authors’ contributions

J L Dieleman and P Pedroza Velandia were responsible for methods development. C Chen, M K Miller-Petrie, and P Pedroza Velandia prepared the first draft. S Chakrabarti and P Pedroza Velandia contributed to data extraction and analysis. S Chakrabarti, C Chen, J L Dieleman, and P Pedroza Velandia accessed and verified the data. C Chen, J L Dieleman, S Hay, M K Miller-Petrie, P Pedroza Velandia, and A Wimo all contributed to subsequent iterations of the draft. A Chapin, Joseph L Dieleman, and G Tsakalos provided project management. All authors contributed to the framing of the research approach, reviewed results, or reviewed and contributed to the final manuscript. J L Dieleman is responsible for the decision to submit this manuscript.

### Data sharing statement

Data used for this study were extracted from publicly available sources that are listed in the appendix. Further details will be made available on the Global Health Data Exchange website.

## Declaration of interests

J L Dieleman reports support from Gates Ventures. A Wimo reports support from WHO, Merk, and Gates Ventures to his institution and private payments from RUD Instrument and Eisai, all outside submitted work. All other authors declare no competing interests.

## Appendix Supplementary materials

Image, application 1
