# Supplementary material for: Global and regional spending on dementia care from 2000–2019 and expected future health spending scenarios from 2020–2050: An economic modelling exercise
Source: eClinicalMedicine. 2022 Mar 13;45:101337. doi: 10.1016/j.eclinm.2022.101337 (PMC8921543; doi:10.1016/j.eclinm.2022.101337)
Supplement: Supplementary file 1 [file mmc1.pdf]

Methods and data appendix

Global and regional spending on dementia care from 2000-2019 and expected future health spending scenarios from 2020-2050: an economic modelling exercise

Authors: Paola Pedroza Velandia, MPH
Molly K Miller-Petrie, MSc
Carina Chen, MA
Suman Chakrabarti, MA
Abigail Chapin, BA
Prof Simon Hay, DSc
Golsum Tsakalos, MS
Anders Wimo, MD
Joseph L Dieleman, PhD

Correspondence: Joseph L Dieleman
dieleman@uw.edu
206-897-3840
2301 5th Ave, Suite 600
Seattle, WA 98121

Version: January 22, 2022

Table of Contents

Literature reviews 3

1. Dementia diagnosis and treatment literature review 3
2. Dementia unit cost literature review 7

Analytical framework and methods 21

Results 27

Sensitivity analyses 81

Literature reviews

1. Dementia diagnosis and treatment literature review

Not all people affected with dementia receive adequate diagnosis and treatment. This is observed in low and middle-income countries as well as high-income countries. We turned to the literature to gather a robust data set that would help us develop a model to better understand dementia diagnosis and treatment rates and how they differ around the world. We extracted diagnosis and treatment rates in community-based care and nursing-home-based care settings.

Dementia diagnosis and treatment rates literature review search terms

Date	Database	Search terms
May 25, 2021	PubMed	(((Dementia Mesh) AND Long-Term Care Mesh AND coverage)))
		((Alzheimer's disease) AND treatment rates) AND outpatient
		(Ambulatory Care) AND Insurance, Long-Term Care
		(Inpatients Mesh) AND (Dementia Mesh OR Alzheimer Disease Mesh)
		Dementia AND (Ambulatory Care Mesh)

Dementia diagnosis and treatment rates literature review flow diagram

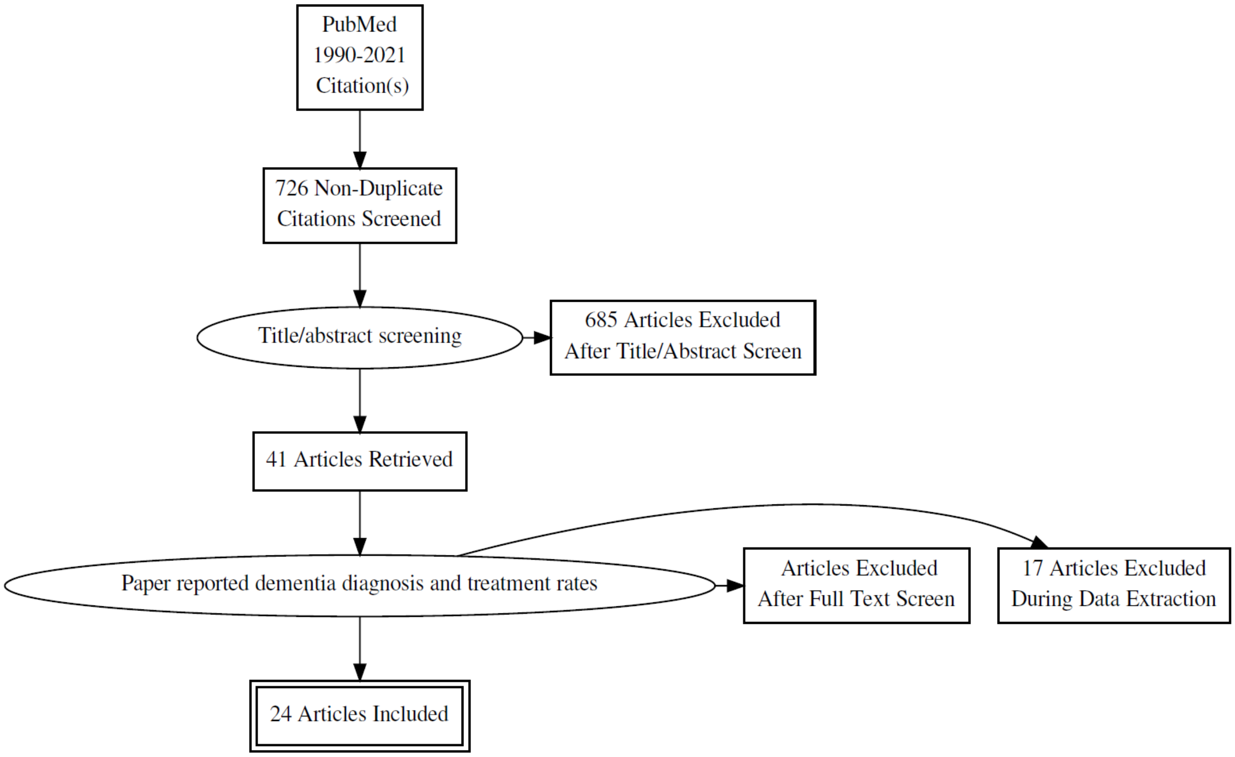

Table S1 Dementia diagnosis and treatment rates bibliography

The papers in the table below are those from which data on diagnosis and treatment rates was extracted. These papers were selected by applying the methods mentioned above. As previously mentioned, we extracted dementia diagnosis rates by care setting.

Publication Year	Title	Authors
1986	The diagnosis and differential diagnosis of dementia.	Ross GW1, Bowen JD.
1989	Prevalence of medically diagnosed dementia in a defined United States population: Rochester, Minnesota, January 1, 1975	E Kokmen, CM Beard, KP Offord, et al.
1990	The Gospel Oak Study, Stage II: the diagnosis of dementia in the community	G Livingston, K Sax, J Willison, et al.
2000	Undetected dementia in community-dwelling older people: The Canadian Study of Health and Aging.	Sternberg SA, Wolfson C, Baumgarten M.
2000	Detection of dementia in primary care: the Linkˆping study.	OlafsdÛttir M1, Skoog I, Marcusson J.
2003	Missing the diagnosis: senile dementia in patients admitted to nursing homes.	Nygaard HA1, Ruths S.
2003	The recognition of dementia in ënon-EMIí nursing home residents in South East England.	MacDonald AJD, Carpenter GI.
2004	Dementia Care in 9 OECD Countries: A Comparative Analysis	Pierre Moise, Michael Schwarzinger, Myung-Yong Um and the Dementia Expertsí Group
2004	The relationship between a dementia diagnosis, chronic illness, medicare expenditures, and hospital use	Bynum JP, Rabins PV, Weller W, et al.
2004	Physician recognition of cognitive impairment: Evaluating the need for improvement	Chodosh J, Petitti DB, Elliott M, et al.
2004	Donepezil use in US nursing homes.	Pedone C1, Lapane KL, Mor V, Bernabei R.
2005	Use of Psychotropic Drugs in Elderly Nursing Home Residents with and without Dementia in Helsinki, Finland	Helka Hosia-Randell1 and Kaisu Pitk®al®a2
2007	Under-diagnosis of common chronic diseases: prevalence and impact on human health	M. E. Falagas,1,2 K. Z. Vardakas,1 P. I. Vergidis1
2007	Access to diagnostic evaluation and treatment for dementiain Europe	Gunhild Waldemar1*, Kieu T. T. Phung1, Alistair Burns2, Jean Georges3, Finn Ronholt Hansen4,Steven Iliffe5, Christine Marking6, Marcel Olde Rikkert7, Jacques Selmes9, Gabriela Stoppe8andNorman Sartorius10on behalf of the European Dementia Consensus Network (EDCON)
2007	Dementia: international comparisons. Summary report for the National Audit Office.	Knapp M, Comas-Herrera A, Somani A et al
2007	Dementia undiagnosed in poor older adults with functional impairment	Wilkins CH, Wilkins KL, Meisel M, Depke M, Williams J, Edwards DF.

2008	The Effectiveness of a Home Care Program for Supporting Caregivers of Persons with Dementia in Developing Countries: A Randomised Controlled Trial from Goa, India	Amit Dias1*, Michael E. Dewey2, Jean DíSouza3, Rajesh Dhume4, Dilip D. Motghare1, K. S. Shaji5, Rajiv Menon6, Martin Prince2, Vikram Patel7
2009	Closing the treatment gap for dementia in India	Amit Dias, Vikram Patel1
2009	Dementia in the acute hospital: prospective cohort study of prevalence and mortality	Elizabeth L. Sampson, Martin R. Blanchard, Louise Jones, Adrian Tookman and Michael King
2009	The underdetection of cognitive impairment in nursing homes in the Dublin area. The need for on-going cognitive assessment	SUZANNE CAHILL1,2,*, ANA M. DIAZ-PONCE3, ROBERT F. COEN4, CATHAL WALSH5 1Dementia Services Information and Development Centre (DSIDC),
2009	Cholinesterase Inhibitor Use in U.S. Nursing Homes: Results from the National Nursing Home Survey	Dallas P. Seitz, MD,wz Andrea Gruneir, PhD,wz David K. Conn, MB, and Paula A. Rochon, MD,wz
2010	New diagnoses of dementia among older patients admitted to postacute care.	Ferretti M, Seematter-Bagnoud L,Martin E, B¸la CJ.
2011	Underdiagnosis of dementia in primary care: Variations in the observed prevalence and comparisons to the expected prevalence	Amanda Connollya, Ella Gaehla, Helen Martinb, Julie Morrisc and Nitin Purandarea*
2011	Estimating the prevalence of dementia: cognitive screening in Glasgow nursing homes.	Lithgow S1, Jackson GA, Browne D.
2011	Public financial support receipt and non-medical resource utilization in Alzheimer's disease results from the PLASA study	Rapp T1, Grand A, Cantet C, Andrieu S, Coley N, Portet F, Vellas B.
2011	Patterns of Dementia Treatment Use in Assisted Living Facilities: A Cross-Sectional Study of 1975 Demented Residents	Nade`ge Barro-Belaygues, MD, Gabor Abellan van Kan, MD, Yves Rolland, MD, PhD, Fati Nourhashemi, MD, PhD, Maria Soto-Martin, MD, Sophie Gillette-Guyonnet, PhD, and Bruno Vellas, MD, PhD
2012	Underrecognition and Undertreatment of Dementia in Italian Nursing Homes	Antonio Cherubini MD, PhD a,b,*, Carmelinda Ruggiero MD, PhD b, Giuseppina DellíAquila MDb, Paolo Eusebi PhD c, Beatrice Gasperini MDb, Elisa Zengarini MDb, Annarita Cerenzia MDb, Giovanni Zuliani MD, PhD d, Antonio Guaita MDe, Fabrizia Lattanzio MD, PhD f
2012	Under-recognition of dementia in long-term care homes in Hong Kong	Sheung-Tak Chenga*, Linda C.W. Lamb and Pizza K. Chowa
2012	Formal and informal care for disabled elderly living in the community: an appraisal of French care composition and costs	Alain Paraponaris, BÈrengËre DavinPierre Verger
2013	Dementia: a state of the nation report on dementia care and support in England	Department of Health, UK.
2014	Rates of formal diagnosis in people screened positive for dementia in primary care: Results of the DelpHi-trial.	Eichler T, Thyrian JR, Hertel J, et al.
2015	Dementia underdiagnosis in Brazil	Antonio Eduardo Nakamura, Davi Opaleye, Giovanni Tani, Cleusa P. Ferri
2015	Diagnosis of dementia by medical practitioners: a national study among older adults in Singapore	Siow Ann Chonga*, Edimansyah Abdina, Janhavi Vaingankara, Li Ling Ngb and Mythily Subramaniama
2015	Prevalence of Dementia in People Aged 60 Years and Above: Results from the WiSE Study	Mythily Subramaniama,1,?, Siow Ann Chonga,1, Janhavi Ajit Vaingankara, Edimansyah Abdina

2015	Who has undiagnosed dementia? A cross-sectional analysis of participants of the Aging, Demographics and Memory Study.	Savva GM, Arthur A.
2016	Calidad del registro del diagnÛstico de demencia en atenciÛn primaria. La situaciÛn en Espaòna en el periodo 2002-2011	MarÌa del Canto de Hoyos-Alonsoa, , Julio Bonisb, VerÛnica Bryantb, MarÌa Victoria Castell Alcal·c,d y ¡ngel Otero Puimed,e
2016	Alzheimerís and other dementias in Canada, 2011 to 2031: a microsimulation Population Health Modeling (POHEM) study of projected prevalence, health burden, health services, and caregiving use	Douglas G. Manuel, Rochelle Garner, Philippe FinËs, Christina Bancej, William Flanagan, Karen Tu, Kim Reimer, Larry W. Chambers and Julie Bernier
2016	Potentially unsafe activities and living conditions of older adults with dementia	Amjad H, Roth DL, Samus QM, Yasar S, Wolff JL.
2017	Prevalence and determinants of undetected dementia in the community: A systematic literature review and a meta-analysis.	Lang L, Clifford A, Wei L, et al.
2018	Underdiagnosis of Dementia: an Observational Study of Patterns in Diagnosis and Awareness in US Older Adults	Halima Amjad, MD, MPH1,2, David L. Roth, PhD1,2, Orla C. Sheehan, MD, PhD1,2, Constantine G. Lyketsos, MD, MHS3, Jennifer L. Wolff, PhD2,4, and Quincy M. Samus, PhD2,3
2018	Comprehensive cost of illness of dementia in Japan: a time trend analysis based on Japanese official statistics	SHIMPEI HANAOKA, KUNICHIKA MATSUMOTO, TAKEFUMI KITAZAWA,, SHIGERU FUJITA, KANAKO SETO,and TOMONORI HASEGAWA

2. Dementia unit cost literature review

The cost of treating dementia varies greatly across geographies. We turned to the literature to extract data on the cost of treating dementia in nursing-home-based care and community-based care settings around the world. The goal was to construct a robust data set with data from low and middle-income countries as well as high-income countries on the cost of treating patients with dementia in community-based care and nursing-home-based care settings.

Dementia unit cost literature review search terms

Date	Database	Search terms
May 25, 2021	PubMed	Alzheimer Disease[MH] AND (Costs and Cost Analysis[MH] OR Health Spendings[MH] OR Cost of Illness[MH]) AND 1990 : 2021[DP]

Dementia unit cost literature review flow diagram

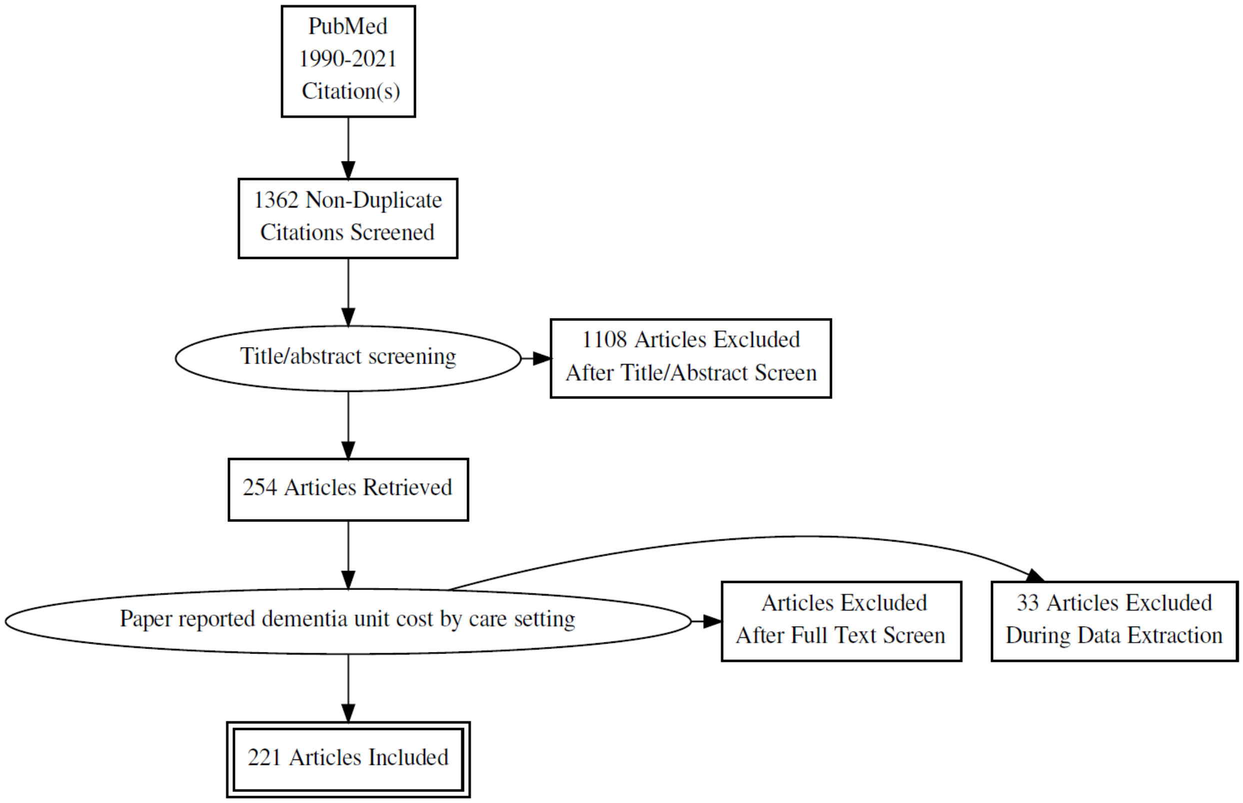

Table S2 Dementia unit costs bibliography

The papers in the table below are those that after going through our literature review process meet our requirements for data extraction. From these papers, we extracted the cost of treating patients with dementia in community-based care and nursing-home-based care settings in low and middle-income countries as well as high-income countries. When available, attributable fractions were also extracted from the papers below.

Publication year	Title	Authors
1992	The Cost of Institutional Care in Alzheimer's Disease: Nursing Home and Hospital Use in a Prospective Cohort	H. Gilbert Welch, John S. Walsh, Eric B. Larson
1993	Alzheimer's disease: the burden of the illness in England.	Gray A , Fenn P
1993	The economic burden of Alzheimer's disease care.	Rice DP, Fox PJ, Max W
1994	The US economic and social costs of Alzheimer's disease revisited.	Ernst RL, Hay JW.
1995	Alzheimer's disease. The unpaid burden of caring.	Max W, Webber P, Fox P.
1995	Economic analysis of Alzheimer's disease in outpatients: impact of symptom severity	SouÍtre EJ, Qing W, Vigoureux I
1996	The economic burden of Alzheimer's disease in Israel.	Rothstein Z , Prohovnik I , Davidson M ,
1997	Cavallo 1997 ADAD the economic & social burden of AD on families in Italy	Cavallo
1998	Alzheimer's Disease Care: Costs And Potential Savings	Joel Leon, Chang-Kuo Cheng, and Peter J. Neumann
1998	Alzheimer's disease under managed care: implications from Medicare utilization and expenditure patterns.	Weiner M, Powe NR, Weller WE, Shaffer TJ, Anderson GF
1998	Cognitive Function and the Costs of Alzheimer Disease An Exploratory Study	Richard L. Ernst; Joel W. Hay; Catharine Fenn
1998	Economic considerations in Alzheimer's disease	Meek PD, McKeithan K, Schumock GT
1999	Andersen 1999 DGCD the cost of dem in Denmark the odense study	Andersen
1999	Boada 1999 MC costs AD Spain	Boada
1999	Chiu 1999 JAN cost comparisons between family based care and nursing home care for dementia in Taiwan	Chiu
1999	Economic impact of Alzheimer's disease in the United Kingdom: Cost of care and disease severity for non-institutionalised patients with Alzheimer's disease	SouÍtre E, Thwaites RM, Yeardley HL.
1999	Leon 1999 AJMC the cost of AD in managed care USA	Leon
1999	ScuveeMoreau 1999 ANB the economic impact of dementia in Belgium	ScuveeMoreau
1999	The Economic Cost of Alzheimer's Disease and Related Dementias to the California Medicaid Program (ìMedi-CalÎ) in 1995	Menzin J, Lang K, Friedman M, Neumann P, Cummings JL.
2000	The economic and social cost of dementia in Ireland.	O'Shea E, O'Reilly S.
2000	Dementia: the cost of care for behaviourally disturbed patients living in the community	Kirchner V, Elloy MD, Silver LE, Kelly CA.
2000	Medicare expenditures associated with Alzheimer disease	Robert L. Kane; Adam Atherly
2000	Potential savings in the cost of caring for Alzheimer's disease. Treatment with rivastigmine.	Hauber AB, Gnanasakthy A, Snyder EH
2000	Model of costs of care for dementia: community-dwelling vs. institutionalization.	van der Roer N, Goes ES, Blom M, Busschbach JJ.
2001	Alzheimer disease: diagnosis, costs, and dimensions of treatment	DeKosky, ST; Orgogozo, JM

2001	Alzheimer's disease in the UK: comparative evidence on cost of illness and volume of health services research funding	Lowin, A; Knapp, M; McCrone, P
2001	Estimation of the family cost of private nursing home care versus home care for patients with dementia in Taiwan	Chiu, L; Shyu, WC
2001	Healthcare utilization and costs in managed care patients with Alzheimer's disease during the last few years of life	McCormick, WC; Hardy, J; Kukull, WA; Bowen, JD; Teri, L; Zitzer, S; Larson, EB
2001	How much does it really cost to care for persons with Alzheimer's disease?	Brummel-Smith, K
2001	Prevalence, Costs, and Treatment of Alzheimer's Disease and Related Dementia: A Managed Care Perspective	Dorothy P. Rice; Howard M. Fillit; Wendy Max
2001	Residential and nursing home care of elderly people with cognitive impairment: prevalence, mortality and costs	Netten A, Darton R, Bebbington A
2001	The costs of caring: medical costs of Alzheimer's disease and the managed care environment	Murman, DL
2002	Alzheimer's disease and related dementias increase costs of comorbidities in managed Medicare	Hill, JW; Futterman, R; Duttagupta, S; Mastey, V; Lloyd, JR; Fillit, H
2002	Costs of dementia	Bianchetti, A; Castelletti, F; Trabucchi, M
2002	Economic cost of Alzheimer disease in Israel	Beeri, MS; Werner, P; Adar, Z; Davidson, M; Noy, S
2002	Effect of Alzheimer disease on the cost of treating other diseases	Sloan, FA; Taylor, DH
2002	Health care utilization and costs of Alzheimer's disease: the role of co-morbid conditions, disease stage, and pharmacotherapy	Fillit, H; Hill, JW; Futterman, R
2002	Primary care expenditures before the onset of Alzheimer's disease	Albert, SM; Glied, S; Andrews, H; Stern, Y; Mayeux, R
2002	The cost of behavioral and psychological symptoms of dementia (BPSD) in community dwelling Alzheimer's disease patients.	Beeri MS,†Werner P,†Davidson M,†Noy S.
2002	The costs of vascular dementia: a comparison with Alzheimer's disease	Fillit, H; Hill, J
2002	The impact of symptom severity on the cost of Alzheimer's disease	Small, GW; McDonnell, DD; Brooks, RL; Papadopoulos, G
2002	The incremental direct costs associated with behavioral symptoms in AD	Murman, DL; Chen, Q; Powell, MC; Kuo, SB; Bradley, CJ; Colenda, CC
2003	An Estimate of the Worldwide Prevalence and Direct Costs of Dementia in 2003	A. Wimo L. Jonsson B. Winblad
2003	Assessment of health economics in Alzheimer's disease (AHEAD): treatment with galantamine in the UK	Ward, A; Caro, JJ; Getsios, D; Ishak, K; O'Brien, J; Bullock, R; AHEAD Study Group
2003	Brodaty 2003 Nat AE economic impact of Dem in Australia	Brodaty
2003	Economic evaluation of galantamine in the treatment of mild to moderate Alzheimer's disease in the United States	Migliaccio-Walle, K; Getsios, D; Caro, JJ; Ishak, KJ; O'Brien, JA; Papadopoulos, G; AHEAD Study Group
2003	Patients with Alzheimer's disease living at home in France: costs and consequences of the disease	Rigaud, AS; Fagnani, F; Bayle, C; Latour, F; Traykov, L; Forette, F
2003	Resource utilisation and cost analysis of memantine in patients with moderate to severe Alzheimer's disease.	Stoffler A, Wirth Y, Mobius HJ.
2003	The economics of Alzheimer disease	Leung, GM; Yeung, RY; Chi, I; Chu, LW
2004	A dependency model for patients with Alzheimer's disease: its validation and relationship to the costs of care ñ the LASER-AD Study	Katona C, Roch B, Guilhaume C, Rive B.
2004	A dependency model for patients with Alzheimer's disease: its validation and relationship to the costs of care--the LASER-AD Study	Livingston, G; Katona, C; Roch, B; Guilhaume, C; Rive, B
2004	Cost effects of a specialized care center for people with Alzheimer's disease	Bloom, BS; Chhatre, S; Jayadevappa, R
2004	Moise 2004 OECD dementia care in 9 OECD countries a comparative analysis	Moise

2004	Out-of-pocket health care expenditures among older Americans with dementia	Langa, KM; Larson, EB; Wallace, RB; Fendrick, AM; Foster, NL; Kabeto, MU; Weir, DR; Willis, RJ; Herzog, AR
2004	Variation in cost of informal caregiving and formal-service use for people with Alzheimer's disease	Harrow, BS; Mahoney, DF; Mendelsohn, AB; Ory, MG; Coon, DW; Belle, SH; Nichols, LO
2004	Cost relation between severity of Alzheimer's disease and cognitive and functional impairment	LÛpez-Pousa, S; Garre-Olmo, J; Turon-Estrada, A; Hern·ndez, F; ExpÛsito, I; Lozano-Gallego, M; Hern·ndez-Ferr·ndiz, M; Gelada-Batlle, E; Pericot-Nierga, I; Vilalta-Franch, J
2004	Costs study in Alzheimer's disease	Atance MartÌnez, JC; Yusta Izquierdo, A; Grupeli Gardel, BE
2005	Cost of Alzheimer's disease in a developing country setting	Zencir, M; Kuzu, N; Beser, NG; Ergin, A; Catak, B; Sahiner, T
2005	J-nsson et al-2005-European Journal of Neurology	J-nsson
2005	Prevalence, resource utilization and costs of vascular dementia compared to Alzheimer's dementia in a population setting	Sicras, A; Rejas, J; Arco, S; Flores, E; Ortega, G; Esparcia, A; Su·rez, A; Gordillo, MJ
2005	The cost of severe dementia	JoÎl, ME
2006	Clinical Characteristics and Longitudinal Changes of Informal Cost of Alzheimerís Disease in the Community	Torgan R, Albert M, Brandt J, Blacker D, et al.
2006	Determinants of costs of care for patients with Alzheimer's disease	Niels Andreasen; Bengt Winblad; Anders Wimo
2006	Longitudinal study of effects of patient characteristics on direct costs in Alzheimer disease	Albert M, Brandt J, Blacker D, et al.
2006	Social-economic costs and quality of life of Alzheimer disease in the Canary Islands, Spain	Pedro Serrano-Aguilar; Lilisbeth Perestelo-Perez; Juan Oliva-Moreno
2006	Social-economic costs and quality of life of Alzheimer disease in the Canary Islands, Spain.	Lopez-Bastida, Serrano-Aguilar P, Perestelo-Perez L
2006	The economic costs of dementia in Korea 2002	Suh
2006	Longitudinal study of effects of patient characteristics on direct costs in AD USA	Zhu
2007	Australia2007 AUS inst of health and welfare 2006 Dementia in Australia	Australia2007
2007	Dementia: international comparisons. Summary report for the National Audit Office	Martin Knapp, Adelina Comas-Herrera, Ami Somani
2007	Economic cost of dementia patients according to the limitation of the activities of daily living in Korea	Im Ok Kang, Sang-Yi Lee, Su Young Kim
2007	Economic impact of dementia in developing countries: an evaluation of costs of Alzheimer-type dementia in Argentina	Allegri RF, Butman J, Arizaga RL, Machnicki G
2007	Economic impact of dementia in developing countries: an evaluation of costs of Alzheimer-type dementia in Argentina	Fernando E Taragano; Diego Sarasola; Leandro Lon
2007	Dementia UK Full Report 2007	Knapp
2008	2008 Alzheimer's disease facts and figures	Alzheimer's Association
2008	Alzheimer's disease and managed care: a convincing case for action	Stephen Gorshow
2008	Cost-effectiveness analysis of donepezil for mild to moderate Alzheimerís disease in Taiwan	Jong-Ling Fuh and Shuu-Jiun Wang
2008	Disability in Alzheimer's disease: causes, consequences, and economic considerations	George J Demakis
2008	Evaluated need, costs of care, and payer perspective in degenerative dementia patients cared for in the United States	Paula R Sherwood; Jersey Liang; Christopher C Colenda
2008	Healthcare costs and utilization for Medicare beneficiaries with Alzheimer's	Sharada Weir; Marilyn S Kramer; Arlene S Ash

2008	Home health and informal care utilization and costs over time in Alzheimer's disease	Deborah Blacker; Mary Sano; Yaakov Stern
2009	Costs of the in-home patients affected by dementia.	Isaia G, M Bo, Nobili G, Cappa G, Mondino S, Pilon S, Massaia M.
2009	2009 Alzheimer's disease facts and figures	Alzheimer's Association
2009	Comparison of costs of care between patients with Alzheimer's disease and dementia with Lewy bodies	Deborah Blacker; Mary Sano; Yaakov Stern
2009	Economic impact of dementia in developing countries: an evaluation of Alzheimer-type dementia in Shanghai, China	Yu-Lei Deng; Wei Xu; Ying Wang; Sheng-Di Chen
2009	Implications of comorbidity on costs for patients with Alzheimer disease	Sharada Weir; Marilyn Schlein Kramer; Arlene S Ash
2009	Modelling costs of dementia in Australia: evidence, gaps, and needs	Geetha Ranmuthugala; Laurie Brown; Marc Budge
2009	Patient dependence and longitudinal changes in costs of care in Alzheimer's disease	Deborah Blacker; Mary Sano; Yaakov Stern
2009	Private costs almost equal health care costs when intervening in mild Alzheimer's: a cohort study alongside the DAISY trial	Ane Eckermann; Dorthe V Buss; Gunhild Waldemar
2009	The cost of dementia in Europe: a review of the evidence, and methodological considerations	Linus Jˆnsson; Anders Wimo
2009	The effects of patient function and dependence on costs of care in Alzheimer's disease	Deborah Blacker; Mary Sano; Yaakov Stern
2010	Costs of dement in Hungary	rsek2010
2010	2010 Alzheimer's disease facts and figures	Alzheimer's Association
2010	Assessing the relationship between health utilities, quality of life, and health care costs in Alzheimer's disease: the CATIE-AD study	E A Miller; R A Rosenheck; L S Schneider
2010	Cost of dementia in Switzerland	; Michael Marti; Sarah Werner; Heini Sommer
2010	Cross sectional observational study on the societal costs of Alzheimer's disease	S Langworth; B Winblad; L Jˆnsson
2010	Cross sectional observational study on the societal costs of Alzheimer's disease.	Mesterton J, Wimo A, By A, Langworth S, Winblad B, Jˆnsson L.
2010	Differences in resource use and costs of dementia care between European countries: baseline data from the ICTUS study.	Reynish E, Ousset PJ, Andrieu S, et al
2010	Dementia2010 UK	LuengoFernandez
2010	Prospective one-year cost-of-illness study in a cohort of patients with dementia of Alzheimer's disease type in Spain: the ECO study	Juan Del Llano; Teresa LeÛn; Javier Rejas
2010	Cost of illness in Alzheimer's disease	Reinhard Rychlik; Christine Grimm; Dietmar Daniel
2011	Costs of care for dementia patients in community setting: an analysis for mild and moderate disease stage.	Schwarzkopf L, Menn P, Kunz S, Holle R, Lauterberg J, Marx P, Mehlig H, Wunder S, Leidl R, Donath C, Graessel E.
2011	Net costs of dementia by disease stage.	Leicht H, Heinrich S, Heider D, Bachmann C, Bickel H, van den Bussche H, Fuchs A, Luppa M, Maier W, M√∂sch E, Pentzek M, Rieder-Heller SG, Tebarth F, Werle J, Weyerer S, Wiese B, Zimmermann T, K√∂nig HH; AgeCoDe study group..
2011	2011 Alzheimer's disease facts and figures	Alzheimer's Association
2011	A review of the methods used to estimate the cost of Alzheimer's disease in the United States	Josephine Mauskopf; Lisa Mucha
2011	A systematic review to assess the policy-making relevance of dementia cost-of-illness studies in the US and Canada	Mark Oremus; S Carolina Aguilar
2011	Clinical and economic characteristics associated with direct costs of Alzheimer's, frontotemporal and vascular dementia in Argentina	ecilia M Serrano; Monica Iturry; Ricardo F Allegri

2011	Cost of disorders of the brain in Europe 2010	Gustavsson A, Svensson M, Jacobi F
2011	Costs of care in a mild-to-moderate Alzheimer clinical trial sample: Key resources and their determinants	Gustavsson A, Cattelin F, Jonsson L.
2011	Costs of informal care for people suffering from dementia	Reiche T, Nissen NP, Gundgaard J
2011	Direct costs of Alzheimer's disease in Germany	Dietmar Daniel; Christine Grimm; Reinhard Rychlik
2011	Direct medical costs in patients with Alzheimer's disease in Taiwan: A population-based study	Chan AL, Cham TM, Lin SJ.
2011	Net costs of dementia by disease stage Germany	Leicht
2011	Predictors of costs of care in Alzheimerís disease: A multinational sample of 1222 patients	Bergvall N, Kolasa K, Wimo A, Winblad B, et al.
2011	Relative importance of patient disease indicators on informal care and caregiver burden in Alzheimer's disease	Gustavsson A, Wimo A, Winblad B
2011	The relation between disease severity and cost of caring for patients with Alzheimer disease in Canada	Robert Sambrook; Nadia Lesnikova; Krista L LanctÙt; Canadian Outcomes Study in Dementia (COSID) Investigators
2012	A longitudinal analysis of the lifetime cost of dementia.	Yang Z, Zhang K, Lin PJ, Clevenger C, Atherly A.
2012	Excess costs of dementia disorders and the role of age and gender - an analysis of German health and long-term care insurance claims data.	Schwarzkopf L, Menn P, Leidl R, Wunder S, Mehlig H, Marx P, Graessel E, Holle R.
2012	Schwarzkopf vih cost of care for dementia patients in community setting Germany	Schwarzkopf
2012	2012 Alzheimer's disease facts and figures	Alzheimer's Association
2012	An economic evaluation of early assessment for Alzheimer's disease in the United Kingdom	Khajak J Ishak; Grant Maclaine; Luis Hern·ndez
2012	Comorbidity burden, health care resource utilization, and health care costs among Medicare Advantage members with Alzheimer's disease.	Suehs B, Davis C, Shah S
2012	Comparison of the impact of Alzheimer's Disease on Medicare and Medicaid.	Mucha L, Forys A, Shih H-C
2012	Cost and care of patients with Alzheimer's disease: clinical predictors in German health care settings	Frank Jessen; Monika-Balzer Geldsetzer; Richard Dodel
2012	Deloitte Updated Dementia Economic Impact Report 2012 New Zealand	Deloitte
2012	Dementia and Out-of-Pocket Spending on Health Care Services	Adeline D., Michael H., Francisco M.
2012	Disease progression and costs of care in Alzheimer's disease patients treated with donepezil: a longitudinal naturalistic cohort	Gustavsson A, Jonsson L, Parmler J
2012	Incremental Dementia-Related Expenditures in a Medicaid Population	Bharmal F., Dedhiya S., Bruce A.
2012	Exploring the relationship between AD severity and longitudinal costs	Rapp
2012	The costs of Alzheimer's disease and the value of effective therapies	Richard G Stefanacci
2012	The societal costs of dementia in Sweden 2012	Wimo
2013	Determinants of care costs of patients with dementia or cognitive impairment.	Handels RL, Wolfs CA, Aalten P, Verhey FR, Severens JL.
2013	2013 Alzheimer's disease facts and figures	Alzheimer's Association
2013	Are community-living and institutionalized dementia patients cared for differently? Evidence on service utilization and costs of care from German insurance claims data	Larissa Schwarzkopf, Petra Menn, Reiner Leidl
2013	Association between illness progression measures and total cost in Alzheimer's disease	Lacey LA, Niecko T, Leibman C
2013	Comparison of informal care time and costs in different age-related dementias: a review	Michel Lamure; Alain Grand; Laurent Molinier
2013	Cost of dementia care in India: Delusion or reality?	Girish N Rao,†Srikala Bharath

2013	Cost-effectiveness analyses for mirtazapine and sertraline in dementia: randomised controlled trial.	Romeo R, Knapp M, Hellier J
2013	Determinants of care costs of patients w dementia or cog inp	Handels
2013	Health disparities in cost of care in patients with Alzheimer's disease: an analysis across 4 state Medicaid populations	Daniel C Malone; Terri L Warholak; Edward P Armstrong
2013	Impact of Dementia on Payments for Long-term and Acute Care in an Elderly Cohort	Greg Arling, Wanzhu Tu, Christopher Callahan
2013	Monetary Costs of Dementia in the United States	Michael D. Hurd, Paco Martorell, Adeline Delavande
2013	Pre-Diagnosis Excess Acute Care Costs in Alzheimerís Patients among a US Medicaid Population	David S. Geldmacher, Noam Y. Kirson, Howard G. Birnbaum
2013	Road to the nursing home: costs and disease progression among medicare beneficiaries with ADRD	Lisa Mucha; Michael Treglia; Vincent Mor
2013	The GERAS Study: a prospective observational study of costs and resource use in community dwellers with Alzheimer's disease in three European countries--study design and baseline findings	Diego Novick; Bruno Vellas; Josep Maria Haro
2013	The burden of caring for dementia patients: caregiver reports from a cross-sectional hospital-based study in China	Joaqui¥n F Mould-Quevedo, Boxiong Tang, Eran Harary
2013	The clinical and economic burden of newly diagnosed Alzheimer's disease in a medicare advantage population	Ashish V Joshi; Warachal E Faison; Sonali N Shah
2013	The effects of dependence and function on costs of care for Alzheimer's disease and mild cognitive impairment in Ireland	D Gallagher; A Ni Mhaolain; B. Lawlor for the Enhancing Care in Alzheimer's Disease (ECAD) Study Team
2013	The impact of memantine in combination with acetylcholinesterase inhibitors on admission of patients with Alzheimer's disease to nursing homes: cost-effectiveness analysis in France	Anna Granghaud; Benoit Rive; SÈbastien Bineau
2013	The use and costs of formal care in newly diagnosed dementia: a three-year prospective follow-up study	Ingelin Testad; Anders Wimo; Dag Aarsland
2014	2014 Alzheimer's disease facts and figures	Alzheimer's Association
2014	Alzheimer's disease costs: what we know and what we should take into account	Giuseppe Lucio Gaeta; Giovanna Ricci; Francesco Amenta
2014	Analysis of burden in caregivers of people with Alzheimer's disease using self-report and supervision hours	Haro JM,†Kahle-Wrobleski K,†Bruno G
2014	Connolly 2014 dem estimating the economic and social cost of dem in Ireland	Connolly
2014	Cost and burden of informal caregiving of dependent older people in a rural Indian community	Ethel M Brinda, Anto P Rajkumar, Ulrika Enemark
2014	Cost of care attributable to Alzheimer's Disease for Medicare enrollees	Juarez D, Davis J
2014	Dementia UK: Update, Second Edition	Prince, M., Knapp, M., Guerchet, M.
2014	Early psychosocial intervention in Alzheimer's disease: cost utility evaluation alongside the Danish Alzheimer's Intervention Study (DAISY)	Dorte V Buss; Kieu T T Phung; Gunhild Waldemar; DAISY Study Investigators
2014	Economic valuation and determinants of informal care to people with Alzheimer's disease	Luz MarÌa PeÒa-Longobardo; Juan Oliva-Moreno
2014	Evaluation of full costs of care for patients with Alzheimer's disease in France: the predominant role of informal care	ChloÈ GervËs; Pauline Chauvin; Martine Marie Bellanger
2014	Health economic evaluation of treatments for Alzheimer's disease: impact of new diagnostic criteria	L Jonsson; A S Khachaturian; M Kramberger
2014	Implications of early treatment among Medicaid patients with Alzheimer's disease	David S. Geldmacher, Noam Y. Kirson, Howard G. Birnbaum
2014	Increased Healthcare Service Utilizations for Patients with Dementia: A Population-Based Study	Shiu-Dong Chung , Shih-Ping Liu , Jau-Jiuan Sheu , Ching-Chun Lin, Herng-Ching Lin
2014	Cost of dementia in the pre enlargement countries of the EU	LuengoFernandez

2014	The costs of dementia from the societal perspective: is care provided in the community really cheaper than nursing home care?	Kˆnig HH, Leicht H, Brettschneider C,
2014	Cost of care for people with dem in 8 EU countries	Wubker
2014	The cost of applying the Dependency Law to Alzheimer disease	Arantza Aiarza; Luis Carlos Abecia; Javier Mar
2015	Predictors of societal costs in dementia patients and their informal caregivers.	Joling
2015	2015 Alzheimer's disease facts and figures	Alzheimerís Association
2015	An Analysis of the Public Financial Support Eligibility Rule for French Dependent Elders with Alzheimer's Disease.	Rapp T, Lacey L, Ousset PJ
2015	Caravau 2015 AN direct costs of dementia in nursing homes	Caravau
2015	Dependence in Alzheimer's disease and service use costs, quality of life, and caregiver burden: the DADE study	Derek King; Timothy Niecko; Loretto Lacey;
2015	Determinants of societal costs in Alzheimer's disease: GERAS study baseline results	Giuseppe Bruno; Bruno Vellas; Josep Maria Haro
2015	Diagnostic Pathways to Alzheimer Disease: Costs Incurred in a Medicare Population	Joanna M Kubisiak; Khaled Sarsour; Craig A Hunter
2015	Epidemiological and economic burden of Alzheimer's disease: a systematic literature review of data across Europe and the United States of America	Anneloes van Walsem; CÈline Faure; William C Maier
2015	Gender Differences: A Lifetime Analysis of the Economic Burden of Alzheimer's Disease	Zhou Yang; Allan Levey
2015	Hospital admissions, outpatient visits and healthcare costs of community-dwellers with Alzheimer's disease	Marjaana Koponen; Hilkka Soininen; Sirpa Hartikainen
2015	Longitudinal costs of caring for people with Alzheimer's disease	Paddy Gillespie, Eamon OíShea, John Cullinan
2015	Medical management, costs and consequences of Alzheimerís disease in Germany: an analysis of health claims data	Bernd Br¸ggenj¸rgen, Frank Andersohn, Nadja Ezzat
2015	Medical management, costs, and consequences of Alzheimer's disease in Germany: an analysis of health claims data.	..
2015	Memantine for treatment of moderate or severe Alzheimer's disease patients in urban China: clinical and economic outcomes from a health economic model	Emilie Clay; Mondher Toumi; Dominique Milea
2015	Methodological challenges in assessing the impact of comorbidities on costs in Alzheimer's disease clinical trials	Jonathan Kurlander; Catherine Reed; Mark Belger
2015	Monetary costs of agitation in older adults with Alzheimer's disease in the UK: prospective cohort study	Cornelius Katona; Claudia Cooper; Gill Livingston
2015	PMH18 - Observational Study of Resource use and Cost of Alzheimerís Disease in Europe (Geras) - 18-Month Results from the French Cohort	Chartier F, Baraille L, Belger M.
2015	REDIC 2015 Rapport Kortversjon Norway	REDIC
2015	Relationship between global severity of patients with Alzheimer's disease and costs of care in Spain; results from the co-dependence study in Spain	J Darb‡; L Kaskens; L Lacey
2015	Relationship between patient dependence and direct medical-, social-, indirect-, and informal-care costs in Spain	Darb‡ J, Kaskens L
2015	The economic impact of moderate stage Alzheimer's disease in Italy: evidence from the UP-TECH randomized trial	Antonio Cherubini; Andrea Corsonello; Fabrizia Lattanzio; UP-TECH research group
2015	The hidden cost of dementia in Wales	Bo Hu, Raphael Wittenberg, Martin Knapp
2015	The main cost drivers in dementia: a systematic review	Sandra Schaller, Josephine Mauskopf, Christine Kriza
2015	World Alzheimer Report 2015: The Global Impact of Dementia	Martin Prince, Anders Wimo, Meelenn Guerchet

2015	Outpatient Care of People with Dementia within Residential Communities in Germany--Care Potential and Cost.	W¸bbeler M1, Aflmann G1, Blaut S2, Lueke S1, Hoffmann W1, Flefla S2.
2015	The Epidemiology of Dementia and Alzheimer Disease in Portugal: Estimations of Prevalence and Treatment-Costs	Sandra Freitas; VÌtor Rodrigues; ≈lvaro Carvalho
2016	Cost Related to Dementia in the Young and the Impact of Etiological Subtype on Cost.	Kandiah N, Wang V, Lin X, Nyu MM, Lim L, Ng A, Hameed S, Wee HL.
2016	Cost of Dementia and Its Correlation With Dependence.	√Ökerborg √ñ, Lang A, Wimo A, Sk√∂ldunger A, Fratiglioni L, Gaudig M, Rosenlund M.
2016	Direct and indirect costs and resource use in dementia care: A cross-sectional study in patients living at home.	Farr√© M, Haro JM, Kostov B, Alvira C, Risco E, Miguel S, Cabrera E, Zabalegui A.
2016	2016 Alzheimer's disease facts and figures	Alzheimer's Association
2016	Abdin 2016 JAD the societal cost of dementia in Singapore	Abdin
2016	Alzheimer's and Parkinson's Diseases: Expected Economic Impact on Europe-A Call for a Uniform European Strategy	Blanka Klimova; Michal Novotny; Kamil Kuca
2016	Alzheimer's and other dementias in Canada, 2011 to 2031: a microsimulation Population Health Modeling (POHEM) study of projected prevalence, health burden, health services, and caregiving use	Kim Reimer; Larry W Chambers; Julie Bernier
2016	Assessing the economic burden of Alzheimer's disease patients first diagnosed by specialists	J Scott Andrews; Daniel Ball; Kristin Kahle-Wrobleski
2016	Consequences of Anosognosia on the Cost of Caregivers' Care in Alzheimer's Disease	Laia CalvÛ-Perxas; Jordi GascÛn-Bayarri; Josep-LluÌs Conde-Sala
2016	Cost of Dementia and Its Correlation With Dependence	≈kerborg ÷1, Lang A1, Wimo A2, Skˆldunger A2, Fratiglioni L3, Gaudig M4, Rosenlund M1,5.
2016	Cost-effectiveness of donepezil and memantine in moderate to severe Alzheimer's disease (the DOMINO-AD trial)	Martin Knapp; Derek King; RenÈe Romeo
2016	Frailty: a costly phenomenon in caring for elders with cognitive impairment	Butler A, Gallagher D, Gillespie P
2016	Health, social and economic consequences of dementias: a comparative national cohort study	S Frahm-Falkenberg; R Ibsen; J Kjellberg; P Jennum
2016	Hospital care and drug costs from five years before until two years after the diagnosis of Alzheimer's disease in a Finnish nationwide cohort	Antti Tanskanen; Jari Tiihonen; Sirpa Hartikainen
2016	Identifying factors of activities of daily living important for cost and caregiver outcomes in Alzheimer's disease.	Reed C, Belger M, Vellas B
2016	Economic impact of dementia by disease severity exploring the relationship between stage of dementia and cost of care in Taiwan	Ku
2016	Prevalence and Monetary Costs of Dementia in Canada	larry Chambers, Christina Bancej, Ian McDowell
2016	Resource utilisation, costs and clinical outcomes in non-institutionalised patients with Alzheimer's disease: 18-month UK results from the GERAS observational study	Jeremie Lebrec; Mark Belger; Roy W Jones
2016	Socio-economic Aspects of Alzheimer's Disease	Hana Mohelsk·; Josef Dolejö; Kamil Kuca
2016	The Impact of Alzheimer's Disease in an Aging Rural Population	Vivian Minkemeyer; Courtney Wellman; Lynne Goebel
2016	The Impact of Alzheimer's Disease on the Chinese Economy	Henning Tarp Jensen; H Michael Arrighi; Richard D Smith
2016	The economic burden of the care and treatment for people with AD. The outlook for the Czech Republic	NA
2016	The worldwide costs of dementia 2015 and comparisons with 2010	Anders Wimo, MaCelenn Guerchet, Gemma-Claire Ali
2016	economic analysis of formal care informal care and productivity losses in primary care patients in Germany	Michalowsky2016
2017	Monetary cost of family caregiving for people with dementia in Singapore.	Woo LL, Thompson CL, Magadi H.

2017	Economic impact of cognitive impairment and dementia.	Cantarero Prieto D.
2017	Actual expense associated with patients with Alzheimer's disease in Colombia	Sergio I Prada; Yuri Takeuchi; Angela M Merch·n-Galvis; Yoseth Ariza-Ara˙jo
2017	Costs and quality of life in community-dwelling patients with Alzheimer's disease in Spain: results from the GERAS II observational study	Javier Olazar·n; Luis Ag¸era-Ortiz; Josep MarÌa ArgimÛn; Catherine Reed; Antonio Ciudad; Paula Andrade; Tatiana Dilla
2017	Deloitte 2017 Economic Impacts of Dementia 2016 in New Zealand	Deloitte
2017	Dementia in sub-Saharan Africa Challenges and opportunities	MaÎlenn Guerchet, Rosie Mayston, Pter Lloyd-Sherlock
2017	Direct and indirect cost of managing alzheimer's disease and related dementias in the United States	Arijita Deb; James Douglas Thornton; Usha Sambamoorthi; Kim Innes
2017	Economic cost of demenita in Australia	Laurie Brown, Erick Hansnata and Hai Anh La
2017	Executive function, episodic memory, and Medicare expenditures	Bender AC, Austin AM, Grodstein F
2017	Cost of dementia in the Czech Republic	Holmerova
2017	Literature Review of The Cost of Dementia on China	Gurnani M
2017	Monetary cost of family caregiving for people with dementia inSingapore	Lai Leng Woo, Claire L.Thompson, HarishMagadic
2017	Societal and Family Lifetime Cost of Dementia: Implications for Policy	Eric Jutkowitz. Robert L. Kane, Joseph E. Gaugler
2017	The cost of care homes for people with dementia in England: a modelling approach	Renee Romeo , Martin Knapp, Suzanne Salverda
2017	The cost of dementia in an unequal country: The case of Chile	Daniel A. Hojman , Fabian Duarte, Jaime Ruiz-Tagle
2017	Trends in prevalence, incidence, health system use and cost by persons with dementia in Ontario from 2004 and 2013: a population-based study	Susan Bronskill, Jun Guan, Marian Vermeulen
2017	What Drives Country Differences in Cost of Alzheimer's Disease? An Explanation from Resource Use in the GERAS Study	Catherine Reed; Michael Happich; Josep Maria Argimon
2017	WHO the economic burden of dementia in China 1990 to 2013	Xu
2018	Healthcare utilization and costs in primary care patients with dementia: baseline results of the DelpHi-trial.	Michalowsky B, Flessa S, Eichler T, Hertel J, Dreier A, Zwingmann I, Wucherer D, Rau H, Thyrian JR, Hoffmann W.
2018	2018 Alzheimer's Disease Facts and Figures	Joseph Gaugler, Bryan James, Tricia Johnson
2018	A Comprehensive Measure of the Costs of Caring for a Parent: Differences According to Functional Status	Norma B. Coe, Meghan M. Skira, Eric B. Larson
2018	An Exploratory Cost-Effectiveness Analysis of the Connected Health Intervention to Improve Care for People with Dementia: A Simulation Analysis	William N. Dowd, Alexander J. Cowell, Daniel Regan
2018	Attributable Cost of Dementia: Demonstrating Pitfalls of Ignoring Multiple Health Care System Utilization	Lianlian Lei, Susan G. Cooley, Ciaran S. Phibbs
2018	Cost and resource use associated with AD in italy	Bruno
2018	Comprehensive cost of illness of dementia in Japan: a time trend analysis based on Japanese official statistics	Shimpei Hanaoka Kunichika Matsumoto Takefumi Kitazawa
2018	Dementia -- the true cost: Fixing the care crisis	Rachel Hutchings, Dominic Carter, Katie Bennett
2018	Does Structured Exercise Improve Cognitive Impairment in Peoplewith Mild to Moderate Dementia? A Cost-Effectiveness Analysisfrom a Confirmatory Randomised Controlled Trial: The Dementiaand Physical Activity (DAPA) Trial	Iftekhar Khan, Stavros Petrou, Kamran Khan

2018	Evaluation of an integrated primary care-led dementia shared care program in Singapore: An effectiveness and cost-effectiveness study	Nakul Saxena, Pradeep Paul George, Kelvin WS Teo
2018	Ferretti 2018 PO An assessement of direct and indirect costs of dementia in Brazil	Ferretti
2018	Improving the quality of life of care home residents with dementia :Cost-effectiveness of an optimized intervention for residents with clinically significant agitation in dementia	Renee Romeoa, Darshan Zalaa, Martin Knapp
2018	Resource Use and Cost of Alzheimer's Disease in France: 18-Month Results from the GERAS Observational Study.	Rapp T, Andrieu S, Chartier F
2018	The cost of Alzheimer's disease in China and re-estimation of costs worldwide	Jia J, Wei C, Chen S
2018	The estimated cost of dementia in Japan, the most aged society in the world	Mitsuhiro SadoI, Akira Ninomiya, Ryo Shikimoto
2018	The personal cost of dementia care in Japan: A comparative analysis of residence types	Takayo Nakabe Noriko Sasaki Hironori Uematsu

Table S3: Number of data points by geography and category

	Dementia prevalence	Socio-demographic index	Total health expenditure	Diagnosis and treatment rates	Institutionalization rates	Dementia costs
China	20	20	20	2	4	19
North Korea	20	20	20			
Taiwan (Province of China)	20	20	20		1	31
Cambodia	20	20	20			
Indonesia	20	20	20			
Laos	20	20	20			
Malaysia	20	20	20			
Maldives	20	20	20			
Myanmar	20	20	20			
Philippines	20	20	20			
Sri Lanka	20	20	20			
Thailand	20	20	20	1		
Timor-Leste	20	20	20			
Vietnam	20	20	20			
Fiji	20	20	20			
Kiribati	20	20	20			
Marshall Islands	20	20	20			
Federated States of Micronesia	20	20	20			
Papua New Guinea	20	20	20			
Samoa	20	20	20			
Solomon Islands	20	20	20			
Tonga	20	20	20			
Vanuatu	20	20	20			
Armenia	20	20	20			
Azerbaijan	20	20	20			
Georgia	20	20	20			
Kazakhstan	20	20	20			
Kyrgyzstan	20	20	20			
Mongolia	20	20	20			
Tajikistan	20	20	20			
Turkmenistan	20	20	20			
Uzbekistan	20	20	20			
Albania	20	20	20			
Bosnia and Herzegovina	20	20	20			
Bulgaria	20	20	20			
Croatia	20	20	20			

Czech Republic	20	20	20			7
Hungary	20	20	20			2
Macedonia	20	20	20			
Montenegro	20	20	20			
Poland	20	20	20			
Romania	20	20	20			
Serbia	20	20	20			
Slovakia	20	20	20			
Slovenia	20	20	20			
Belarus	20	20	20			
Estonia	20	20	20			
Latvia	20	20	20			
Lithuania	20	20	20			
Moldova	20	20	20			
Russian Federation	20	20	20		1	
Ukraine	20	20	20			
Brunei	20	20	20			
Japan	20	20	20		3	5
South Korea	20	20	20		1	4
Singapore	20	20	20		1	2
Australia	20	20	20		1	51
New Zealand	20	20	20			1
Andorra	20	20	20			
Austria	20	20	20			
Belgium	20	20	20			6
Cyprus	20	20	20			
Denmark	20	20	20		2	9
Finland	20	20	20	1	2	7
France	20	20	20		2	14
Germany	20	20	20	2	2	67
Greece	20	20	20			
Iceland	20	20	20			
Ireland	20	20	20	1		5
Israel	20	20	20		1	14
Italy	20	20	20	1	1	2
Luxembourg	20	20	20			
Malta	20	20	20			
Netherlands	20	20	20	1		7
Norway	20	20	20	2	1	4
Portugal	20	20	20			1
Spain	20	20	20	1	5	23
Sweden	20	20	20	2	4	31
Switzerland	20	20	20	2		3
United Kingdom	20	20	20	8	8	14
Argentina	20	20	20		2	5
Chile	20	20	20		1	5
Uruguay	20	20	20			
Canada	20	20	20	2	1	1
USA	20	20	20	16	13	135
Antigua and Barbuda	20	20	20			
The Bahamas	20	20	20			
Barbados	20	20	20			
Belize	20	20	20			
Cuba	20	20	20			
Dominica	20	20	20			
Dominican Republic	20	20	20		1	
Grenada	20	20	20			
Guyana	20	20	20			
Haiti	20	20	20			
Jamaica	20	20	20			
Saint Lucia	20	20	20			
Saint Vincent and the Grenadines	20	20	20			
Suriname	20	20	20			
Trinidad and Tobago	20	20	20			
Bolivia	20	20	20			
Ecuador	20	20	20			
Peru	20	20	20		1	

Colombia	20	20	20		1	
Costa Rica	20	20	20			
El Salvador	20	20	20			
Guatemala	20	20	20			
Honduras	20	20	20			
Mexico	20	20	20		1	4
Nicaragua	20	20	20			
Panama	20	20	20			
Venezuela	20	20	20		1	
Brazil	20	20	20	1		2
Paraguay	20	20	20			
Algeria	20	20	20			
Bahrain	20	20	20			
Egypt	20	20	20			
Iran	20	20	20			
Iraq	20	20	20			
Jordan	20	20	20			
Kuwait	20	20	20			
Lebanon	20	20	20			
Libya	20	20	20			
Morocco	20	20	20			
Palestine	20	20	20			
Oman	20	20	20			
Qatar	20	20	20			
Saudi Arabia	20	20	20			
Syria	20	20	20			
Tunisia	20	20	20			
Turkey	20	20	20		1	1
United Arab Emirates	20	20	20			
Yemen	20	20	20			
Afghanistan	20	20	20			
Bangladesh	20	20	20			
Bhutan	20	20	20			
India	20	20	20	1	1	
Nepal	20	20	20			
Pakistan	20	20	20			
Angola	20	20	20			
Central African Republic	20	20	20			
Congo (Brazzaville)	20	20	20			
DR Congo	20	20	20			
Equatorial Guinea	20	20	20			
Gabon	20	20	20			
Burundi	20	20	20			
Comoros	20	20	20			
Djibouti	20	20	20			
Eritrea	20	20	20			
Ethiopia	20	20	20			
Kenya	20	20	20			
Madagascar	20	20	20			
Malawi	20	20	20			
Mauritius	20	20	20			
Mozambique	20	20	20			
Rwanda	20	20	20			
Seychelles	20	20	20			
Somalia	20	20	20			
Tanzania	20	20	20			
Uganda	20	20	20			
Zambia	20	20	20			
Botswana	20	20	20			
Lesotho	20	20	20			
Namibia	20	20	20			
South Africa	20	20	20			
Swaziland	20	20	20			
Zimbabwe	20	20	20			
Benin	20	20	20			
Burkina Faso	20	20	20			
Cameroon	20	20	20			
Cape Verde	20	20	20			

Chad	20	20	20			
Cote d'Ivoire	20	20	20			
The Gambia	20	20	20			
Ghana	20	20	20			
Guinea	20	20	20			
Guinea-Bissau	20	20	20			
Liberia	20	20	20			
Mali	20	20	20			
Mauritania	20	20	20			
Niger	20	20	20			
Nigeria	20	20	20			
Sao Tome and Principe	20	20	20			
Senegal	20	20	20			
Sierra Leone	20	20	20			
Togo	20	20	20			
American Samoa	20	20	20			
Bermuda	20	20	20			
Greenland	20	20	20			
Guam	20	20	20			
Northern Mariana Islands	20	20	20			
Puerto Rico	20	20	20			
Virgin Islands	20	20	20			
South Sudan	20	20	20			
Sudan	20	20	20			

Analytical framework and methods

Figure S1: Dementia spending analytical framework

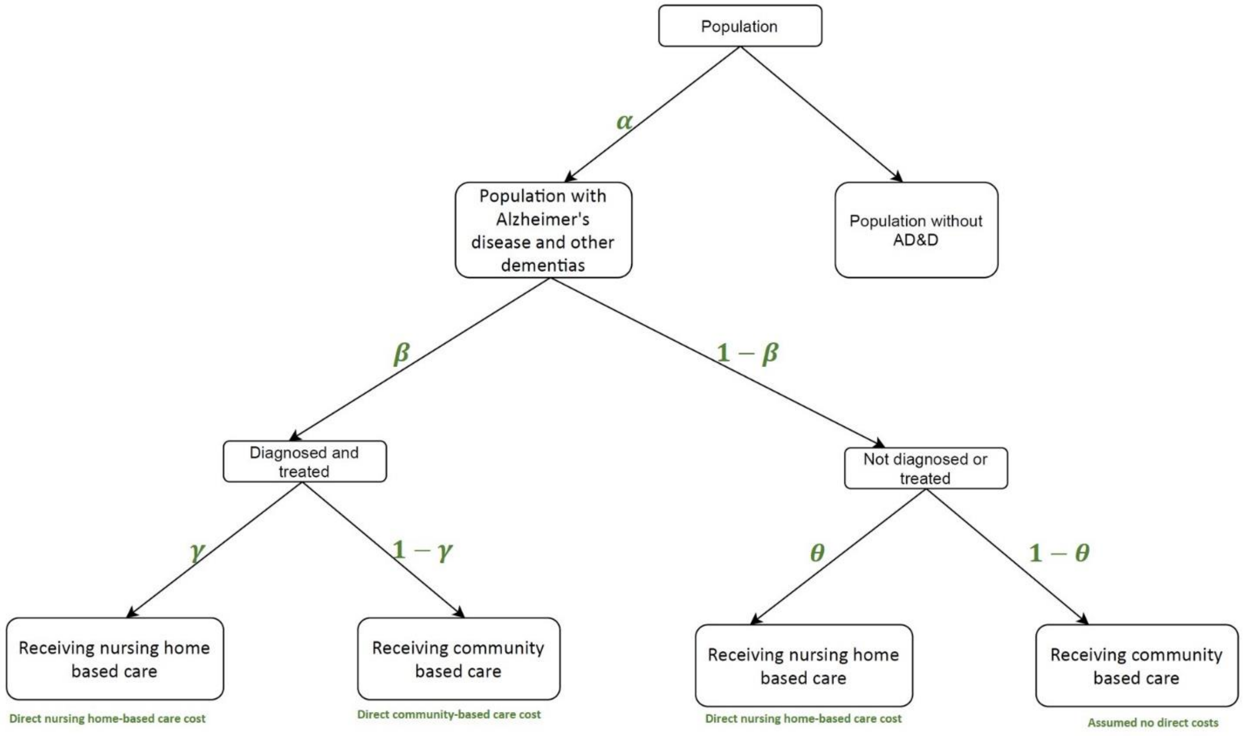

Variable	Description
α	Dementia prevalence rate
β	Diagnosis and treatment rate for those who have dementia
γ	Nursing home-based-care rate for those who have been diagnosed with dementia
1-γ	Community-based care rate for those who have been diagnosed with dementia
θ	Nursing-home-based care rate for those with dementia but without a diagnosis
Direct nursing home-based-care cost	Estimated annual healthcare spending for those diagnosed with dementia receiving nursing-home-based care
Direct community-based care cost	Estimated annual healthcare spending for those diagnosed with dementia receiving community-based care

It was assumed that there is no direct spending on dementia for those without dementia, and that there was no spending on dementia for those with dementia who are undiagnosed and living in the community.

Equations:

Nursing-home-based care cost

$$Y_{ip} = Pop * \left[\alpha\beta\gamma Y_{diagnosed\,\&\,NHBC} + \alpha(1-\beta)\theta Y_{undiagnosed\,\&\,NHBC}\right]$$

Community-based care cost

$$Y_{op} = Pop * \left[\alpha\beta(1-\gamma) Y_{Treated\,\&\,CBC}\right]$$

Total spending

$$Total\ spending = Y_{ip} + Y_{op}$$

Nursing-home-based care rates for patients with dementia who lack a diagnosis:

During our dementia diagnosis and treatment literature review we extracted diagnosis and treatment rates for patients with dementia in community-based care, nursing-home-based care, and not reported settings—beta-community, beta-institution, and beta, respectively. We used this data to estimate nursing-home-based care rates for those with dementia, but without a diagnosis using the equation below.

$$\theta = \gamma * \beta * \frac{1 - \beta_{NBHC}}{(1-\beta) * \beta_{NHBC}}$$

Future scenarios assumptions:

In our future dementia-spending model, we capped diagnosis and treatment rates at 90% and kept the rate of patients diagnosed with dementia who receive nursing home-based-care under 80%.

Outliers

We identified outliers by applying Cook's distance analysis with a threshold of 4/sample size[1]. We also assumed that data points within the same country should follow the community based care cost < nursing home based care cost inequality. Points that did not follow that inequality were also labeled as outliers and are presented below.

Country	Year	Care setting	Value	Currency
South Korea	2004	CBC	8826	2019 USD
Japan	2014	NHBC	5916	2019 USD
Finland	2009	NHBC	10 381	2019 USD
United Kingdom	2006	CBC	54 498	2019 USD

[1] "Cook's Distance — Yellowbrick v1.3.Post1 Documentation."

Taiwan	2010	NHBC	4157	2019 USD
China	2000	NHBC	617	2019 USD
Germany	2008	CBC	54 968	2019 USD
Spain	2001	CBC	47 669	2019 USD
The Netherlands	2012	NHBC	199 128	2019 USD

Estimating spending attributable to dementia

Dementia is a disease commonly diagnosed among the elderly. People with dementia often suffer from co-morbidities associated with aging. For this reason, it is important to differentiate between the costs to treat and care for someone with dementia and costs to treat other diseases common among this demographic. We conducted a meta-analysis of the dementia attributable fraction by care setting—community-based care, nursing-home-based care, not reported, and mixed using the metaphor library in R[2]. Using the rma function we ran a meta-analysis with mixed effects by care setting. With the simulate.rma function of the same R-library we ran simulations to generate uncertainty intervals. These functions fit meta-analytic fixed- and random/mixed-effects via linear (mixed-effects) models. Once we had a set of dementia attributable fraction estimates by care setting, we multiplied total dementia cost by the corresponding attributable fraction. Due to lack of data, we were not able to estimate attributable fractions that are time and geography specific.

Currency conversion process

Our currency conversion process follows the process described in figure S2. For data reported in nominal LCU we used country specific deflator series to estimate real LCU. We then used country specific exchange rates to calculate real USD. When data was reported in nominal PPP we used country specific PPP conversion rates to estimate nominal LCU. All deflators and exchange rates were extracted from the World Bank[3], International Monetary Fund[4], Penn World Tables[5], the United Nations National Accounts[6], and the World Health Organization[7], and were imputed to provide a complete series for each of the variables between 1950 and 2020. We then used several models including ordinary least-squares regression and mixed effects models, to complete each source series from 1950 to 2020.

[2] "Metafor.Pdf."
[3] "Search | Data Catalog."
[4] "World Economic Outlook Databases."
[5] "PWT 10.0."
[6] "Basic Data Selection - AmaWebClient."
[7] "Global Health Expenditure Database."

Figure S2: Currency conversion process

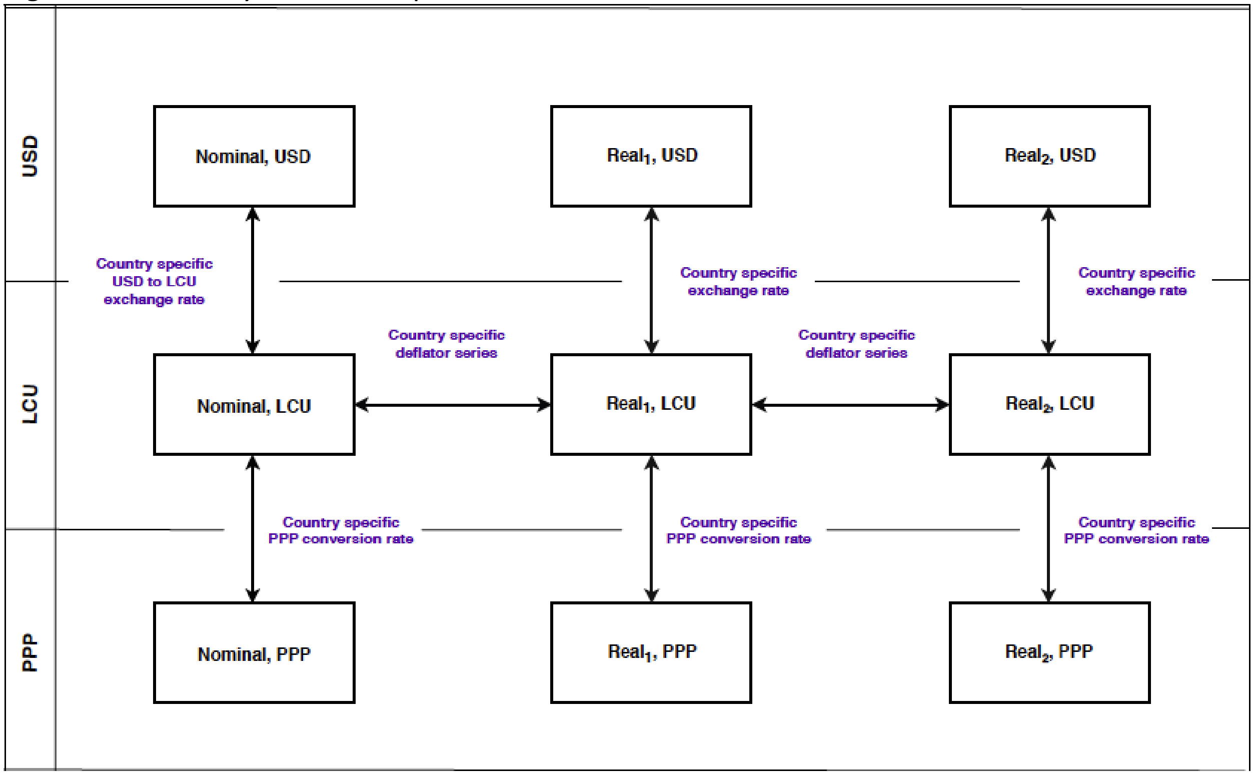

About spatiotemporal Gaussian process regression

The approach is a stochastic modelling technique that is designed to detect signals amidst noisy data. It also serves as a powerful tool for interpolating non-linear trends.[8] [9] Unlike classical linear models that assume that the trend underlying data follows a definitive functional form, GPR assumes that the specific trend of interest follows a Gaussian process, which is defined by a mean function and a covariance function.[10]
There are three main stages in ST-GPR modeling:

- Stage 1: Linear model. Conducts a linear regression to capture general trends in the data. Incorporates covariate information and produces a complete time-series.
- Stage 2: Spatiotemporal smoothing. Calculates residuals across time and space between data and linear predictions. Smooths residuals across time and space. Adds them to the linear regression in previous stage.
- Stage 3: Gaussian Process Regression (GPR). Incorporates uncertainty and makes predictions smoother. Generates better predictions for location that have data.

[8] Vasudevan S, Ramos F, Nettleton E, Durrant-Whyte H, Blair A. Gaussian Process modeling of large scale terrain. In: 2009 IEEE International Conference on Robotics and Automation. 2009: 1047–53.
[9] Rasmussen CE, Williams CKI. Gaussian Processes for Machine Learning. Cambridge, Mass: The MIT Press, 2005.
[10] Murray et al., "Global Burden of 87 Risk Factors in 204 Countries and Territories, 1990–2019."

Model fit summary

Community-based care costs

The table below reports all the data we had for direct, total spending for dementia patients who receive community based care. It also reports the estimates from our model as well as the ratio between estimated spending over observed spending.

Table S4: Estimates and observed data for direct, total spending in community based care.

	Year	Estimate	Observed data	Ratio (estimate/observed data)
Canada	2000	3426	3089	1.1
United States	2002	20335	20582	1.0
United States	2004	21238	10929	1.9
United States	2004	21238	26191	0.8
United States	2009	23453	30445	0.8
United States	2014	23356	7921	2.9
United States	2015	24097	30602	0.8
United States	2016	24634	30753	0.8
Argentina	2001	4105	2292	1.8
Argentina	2001	4105	6722	0.6
Chile	2011	2539	2288	1.1
Chile	2011	2539	2068	1.2
Chile	2011	2539	2294	1.1
Chile	2011	2539	2517	1.0
Chile	2011	2539	2850	0.9
France	1996	21207	14588	1.5
France	1996	21207	29522	0.7
France	2004	21432	10982	2.0
France	2004	21432	40330	0.5
France	2005	20991	8568	2.5
Germany	2008	10917	13617	0.8
Germany	2008	10917	13348	0.8
Germany	2014	9614	2152	4.5
Germany	2014	9614	18313	0.5
Ireland	2009	4188	3108	1.3
Ireland	2014	4410	4544	1.0
Israel	1999	13179	11957	1.1
Israel	1999	13179	20857	0.6
Israel	1999	13179	8175	1.6
Italy	2009	18091	19218	0.9

Netherlands	2012	24758	26256	0.9
Spain	2001	9887	9628	1.0
Sweden	2007	16199	16445	1.0
Switzerland	2007	20133	9334	2.2
Switzerland	2007	20133	34254	0.6
United Kingdom	2010	10326	11723	0.9
United Kingdom	2010	10326	8539	1.2
Turkey	2003	3171	3013	1.1
China	2000	3123	1603	1.9
China	2000	3123	6081	0.5
China	2006	3676	1829	2.0
China	2006	3676	5794	0.6
Taiwan	2002	6999	6768	1.0

Notes: All spending measured in 2019 US dollars.

Nursing home-based care

The table below reports all the data we had for direct total spending for dementia patients who receive nursing home based care. It also reports the estimates from our model as well as the ratio between estimated spending over observed spending.

Table S5: Estimates and observed data for direct, total spending in community based care.

	Year	Estimate	Observed data	Ratio (estimate/observed data)
Czech Republic	2015	18164	17708	1.0
United States	2009	80236	81487	1.0
United States	2015	83813	81908	1.0
United States	2016	84939	82310	1.0
Argentina	2001	7560	4231	1.8
Argentina	2001	7560	12265	0.6
Israel	1999	18482	17278	1.1
Israel	1999	18482	19992	0.9
Israel	1999	18482	17024	1.1
Portugal	2012	21140	20596	1.0
Sweden	2007	59313	59916	1.0
Switzerland	2007	43782	42671	1.0
United Kingdom	2015	58271	57758	1.0
China	2000	6006	5912	1.0
China	2009	8645	8126	1.1
Taiwan	1996	20038	19668	1.0

Notes: All spending measured in 2019 US dollars.

Results

Figure S4: Dementia spending from 2000 to 2019 and future dementia spending scenarios from 2020 to 2050—baseline and alternative scenarios*

Time series of the dementia spending over total health spending fraction.

The purple line represents the observed dementia spending over total health spending ratio from 2000 to 2019 as well as forecasted ratio for the reference scenario from 2020 to 2050.

The purple shadow illustrates the 95% uncertainty interval for the observed dementia spending over total health spending ratio from 2020 to 2019 and the forecasted reference scenario from 2020 to 2050.

The green line represents the ratio between forecasted dementia spending with accelerated nursing home-based-care rates and total health spending from 2020 to 2050.

The blue line represents the ratio between forecasted dementia spending with accelerated diagnosis and treatment rates and total health spending from 2020 to 2050.

The red line represents the ratio between forecasted dementia spending with accelerated unit costs and total health spending from 2020 to 2050.

* Dementia spending over total health spending decreased in 2020 due to the increase in global health spending in response to the Covid-19 pandemic. The fraction sharply increases after 2020 as we expect a reduction in THE in the coming years due to slower than usual GDP growth and increased national debt. The expectation is that resources needed for dementia will not be affected by increased spending on Covid-19.

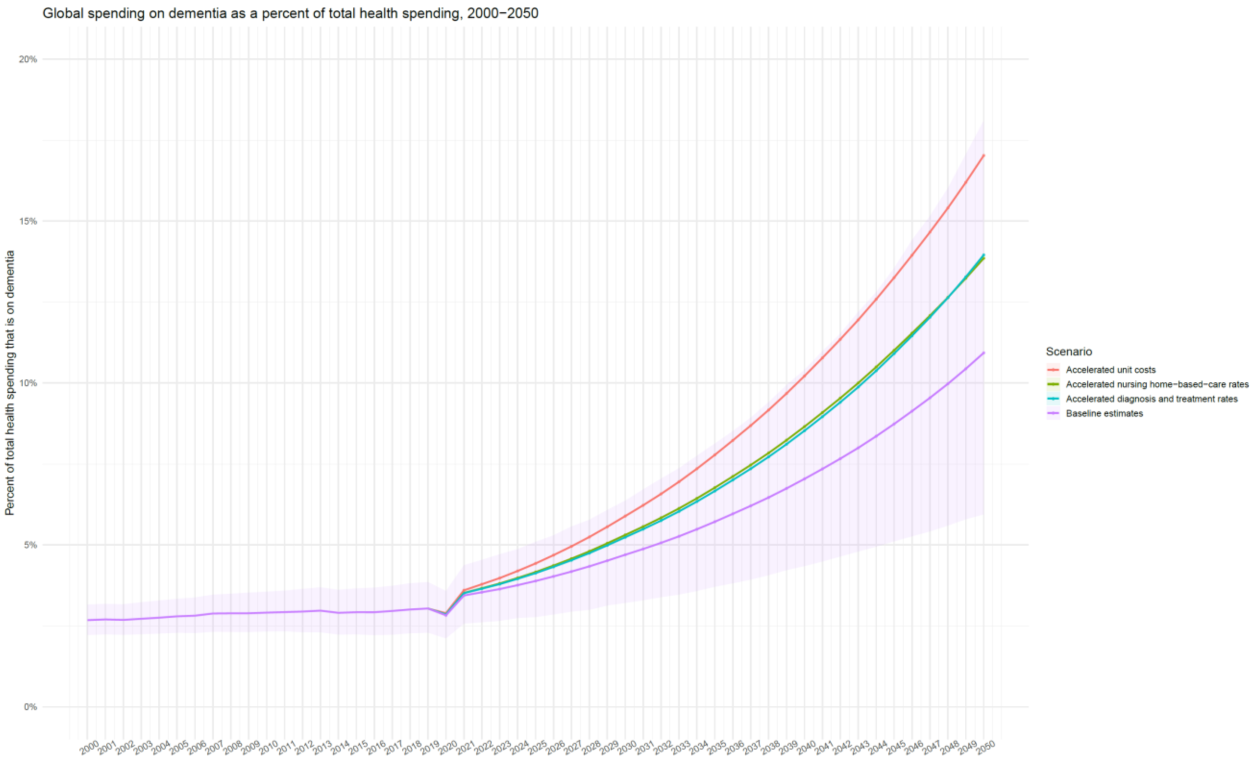

Figure S5: Map of dementia spending per person (2019 USD)

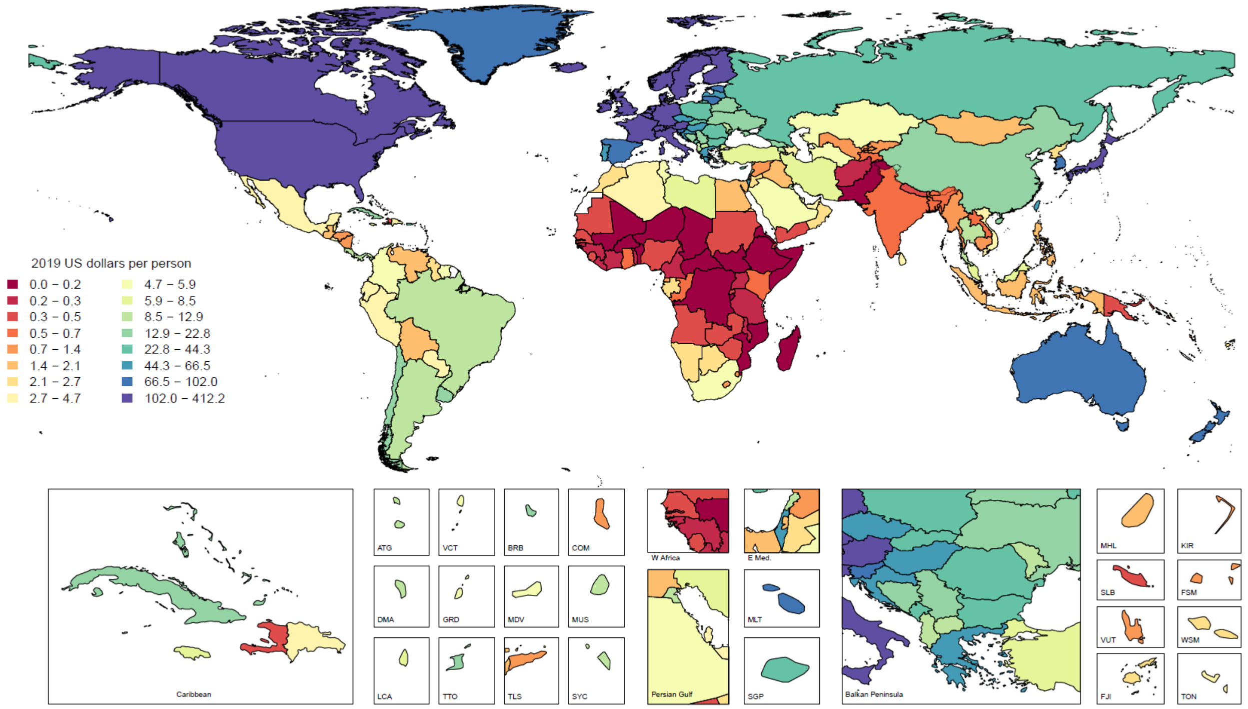

Figure S6: Map of dementia spending per prevalent case (2019 USD)

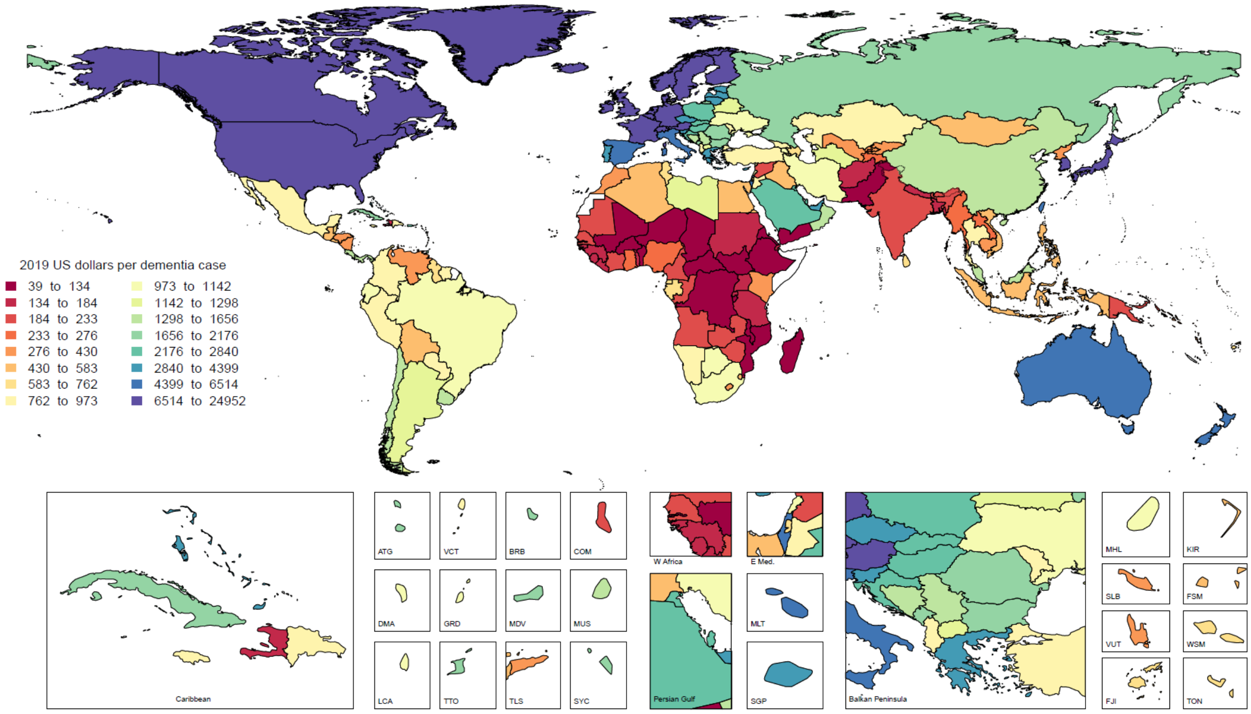

Figure S7: Map of dementia spending and total health spending per capita ratio

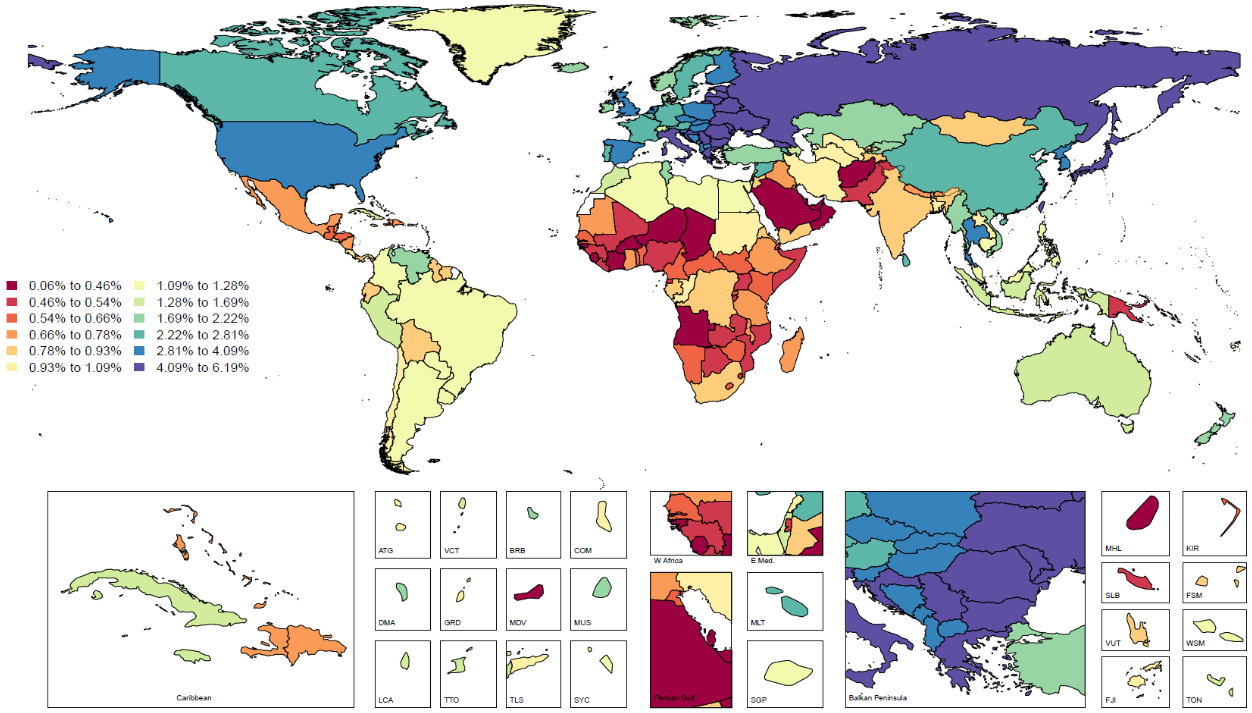

Figure S8: Global spending on dementia per person relative to economic development in 2019 (2019 PPP)

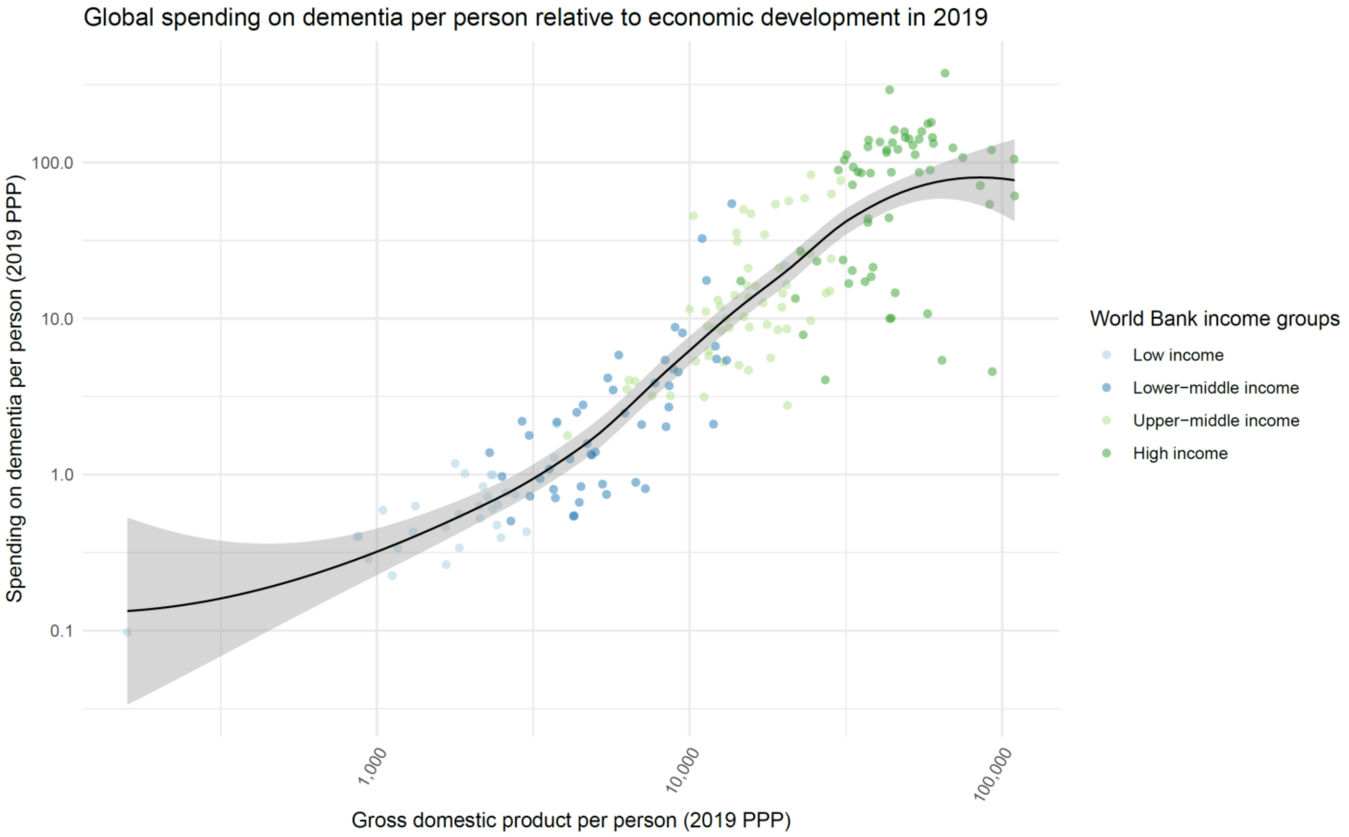

Figure S9: Global spending on dementia per person relative to total health expenditure in 2019 (2019 USD)

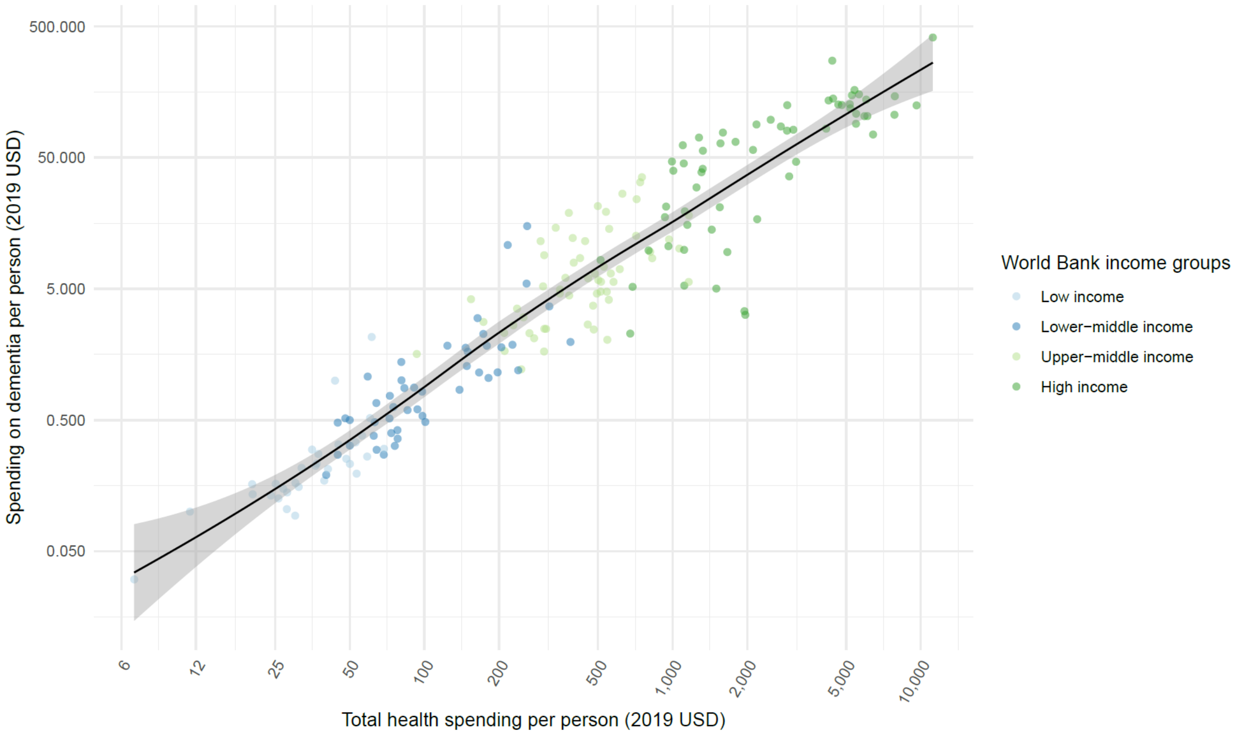

Figure S10: Global spending on dementia per person relative to total health expenditure in 2019 (2019 PPP)

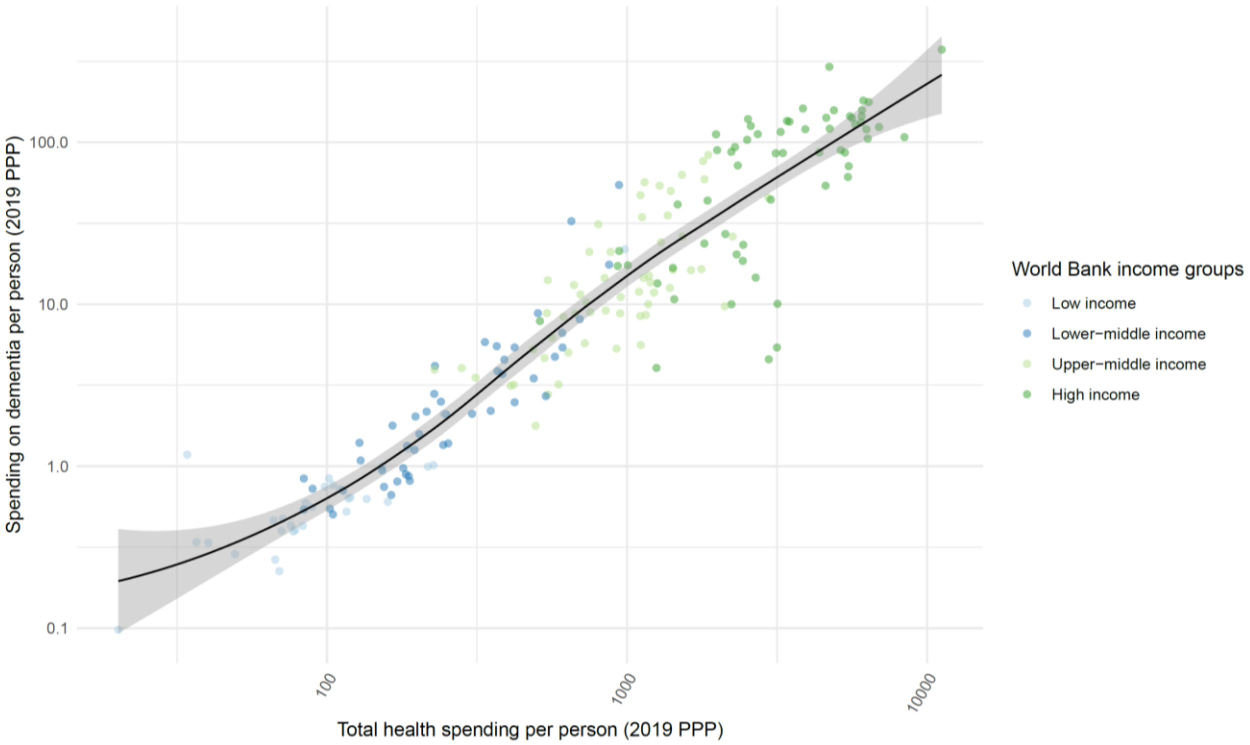

Table S6: Attributable spending by care setting per person by World Bank income group and GBD super region in 2019 (2019 USD)

	Community based care spending per person (2019)	Nursing Home based care spending per person (2019)
**World Bank Income Groups**		
High income	$29.0 ($21.7 - $37.9)	$160.2 ($117.5 - $206.4)
Upper middle income	$4.3 ($2.2 - $7.2)	$7.2 ($3.3 - $12.1)
Lower middle income	$0.4 ($0.2 - $0.5)	$0.7 ($0.3 - $1.2)
Low income	$0.1 ($0.1 - $0.2)	$0.2 ($0.1 - $0.2)
**Global Burden of Disease Super Regions**		
Central Europe, Eastern Europe, and Central Asia	$3.5 ($2.0 - $5.3)	$19.9 ($9.3 - $31.8)
High-income	$31.6 ($23.6 - $41.4)	$174.7 ($128.8 - $225.1)
Latin America and Caribbean	$2.2 ($1.2 - $3.6)	$4.2 ($2.0 - $6.9)
North Africa and Middle East	$1.4 ($0.8 - $2.2)	$2.1 ($1.0 - $3.3)
South Asia	$0.2 ($0.1 - $0.3)	$0.4 ($0.2 - $0.7)
Southeast Asia, East Asia, and Oceania	$4.3 ($2.1 - $7.8)	$5.9 ($2.4 - $10.7)
Sub-Saharan Africa	$0.2 ($0.1 - $0.3)	$0.3 ($0.2 - $0.5)

Notes: All spending measured in 2019 US dollars.

Table S7: Attributable dementia spending by GBD regions and countries in 2000 (2019 USD)

	Spending attributable to dementia, 2000 (Thousand)	Spending per prevalent case,2000 (USD)	Spending per person,2000 (USD)	Spending per person over 65 years old (USD)	Spending as a percent of THE, 2000
Central Asia	$94 784 ($54 289 - $147 485)	$320.00 ($186.15 - $486.76)	$1.27 ($0.73 - $1.98)	$16.72 ($9.58 - $26.02)	1.7% (1.0% - 2.7%)
Armenia	$5213 ($2449 - $9315)	$281.08 ($138.09 - $496.03)	$1.57 ($0.74 - $2.81)	$15.30 ($7.19 - $27.33)	2.4% (1.1% - 4.3%)
Azerbaijan	$6287 ($3068 - $11 368)	$205.78 ($101.84 - $367.70)	$0.76 ($0.37 - $1.38)	$10.73 ($5.24 - $19.40)	1.9% (1.0% - 3.5%)
Georgia	$18 415 ($8927 - $33 796)	$384.48 ($181.73 - $693.08)	$3.89 ($1.89 - $7.15)	$27.92 ($13.53 - $51.23)	4.2% (2.0% - 7.8%)
Kazakhstan	$39 096 ($18 316 - $72 860)	$537.76 ($262.11 - $1004.40)	$2.61 ($1.22 - $4.86)	$31.61 ($14.81 - $58.90)	1.6% (0.7% - 2.9%)
Kyrgyzstan	$4769 ($2305 - $8353)	$200.18 ($96.80 - $341.25)	$0.95 ($0.46 - $1.66)	$12.21 ($5.90 - $21.38)	2.4% (1.2% - 4.2%)
Mongolia	$1424 ($695 - $2490)	$258.31 ($132.32 - $459.45)	$0.58 ($0.28 - $1.02)	$10.42 ($5.09 - $18.22)	0.9% (0.4% - 1.5%)
Tajikistan	$1613 ($753 - $2816)	$84.92 ($40.99 - $145.48)	$0.25 ($0.12 - $0.44)	$3.73 ($1.74 - $6.52)	1.9% (0.9% - 3.5%)
Turkmenistan	$5771 ($2904 - $10 261)	$472.39 ($230.45 - $831.30)	$1.37 ($0.69 - $2.43)	$20.92 ($10.53 - $37.20)	0.9% (0.4% - 1.6%)
Uzbekistan	$12 194 ($5865 - $21 882)	$185.02 ($91.09 - $334.17)	$0.48 ($0.23 - $0.87)	$7.57 ($3.64 - $13.59)	1.3% (0.6% - 2.4%)
Central Europe	$1 421 401 ($811 542 - $2 166 303)	$1150.87 ($682.75 - $1723.28)	$11.79 ($6.73 - $17.97)	$83.91 ($47.91 - $127.88)	2.8% (1.6% - 4.2%)
Albania	$8367 ($4123 - $14 989)	$466.28 ($228.80 - $791.87)	$2.62 ($1.29 - $4.70)	$29.79 ($14.68 - $53.37)	1.5% (0.7% - 2.8%)
Bosnia and Herzegovina	$18 531 ($8790 - $34 207)	$610.19 ($295.51 - $1086.51)	$4.53 ($2.15 - $8.35)	$38.44 ($18.23 - $70.95)	2.1% (1.0% - 3.8%)
Bulgaria	$77 593 ($37 613 - $137 193)	$824.42 ($404.68 - $1403.11)	$9.77 ($4.73 - $17.27)	$55.84 ($27.07 - $98.73)	3.6% (1.7% - 6.2%)
Croatia	$80 056 ($35 856 - $144 331)	$1475.88 ($657.51 - $2607.47)	$17.52 ($7.85 - $31.59)	$108.52 ($48.60 - $195.65)	2.7% (1.2% - 4.8%)
Czechia	$243 252 ($113 492 - $466 160)	$1964.88 ($920.89 - $3680.15)	$23.75 ($11.08 - $45.52)	$161.19 ($75.21 - $308.91)	2.9% (1.4% - 5.6%)
Hungary	$195 116 ($89 391 - $358 407)	$1533.46 ($728.90 - $2765.31)	$19.13 ($8.76 - $35.13)	$120.15 ($55.05 - $220.70)	2.9% (1.3% - 5.2%)
Montenegro	$4569 ($2196 - $8456)	$848.40 ($408.50 - $1555.62)	$7.19 ($3.46 - $13.31)	$58.36 ($28.04 - $108.01)	2.6% (1.3% - 5.0%)
North Macedonia	$11 357 ($5440 - $20 909)	$835.78 ($399.44 - $1521.44)	$5.67 ($2.71 - $10.43)	$50.13 ($24.02 - $92.30)	1.8% (0.8% - 3.3%)
Poland	$434 752 ($207 412 - $789 856)	$1115.97 ($536.96 - $2013.36)	$11.35 ($5.42 - $20.63)	$85.51 ($40.80 - $155.36)	2.7% (1.3% - 5.0%)
Romania	$155 068 ($73 432 - $284 292)	$717.24 ($344.44 - $1330.38)	$6.91 ($3.27 - $12.68)	$48.57 ($23.00 - $89.04)	3.0% (1.4% - 5.5%)
Serbia	$53 324 ($24 681 - $102 321)	$627.53 ($293.77 - $1210.66)	$5.57 ($2.58 - $10.69)	$39.18 ($18.13 - $75.18)	3.1% (1.4% - 5.7%)
Slovakia	$75 418 ($36 067 - $137 956)	$1415.44 ($697.43 - $2472.22)	$13.99 ($6.69 - $25.60)	$111.37 ($53.26 - $203.73)	2.6% (1.2% - 4.8%)
Slovenia	$63 993 ($29 336 - $126 565)	$2637.75 ($1205.05 - $5114.93)	$32.12 ($14.72 - $63.53)	$215.57 ($98.82 - $426.35)	2.4% (1.1% - 4.6%)

Eastern Europe	$1 866 721 ($979 606 - $3 218 737)	$922.13 ($476.07 - $1594.73)	$8.47 ($4.44 - $14.60)	$62.35 ($32.72 - $107.51)	3.2% (1.6% - 5.6%)
Belarus	$57 931 ($28 147 - $104 417)	$563.02 ($267.07 - $1018.40)	$5.66 ($2.75 - $10.20)	$39.72 ($19.30 - $71.59)	3.8% (1.8% - 7.0%)
Estonia	$26 770 ($12 059 - $50 687)	$1633.83 ($755.00 - $3063.45)	$19.21 ($8.65 - $36.37)	$121.70 ($54.82 - $230.43)	3.3% (1.5% - 6.2%)
Latvia	$38 777 ($17 059 - $72 730)	$1362.93 ($587.54 - $2536.71)	$16.26 ($7.15 - $30.50)	$103.30 ($45.44 - $193.74)	3.8% (1.7% - 7.1%)
Lithuania	$53 782 ($24 019 - $98 670)	$1400.87 ($627.65 - $2490.77)	$15.28 ($6.82 - $28.03)	$104.03 ($46.46 - $190.85)	3.4% (1.5% - 6.2%)
Republic of Moldova	$10 936 ($5045 - $19 697)	$362.94 ($171.65 - $661.03)	$2.61 ($1.20 - $4.70)	$23.18 ($10.69 - $41.74)	3.2% (1.5% - 5.9%)
Russian Federation	$1 400 684 ($650 218 - $2 657 743)	$1072.34 ($494.25 - $2027.24)	$9.40 ($4.36 - $17.83)	$71.42 ($33.15 - $135.52)	3.0% (1.4% - 5.6%)
Ukraine	$277 838 ($131 676 - $515 395)	$553.52 ($256.48 - $1009.28)	$5.59 ($2.65 - $10.38)	$38.15 ($18.08 - $70.76)	4.7% (2.2% - 8.7%)
Australasia	$738 061 ($387 403 - $1 239 165)	$3153.73 ($1697.35 - $5189.14)	$32.55 ($17.09 - $54.66)	$239.85 ($125.90 - $402.70)	1.0% (0.5% - 1.7%)
Australia	$625 824 ($314 603 - $1 088 290)	$3199.63 ($1649.21 - $5501.55)	$33.14 ($16.66 - $57.63)	$243.36 ($122.34 - $423.19)	1.0% (0.5% - 1.7%)
New Zealand	$112 237 ($52 763 - $196 943)	$2921.08 ($1375.62 - $5060.04)	$29.65 ($13.94 - $52.02)	$222.02 ($104.37 - $389.57)	1.2% (0.6% - 2.2%)
High-income Asia Pacific	$7 227 528 ($3 465 576 - $12 731 229)	$3501.58 ($1767.88 - $6105.82)	$40.11 ($19.23 - $70.66)	$260.87 ($125.09 - $459.53)	2.1% (1.0% - 3.7%)
Brunei Darussalam	$842 ($388 - $1514)	$1590.23 ($767.53 - $2837.52)	$2.53 ($1.17 - $4.55)	$54.54 ($25.12 - $98.13)	0.3% (0.1% - 0.5%)
Japan	$6 692 188 ($3 172 247 - $11 995 519)	$3729.42 ($1814.63 - $6606.87)	$51.86 ($24.59 - $92.97)	$285.82 ($135.48 - $512.32)	2.2% (1.0% - 3.9%)
Republic of Korea	$509 127 ($231 754 - $886 904)	$2013.38 ($923.89 - $3551.51)	$10.88 ($4.95 - $18.96)	$128.45 ($58.47 - $223.76)	1.6% (0.7% - 2.7%)
Singapore	$25 369 ($12 925 - $43 739)	$1576.38 ($791.96 - $2653.84)	$6.30 ($3.21 - $10.86)	$81.40 ($41.47 - $140.33)	0.5% (0.3% - 0.9%)
High-income North America	$73 914 781 ($64 574 054 - $84 625 516)	$18337.96 ($15528.44 - $21545.50)	$237.64 ($207.61 - $272.07)	$1724.98 ($1506.99 - $1974.94)	3.8% (3.3% - 4.4%)
Canada	$1 546 329 ($662 579 - $2 869 246)	$4824.44 ($2000.16 - $8673.86)	$51.03 ($21.87 - $94.69)	$370.86 ($158.91 - $688.13)	1.6% (0.7% - 2.9%)
Greenland	$1109 ($519 - $1967)	$5483.66 ($2640.44 - $9751.46)	$19.73 ($9.24 - $34.99)	$294.42 ($137.84 - $522.29)	0.5% (0.2% - 0.8%)
United States of America	$72 367 342 ($62 834 954 - $83 161 504)	$19502.18 ($16441.01 - $22964.82)	$257.83 ($223.87 - $296.28)	$1871.10 ($1624.64 - $2150.19)	3.9% (3.4% - 4.5%)
Southern Latin America	$318 280 ($177 501 - $530 411)	$816.41 ($447.16 - $1324.95)	$5.76 ($3.21 - $9.60)	$51.79 ($28.88 - $86.30)	0.8% (0.4% - 1.4%)
Argentina	$233 915 ($114 559 - $415 165)	$851.25 ($417.93 - $1516.81)	$6.36 ($3.11 - $11.28)	$54.68 ($26.78 - $97.04)	0.9% (0.4% - 1.6%)
Chile	$52 655 ($25 221 - $95 756)	$653.58 ($313.24 - $1192.34)	$3.48 ($1.67 - $6.33)	$37.90 ($18.16 - $68.93)	0.5% (0.2% - 0.9%)
Uruguay	$31 708 ($16 413 - $57 048)	$919.53 ($479.56 - $1603.91)	$9.61 ($4.98 - $17.30)	$66.26 ($34.30 - $119.22)	1.0% (0.5% - 1.7%)
Western Europe	$23 275 954 ($13 795 957 - $33 781 659)	$4545.91 ($2717.53 - $6495.91)	$58.38 ($34.61 - $84.74)	$337.88 ($200.26 - $490.38)	2.0% (1.2% - 2.9%)

Andorra	$2472 ($1227 - $4560)	$4394.23 ($2127.60 - $8116.69)	$37.81 ($18.76 - $69.74)	$295.44 ($146.59 - $544.95)	1.6% (0.8% - 2.9%)
Austria	$489 174 ($235 711 - $878 218)	$4885.92 ($2419.94 - $8640.96)	$60.98 ($29.39 - $109.49)	$370.12 ($178.34 - $664.48)	1.6% (0.8% - 2.8%)
Belgium	$571 097 ($284 120 - $1 011 103)	$4184.19 ($2099.36 - $7407.78)	$55.67 ($27.70 - $98.56)	$309.86 ($154.15 - $548.59)	1.8% (0.9% - 3.2%)
Cyprus	$11 161 ($5412 - $19 898)	$1921.95 ($935.05 - $3396.63)	$12.16 ($5.90 - $21.68)	$106.45 ($51.62 - $189.78)	1.3% (0.6% - 2.4%)
Denmark	$359 569 ($180 297 - $637 434)	$5519.47 ($2762.92 - $9827.70)	$67.38 ($33.78 - $119.45)	$416.40 ($208.79 - $738.19)	1.6% (0.8% - 2.9%)
Finland	$315 098 ($160 930 - $541 885)	$5003.13 ($2599.36 - $8588.29)	$60.72 ($31.01 - $104.42)	$374.89 ($191.47 - $644.71)	2.2% (1.1% - 3.7%)
France	$3 144 745 ($1 600 740 - $5 433 224)	$4744.95 ($2481.49 - $8044.93)	$52.46 ($26.70 - $90.64)	$307.65 ($156.60 - $531.52)	1.5% (0.8% - 2.7%)
Germany	$4 800 284 ($2 162 403 - $8 602 346)	$4227.38 ($1960.04 - $7492.56)	$58.31 ($26.27 - $104.50)	$331.97 ($149.54 - $594.91)	1.6% (0.7% - 2.8%)
Greece	$288 385 ($137 623 - $504 855)	$2356.01 ($1084.64 - $4039.60)	$26.00 ($12.41 - $45.52)	$151.09 ($72.10 - $264.51)	1.8% (0.9% - 3.2%)
Iceland	$13 063 ($6333 - $24 368)	$5111.68 ($2539.15 - $9017.22)	$46.76 ($22.67 - $87.23)	$355.65 ($172.41 - $663.45)	1.1% (0.5% - 2.0%)
Ireland	$122 423 ($52 938 - $234 269)	$3866.71 ($1679.53 - $7146.73)	$31.66 ($13.69 - $60.58)	$253.57 ($109.65 - $485.22)	1.2% (0.5% - 2.3%)
Israel	$165 361 ($78 530 - $298 894)	$3687.82 ($1796.30 - $6670.67)	$25.86 ($12.28 - $46.75)	$220.62 ($104.77 - $398.78)	1.2% (0.6% - 2.1%)
Italy	$3 385 677 ($1 674 836 - $6 370 390)	$3560.35 ($1769.05 - $6644.23)	$59.75 ($29.56 - $112.43)	$310.37 ($153.54 - $583.99)	2.3% (1.2% - 4.4%)
Luxembourg	$26 025 ($11 992 - $49 597)	$6581.21 ($3066.35 - $12203.77)	$60.02 ($27.66 - $114.39)	$394.32 ($181.70 - $751.46)	1.1% (0.5% - 2.2%)
Malta	$7586 ($3741 - $12 965)	$2089.00 ($1040.37 - $3449.52)	$18.87 ($9.30 - $32.24)	$139.75 ($68.91 - $238.85)	1.6% (0.8% - 2.7%)
Netherlands	$994 830 ($509 828 - $1 717 035)	$5563.93 ($2833.43 - $9773.59)	$62.60 ($32.08 - $108.04)	$419.88 ($215.18 - $724.70)	1.8% (0.9% - 3.1%)
Norway	$349 722 ($163 868 - $620 901)	$5736.45 ($2777.21 - $10201.22)	$78.08 ($36.58 - $138.62)	$467.64 ($219.12 - $830.25)	1.5% (0.7% - 2.6%)
Portugal	$260 116 ($125 484 - $462 813)	$2236.94 ($1107.11 - $3962.07)	$24.67 ($11.90 - $43.89)	$145.70 ($70.29 - $259.24)	1.6% (0.8% - 2.8%)
Spain	$1 438 150 ($693 627 - $2 604 728)	$2653.07 ($1303.21 - $4805.35)	$35.24 ($17.00 - $63.83)	$198.29 ($95.64 - $359.14)	2.1% (1.0% - 3.7%)
Sweden	$727 757 ($324 590 - $1 290 660)	$5670.30 ($2564.55 - $9808.41)	$81.73 ($36.45 - $144.94)	$445.11 ($198.53 - $789.40)	2.7% (1.2% - 4.9%)
Switzerland	$573 201 ($281 948 - $1 029 019)	$5868.28 ($2886.32 - $10455.62)	$78.54 ($38.63 - $140.99)	$476.97 ($234.61 - $856.27)	1.2% (0.6% - 2.1%)
United Kingdom	$5 230 047 ($2 490 009 - $9 477 657)	$7847.28 ($3665.48 - $14130.63)	$87.81 ($41.81 - $159.13)	$521.61 ($248.34 - $945.25)	3.5% (1.7% - 6.3%)
Andean Latin America	$57 316 ($32 649 - $90 251)	$397.75 ($230.18 - $623.25)	$1.24 ($0.71 - $1.95)	$15.88 ($9.04 - $25.00)	0.7% (0.4% - 1.1%)
Bolivia (Plurinational State of)	$5472 ($2807 - $9542)	$274.01 ($142.38 - $471.21)	$0.66 ($0.34 - $1.14)	$8.80 ($4.51 - $15.34)	0.6% (0.3% - 1.0%)
Ecuador	$16 683 ($8415 - $28 714)	$407.01 ($204.95 - $701.02)	$1.35 ($0.68 - $2.33)	$17.12 ($8.64 - $29.47)	0.7% (0.4% - 1.3%)
Peru	$35 161 ($17 041 - $60 112)	$422.86 ($210.81 - $719.48)	$1.38 ($0.67 - $2.36)	$17.46 ($8.46 - $29.85)	0.7% (0.4% - 1.3%)

Caribbean	$120 668 ($72 338 - $180 418)	$692.25 ($421.34 - $1017.41)	$3.11 ($1.87 - $4.65)	$33.59 ($20.14 - $50.23)	1.0% (0.6% - 1.6%)
Antigua and Barbuda	$410 ($199 - $702)	$1135.60 ($560.89 - $1970.41)	$5.34 ($2.60 - $9.14)	$61.15 ($29.75 - $104.77)	0.7% (0.4% - 1.3%)
Bahamas	$2050 ($1023 - $3742)	$2034.45 ($1005.78 - $3621.46)	$6.62 ($3.30 - $12.08)	$96.65 ($48.24 - $176.44)	0.5% (0.2% - 0.8%)
Barbados	$3047 ($1508 - $5452)	$1480.19 ($744.40 - $2560.63)	$11.85 ($5.86 - $21.21)	$88.48 ($43.78 - $158.32)	1.2% (0.6% - 2.1%)
Belize	$247 ($126 - $440)	$393.54 ($201.44 - $668.40)	$1.03 ($0.52 - $1.83)	$15.09 ($7.69 - $26.87)	0.6% (0.3% - 1.0%)
Bermuda	$1676 ($840 - $2959)	$3828.42 ($1887.91 - $6740.09)	$26.47 ($13.27 - $46.73)	$220.54 ($110.55 - $389.36)	0.6% (0.3% - 1.1%)
Cuba	$44 985 ($22 388 - $80 962)	$631.65 ($313.76 - $1099.97)	$3.95 ($1.97 - $7.11)	$35.49 ($17.66 - $63.88)	1.2% (0.6% - 2.2%)
Dominica	$383 ($189 - $685)	$738.97 ($369.20 - $1292.80)	$5.39 ($2.66 - $9.64)	$43.53 ($21.44 - $77.80)	1.6% (0.8% - 2.8%)
Dominican Republic	$10 614 ($5244 - $18 180)	$369.28 ($180.08 - $625.85)	$1.23 ($0.61 - $2.10)	$15.47 ($7.64 - $26.50)	0.6% (0.3% - 1.1%)
Grenada	$307 ($150 - $542)	$700.09 ($348.61 - $1182.21)	$3.00 ($1.46 - $5.28)	$39.11 ($19.11 - $68.89)	0.7% (0.4% - 1.3%)
Guyana	$529 ($272 - $915)	$311.71 ($159.07 - $527.94)	$0.69 ($0.35 - $1.19)	$10.77 ($5.53 - $18.64)	0.6% (0.3% - 1.0%)
Haiti	$1979 ($1021 - $3526)	$125.57 ($64.01 - $217.91)	$0.24 ($0.12 - $0.43)	$3.43 ($1.77 - $6.11)	0.6% (0.3% - 1.1%)
Jamaica	$7919 ($3971 - $14 133)	$580.96 ($290.46 - $1018.22)	$3.02 ($1.51 - $5.39)	$32.15 ($16.12 - $57.38)	1.1% (0.5% - 1.9%)
Puerto Rico	$39 334 ($19 017 - $68 334)	$1350.24 ($647.87 - $2473.83)	$10.15 ($4.91 - $17.63)	$80.96 ($39.14 - $140.65)	1.3% (0.6% - 2.4%)
Saint Lucia	$482 ($234 - $833)	$799.54 ($396.77 - $1375.76)	$3.10 ($1.50 - $5.36)	$36.32 ($17.62 - $62.75)	0.6% (0.3% - 1.1%)
Saint Vincent and the Grenadines	$216 ($108 - $377)	$476.54 ($243.27 - $834.25)	$1.96 ($0.98 - $3.42)	$22.19 ($11.06 - $38.72)	0.8% (0.4% - 1.4%)
Suriname	$710 ($355 - $1214)	$441.08 ($222.36 - $745.44)	$1.58 ($0.79 - $2.70)	$19.56 ($9.78 - $33.46)	0.6% (0.3% - 1.1%)
Trinidad and Tobago	$5123 ($2623 - $8900)	$935.60 ($481.08 - $1615.12)	$3.96 ($2.03 - $6.88)	$47.84 ($24.50 - $83.12)	0.8% (0.4% - 1.4%)
United States Virgin Islands	$656 ($315 - $1154)	$1189.28 ($583.23 - $2110.96)	$5.91 ($2.84 - $10.40)	$59.21 ($28.42 - $104.15)	1.1% (0.5% - 2.0%)
Central Latin America	$305 140 ($182 462 - $465 867)	$524.58 ($323.15 - $782.20)	$1.53 ($0.92 - $2.34)	$21.17 ($12.66 - $32.31)	0.4% (0.3% - 0.7%)
Colombia	$57 314 ($30 390 - $100 627)	$412.25 ($220.68 - $724.85)	$1.44 ($0.76 - $2.52)	$18.66 ($9.89 - $32.76)	0.6% (0.3% - 1.0%)
Costa Rica	$11 072 ($5380 - $20 032)	$754.89 ($374.02 - $1349.07)	$2.84 ($1.38 - $5.14)	$38.43 ($18.67 - $69.52)	0.6% (0.3% - 1.0%)
El Salvador	$10 390 ($5251 - $18 670)	$439.71 ($221.94 - $788.04)	$1.79 ($0.90 - $3.21)	$21.66 ($10.95 - $38.92)	0.6% (0.3% - 1.1%)
Guatemala	$7190 ($3664 - $12 387)	$323.03 ($167.69 - $561.78)	$0.66 ($0.34 - $1.14)	$9.09 ($4.63 - $15.66)	0.3% (0.2% - 0.6%)
Honduras	$3270 ($1718 - $5611)	$233.38 ($122.39 - $393.14)	$0.53 ($0.28 - $0.91)	$7.81 ($4.10 - $13.40)	0.5% (0.2% - 0.8%)
Mexico	$156 476 ($78 374 - $276 682)	$583.20 ($307.08 - $1030.88)	$1.55 ($0.77 - $2.73)	$21.66 ($10.85 - $38.30)	0.4% (0.2% - 0.7%)
Nicaragua	$2176 ($1052 - $3669)	$192.73 ($94.90 - $332.85)	$0.44 ($0.21 - $0.75)	$7.20 ($3.48 - $12.14)	0.5% (0.3% - 0.9%)

Panama	$9476 ($4731 - $16 277)	$752.02 ($381.51 - $1264.07)	$3.26 ($1.63 - $5.59)	$39.81 ($19.87 - $68.39)	0.6% (0.3% - 1.1%)
Venezuela (Bolivarian Republic of)	$47 773 ($25 297 - $80 032)	$629.35 ($336.24 - $1050.84)	$2.05 ($1.09 - $3.44)	$29.79 ($15.77 - $49.90)	0.5% (0.3% - 0.9%)
Tropical Latin America	$611 781 ($318 444 - $1 071 520)	$693.46 ($368.04 - $1219.39)	$3.41 ($1.78 - $5.97)	$44.43 ($23.13 - $77.81)	0.6% (0.3% - 1.1%)
Brazil	$603 546 ($308 117 - $1 061 158)	$700.59 ($368.61 - $1241.16)	$3.46 ($1.77 - $6.09)	$45.05 ($23.00 - $79.21)	0.6% (0.3% - 1.1%)
Paraguay	$8235 ($4074 - $14 622)	$397.17 ($197.85 - $697.87)	$1.61 ($0.80 - $2.85)	$22.06 ($10.91 - $39.17)	0.8% (0.4% - 1.5%)
North Africa and Middle East	$470 854 ($306 439 - $681 017)	$334.75 ($217.04 - $472.83)	$1.09 ($0.71 - $1.58)	$15.81 ($10.29 - $22.86)	0.5% (0.3% - 0.8%)
Afghanistan	$2971 ($1489 - $5293)	$64.47 ($33.63 - $111.09)	$0.15 ($0.08 - $0.27)	$1.98 ($0.99 - $3.54)	0.7% (0.3% - 1.3%)
Algeria	$23 326 ($12 106 - $42 945)	$235.95 ($125.05 - $423.08)	$0.75 ($0.39 - $1.38)	$11.48 ($5.96 - $21.14)	0.7% (0.4% - 1.3%)
Bahrain	$1223 ($618 - $2148)	$1077.70 ($552.37 - $1932.58)	$1.89 ($0.95 - $3.31)	$42.79 ($21.63 - $75.15)	0.2% (0.1% - 0.4%)
Egypt	$51 106 ($25 555 - $92 477)	$247.79 ($124.02 - $440.70)	$0.75 ($0.38 - $1.37)	$11.31 ($5.66 - $20.46)	0.7% (0.4% - 1.3%)
Iran (Islamic Republic of)	$94 098 ($46 223 - $162 376)	$432.31 ($215.18 - $731.27)	$1.40 ($0.69 - $2.41)	$21.30 ($10.46 - $36.76)	0.5% (0.3% - 0.9%)
Iraq	$13 691 ($7042 - $24 875)	$204.53 ($103.69 - $371.99)	$0.51 ($0.26 - $0.92)	$7.95 ($4.09 - $14.44)	0.5% (0.3% - 0.9%)
Jordan	$6086 ($3187 - $10 991)	$603.78 ($315.14 - $1056.63)	$1.29 ($0.67 - $2.32)	$22.31 ($11.68 - $40.29)	0.3% (0.2% - 0.6%)
Kuwait	$7739 ($3802 - $13 861)	$1403.19 ($697.90 - $2419.74)	$4.05 ($1.99 - $7.25)	$71.75 ($35.25 - $128.51)	0.3% (0.2% - 0.6%)
Lebanon	$15 490 ($7775 - $27 441)	$725.15 ($362.36 - $1274.27)	$4.18 ($2.10 - $7.41)	$40.65 ($20.40 - $72.01)	0.4% (0.2% - 0.8%)
Libya	$17 883 ($9023 - $31 164)	$1037.17 ($551.62 - $1749.20)	$3.52 ($1.78 - $6.13)	$56.94 ($28.73 - $99.22)	0.6% (0.3% - 1.0%)
Morocco	$20 449 ($10 580 - $36 106)	$178.75 ($94.53 - $316.55)	$0.69 ($0.36 - $1.21)	$9.12 ($4.72 - $16.10)	0.9% (0.4% - 1.6%)
Oman	$3306 ($1680 - $5805)	$763.55 ($399.71 - $1315.93)	$1.44 ($0.73 - $2.52)	$29.41 ($14.95 - $51.64)	0.2% (0.1% - 0.4%)
Palestine	$2745 ($1413 - $4643)	$388.09 ($198.76 - $656.65)	$0.90 ($0.46 - $1.52)	$12.90 ($6.64 - $21.82)	0.3% (0.2% - 0.6%)
Qatar	$1005 ($491 - $1738)	$1530.43 ($763.44 - $2633.34)	$1.71 ($0.84 - $2.96)	$53.73 ($26.28 - $92.97)	0.1% (0.1% - 0.2%)
Saudi Arabia	$42 683 ($21 712 - $77 322)	$964.82 ($490.49 - $1681.69)	$2.04 ($1.04 - $3.70)	$40.22 ($20.46 - $72.85)	0.2% (0.1% - 0.4%)
Sudan	$7283 ($3555 - $13 191)	$94.99 ($46.01 - $169.25)	$0.27 ($0.13 - $0.48)	$3.54 ($1.73 - $6.42)	0.8% (0.4% - 1.4%)
Syrian Arab Republic	$6358 ($3345 - $10 998)	$170.55 ($90.68 - $288.54)	$0.38 ($0.20 - $0.66)	$6.15 ($3.23 - $10.63)	0.6% (0.3% - 1.0%)
Tunisia	$12 821 ($6527 - $21 811)	$265.83 ($136.59 - $449.53)	$1.30 ($0.66 - $2.20)	$15.84 ($8.06 - $26.94)	1.2% (0.6% - 2.0%)
Turkey	$131 388 ($68 925 - $222 573)	$382.48 ($199.24 - $645.24)	$1.90 ($1.00 - $3.21)	$23.53 ($12.34 - $39.86)	0.9% (0.5% - 1.5%)
United Arab Emirates	$4000 ($2149 - $7147)	$1768.92 ($949.16 - $3126.24)	$1.24 ($0.67 - $2.21)	$52.22 ($28.06 - $93.31)	0.1% (0.0% - 0.1%)
Yemen	$5198 ($2717 - $9067)	$141.01 ($72.94 - $248.92)	$0.28 ($0.15 - $0.49)	$4.05 ($2.11 - $7.06)	0.4% (0.2% - 0.8%)
South Asia	$178 682 ($97 843 - $291 670)	$84.43 ($47.11 - $136.29)	$0.13 ($0.07 - $0.22)	$1.98 ($1.09 - $3.24)	0.5% (0.3% - 0.8%)

Bangladesh	$12 354<br>($6246 - $21 149)	$56.03<br>($28.55 - $96.89)	$0.10<br>($0.05 - $0.17)	$1.60<br>($0.81 - $2.74)	0.6%<br>(0.3% - 1.1%)
Bhutan	$144<br>($71 - $241)	$127.25<br>($64.12 - $215.93)	$0.22<br>($0.11 - $0.37)	$3.33<br>($1.64 - $5.58)	0.3%<br>(0.2% - 0.6%)
India	$147 518<br>($76 995 - $256 488)	$90.49<br>($47.80 - $155.27)	$0.14<br>($0.07 - $0.25)	$2.10<br>($1.10 - $3.65)	0.5%<br>(0.2% - 0.8%)
Nepal	$2514<br>($1215 - $4601)	$60.94<br>($30.78 - $108.46)	$0.10<br>($0.05 - $0.19)	$1.50<br>($0.73 - $2.75)	0.5%<br>(0.2% - 1.0%)
Pakistan	$16 151<br>($7900 - $28 695)	$72.27<br>($36.36 - $125.76)	$0.11<br>($0.06 - $0.20)	$1.56<br>($0.76 - $2.76)	0.5%<br>(0.2% - 0.8%)
East Asia	$2 466 454<br>($1 271 931 - $4 086 623)	$381.16<br>($192.57 - $631.78)	$1.83<br>($0.94 - $3.03)	$22.13<br>($11.41 - $36.67)	1.7%<br>(0.8% - 2.8%)
China	$2 183 387<br>($1 076 704 - $3 686 364)	$348.15<br>($170.73 - $599.44)	$1.68<br>($0.83 - $2.83)	$20.38<br>($10.05 - $34.42)	1.7%<br>(0.8% - 2.9%)
Democratic People's Republic of Korea	$23 058<br>($11 564 - $40 182)	$248.92<br>($124.93 - $436.13)	$0.96<br>($0.48 - $1.68)	$10.89<br>($5.46 - $18.98)	1.7%<br>(0.8% - 2.9%)
Taiwan (Province of China)	$260 008<br>($121 752 - $482 842)	$2430.86<br>($1194.52 - $4331.71)	$11.67<br>($5.47 - $21.67)	$117.03<br>($54.80 - $217.33)	1.5%<br>(0.7% - 2.7%)
Oceania	$4897<br>($2990 - $7165)	$347.20<br>($219.33 - $501.82)	$0.63<br>($0.38 - $0.92)	$9.85<br>($6.02 - $14.42)	0.6%<br>(0.4% - 0.9%)
American Samoa	$200<br>($96 - $348)	$1445.77<br>($705.03 - $2495.12)	$3.41<br>($1.64 - $5.95)	$51.22<br>($24.59 - $89.41)	0.6%<br>(0.3% - 1.0%)
Fiji	$800<br>($407 - $1424)	$491.17<br>($246.14 - $869.27)	$0.98<br>($0.50 - $1.75)	$17.38<br>($8.85 - $30.93)	0.7%<br>(0.3% - 1.2%)
Guam	$946<br>($453 - $1703)	$1845.06<br>($907.77 - $3257.74)	$5.96<br>($2.85 - $10.72)	$78.91<br>($37.79 - $142.01)	1.0%<br>(0.5% - 1.8%)
Kiribati	$82<br>($41 - $147)	$534.11<br>($271.64 - $922.02)	$0.94<br>($0.47 - $1.68)	$15.24<br>($7.57 - $27.26)	0.4%<br>(0.2% - 0.8%)
Marshall Islands	$54<br>($28 - $94)	$768.12<br>($378.13 - $1301.08)	$1.04<br>($0.53 - $1.81)	$20.72<br>($10.51 - $35.98)	0.3%<br>(0.1% - 0.5%)
Micronesia (Federated States of)	$72<br>($35 - $128)	$340.02<br>($170.52 - $600.07)	$0.65<br>($0.32 - $1.16)	$10.82<br>($5.31 - $19.34)	0.7%<br>(0.3% - 1.2%)
Northern Mariana Islands	$112<br>($55 - $195)	$1278.76<br>($613.93 - $2150.58)	$1.54<br>($0.75 - $2.68)	$31.87<br>($15.50 - $55.44)	0.4%<br>(0.2% - 0.7%)
Papua New Guinea	$1854<br>($940 - $3243)	$195.76<br>($101.50 - $344.95)	$0.33<br>($0.17 - $0.58)	$5.24<br>($2.66 - $9.17)	0.6%<br>(0.3% - 1.0%)
Samoa	$288<br>($145 - $508)	$544.15<br>($283.23 - $949.99)	$1.66<br>($0.84 - $2.93)	$24.29<br>($12.24 - $42.88)	1.1%<br>(0.5% - 1.9%)
Solomon Islands	$195<br>($100 - $336)	$310.21<br>($157.83 - $536.22)	$0.43<br>($0.22 - $0.75)	$6.28<br>($3.20 - $10.81)	0.4%<br>(0.2% - 0.7%)
Tonga	$192<br>($97 - $335)	$551.55<br>($267.49 - $927.65)	$1.90<br>($0.96 - $3.32)	$23.48<br>($11.86 - $41.02)	1.0%<br>(0.5% - 1.8%)
Vanuatu	$102<br>($49 - $171)	$313.90<br>($157.88 - $532.20)	$0.53<br>($0.25 - $0.89)	$8.33<br>($4.00 - $14.07)	0.5%<br>(0.2% - 0.9%)
Southeast Asia	$413 010<br>($245 494 - $607 873)	$268.12<br>($161.07 - $388.93)	$0.76<br>($0.45 - $1.11)	$10.89<br>($6.48 - $16.03)	1.4%<br>(0.8% - 2.0%)
Cambodia	$3816<br>($1908 - $6758)	$149.41<br>($78.02 - $262.65)	$0.30<br>($0.15 - $0.54)	$4.80<br>($2.40 - $8.50)	0.8%<br>(0.4% - 1.5%)
Indonesia	$118 004<br>($56 863 - $207 207)	$220.47<br>($108.02 - $389.42)	$0.55<br>($0.27 - $0.97)	$8.39<br>($4.04 - $14.73)	1.4%<br>(0.7% - 2.4%)
Lao People's Democratic Republic	$1724<br>($875 - $2949)	$157.72<br>($79.84 - $268.46)	$0.32<br>($0.16 - $0.55)	$4.73<br>($2.40 - $8.09)	0.8%<br>(0.4% - 1.4%)
Malaysia	$37 141<br>($17 976 - $65 836)	$589.24<br>($284.61 - $1032.93)	$1.56<br>($0.75 - $2.76)	$25.72<br>($12.45 - $45.59)	1.0%<br>(0.5% - 1.8%)
Maldives	$530<br>($258 - $941)	$957.73<br>($482.90 - $1629.79)	$1.87<br>($0.91 - $3.33)	$30.32<br>($14.80 - $53.90)	0.3%<br>(0.2% - 0.6%)
Mauritius	$2828<br>($1418 - $4908)	$553.43<br>($275.93 - $927.84)	$2.33<br>($1.17 - $4.04)	$29.32<br>($14.70 - $50.89)	1.4%<br>(0.7% - 2.4%)
Myanmar	$6640<br>($3488 - $11 813)	$51.82<br>($27.14 - $88.81)	$0.14<br>($0.08 - $0.25)	$1.91<br>($1.01 - $3.41)	2.2%<br>(1.1% - 4.0%)

Philippines	$47 340 ($22 865 - $84 084)	$263.28 ($125.93 - $477.10)	$0.59 ($0.29 - $1.05)	$9.07 ($4.38 - $16.10)	1.2% (0.5% - 2.1%)
Seychelles	$497 ($248 - $881)	$1229.84 ($617.99 - $2207.07)	$6.07 ($3.02 - $10.75)	$65.98 ($32.86 - $116.85)	1.1% (0.5% - 1.9%)
Sri Lanka	$24 394 ($12 069 - $42 920)	$344.47 ($171.28 - $606.25)	$1.30 ($0.64 - $2.28)	$16.91 ($8.37 - $29.76)	1.7% (0.9% - 3.1%)
Thailand	$116 825 ($57 790 - $206 595)	$481.64 ($239.37 - $856.31)	$1.86 ($0.92 - $3.29)	$24.25 ($12.00 - $42.89)	1.5% (0.7% - 2.6%)
Timor-Leste	$236 ($120 - $416)	$154.04 ($78.82 - $262.52)	$0.26 ($0.13 - $0.46)	$3.71 ($1.88 - $6.54)	0.6% (0.3% - 1.1%)
Viet Nam	$53 032 ($26 809 - $90 115)	$191.61 ($99.18 - $328.68)	$0.66 ($0.33 - $1.12)	$8.68 ($4.39 - $14.76)	1.7% (0.9% - 3.0%)
Central Sub-Saharan Africa	$10 435 ($6335 - $15 502)	$80.46 ($51.31 - $115.98)	$0.14 ($0.08 - $0.21)	$1.98 ($1.20 - $2.95)	0.5% (0.3% - 0.7%)
Angola	$2850 ($1446 - $4700)	$140.58 ($73.08 - $236.21)	$0.19 ($0.10 - $0.32)	$2.89 ($1.47 - $4.77)	0.3% (0.1% - 0.5%)
Central African Republic	$461 ($232 - $796)	$79.48 ($41.35 - $133.83)	$0.13 ($0.06 - $0.22)	$1.97 ($0.99 - $3.40)	0.6% (0.3% - 1.0%)
Congo	$935 ($479 - $1586)	$143.29 ($73.32 - $244.03)	$0.29 ($0.15 - $0.50)	$4.41 ($2.26 - $7.47)	0.6% (0.3% - 1.1%)
Democratic Republic of the Congo	$3962 ($1987 - $6866)	$42.87 ($21.45 - $73.24)	$0.08 ($0.04 - $0.13)	$1.08 ($0.54 - $1.86)	0.9% (0.4% - 1.6%)
Equatorial Guinea	$347 ($179 - $613)	$314.40 ($158.87 - $547.36)	$0.52 ($0.27 - $0.92)	$7.38 ($3.81 - $13.05)	0.3% (0.2% - 0.6%)
Gabon	$1882 ($942 - $3234)	$514.02 ($257.09 - $902.39)	$1.51 ($0.76 - $2.59)	$19.69 ($9.86 - $33.84)	0.6% (0.3% - 1.0%)
Eastern Sub-Saharan Africa	$30 293 ($19 948 - $43 282)	$84.02 ($55.55 - $115.56)	$0.12 ($0.08 - $0.17)	$1.79 ($1.18 - $2.55)	0.5% (0.3% - 0.7%)
Burundi	$727 ($356 - $1304)	$69.68 ($35.62 - $125.45)	$0.12 ($0.06 - $0.21)	$1.68 ($0.83 - $3.02)	0.6% (0.3% - 1.1%)
Comoros	$269 ($136 - $472)	$199.77 ($103.26 - $352.28)	$0.49 ($0.25 - $0.85)	$6.39 ($3.22 - $11.20)	0.6% (0.3% - 1.1%)
Djibouti	$128 ($68 - $217)	$173.47 ($90.65 - $296.23)	$0.20 ($0.11 - $0.34)	$3.30 ($1.77 - $5.60)	0.3% (0.2% - 0.5%)
Eritrea	$387 ($199 - $680)	$91.11 ($48.58 - $161.98)	$0.09 ($0.05 - $0.17)	$1.63 ($0.84 - $2.86)	0.4% (0.2% - 0.7%)
Ethiopia	$4309 ($2176 - $7504)	$48.23 ($24.91 - $82.30)	$0.06 ($0.03 - $0.11)	$0.96 ($0.48 - $1.67)	0.5% (0.3% - 0.9%)
Kenya	$8400 ($4162 - $14 161)	$180.69 ($94.21 - $314.25)	$0.27 ($0.13 - $0.46)	$4.29 ($2.13 - $7.23)	0.5% (0.2% - 0.8%)
Madagascar	$1903 ($996 - $3310)	$83.00 ($44.93 - $143.36)	$0.12 ($0.06 - $0.21)	$1.80 ($0.94 - $3.14)	0.6% (0.3% - 1.0%)
Malawi	$1227 ($611 - $2145)	$66.97 ($33.15 - $111.33)	$0.11 ($0.06 - $0.19)	$1.48 ($0.74 - $2.59)	0.7% (0.3% - 1.2%)
Mozambique	$1577 ($818 - $2796)	$54.36 ($28.18 - $92.44)	$0.09 ($0.05 - $0.16)	$1.24 ($0.64 - $2.20)	0.7% (0.3% - 1.1%)
Rwanda	$653 ($322 - $1142)	$60.98 ($30.99 - $105.19)	$0.08 ($0.04 - $0.14)	$1.30 ($0.64 - $2.27)	0.6% (0.3% - 1.1%)
Somalia	$291 ($144 - $514)	$27.23 ($13.59 - $48.88)	$0.03 ($0.01 - $0.05)	$0.44 ($0.22 - $0.77)	0.5% (0.2% - 0.9%)
South Sudan	$1190 ($625 - $2064)	$103.09 ($53.15 - $177.06)	$0.16 ($0.09 - $0.29)	$2.44 ($1.28 - $4.23)	0.4% (0.2% - 0.6%)
Uganda	$2977 ($1581 - $5273)	$91.55 ($47.06 - $156.70)	$0.12 ($0.07 - $0.22)	$1.72 ($0.91 - $3.05)	0.5% (0.2% - 0.8%)
United Republic of Tanzania	$4427 ($2287 - $7639)	$74.30 ($38.43 - $127.92)	$0.13 ($0.07 - $0.22)	$1.75 ($0.90 - $3.02)	0.7% (0.4% - 1.3%)
Zambia	$1830 ($995 - $3197)	$144.35 ($76.07 - $244.95)	$0.18 ($0.10 - $0.32)	$2.71 ($1.47 - $4.73)	0.3% (0.2% - 0.6%)
Southern Sub-Saharan Africa	$106 095 ($55 042 - $178 852)	$603.30 ($320.54 - $1014.30)	$1.66 ($0.86 - $2.80)	$24.02 ($12.46 - $40.50)	0.6% (0.3% - 1.0%)
Botswana	$1607 ($810 - $2731)	$500.06 ($254.69 - $857.70)	$0.95 ($0.48 - $1.62)	$15.85 ($7.99 - $26.95)	0.4% (0.2% - 0.7%)
Eswatini	$472 ($227 - $813)	$302.83 ($144.66 - $530.23)	$0.46 ($0.22 - $0.80)	$7.91 ($3.81 - $13.63)	0.4% (0.2% - 0.6%)
Lesotho	$755 ($382 - $1275)	$138.47 ($68.90 - $230.87)	$0.38 ($0.19 - $0.64)	$5.14 ($2.60 - $8.68)	0.8% (0.4% - 1.4%)

Namibia	$2766<br>($1440 - $4829)	$600.88<br>($323.06 - $1010.50)	$1.50<br>($0.78 - $2.62)	$20.98<br>($10.92 - $36.62)	0.4%<br>(0.2% - 0.8%)
South Africa	$95 479<br>($47 065 - $165 920)	$689.91<br>($345.44 - $1218.28)	$2.10<br>($1.03 - $3.65)	$29.87<br>($14.73 - $51.91)	0.6%<br>(0.3% - 1.0%)
Zimbabwe	$5017<br>($2461 - $9211)	$221.53<br>($111.25 - $390.39)	$0.42<br>($0.21 - $0.77)	$6.43<br>($3.15 - $11.80)	0.5%<br>(0.2% - 0.9%)
Western Sub-Saharan Africa	$46 215<br>($28 338 - $70 284)	$121.63<br>($75.01 - $179.76)	$0.18<br>($0.11 - $0.27)	$2.42<br>($1.49 - $3.68)	0.5%<br>(0.3% - 0.7%)
Benin	$942<br>($486 - $1648)	$97.40<br>($51.05 - $166.11)	$0.14<br>($0.07 - $0.25)	$1.93<br>($1.00 - $3.38)	0.5%<br>(0.2% - 0.8%)
Burkina Faso	$1092<br>($577 - $1825)	$53.76<br>($27.73 - $90.40)	$0.09<br>($0.05 - $0.15)	$1.16<br>($0.61 - $1.93)	0.7%<br>(0.3% - 1.1%)
Cabo Verde	$410<br>($208 - $726)	$249.42<br>($129.45 - $447.28)	$0.91<br>($0.46 - $1.62)	$10.50<br>($5.33 - $18.58)	0.9%<br>(0.4% - 1.6%)
Cameroon	$3021<br>($1533 - $5152)	$151.10<br>($77.15 - $257.52)	$0.20<br>($0.10 - $0.34)	$2.89<br>($1.47 - $4.92)	0.4%<br>(0.2% - 0.7%)
Chad	$1074<br>($553 - $1850)	$81.70<br>($42.95 - $140.74)	$0.13<br>($0.07 - $0.22)	$1.60<br>($0.83 - $2.76)	0.4%<br>(0.2% - 0.8%)
CÃ´te d'Ivoire	$2934<br>($1496 - $5329)	$166.34<br>($84.18 - $289.62)	$0.17<br>($0.09 - $0.31)	$2.73<br>($1.39 - $4.95)	0.3%<br>(0.1% - 0.5%)
Gambia	$136<br>($70 - $229)	$70.45<br>($35.91 - $115.25)	$0.10<br>($0.05 - $0.17)	$1.37<br>($0.71 - $2.31)	0.6%<br>(0.3% - 1.0%)
Ghana	$3796<br>($1968 - $6819)	$129.53<br>($67.55 - $234.02)	$0.20<br>($0.10 - $0.36)	$2.92<br>($1.51 - $5.24)	0.6%<br>(0.3% - 1.0%)
Guinea	$1549<br>($773 - $2689)	$86.71<br>($44.84 - $146.20)	$0.19<br>($0.10 - $0.34)	$2.20<br>($1.10 - $3.82)	0.7%<br>(0.3% - 1.2%)
Guinea-Bissau	$169<br>($86 - $297)	$109.73<br>($56.87 - $188.61)	$0.13<br>($0.07 - $0.24)	$2.05<br>($1.05 - $3.61)	0.4%<br>(0.2% - 0.7%)
Liberia	$289<br>($148 - $520)	$58.39<br>($29.10 - $105.33)	$0.10<br>($0.05 - $0.18)	$1.28<br>($0.66 - $2.30)	0.7%<br>(0.3% - 1.2%)
Mali	$1773<br>($912 - $3030)	$95.12<br>($49.14 - $159.53)	$0.16<br>($0.08 - $0.27)	$1.97<br>($1.01 - $3.36)	0.5%<br>(0.2% - 0.9%)
Mauritania	$770<br>($396 - $1344)	$160.87<br>($83.26 - $276.76)	$0.29<br>($0.15 - $0.51)	$3.93<br>($2.02 - $6.86)	0.5%<br>(0.3% - 0.9%)
Niger	$819<br>($403 - $1411)	$66.74<br>($33.07 - $114.68)	$0.07<br>($0.04 - $0.13)	$0.98<br>($0.48 - $1.68)	0.3%<br>(0.2% - 0.6%)
Nigeria	$23 904<br>($11 945 - $43 539)	$136.35<br>($69.07 - $237.76)	$0.19<br>($0.10 - $0.35)	$2.63<br>($1.31 - $4.79)	0.5%<br>(0.2% - 0.9%)
Sao Tome and Principe	$83<br>($42 - $145)	$265.35<br>($132.06 - $456.92)	$0.58<br>($0.29 - $1.01)	$7.25<br>($3.65 - $12.69)	0.5%<br>(0.2% - 0.9%)
Senegal	$2017<br>($1039 - $3550)	$124.42<br>($64.89 - $213.76)	$0.20<br>($0.11 - $0.36)	$2.88<br>($1.48 - $5.07)	0.5%<br>(0.3% - 0.9%)
Sierra Leone	$955<br>($494 - $1707)	$108.95<br>($56.51 - $190.16)	$0.22<br>($0.11 - $0.39)	$2.69<br>($1.39 - $4.81)	0.6%<br>(0.3% - 1.1%)
Togo	$482<br>($240 - $829)	$85.00<br>($42.94 - $147.62)	$0.10<br>($0.05 - $0.17)	$1.59<br>($0.80 - $2.74)	0.5%<br>(0.2% - 0.8%)

Notes: All spending measured in 2019 US dollars.

Table S8: Attributable dementia spending by GBD regions and countries in 2019 (2019 USD)

	Spending attributable to dementia, 2019 (Thousand)	Spending per prevalent case, 2019 (USD)	Spending per person, 2019 (USD)	Spending per person over 65 years old (USD)	Spending as a percent of THE, 2019	Annualized rate of change from 2000 to 2019
Central Asia	$289 620 ($170 175 - $422 883)	$844.66 ($498.49 - $1229.36)	$3.10 ($1.82 - $4.52)	$42.02 ($24.69 - $61.35)	1.5% (0.9% - 2.3%)	6.1% (4.3% - 7.7%)
Armenia	$34 656 ($16 139 - $63 119)	$1211.10 ($566.15 - $2205.24)	$11.48 ($5.34 - $20.90)	$85.89 ($40.00 - $156.43)	2.7% (1.2% - 4.9%)	10.5% (6.9% - 14.4%)
Azerbaijan	$28 939 ($13 441 - $53 074)	$731.71 ($346.85 - $1313.11)	$2.82 ($1.31 - $5.16)	$39.39 ($18.29 - $72.23)	0.9% (0.4% - 1.7%)	8.3% (4.8% - 12.1%)
Georgia	$52 590 ($25 279 - $95 827)	$1056.78 ($504.68 - $1868.42)	$14.35 ($6.90 - $26.15)	$85.56 ($41.13 - $155.91)	4.3% (2.0% - 8.1%)	5.7% (2.0% - 9.3%)
Kazakhstan	$95 460 ($42 902 - $171 063)	$1077.63 ($494.01 - $1854.42)	$5.19 ($2.33 - $9.30)	$57.04 ($25.64 - $102.22)	1.9% (0.8% - 3.4%)	4.8% (1.3% - 8.1%)
Kyrgyzstan	$9156 ($4517 - $16 706)	$374.05 ($182.58 - $677.77)	$1.40 ($0.69 - $2.56)	$19.98 ($9.86 - $36.46)	1.7% (0.8% - 3.2%)	3.5% (0.2% - 7.2%)
Mongolia	$6133 ($2812 - $11 113)	$649.43 ($310.61 - $1143.14)	$1.81 ($0.83 - $3.28)	$28.41 ($13.03 - $51.48)	1.0% (0.5% - 1.9%)	8.0% (4.6% - 11.9%)
Tajikistan	$4926 ($2361 - $8792)	$272.69 ($132.61 - $495.58)	$0.52 ($0.25 - $0.93)	$9.30 ($4.46 - $16.59)	0.9% (0.4% - 1.7%)	6.0% (2.6% - 9.9%)
Turkmenistan	$28 235 ($13 460 - $50 420)	$1396.56 ($647.82 - $2471.98)	$5.55 ($2.65 - $9.92)	$75.34 ($35.92 - $134.53)	0.9% (0.4% - 1.7%)	8.7% (5.0% - 12.3%)
Uzbekistan	$29 524 ($14 242 - $53 192)	$460.33 ($211.75 - $837.25)	$0.88 ($0.42 - $1.58)	$15.64 ($7.54 - $28.18)	0.9% (0.4% - 1.7%)	4.8% (1.2% - 8.5%)
Central Europe	$4 382 529 ($2 399 304 - $6 753 711)	$2529.19 ($1424.57 - $3780.06)	$38.37 ($21.01 - $59.13)	$196.27 ($107.45 - $302.46)	3.9% (2.1% - 5.9%)	6.1% (4.6% - 7.7%)
Albania	$31 052 ($14 676 - $55 136)	$976.70 ($474.51 - $1772.69)	$11.41 ($5.40 - $20.27)	$71.36 ($33.73 - $126.71)	2.8% (1.3% - 5.0%)	7.1% (3.5% - 10.8%)
Bosnia and Herzegovina	$63 595 ($30 348 - $113 569)	$1558.26 ($762.17 - $2783.14)	$19.27 ($9.20 - $34.42)	$107.57 ($51.33 - $192.09)	3.3% (1.6% - 5.9%)	6.7% (3.2% - 10.4%)
Bulgaria	$233 429 ($112 296 - $446 575)	$2062.73 ($986.31 - $3925.23)	$33.66 ($16.19 - $64.40)	$150.49 ($72.39 - $287.89)	4.0% (1.9% - 7.8%)	6.0% (2.4% - 9.8%)
Croatia	$189 119 ($86 930 - $350 021)	$2531.33 ($1180.28 - $4693.21)	$44.52 ($20.46 - $82.40)	$210.39 ($96.71 - $389.39)	4.4% (2.0% - 8.1%)	4.6% (1.0% - 8.4%)
Czech Republic	$680 029 ($316 553 - $1 213 241)	$3893.63 ($1860.34 - $7267.16)	$63.89 ($29.74 - $113.99)	$301.39 ($140.30 - $537.70)	3.6% (1.7% - 6.5%)	5.6% (1.9% - 9.2%)
Hungary	$437 858 ($203 882 - $823 188)	$2680.21 ($1238.41 - $4981.21)	$45.26 ($21.07 - $85.09)	$216.23 ($100.68 - $406.52)	3.7% (1.7% - 7.0%)	4.3% (0.6% - 7.9%)
Macedonia	$26 191 ($12 008 - $46 520)	$1403.99 ($657.06 - $2513.27)	$12.17 ($5.58 - $21.61)	$82.99 ($38.05 - $147.40)	2.5% (1.0% - 4.6%)	4.5% (1.1% - 8.2%)
Montenegro	$14 482 ($6495 - $26 539)	$2115.66 ($942.95 - $3827.86)	$23.35 ($10.47 - $42.78)	$145.79 ($65.38 - $267.16)	3.2% (1.4% - 6.2%)	6.2% (2.7% - 9.8%)
Poland	$1 515 791 ($676 441 - $2 809 451)	$2593.02 ($1138.93 - $4887.72)	$39.44 ($17.60 - $73.10)	$210.54 ($93.95 - $390.22)	4.0% (1.8% - 7.5%)	6.8% (3.1% - 10.5%)
Romania	$607 281 ($279 769 - $1 139 288)	$1982.28 ($894.91 - $3523.19)	$31.57 ($14.54 - $59.22)	$156.82 ($72.24 - $294.19)	4.6% (2.1% - 8.6%)	7.4% (3.8% - 11.1%)
Serbia	$183 606 ($79 713 - $341 797)	$1690.53 ($729.19 - $3147.23)	$20.99 ($9.11 - $39.08)	$107.79 ($46.80 - $200.66)	4.1% (1.8% - 7.7%)	6.7% (3.1% - 10.4%)

Slovakia	$220 925 ($96 129 - $408 416)	$3212.94 ($1400.87 - $6018.68)	$40.63 ($17.68 - $75.11)	$234.21 ($101.91 - $432.97)	3.0% (1.3% - 5.6%)	5.8% (2.0% - 9.6%)
Slovenia	$179 166 ($82 144 - $337 209)	$4408.58 ($2050.30 - $8164.81)	$86.38 ($39.60 - $162.57)	$409.15 ($187.59 - $770.06)	3.9% (1.8% - 7.3%)	5.6% (1.8% - 9.1%)
Eastern Europe	$5 097 926 ($2 295 341 - $8 863 234)	$2026.63 ($959.31 - $3526.05)	$24.28 ($10.93 - $42.21)	$147.80 ($66.55 - $256.96)	4.7% (2.1% - 8.1%)	5.4% (2.5% - 8.3%)
Belarus	$179 743 ($83 164 - $336 829)	$1458.50 ($663.43 - $2733.97)	$18.92 ($8.75 - $35.45)	$116.13 ($53.73 - $217.63)	4.7% (2.1% - 8.8%)	6.1% (2.7% - 10.1%)
Estonia	$102 100 ($43 972 - $193 362)	$4253.22 ($1829.94 - $7949.18)	$77.80 ($33.51 - $147.34)	$371.78 ($160.12 - $704.09)	5.2% (2.2% - 9.8%)	7.3% (3.8% - 11.1%)
Latvia	$119 041 ($53 251 - $226 415)	$3453.09 ($1538.87 - $6414.09)	$62.15 ($27.80 - $118.21)	$289.26 ($129.40 - $550.18)	5.8% (2.6% - 11.1%)	6.1% (2.4% - 9.9%)
Lithuania	$196 231 ($81 728 - $370 559)	$3902.05 ($1685.10 - $7292.28)	$70.23 ($29.25 - $132.62)	$337.00 ($140.36 - $636.39)	5.5% (2.3% - 10.3%)	7.0% (3.2% - 10.7%)
Moldova	$39 846 ($17 929 - $75 780)	$946.29 ($421.11 - $1765.78)	$10.80 ($4.86 - $20.55)	$71.09 ($31.99 - $135.19)	4.6% (2.0% - 8.8%)	7.0% (3.2% - 10.8%)
Russian Federation	$3 798 972 ($1 547 981 - $7 121 138)	$2259.74 ($955.32 - $4291.11)	$25.89 ($10.55 - $48.54)	$161.69 ($65.88 - $303.09)	4.4% (1.8% - 8.3%)	5.4% (1.6% - 9.1%)
Ukraine	$661 991 ($279 546 - $1 285 964)	$1180.57 ($480.67 - $2223.10)	$15.03 ($6.35 - $29.20)	$86.87 ($36.69 - $168.76)	6.6% (2.7% - 12.7%)	4.6% (1.1% - 8.5%)
Australasia	$2 557 342 ($1 297 985 - $4 467 421)	$6750.75 ($3537.55 - $11685.26)	$87.99 ($44.66 - $153.71)	$498.53 ($253.03 - $870.88)	1.7% (0.9% - 3.0%)	6.7% (3.6% - 9.9%)
Australia	$2 187 080 ($1 046 571 - $3 948 569)	$6871.67 ($3402.13 - $12546.15)	$89.02 ($42.60 - $160.72)	$506.16 ($242.21 - $913.82)	1.7% (0.8% - 3.0%)	6.8% (3.3% - 10.5%)
New Zealand	$370 262 ($176 792 - $669 301)	$6115.65 ($3014.85 - $11134.10)	$82.36 ($39.33 - $148.88)	$457.78 ($218.58 - $827.49)	1.9% (0.9% - 3.5%)	6.5% (2.9% - 10.1%)
High-income Asia Pacific	$40 116 621 ($19 275 406 - $74 417 632)	$8774.54 ($4197.08 - $15957.91)	$214.19 ($102.92 - $397.34)	$867.95 ($417.04 - $1610.08)	5.6% (2.7% - 10.5%)	9.4% (6.0% - 13.0%)
Brunei	$2255 ($1043 - $4189)	$2255.66 ($1024.24 - $4196.97)	$5.16 ($2.39 - $9.58)	$89.36 ($41.34 - $166.03)	0.7% (0.3% - 1.3%)	5.3% (1.7% - 8.9%)
Japan	$34 803 080 ($16 216 157 - $67 062 585)	$8856.80 ($4029.47 - $17027.44)	$272.35 ($126.90 - $524.79)	$934.09 ($435.23 - $1799.91)	6.2% (2.9% - 12.0%)	9.0% (5.4% - 13.0%)
Singapore	$196 569 ($93 988 - $337 356)	$4370.57 ($2062.06 - $7265.64)	$34.68 ($16.58 - $59.53)	$267.47 ($127.89 - $459.04)	1.2% (0.6% - 2.0%)	11.4% (7.8% - 14.9%)
South Korea	$5 114 717 ($2 118 649 - $10 088 032)	$8576.88 ($3534.54 - $16344.59)	$95.78 ($39.68 - $188.92)	$623.69 ($258.35 - $1230.13)	4.0% (1.6% - 7.8%)	12.9% (9.0% - 16.8%)
High-income North America	$126 691 992 ($102 678 264 - $154 658 508)	$23378.35 ($18462.63 - $28969.21)	$347.53 ($281.65 - $424.24)	$1972.35 ($1598.50 - $2407.73)	3.4% (2.7% - 4.1%)	2.9% (1.6% - 4.1%)
Canada	$4 258 078 ($1 821 937 - $8 517 807)	$8005.51 ($3462.17 - $15399.01)	$116.60 ($49.89 - $233.24)	$612.36 ($262.01 - $1224.95)	2.3% (1.0% - 4.6%)	5.5% (1.7% - 9.1%)
Greenland	$4046 ($1926 - $7181)	$11013.30 ($5166.71 - $20069.99)	$72.01 ($34.28 - $127.79)	$676.46 ($322.03 - $1200.42)	1.1% (0.5% - 1.9%)	7.1% (3.3% - 10.8%)
United States	$122 429 867 ($99 041 778 - $150 811 724)	$25047.24 ($19493.21 - $31295.44)	$373.29 ($301.98 - $459.82)	$2137.60 ($1729.25 - $2633.14)	3.4% (2.7% - 4.2%)	2.8% (1.6% - 4.0%)
Southern Latin America	$825 283 ($446 774 - $1 338 563)	$1421.09 ($770.35 - $2308.85)	$12.36 ($6.69 - $20.05)	$95.19 ($51.53 - $154.40)	1.2% (0.7% - 2.0%)	5.2% (2.7% - 7.6%)

Argentina	$507 748 ($249 951 - $918 657)	$1344.97 ($666.22 - $2420.65)	$11.25 ($5.54 - $20.36)	$88.55 ($43.59 - $160.21)	1.4% (0.7% - 2.6%)	4.2% (0.9% - 7.6%)
Chile	$249 587 ($114 726 - $453 374)	$1567.84 ($706.04 - $2919.77)	$13.71 ($6.30 - $24.91)	$105.19 ($48.35 - $191.08)	1.0% (0.4% - 1.8%)	8.5% (4.9% - 12.3%)
Uruguay	$67 946 ($32 374 - $120 940)	$1541.88 ($762.74 - $2761.84)	$19.77 ($9.42 - $35.20)	$120.74 ($57.53 - $214.91)	1.2% (0.6% - 2.1%)	4.1% (0.4% - 7.7%)
Western Europe	$53 324 015 ($30 910 838 - $79 670 673)	$7307.24 ($4307.48 - $10742.27)	$122.34 ($70.92 - $182.79)	$581.32 ($336.98 - $868.54)	2.8% (1.6% - 4.3%)	4.4% (3.1% - 5.8%)
Andorra	$6565 ($2969 - $12 305)	$6675.63 ($3016.73 - $12502.36)	$79.03 ($35.75 - $148.14)	$513.84 ($232.43 - $963.18)	1.7% (0.8% - 3.3%)	5.2% (1.5% - 8.9%)
Austria	$1 105 342 ($514 415 - $1 998 161)	$8157.88 ($3701.63 - $15060.94)	$123.97 ($57.69 - $224.10)	$624.91 ($290.83 - $1129.68)	2.4% (1.1% - 4.3%)	4.4% (0.8% - 8.2%)
Belgium	$1 377 333 ($646 909 - $2 518 848)	$7717.17 ($3799.46 - $13778.08)	$120.62 ($56.65 - $220.58)	$599.81 ($281.72 - $1096.92)	2.5% (1.2% - 4.6%)	4.7% (1.3% - 8.0%)
Cyprus	$38 590 ($17 288 - $71 730)	$3383.60 ($1564.27 - $6159.36)	$29.38 ($13.16 - $54.61)	$193.61 ($86.74 - $359.87)	2.4% (1.1% - 4.4%)	6.7% (3.2% - 10.3%)
Denmark	$769 744 ($345 586 - $1 396 767)	$9972.88 ($4650.21 - $18027.81)	$132.65 ($59.56 - $240.71)	$639.61 ($287.16 - $1160.62)	2.1% (1.0% - 3.9%)	4.1% (0.6% - 7.6%)
Finland	$754 293 ($362 765 - $1 297 329)	$8218.89 ($3839.85 - $13826.77)	$136.30 ($65.55 - $234.42)	$584.75 ($281.23 - $1005.73)	3.0% (1.5% - 5.2%)	4.7% (1.4% - 8.0%)
France	$8 053 147 ($3 967 111 - $14 279 271)	$7174.29 ($3542.49 - $12611.18)	$121.64 ($59.92 - $215.68)	$574.65 ($283.08 - $1018.93)	2.6% (1.3% - 4.6%)	5.1% (1.9% - 8.7%)
Germany	$12 433 135 ($5 604 078 - $23 535 655)	$7868.64 ($3572.30 - $14694.97)	$146.42 ($66.00 - $277.17)	$657.39 ($296.31 - $1244.42)	2.7% (1.2% - 5.2%)	5.1% (1.7% - 8.8%)
Greece	$664 129 ($307 014 - $1 183 414)	$3431.97 ($1599.08 - $6169.31)	$64.25 ($29.70 - $114.48)	$279.82 ($129.36 - $498.61)	3.9% (1.8% - 7.0%)	4.4% (1.0% - 7.9%)
Iceland	$35 492 ($15 100 - $67 417)	$8768.14 ($3754.46 - $16347.59)	$102.91 ($43.79 - $195.48)	$630.95 ($268.44 - $1198.47)	1.9% (0.8% - 3.5%)	5.3% (1.7% - 9.0%)
Ireland	$516 947 ($219 201 - $955 566)	$10476.11 ($4403.16 - $19721.15)	$105.28 ($44.64 - $194.60)	$675.00 ($286.22 - $1247.73)	1.9% (0.8% - 3.6%)	7.9% (4.1% - 11.7%)
Israel	$429 674 ($199 479 - $763 896)	$5426.06 ($2470.08 - $9519.55)	$46.15 ($21.43 - $82.05)	$327.82 ($152.19 - $582.82)	1.5% (0.7% - 2.7%)	5.2% (1.4% - 8.9%)
Italy	$7 323 412 ($3 643 083 - $12 775 616)	$5356.52 ($2722.96 - $9140.73)	$121.42 ($60.40 - $211.82)	$514.65 ($256.02 - $897.80)	4.1% (2.1% - 7.2%)	4.2% (0.7% - 7.7%)
Luxembourg	$62 646 ($27 635 - $116 755)	$9912.93 ($4396.92 - $17979.91)	$101.28 ($44.68 - $188.76)	$647.62 ($285.69 - $1206.99)	1.6% (0.7% - 3.1%)	4.7% (1.1% - 8.7%)
Malta	$34 480 ($16 485 - $64 106)	$5287.25 ($2555.48 - $9730.24)	$78.50 ($37.53 - $145.96)	$350.61 ($167.63 - $651.87)	2.5% (1.2% - 4.6%)	8.3% (4.8% - 11.7%)
Netherlands	$2 737 096 ($1 357 830 - $4 940 496)	$10620.84 ($5243.57 - $18805.90)	$159.53 ($79.14 - $287.96)	$782.21 ($388.04 - $1411.89)	3.0% (1.5% - 5.4%)	5.5% (2.0% - 8.9%)
Norway	$755 291 ($352 559 - $1 392 944)	$10648.52 ($4950.07 - $19046.44)	$141.21 ($65.91 - $260.42)	$768.86 ($358.89 - $1417.97)	1.7% (0.8% - 3.2%)	4.1% (0.9% - 7.7%)
Portugal	$583 137 ($292 273 - $1 026 876)	$3071.53 ($1587.89 - $5430.18)	$54.75 ($27.44 - $96.41)	$240.36 ($120.47 - $423.26)	2.8% (1.4% - 5.1%)	4.4% (1.0% - 8.0%)
Spain	$3 852 043 ($1 810 118 - $7 124 551)	$5066.79 ($2397.04 - $9221.27)	$83.70 ($39.33 - $154.81)	$411.09 ($193.17 - $760.32)	3.1% (1.4% - 5.7%)	5.3% (1.8% - 8.9%)

Sweden	$1 502 037 ($659 536 - $2 713 308)	$10278.31 ($4375.01 - $18488.44)	$146.93 ($64.52 - $265.42)	$684.25 ($300.45 - $1236.04)	2.5% (1.1% - 4.6%)	3.9% (0.5% - 7.4%)
Switzerland	$1 081 263 ($498 540 - $1 908 686)	$7971.70 ($3827.72 - $14322.67)	$123.22 ($56.81 - $217.51)	$628.00 ($289.55 - $1108.56)	1.2% (0.6% - 2.1%)	3.4% (0.1% - 6.9%)
United Kingdom	$9 208 211 ($4 224 835 - $16 941 034)	$11055.28 ($5177.04 - $19984.94)	$136.99 ($62.85 - $252.02)	$713.52 ($327.37 - $1312.71)	3.5% (1.6% - 6.4%)	3.1% (-0.5% - 6.6%)
Andean Latin America	$258 057 ($141 812 - $412 091)	$876.60 ($486.35 - $1392.83)	$4.06 ($2.23 - $6.48)	$42.57 ($23.40 - $67.98)	1.1% (0.6% - 1.8%)	8.2% (5.8% - 10.8%)
Bolivia	$22 420 ($11 146 - $40 321)	$579.78 ($293.71 - $1031.07)	$1.87 ($0.93 - $3.36)	$21.47 ($10.67 - $38.61)	0.8% (0.4% - 1.5%)	7.7% (4.0% - 11.4%)
Ecuador	$80 627 ($39 549 - $139 331)	$1062.77 ($524.53 - $1821.40)	$4.58 ($2.25 - $7.92)	$49.34 ($24.20 - $85.26)	0.9% (0.4% - 1.6%)	8.6% (5.0% - 12.5%)
Peru	$155 010 ($70 381 - $286 260)	$862.00 ($406.34 - $1547.65)	$4.56 ($2.07 - $8.42)	$45.82 ($20.80 - $84.61)	1.3% (0.6% - 2.4%)	8.1% (4.4% - 11.7%)
Caribbean	$444 453 ($248 637 - $682 664)	$1668.80 ($934.11 - $2548.08)	$9.77 ($5.46 - $15.00)	$85.41 ($47.78 - $131.18)	1.5% (0.8% - 2.3%)	7.1% (5.0% - 9.0%)
Antigua and Barbuda	$907 ($424 - $1624)	$1928.80 ($897.26 - $3393.55)	$10.24 ($4.79 - $18.35)	$97.12 ($45.38 - $173.99)	1.4% (0.7% - 2.5%)	4.2% (0.6% - 7.9%)
The Bahamas	$6299 ($2969 - $11 626)	$3649.20 ($1747.25 - $6727.56)	$16.71 ($7.88 - $30.85)	$185.02 ($87.21 - $341.52)	0.8% (0.4% - 1.6%)	6.1% (2.6% - 9.8%)
Barbados	$5768 ($2724 - $10 496)	$2134.93 ($991.09 - $3733.96)	$19.37 ($9.15 - $35.25)	$118.55 ($55.98 - $215.72)	1.8% (0.8% - 3.2%)	3.4% (-0.0% - 7.1%)
Belize	$861 ($408 - $1561)	$699.68 ($328.54 - $1248.60)	$2.10 ($0.99 - $3.81)	$30.76 ($14.56 - $55.75)	0.8% (0.4% - 1.4%)	6.8% (3.4% - 10.6%)
Bermuda	$6520 ($3155 - $11 607)	$7994.24 ($3827.14 - $14194.61)	$101.83 ($49.28 - $181.28)	$509.74 ($246.66 - $907.47)	1.4% (0.7% - 2.6%)	7.4% (3.6% - 11.1%)
Cuba	$203 600 ($97 077 - $363 971)	$1890.89 ($917.45 - $3305.97)	$17.92 ($8.55 - $32.04)	$110.31 ($52.60 - $197.20)	1.4% (0.7% - 2.6%)	8.3% (4.7% - 11.8%)
Dominica	$575 ($273 - $1021)	$1181.65 ($563.53 - $2037.23)	$8.37 ($3.98 - $14.86)	$64.84 ($30.82 - $115.11)	1.6% (0.8% - 2.9%)	2.1% (-1.3% - 5.7%)
Dominican Republic	$39 769 ($19 871 - $73 315)	$909.67 ($462.41 - $1628.61)	$3.65 ($1.83 - $6.74)	$39.94 ($19.95 - $73.62)	0.8% (0.4% - 1.4%)	7.2% (3.9% - 10.8%)
Grenada	$572 ($274 - $1043)	$1166.25 ($570.47 - $2102.83)	$5.54 ($2.66 - $10.11)	$54.32 ($26.05 - $99.11)	1.0% (0.5% - 1.8%)	3.3% (-0.1% - 6.9%)
Guyana	$1878 ($939 - $3425)	$780.42 ($379.64 - $1381.88)	$2.44 ($1.22 - $4.44)	$30.61 ($15.30 - $55.83)	0.9% (0.4% - 1.5%)	6.9% (3.5% - 10.3%)
Haiti	$4090 ($2109 - $7439)	$170.93 ($89.42 - $306.48)	$0.33 ($0.17 - $0.60)	$4.98 ($2.57 - $9.05)	0.7% (0.4% - 1.3%)	3.9% (0.7% - 7.2%)
Jamaica	$17 067 ($8024 - $30 754)	$961.60 ($453.39 - $1802.61)	$6.07 ($2.85 - $10.94)	$57.69 ($27.12 - $103.96)	1.8% (0.8% - 3.2%)	4.1% (0.7% - 7.5%)
Puerto Rico	$128 993 ($61 684 - $230 351)	$2671.09 ($1245.65 - $4697.46)	$36.63 ($17.52 - $65.41)	$174.59 ($83.49 - $311.78)	2.6% (1.2% - 4.7%)	6.4% (3.1% - 10.1%)
Saint Lucia	$1291 ($619 - $2254)	$1190.85 ($566.97 - $2079.07)	$7.39 ($3.54 - $12.91)	$64.75 ($31.03 - $113.08)	1.3% (0.6% - 2.3%)	5.3% (1.7% - 8.7%)

Saint Vincent and the Grenadines	$542 ($272 - $958)	$840.68 ($415.79 - $1459.03)	$4.79 ($2.40 - $8.47)	$41.62 ($20.85 - $73.57)	1.4% (0.7% - 2.5%)	5.0% (1.3% - 8.9%)
Suriname	$2678 ($1320 - $4630)	$950.60 ($470.74 - $1627.34)	$4.65 ($2.29 - $8.04)	$46.83 ($23.09 - $80.97)	1.0% (0.5% - 1.8%)	7.2% (3.6% - 10.7%)
Trinidad and Tobago	$20 825 ($9439 - $37 309)	$2261.27 ($1047.01 - $3994.32)	$15.01 ($6.80 - $26.89)	$114.38 ($51.84 - $204.91)	1.3% (0.6% - 2.4%)	7.6% (4.2% - 11.4%)
Virgin Islands, U.S.	$2217 ($962 - $4120)	$2444.88 ($1076.52 - $4581.37)	$21.32 ($9.25 - $39.63)	$111.04 ($48.19 - $206.40)	1.3% (0.5% - 2.4%)	6.6% (3.0% - 10.7%)
Central Latin America	$988 873 ($590 570 - $1 510 832)	$820.03 ($481.49 - $1223.88)	$3.96 ($2.36 - $6.04)	$40.86 ($24.41 - $62.43)	0.8% (0.5% - 1.3%)	6.4% (4.3% - 8.6%)
Colombia	$272 851 ($141 455 - $473 620)	$831.53 ($422.74 - $1439.44)	$5.71 ($2.96 - $9.91)	$50.54 ($26.20 - $87.73)	1.1% (0.6% - 2.0%)	8.6% (5.2% - 12.0%)
Costa Rica	$46 686 ($23 411 - $84 628)	$1550.03 ($802.95 - $2750.72)	$9.90 ($4.96 - $17.94)	$92.05 ($46.16 - $166.85)	1.0% (0.5% - 1.8%)	7.9% (4.0% - 11.5%)
El Salvador	$23 169 ($11 264 - $41 058)	$614.21 ($295.48 - $1060.92)	$3.70 ($1.80 - $6.56)	$35.88 ($17.44 - $63.58)	1.1% (0.6% - 2.0%)	4.3% (0.8% - 7.7%)
Guatemala	$29 132 ($14 684 - $51 183)	$538.83 ($274.21 - $952.76)	$1.64 ($0.83 - $2.88)	$21.95 ($11.06 - $38.56)	0.6% (0.3% - 1.1%)	7.7% (4.1% - 11.3%)
Honduras	$10 443 ($5388 - $18 226)	$396.15 ($205.59 - $695.22)	$1.06 ($0.55 - $1.86)	$14.57 ($7.52 - $25.43)	0.6% (0.3% - 1.0%)	6.3% (3.0% - 9.8%)
Mexico	$513 099 ($256 484 - $895 665)	$955.75 ($488.15 - $1651.24)	$4.11 ($2.05 - $7.17)	$43.63 ($21.81 - $76.16)	0.7% (0.4% - 1.3%)	6.4% (3.1% - 10.1%)
Nicaragua	$7711 ($3818 - $13 340)	$378.78 ($186.15 - $645.15)	$1.18 ($0.59 - $2.05)	$16.28 ($8.06 - $28.17)	0.7% (0.3% - 1.2%)	6.9% (3.1% - 10.4%)
Panama	$40 550 ($20 210 - $74 207)	$1630.77 ($812.61 - $2873.37)	$9.75 ($4.86 - $17.84)	$92.45 ($46.08 - $169.18)	0.8% (0.4% - 1.5%)	7.9% (4.3% - 11.6%)
Venezuela	$45 228 ($22 642 - $81 228)	$306.90 ($151.39 - $541.85)	$1.61 ($0.81 - $2.89)	$15.44 ($7.73 - $27.72)	1.8% (0.9% - 3.4%)	-0.3% (-3.9% - 3.1%)
Tropical Latin America	$2 048 329 ($1 026 526 - $3 551 529)	$1179.86 ($615.19 - $1998.29)	$9.16 ($4.59 - $15.88)	$85.63 ($42.91 - $148.47)	1.1% (0.6% - 2.0%)	6.6% (3.1% - 10.1%)
Brazil	$2 017 500 ($999 063 - $3 510 192)	$1186.54 ($611.94 - $2016.79)	$9.31 ($4.61 - $16.20)	$86.44 ($42.80 - $150.39)	1.1% (0.6% - 2.0%)	6.6% (3.1% - 10.1%)
Paraguay	$30 828 ($14 914 - $55 742)	$862.12 ($413.22 - $1566.49)	$4.45 ($2.15 - $8.04)	$53.11 ($25.69 - $96.03)	1.1% (0.5% - 2.0%)	7.2% (3.6% - 11.0%)
North Africa and Middle East	$2 100 841 ($1 302 812 - $3 065 548)	$847.91 ($518.21 - $1213.33)	$3.45 ($2.14 - $5.04)	$47.88 ($29.69 - $69.87)	1.0% (0.6% - 1.4%)	8.2% (6.5% - 9.9%)
Afghanistan	$8777 ($4418 - $15 617)	$160.71 ($81.91 - $279.71)	$0.23 ($0.12 - $0.41)	$3.99 ($2.01 - $7.11)	0.5% (0.2% - 0.9%)	5.9% (2.2% - 9.4%)
Algeria	$124 148 ($60 231 - $215 764)	$643.37 ($319.91 - $1108.28)	$2.97 ($1.44 - $5.16)	$35.56 ($17.25 - $61.81)	1.1% (0.5% - 2.0%)	9.2% (5.6% - 13.0%)
Bahrain	$7207 ($3520 - $12 543)	$1921.12 ($971.95 - $3299.70)	$5.00 ($2.44 - $8.69)	$112.10 ($54.75 - $195.11)	0.4% (0.2% - 0.7%)	9.8% (6.2% - 13.7%)
Egypt	$161 901 ($81 442 - $291 019)	$548.30 ($272.16 - $964.37)	$1.63 ($0.82 - $2.94)	$24.34 ($12.24 - $43.75)	1.1% (0.5% - 2.0%)	6.3% (2.6% - 9.8%)
Iran	$562 704 ($279 470 - $985 802)	$1197.38 ($593.48 - $2080.49)	$6.68 ($3.32 - $11.69)	$81.51 ($40.48 - $142.80)	1.4% (0.7% - 2.5%)	9.9% (6.1% - 13.8%)

Iraq	$68 010 ($34 580 - $118 273)	$573.96 ($284.33 - $1012.52)	$1.61 ($0.82 - $2.81)	$26.53 ($13.49 - $46.14)	0.8% (0.4% - 1.4%)	8.8% (5.1% - 12.7%)
Jordan	$28 063 ($13 882 - $49 493)	$878.54 ($432.25 - $1528.64)	$2.41 ($1.19 - $4.25)	$40.92 ($20.24 - $72.17)	0.8% (0.4% - 1.5%)	8.4% (4.8% - 12.3%)
Kuwait	$40 066 ($19 370 - $69 598)	$2808.47 ($1361.63 - $4837.97)	$9.05 ($4.38 - $15.72)	$194.19 ($93.88 - $337.32)	0.6% (0.3% - 1.0%)	9.0% (5.4% - 12.6%)
Lebanon	$42 498 ($20 396 - $74 473)	$1064.76 ($509.22 - $1837.39)	$8.21 ($3.94 - $14.39)	$74.00 ($35.51 - $129.67)	0.9% (0.4% - 1.5%)	5.5% (1.9% - 9.0%)
Libya	$41 790 ($20 452 - $75 455)	$1373.65 ($680.31 - $2428.95)	$6.20 ($3.04 - $11.20)	$95.40 ($46.69 - $172.25)	1.3% (0.6% - 2.4%)	4.5% (1.0% - 8.0%)
Morocco	$79 621 ($40 218 - $138 156)	$457.23 ($233.88 - $786.16)	$2.21 ($1.12 - $3.84)	$27.18 ($13.73 - $47.16)	1.2% (0.6% - 2.1%)	7.4% (3.8% - 11.2%)
Oman	$9984 ($4914 - $18 095)	$1482.01 ($733.48 - $2648.48)	$2.18 ($1.07 - $3.95)	$57.83 ($28.47 - $104.82)	0.4% (0.2% - 0.6%)	6.0% (2.5% - 9.5%)
Palestine	$9356 ($4937 - $16 930)	$778.35 ($401.98 - $1381.50)	$1.89 ($1.00 - $3.42)	$32.89 ($17.36 - $59.52)	0.5% (0.3% - 1.0%)	6.7% (3.4% - 10.3%)
Qatar	$8661 ($4289 - $15 037)	$3218.70 ($1605.56 - $5477.11)	$3.02 ($1.50 - $5.25)	$133.72 ($66.23 - $232.17)	0.2% (0.1% - 0.3%)	12.0% (8.4% - 15.6%)
Saudi Arabia	$169 825 ($83 257 - $311 313)	$2504.29 ($1251.69 - $4542.81)	$4.75 ($2.33 - $8.71)	$123.34 ($60.47 - $226.10)	0.4% (0.2% - 0.6%)	7.5% (3.9% - 11.3%)
Sudan	$19 045 ($9265 - $33 440)	$189.78 ($92.73 - $329.86)	$0.47 ($0.23 - $0.82)	$7.69 ($3.74 - $13.50)	0.9% (0.4% - 1.6%)	5.2% (1.5% - 9.1%)
Syria	$14 057 ($7087 - $24 453)	$221.41 ($108.97 - $381.14)	$0.97 ($0.49 - $1.69)	$12.19 ($6.15 - $21.21)	1.6% (0.8% - 2.9%)	4.3% (0.7% - 7.9%)
Tunisia	$60 058 ($29 716 - $107 566)	$710.68 ($361.33 - $1239.26)	$5.19 ($2.57 - $9.30)	$49.44 ($24.46 - $88.55)	2.0% (1.0% - 3.8%)	8.5% (4.9% - 11.9%)
Turkey	$605 103 ($291 683 - $1 109 100)	$943.07 ($457.58 - $1706.89)	$7.44 ($3.59 - $13.63)	$71.99 ($34.70 - $131.95)	1.9% (0.9% - 3.4%)	8.4% (4.6% - 12.1%)
United Arab Emirates	$30 779 ($14 701 - $54 761)	$3819.62 ($1832.91 - $6758.72)	$3.33 ($1.59 - $5.93)	$181.23 ($86.56 - $322.44)	0.2% (0.1% - 0.4%)	11.3% (7.4% - 15.2%)
Yemen	$9183 ($4807 - $16 144)	$142.09 ($73.09 - $247.27)	$0.29 ($0.15 - $0.51)	$4.96 ($2.59 - $8.71)	0.6% (0.3% - 1.0%)	3.1% (-0.3% - 6.8%)
South Asia	$1 013 502 ($543 088 - $1 735 193)	$218.70 ($119.36 - $360.02)	$0.56 ($0.30 - $0.96)	$6.94 ($3.72 - $11.88)	0.8% (0.4% - 1.5%)	9.5% (6.1% - 12.5%)
Bangladesh	$83 065 ($41 001 - $144 475)	$154.43 ($75.47 - $270.02)	$0.52 ($0.26 - $0.91)	$6.16 ($3.04 - $10.72)	1.2% (0.5% - 2.1%)	10.6% (6.9% - 14.3%)
Bhutan	$446 ($210 - $787)	$207.06 ($98.49 - $358.82)	$0.59 ($0.28 - $1.04)	$7.56 ($3.56 - $13.34)	0.6% (0.3% - 1.1%)	6.1% (2.5% - 9.7%)
India	$874 923 ($440 300 - $1 558 431)	$236.99 ($120.82 - $408.21)	$0.63 ($0.32 - $1.12)	$7.54 ($3.79 - $13.43)	0.8% (0.4% - 1.6%)	9.8% (5.8% - 13.4%)
Nepal	$11 745 ($6018 - $20 696)	$149.95 ($75.89 - $262.22)	$0.39 ($0.20 - $0.68)	$4.85 ($2.48 - $8.54)	0.7% (0.3% - 1.3%)	8.5% (4.6% - 12.3%)
Pakistan	$43 322 ($21 347 - $76 978)	$133.91 ($66.32 - $230.24)	$0.19 ($0.10 - $0.34)	$3.09 ($1.52 - $5.50)	0.5% (0.2% - 0.9%)	5.3% (1.8% - 8.9%)
East Asia	$20 172 174 ($10 113 689 - $34 436 808)	$1490.51 ($763.14 - $2502.93)	$13.70 ($6.87 - $23.39)	$100.76 ($50.52 - $172.02)	2.5% (1.3% - 4.3%)	11.7% (8.4% - 15.0%)
China	$18 828 741 ($9 074 270 - $32 736 602)	$1436.40 ($702.14 - $2442.71)	$13.24 ($6.38 - $23.02)	$97.35 ($46.92 - $169.25)	2.5% (1.2% - 4.3%)	12.0% (8.4% - 15.8%)

North Korea	$55 408 ($26 793 - $102 285)	$321.67 ($163.67 - $577.48)	$2.11 ($1.02 - $3.90)	$18.58 ($8.98 - $34.29)	2.6% (1.2% - 4.9%)	4.7% (1.2% - 8.1%)
Taiwan	$1 288 024 ($592 884 - $2 302 895)	$5060.11 ($2270.88 - $9237.48)	$54.53 ($25.10 - $97.50)	$339.38 ($156.22 - $606.80)	3.6% (1.6% - 6.4%)	8.8% (5.4% - 12.6%)
Oceania	$11 360 ($6807 - $16 646)	$511.46 ($308.95 - $749.92)	$0.90 ($0.54 - $1.32)	$14.20 ($8.51 - $20.80)	0.8% (0.5% - 1.3%)	4.5% (2.8% - 6.1%)
American Samoa	$344 ($178 - $596)	$1604.06 ($834.09 - $2781.40)	$6.21 ($3.20 - $10.73)	$73.58 ($37.92 - $127.28)	0.9% (0.4% - 1.5%)	2.9% (-0.3% - 6.4%)
Federated States of Micronesia	$127 ($65 - $220)	$555.33 ($289.25 - $953.28)	$1.24 ($0.63 - $2.16)	$20.45 ($10.43 - $35.57)	0.8% (0.4% - 1.4%)	3.1% (-0.4% - 6.3%)
Fiji	$2067 ($1040 - $3671)	$760.09 ($392.95 - $1352.10)	$2.27 ($1.14 - $4.03)	$29.18 ($14.68 - $51.83)	1.1% (0.5% - 2.0%)	5.1% (1.6% - 8.7%)
Guam	$2940 ($1397 - $5255)	$2712.56 ($1288.92 - $4822.33)	$17.23 ($8.19 - $30.80)	$151.49 ($71.97 - $270.72)	1.2% (0.6% - 2.3%)	6.2% (2.8% - 9.7%)
Kiribati	$127 ($64 - $225)	$624.17 ($318.74 - $1096.49)	$1.07 ($0.54 - $1.90)	$18.03 ($9.06 - $31.83)	0.4% (0.2% - 0.8%)	2.4% (-0.9% - 5.9%)
Marshall Islands	$111 ($57 - $193)	$1161.08 ($609.31 - $1906.35)	$1.96 ($1.01 - $3.40)	$32.70 ($16.90 - $56.83)	0.4% (0.2% - 0.8%)	3.9% (0.8% - 7.1%)
Northern Mariana Islands	$334 ($167 - $585)	$1755.09 ($885.72 - $3018.87)	$7.87 ($3.93 - $13.77)	$84.60 ($42.30 - $148.07)	1.0% (0.5% - 1.8%)	5.9% (2.6% - 9.6%)
Papua New Guinea	$3869 ($1929 - $6982)	$260.84 ($131.48 - $461.02)	$0.39 ($0.20 - $0.71)	$6.43 ($3.21 - $11.60)	0.6% (0.3% - 1.2%)	3.9% (0.6% - 7.4%)
Samoa	$542 ($260 - $1010)	$796.54 ($395.69 - $1464.70)	$2.56 ($1.23 - $4.78)	$37.47 ($17.96 - $69.81)	1.1% (0.5% - 2.1%)	3.4% (-0.1% - 6.8%)
Solomon Islands	$318 ($153 - $574)	$352.22 ($172.74 - $630.00)	$0.48 ($0.23 - $0.88)	$8.16 ($3.94 - $14.72)	0.4% (0.2% - 0.8%)	2.6% (-0.7% - 5.9%)
Tonga	$347 ($178 - $640)	$780.44 ($398.34 - $1375.43)	$3.39 ($1.74 - $6.26)	$38.72 ($19.91 - $71.55)	1.4% (0.7% - 2.6%)	3.2% (-0.2% - 6.4%)
Vanuatu	$234 ($118 - $412)	$370.91 ($191.33 - $657.73)	$0.79 ($0.40 - $1.40)	$11.33 ($5.72 - $19.98)	0.8% (0.4% - 1.5%)	4.5% (1.0% - 7.9%)
Southeast Asia	$1 913 997 ($1 144 994 - $2 826 537)	$687.17 ($411.90 - $1010.28)	$2.84 ($1.70 - $4.20)	$33.74 ($20.18 - $49.82)	1.8% (1.0% - 2.6%)	8.4% (6.8% - 10.1%)
Cambodia	$16 463 ($8471 - $29 767)	$318.80 ($163.48 - $559.35)	$0.99 ($0.51 - $1.79)	$13.22 ($6.80 - $23.91)	1.1% (0.5% - 1.9%)	8.0% (4.5% - 12.1%)
Indonesia	$480 460 ($225 143 - $859 981)	$575.02 ($278.39 - $1032.17)	$1.85 ($0.87 - $3.31)	$25.05 ($11.74 - $44.84)	1.4% (0.7% - 2.6%)	7.7% (4.1% - 11.4%)
Laos	$4705 ($2214 - $8774)	$271.84 ($132.90 - $474.85)	$0.66 ($0.31 - $1.23)	$9.97 ($4.69 - $18.59)	1.1% (0.5% - 2.0%)	5.4% (2.0% - 8.9%)
Malaysia	$181 419 ($85 900 - $326 804)	$1413.93 ($670.70 - $2498.97)	$5.80 ($2.74 - $10.44)	$67.65 ($32.03 - $121.86)	1.3% (0.6% - 2.3%)	8.7% (5.2% - 12.3%)
Maldives	$2654 ($1348 - $4799)	$1856.36 ($951.24 - $3219.31)	$5.32 ($2.70 - $9.63)	$94.63 ($48.06 - $171.13)	0.5% (0.2% - 0.8%)	8.8% (5.4% - 12.2%)
Mauritius	$15 556 ($7661 - $27 048)	$1649.64 ($814.69 - $2844.61)	$12.19 ($6.00 - $21.19)	$94.92 ($46.74 - $165.03)	1.8% (0.9% - 3.2%)	9.4% (5.8% - 13.2%)
Myanmar	$58 639 ($28 891 - $108 053)	$280.39 ($139.24 - $515.67)	$1.07 ($0.53 - $1.98)	$12.90 ($6.36 - $23.77)	1.9% (0.9% - 3.5%)	12.2% (8.2% - 16.0%)

Philippines	$191 308 ($91 977 - $330 891)	$586.77 ($289.62 - $1002.49)	$1.71 ($0.82 - $2.95)	$23.01 ($11.06 - $39.80)	1.2% (0.6% - 2.1%)	7.6% (4.2% - 11.3%)
Sri Lanka	$86 973 ($41 207 - $156 843)	$662.70 ($328.35 - $1174.68)	$3.98 ($1.89 - $7.18)	$34.09 ($16.15 - $61.48)	2.5% (1.2% - 4.8%)	6.9% (3.3% - 10.4%)
Seychelles	$964 ($477 - $1630)	$1759.35 ($886.48 - $2963.11)	$9.44 ($4.67 - $15.96)	$98.80 ($48.83 - $167.00)	1.1% (0.6% - 2.0%)	3.6% (0.3% - 6.9%)
Thailand	$598 789 ($278 087 - $1 062 475)	$975.76 ($470.28 - $1702.66)	$8.54 ($3.97 - $15.15)	$64.17 ($29.80 - $113.85)	2.9% (1.4% - 5.3%)	9.0% (5.5% - 12.5%)
Timor-Leste	$1120 ($560 - $1999)	$327.42 ($165.37 - $566.14)	$0.84 ($0.42 - $1.50)	$10.39 ($5.20 - $18.55)	1.0% (0.5% - 1.8%)	8.6% (5.1% - 12.3%)
Vietnam	$274 943 ($132 661 - $489 392)	$601.08 ($299.63 - $1074.57)	$2.85 ($1.38 - $5.08)	$33.95 ($16.38 - $60.42)	2.0% (0.9% - 3.5%)	9.0% (5.3% - 12.8%)
Central Sub-Saharan Africa	$33 885 ($19 788 - $51 511)	$153.56 ($90.31 - $230.50)	$0.26 ($0.15 - $0.39)	$4.40 ($2.57 - $6.69)	0.6% (0.4% - 1.0%)	6.4% (4.3% - 8.3%)
Angola	$9845 ($4803 - $17 089)	$229.73 ($115.16 - $393.40)	$0.33 ($0.16 - $0.57)	$5.59 ($2.73 - $9.70)	0.4% (0.2% - 0.7%)	6.7% (3.1% - 10.3%)
Central African Republic	$742 ($375 - $1276)	$93.74 ($47.69 - $163.01)	$0.14 ($0.07 - $0.24)	$2.41 ($1.22 - $4.14)	0.7% (0.3% - 1.2%)	2.5% (-0.8% - 6.2%)
Congo	$2639 ($1297 - $4834)	$238.42 ($115.48 - $429.32)	$0.50 ($0.25 - $0.92)	$8.51 ($4.18 - $15.59)	0.9% (0.4% - 1.7%)	5.6% (1.9% - 9.4%)
Democratic Republic of the Congo	$14 824 ($7424 - $26 128)	$97.82 ($47.82 - $172.13)	$0.17 ($0.08 - $0.30)	$2.89 ($1.45 - $5.09)	0.9% (0.4% - 1.6%)	7.2% (3.8% - 10.8%)
Equatorial Guinea	$1774 ($815 - $3267)	$808.02 ($384.29 - $1551.63)	$1.25 ($0.57 - $2.30)	$25.10 ($11.53 - $46.24)	0.5% (0.2% - 0.9%)	8.9% (4.9% - 12.8%)
Gabon	$4062 ($1935 - $7334)	$799.04 ($375.48 - $1452.84)	$2.32 ($1.11 - $4.19)	$35.12 ($16.73 - $63.41)	0.8% (0.4% - 1.5%)	4.1% (0.6% - 7.8%)
Eastern Sub-Saharan Africa	$98 373 ($60 035 - $141 343)	$159.07 ($95.77 - $226.91)	$0.24 ($0.15 - $0.34)	$3.96 ($2.42 - $5.69)	0.6% (0.3% - 0.8%)	6.4% (4.7% - 7.9%)
Burundi	$1585 ($754 - $3005)	$100.98 ($49.25 - $184.70)	$0.13 ($0.06 - $0.25)	$2.14 ($1.02 - $4.05)	0.5% (0.2% - 0.9%)	4.2% (0.6% - 7.9%)
Comoros	$558 ($268 - $996)	$251.44 ($119.16 - $444.50)	$0.78 ($0.37 - $1.39)	$10.25 ($4.91 - $18.28)	1.0% (0.5% - 1.9%)	3.9% (0.4% - 7.5%)
Djibouti	$389 ($197 - $687)	$204.31 ($105.62 - $350.52)	$0.32 ($0.16 - $0.57)	$5.47 ($2.78 - $9.66)	0.6% (0.3% - 1.0%)	6.0% (2.5% - 9.7%)
Eritrea	$704 ($344 - $1245)	$82.90 ($42.26 - $146.26)	$0.10 ($0.05 - $0.19)	$2.00 ($0.98 - $3.54)	0.4% (0.2% - 0.7%)	3.2% (-0.1% - 6.7%)
Ethiopia	$18 008 ($9035 - $32 701)	$107.05 ($54.60 - $189.53)	$0.17 ($0.08 - $0.30)	$2.75 ($1.38 - $5.00)	0.5% (0.3% - 1.1%)	7.8% (4.0% - 11.5%)
Kenya	$27 316 ($12 892 - $50 711)	$337.04 ($155.20 - $613.13)	$0.54 ($0.26 - $1.01)	$9.73 ($4.59 - $18.07)	0.6% (0.3% - 1.1%)	6.4% (2.7% - 10.0%)
Madagascar	$3763 ($1901 - $6577)	$108.98 ($55.75 - $185.40)	$0.14 ($0.07 - $0.25)	$2.53 ($1.28 - $4.43)	0.6% (0.3% - 1.1%)	3.7% (0.3% - 7.0%)
Malawi	$4107 ($2056 - $7163)	$141.48 ($71.19 - $242.89)	$0.22 ($0.11 - $0.39)	$3.85 ($1.93 - $6.71)	0.5% (0.3% - 0.9%)	6.6% (3.1% - 10.1%)

Mozambique	$4737 ($2492 - $8558)	$115.96 ($60.68 - $203.62)	$0.16 ($0.08 - $0.29)	$2.58 ($1.36 - $4.66)	0.5% (0.2% - 0.8%)	6.0% (2.5% - 9.6%)
Rwanda	$4369 ($2169 - $7791)	$189.44 ($96.76 - $336.12)	$0.34 ($0.17 - $0.61)	$5.74 ($2.85 - $10.24)	0.7% (0.4% - 1.4%)	10.5% (6.9% - 14.2%)
Somalia	$666 ($335 - $1192)	$32.59 ($16.67 - $56.03)	$0.03 ($0.02 - $0.06)	$0.53 ($0.27 - $0.95)	0.5% (0.2% - 0.9%)	4.5% (1.0% - 8.1%)
South Sudan	$1586 ($815 - $2828)	$110.77 ($56.15 - $194.64)	$0.17 ($0.09 - $0.30)	$2.68 ($1.38 - $4.77)	0.6% (0.3% - 1.0%)	1.5% (-2.0% - 5.0%)
Tanzania	$16 113 ($7892 - $27 636)	$161.72 ($79.05 - $274.35)	$0.28 ($0.14 - $0.49)	$4.28 ($2.10 - $7.34)	0.7% (0.3% - 1.1%)	7.0% (3.5% - 10.6%)
Uganda	$8984 ($4415 - $16 150)	$163.23 ($80.35 - $291.30)	$0.22 ($0.11 - $0.39)	$3.62 ($1.78 - $6.51)	0.5% (0.2% - 0.9%)	6.0% (2.2% - 9.6%)
Zambia	$5487 ($2641 - $9405)	$229.18 ($112.94 - $393.84)	$0.30 ($0.14 - $0.52)	$5.30 ($2.55 - $9.08)	0.5% (0.2% - 0.8%)	5.9% (2.4% - 9.6%)
Southern Sub-Saharan Africa	$277 947 ($137 447 - $468 914)	$1084.71 ($540.61 - $1808.66)	$3.54 ($1.75 - $5.97)	$46.47 ($22.98 - $78.40)	0.8% (0.4% - 1.3%)	5.2% (2.0% - 8.3%)
Botswana	$5670 ($2809 - $10 294)	$1085.29 ($550.41 - $1970.11)	$2.42 ($1.20 - $4.40)	$40.11 ($19.87 - $72.81)	0.5% (0.2% - 0.9%)	6.9% (3.6% - 10.5%)
Lesotho	$1811 ($863 - $3298)	$374.45 ($181.82 - $696.09)	$0.87 ($0.41 - $1.58)	$12.89 ($6.14 - $23.47)	0.7% (0.3% - 1.2%)	4.7% (1.4% - 8.1%)
Namibia	$6309 ($3211 - $11 193)	$964.17 ($508.26 - $1671.26)	$2.63 ($1.34 - $4.66)	$37.11 ($18.89 - $65.85)	0.5% (0.3% - 0.9%)	4.4% (1.0% - 8.0%)
South Africa	$256 458 ($120 478 - $443 076)	$1213.24 ($583.02 - $2061.09)	$4.61 ($2.17 - $7.97)	$56.79 ($26.68 - $98.12)	0.8% (0.4% - 1.4%)	5.3% (1.8% - 8.8%)
Swaziland	$1377 ($633 - $2446)	$632.80 ($294.39 - $1104.88)	$1.21 ($0.55 - $2.14)	$19.17 ($8.82 - $34.06)	0.4% (0.2% - 0.8%)	5.8% (2.3% - 9.6%)
Zimbabwe	$6323 ($3011 - $11 392)	$242.36 ($114.16 - $435.15)	$0.42 ($0.20 - $0.76)	$6.72 ($3.20 - $12.10)	0.6% (0.3% - 1.2%)	1.2% (-2.0% - 4.7%)
Western Sub-Saharan Africa	$141 645 ($81 265 - $215 199)	$234.10 ($135.13 - $354.84)	$0.31 ($0.18 - $0.47)	$4.96 ($2.85 - $7.54)	0.5% (0.3% - 0.7%)	6.1% (3.9% - 8.1%)
Benin	$1941 ($1020 - $3277)	$118.81 ($61.01 - $202.44)	$0.15 ($0.08 - $0.26)	$2.35 ($1.24 - $3.97)	0.4% (0.2% - 0.8%)	3.9% (0.5% - 7.3%)
Burkina Faso	$4035 ($2044 - $6825)	$127.00 ($64.14 - $217.81)	$0.18 ($0.09 - $0.30)	$2.64 ($1.34 - $4.46)	0.4% (0.2% - 0.8%)	7.1% (3.6% - 10.7%)
Cameroon	$8144 ($4071 - $14 661)	$214.35 ($107.87 - $375.16)	$0.28 ($0.14 - $0.50)	$4.84 ($2.42 - $8.71)	0.5% (0.2% - 0.9%)	5.4% (2.0% - 8.9%)
Cape Verde	$1050 ($527 - $1841)	$482.23 ($238.77 - $843.45)	$1.86 ($0.93 - $3.27)	$24.51 ($12.29 - $42.98)	1.0% (0.5% - 1.8%)	5.1% (1.3% - 8.6%)
Chad	$1800 ($908 - $3140)	$96.98 ($49.35 - $171.18)	$0.11 ($0.06 - $0.19)	$1.58 ($0.80 - $2.75)	0.4% (0.2% - 0.7%)	2.8% (-0.6% - 6.6%)
Cote d'Ivoire	$7276 ($3745 - $12 636)	$230.03 ($121.21 - $396.38)	$0.28 ($0.14 - $0.48)	$4.75 ($2.45 - $8.26)	0.4% (0.2% - 0.6%)	4.9% (1.6% - 8.5%)
The Gambia	$541 ($275 - $950)	$152.08 ($77.27 - $266.97)	$0.24 ($0.12 - $0.42)	$3.83 ($1.95 - $6.72)	0.6% (0.3% - 1.0%)	7.5% (4.1% - 11.1%)
Ghana	$16 426 ($8379 - $29 299)	$308.67 ($156.72 - $549.91)	$0.52 ($0.27 - $0.93)	$8.31 ($4.24 - $14.83)	0.7% (0.3% - 1.2%)	8.0% (4.5% - 11.8%)
Guinea	$3277 ($1665 - $5726)	$158.65 ($80.68 - $267.39)	$0.26 ($0.13 - $0.45)	$3.74 ($1.90 - $6.53)	0.5% (0.3% - 1.0%)	4.0% (0.7% - 7.6%)

Guinea-Bissau	$383 ($188 - $641)	$178.74 ($87.90 - $304.49)	$0.20 ($0.10 - $0.34)	$3.55 ($1.74 - $5.95)	0.3% (0.2% - 0.6%)	4.4% (1.0% - 7.9%)
Liberia	$1303 ($635 - $2276)	$191.52 ($96.21 - $334.88)	$0.27 ($0.13 - $0.48)	$4.86 ($2.37 - $8.50)	0.4% (0.2% - 0.7%)	8.3% (4.4% - 11.7%)
Mali	$3130 ($1530 - $5505)	$104.81 ($50.80 - $183.35)	$0.14 ($0.07 - $0.25)	$2.05 ($1.00 - $3.60)	0.4% (0.2% - 0.8%)	3.0% (-0.4% - 6.7%)
Mauritania	$1966 ($996 - $3486)	$247.74 ($126.34 - $429.61)	$0.49 ($0.25 - $0.87)	$7.34 ($3.72 - $13.01)	0.8% (0.4% - 1.5%)	5.1% (1.5% - 8.7%)
Niger	$2275 ($1123 - $3946)	$92.57 ($44.98 - $160.78)	$0.10 ($0.05 - $0.17)	$1.41 ($0.69 - $2.44)	0.3% (0.2% - 0.6%)	5.5% (2.1% - 9.2%)
Nigeria	$77 787 ($36 310 - $138 051)	$292.14 ($141.07 - $508.31)	$0.36 ($0.17 - $0.64)	$5.99 ($2.80 - $10.64)	0.5% (0.2% - 0.9%)	6.4% (2.8% - 10.1%)
Sao Tome and Principe	$124 ($62 - $223)	$332.28 ($169.76 - $586.27)	$0.60 ($0.30 - $1.09)	$10.42 ($5.25 - $18.77)	0.5% (0.3% - 1.0%)	2.1% (-1.3% - 5.8%)
Senegal	$5835 ($2817 - $10 545)	$212.16 ($101.51 - $376.24)	$0.39 ($0.19 - $0.70)	$5.78 ($2.79 - $10.45)	0.6% (0.3% - 1.0%)	5.7% (2.3% - 9.5%)
Sierra Leone	$2548 ($1290 - $4591)	$203.23 ($100.66 - $357.59)	$0.31 ($0.16 - $0.55)	$4.83 ($2.45 - $8.71)	0.4% (0.2% - 0.8%)	5.3% (1.8% - 9.1%)
Togo	$1806 ($936 - $3114)	$160.71 ($84.50 - $273.67)	$0.23 ($0.12 - $0.39)	$3.81 ($1.97 - $6.57)	0.5% (0.3% - 0.9%)	7.2% (3.7% - 10.9%)

Notes: All spending measured in 2019 US dollars.

Table S9: Attributable dementia spending by GBD regions and countries in 2019 (2019 PPP)

	Spending attributable to dementia, 2019 PPP (Thousand)	Spending per prevalent case,2019 (PPP)	Spending per person,2019 (PPP)	Spending per person over 65 years old (PPP)
Central Asia	$898 437 ($527 520 - $1 313 686)	$2620.26 ($1553.13 - $3753.47)	$9.61 ($5.64 - $14.05)	$130.34 ($76.53 - $190.59)
Armenia	$106 695 ($49 688 - $194 323)	$3728.60 ($1742.99 - $6789.24)	$35.33 ($16.46 - $64.35)	$264.43 ($123.15 - $481.60)
Azerbaijan	$90 600 ($42 080 - $166 161)	$2290.78 ($1085.88 - $4111.00)	$8.81 ($4.09 - $16.17)	$123.31 ($57.27 - $226.15)
Georgia	$172 533 ($82 933 - $314 381)	$3466.98 ($1655.71 - $6129.73)	$47.08 ($22.63 - $85.79)	$280.71 ($134.93 - $511.49)
Kazakhstan	$267 202 ($120 088 - $478 822)	$3016.38 ($1382.79 - $5190.69)	$14.53 ($6.53 - $26.03)	$159.66 ($71.76 - $286.12)
Kyrgyzstan	$38 161 ($18 827 - $69 629)	$1558.96 ($760.97 - $2824.81)	$5.84 ($2.88 - $10.65)	$83.28 ($41.09 - $151.95)
Mongolia	$18 319 ($8399 - $33 196)	$1939.91 ($927.83 - $3414.65)	$5.41 ($2.48 - $9.80)	$84.87 ($38.91 - $153.79)
Tajikistan	$19 983 ($9579 - $35 670)	$1106.31 ($538.00 - $2010.59)	$2.11 ($1.01 - $3.76)	$37.72 ($18.08 - $67.32)
Turkmenistan	$60 076 ($28 640 - $107 278)	$2971.43 ($1378.35 - $5259.58)	$11.82 ($5.63 - $21.11)	$160.30 ($76.42 - $286.24)
Uzbekistan	$124 864 ($60 232 - $224 960)	$1946.85 ($895.54 - $3540.92)	$3.71 ($1.79 - $6.68)	$66.15 ($31.91 - $119.17)
Central Europe	$9 422 148 ($5 152 198 - $14 590 636)	$5437.25 ($3058.98 - $8149.34)	$82.49 ($45.11 - $127.74)	$421.96 ($230.74 - $653.42)
Albania	$84 782 ($40 071 - $150 539)	$2666.70 ($1295.55 - $4840.01)	$31.17 ($14.73 - $55.34)	$194.85 ($92.09 - $345.97)
Bosnia and Herzegovina	$165 091 ($78 782 - $294 822)	$4045.19 ($1978.56 - $7224.92)	$50.03 ($23.87 - $89.34)	$279.24 ($133.25 - $498.67)
Bulgaria	$579 231 ($278 652 - $1 108 132)	$5118.46 ($2447.43 - $9740.06)	$83.53 ($40.18 - $159.80)	$373.41 ($179.64 - $714.38)
Croatia	$379 833 ($174 593 - $702 993)	$5084.01 ($2370.52 - $9425.97)	$89.42 ($41.10 - $165.49)	$422.55 ($194.23 - $782.06)
Czech Republic	$1 232 773 ($573 856 - $2 199 391)	$7058.46 ($3372.47 - $13174.07)	$115.82 ($53.92 - $206.64)	$546.36 ($254.33 - $974.76)
Hungary	$903 705 ($420 796 - $1 698 995)	$5531.74 ($2555.98 - $10280.82)	$93.41 ($43.50 - $175.62)	$446.28 ($207.80 - $839.01)
Macedonia	$74 225 ($34 032 - $131 838)	$3978.85 ($1862.08 - $7122.53)	$34.48 ($15.81 - $61.24)	$235.18 ($107.83 - $417.72)
Montenegro	$36 644 ($16 433 - $67 150)	$5353.14 ($2385.89 - $9685.40)	$59.07 ($26.49 - $108.25)	$368.89 ($165.43 - $675.98)
Poland	$3 351 046 ($1 495 448 - $6 211 014)	$5732.53 ($2517.90 - $10805.56)	$87.19 ($38.91 - $161.60)	$465.44 ($207.71 - $862.68)
Romania	$1 471 471 ($677 895 - $2 760 551)	$4803.15 ($2168.41 - $8536.85)	$76.49 ($35.24 - $143.50)	$379.97 ($175.05 - $712.85)
Serbia	$471 848 ($204 856 - $878 382)	$4344.47 ($1873.95 - $8088.03)	$53.95 ($23.42 - $100.42)	$277.01 ($120.27 - $515.67)
Slovakia	$390 742 ($170 021 - $722 351)	$5682.61 ($2477.67 - $10645.01)	$71.86 ($31.27 - $132.85)	$414.24 ($180.24 - $765.79)
Slovenia	$280 752 ($128 720 - $528 405)	$6908.22 ($3212.81 - $12794.21)	$135.35 ($62.06 - $254.74)	$641.14 ($293.95 - $1206.68)
Eastern Europe	$13 056 951 ($5 866 876 - $22 706 370)	$5190.14 ($2431.91 - $8841.68)	$62.18 ($27.94 - $108.14)	$378.55 ($170.09 - $658.30)
Belarus	$538 625 ($249 215 - $1 009 355)	$4370.60 ($1988.05 - $8192.72)	$56.69 ($26.23 - $106.24)	$348.01 ($161.02 - $652.15)
Estonia	$165 500 ($71 278 - $313 433)	$6894.33 ($2966.27 - $12885.36)	$126.11 ($54.31 - $238.83)	$602.64 ($259.55 - $1141.31)
Latvia	$214 364 ($95 892 - $407 719)	$6218.17 ($2771.12 - $11550.20)	$111.92 ($50.07 - $212.88)	$520.89 ($233.01 - $990.73)
Lithuania	$388 628 ($161 859 - $733 876)	$7727.84 ($3337.27 - $14442.05)	$139.08 ($57.93 - $262.64)	$667.42 ($277.97 - $1260.34)

Moldova	$120 125 ($54 052 - $228 456)	$2852.79 ($1269.51 - $5323.28)	$32.57 ($14.66 - $61.94)	$214.30 ($96.43 - $407.56)
Russian Federation	$9 229 061 ($3 760 600 - $17 299 792)	$5489.72 ($2320.80 - $10424.63)	$62.90 ($25.63 - $117.91)	$392.80 ($160.06 - $736.30)
Ukraine	$2 400 644 ($1 013 747 - $4 663 420)	$4281.21 ($1743.09 - $8061.83)	$54.51 ($23.02 - $105.88)	$315.04 ($133.04 - $611.99)
Australasia	$2 511 296 ($1 274 171 - $4 375 948)	$6629.19 ($3501.82 - $11468.55)	$86.41 ($43.84 - $150.56)	$489.55 ($248.39 - $853.05)
Australia	$2 122 098 ($1 015 476 - $3 831 251)	$6667.50 ($3301.05 - $12173.38)	$86.38 ($41.33 - $155.94)	$491.12 ($235.01 - $886.67)
New Zealand	$389 197 ($185 833 - $703 529)	$6428.41 ($3169.03 - $11703.50)	$86.57 ($41.34 - $156.49)	$481.19 ($229.76 - $869.81)
High-income Asia Pacific	$44 812 262 ($21 534 351 - $82 160 519)	$9802.44 ($4742.13 - $17725.93)	$239.27 ($114.98 - $438.68)	$969.55 ($465.91 - $1777.60)
Brunei	$4696 ($2172 - $8724)	$4697.77 ($2133.15 - $8740.87)	$10.74 ($4.97 - $19.96)	$186.11 ($86.10 - $345.78)
Japan	$37 343 072 ($17 399 642 - $71 956 935)	$9503.19 ($4323.55 - $18270.13)	$292.23 ($136.16 - $563.09)	$1002.26 ($466.99 - $1931.27)
Singapore	$305 766 ($146 201 - $524 763)	$6798.49 ($3207.56 - $11301.83)	$53.95 ($25.80 - $92.59)	$416.05 ($198.93 - $714.04)
South Korea	$7 158 727 ($2 965 331 - $14 119 543)	$12004.48 ($4947.06 - $22876.43)	$134.06 ($55.53 - $264.42)	$872.93 ($361.59 - $1721.73)
High-income North America	$127 145 208 ($102 942 019 - $155 142 331)	$23461.76 ($18561.93 - $29039.52)	$348.77 ($282.38 - $425.57)	$1979.40 ($1602.61 - $2415.27)
Canada	$4 711 910 ($2 016 122 - $9 425 648)	$8858.75 ($3831.18 - $17040.26)	$129.02 ($55.21 - $258.10)	$677.62 ($289.94 - $1355.50)
Greenland	$3429 ($1633 - $6086)	$9333.89 ($4378.84 - $17009.52)	$61.03 ($29.05 - $108.31)	$573.31 ($272.92 - $1017.37)
United States	$122 429 867 ($99 041 778 - $150 811 724)	$25047.24 ($19493.21 - $31295.44)	$373.29 ($301.98 - $459.82)	$2137.60 ($1729.25 - $2633.14)
Southern Latin America	$1 697 226 ($913 560 - $2 821 394)	$2922.49 ($1584.87 - $4796.86)	$25.43 ($13.69 - $42.27)	$195.77 ($105.38 - $325.44)
Argentina	$1 180 616 ($581 187 - $2 136 061)	$3127.31 ($1549.10 - $5628.50)	$26.17 ($12.88 - $47.35)	$205.90 ($101.36 - $372.52)
Chile	$423 360 ($194 603 - $769 033)	$2659.43 ($1197.61 - $4952.63)	$23.26 ($10.69 - $42.26)	$178.43 ($82.02 - $324.12)
Uruguay	$93 249 ($44 429 - $165 976)	$2116.06 ($1046.78 - $3790.32)	$27.14 ($12.93 - $48.30)	$165.70 ($78.95 - $294.94)
Western Europe	$64 481 257 ($37 471 779 - $96 532 729)	$8836.10 ($5213.24 - $12927.88)	$147.94 ($85.97 - $221.48)	$702.95 ($408.51 - $1052.37)
Andorra	$7104 ($3214 - $13 317)	$7224.63 ($3264.83 - $13530.55)	$85.53 ($38.69 - $160.32)	$556.10 ($251.55 - $1042.40)
Austria	$1 290 544 ($600 607 - $2 332 957)	$9524.75 ($4321.84 - $17584.43)	$144.74 ($67.36 - $261.65)	$729.62 ($339.56 - $1318.96)
Belgium	$1 610 213 ($756 289 - $2 944 737)	$9021.99 ($4441.88 - $16107.69)	$141.01 ($66.23 - $257.88)	$701.22 ($329.35 - $1282.38)
Cyprus	$57 352 ($25 694 - $106 604)	$5028.64 ($2324.79 - $9153.92)	$43.66 ($19.56 - $81.16)	$287.74 ($128.91 - $534.83)
Denmark	$769 105 ($345 299 - $1 395 608)	$9964.61 ($4646.35 - $18012.85)	$132.54 ($59.51 - $240.51)	$639.08 ($286.92 - $1159.66)
Finland	$784 305 ($377 199 - $1 348 948)	$8545.90 ($3992.63 - $14376.92)	$141.72 ($68.16 - $243.75)	$608.02 ($292.42 - $1045.74)
France	$9 577 753 ($4 718 157 - $16 982 593)	$8532.51 ($4213.14 - $14998.70)	$144.67 ($71.27 - $256.52)	$683.44 ($336.67 - $1211.83)
Germany	$15 047 423 ($6 782 435 - $28 484 446)	$9523.16 ($4323.44 - $17784.85)	$177.21 ($79.87 - $335.45)	$795.62 ($358.61 - $1506.09)
Greece	$1 070 733 ($494 979 - $1 907 945)	$5533.14 ($2578.10 - $9946.38)	$103.58 ($47.88 - $184.57)	$451.14 ($208.55 - $803.88)
Iceland	$30 842 ($13 122 - $58 584)	$7619.30 ($3262.53 - $14205.66)	$89.43 ($38.05 - $169.87)	$548.28 ($233.27 - $1041.44)
Ireland	$590 442 ($250 365 - $1 091 421)	$11965.52 ($5029.16 - $22524.94)	$120.24 ($50.99 - $222.27)	$770.97 ($326.91 - $1425.12)

Israel	$411 717<br>($191 142 - $731 971)	$5199.30<br>($2366.85 - $9121.71)	$44.23<br>($20.53 - $78.63)	$314.12<br>($145.83 - $558.46)
Italy	$9 748 308<br>($4 849 364 - $17 005 821)	$7130.15<br>($3624.58 - $12167.37)	$161.63<br>($80.40 - $281.96)	$685.06<br>($340.79 - $1195.07)
Luxembourg	$65 138<br>($28 734 - $121 399)	$10307.26<br>($4571.82 - $18695.13)	$105.31<br>($46.46 - $196.27)	$673.38<br>($297.05 - $1255.00)
Malta	$53 283<br>($25 475 - $99 066)	$8170.56<br>($3949.06 - $15036.47)	$121.31<br>($58.00 - $225.55)	$541.81<br>($259.04 - $1007.35)
Netherlands	$3 102 427<br>($1 539 065 - $5 599 925)	$12038.44<br>($5943.45 - $21316.00)	$180.83<br>($89.71 - $326.40)	$886.61<br>($439.83 - $1600.34)
Norway	$664 192<br>($310 036 - $1 224 934)	$9364.15<br>($4353.02 - $16749.16)	$124.17<br>($57.96 - $229.01)	$676.12<br>($315.61 - $1246.94)
Portugal	$913 764<br>($457 986 - $1 609 094)	$4813.02<br>($2488.20 - $8508.98)	$85.79<br>($43.00 - $151.07)	$376.64<br>($188.77 - $663.24)
Spain	$5 540 349<br>($2 603 473 - $10 247 159)	$7287.51<br>($3447.64 - $13262.85)	$120.39<br>($56.57 - $222.66)	$591.26<br>($277.84 - $1093.56)
Sweden	$1 614 777<br>($709 039 - $2 916 962)	$11049.77<br>($4703.39 - $19876.13)	$157.96<br>($69.36 - $285.35)	$735.61<br>($323.00 - $1328.81)
Switzerland	$944 229<br>($435 357 - $1 666 789)	$6961.41<br>($3342.62 - $12507.49)	$107.60<br>($49.61 - $189.94)	$548.41<br>($252.86 - $968.07)
United Kingdom	$10 587 247<br>($4 857 553 - $19 478 149)	$12710.93<br>($5952.36 - $22977.91)	$157.50<br>($72.26 - $289.77)	$820.38<br>($376.40 - $1509.31)
Andean Latin America	$508 380<br>($281 794 - $813 128)	$1726.93<br>($960.11 - $2744.80)	$7.99<br>($4.43 - $12.79)	$83.87<br>($46.49 - $134.14)
Bolivia	$56 986<br>($28 332 - $102 485)	$1473.63<br>($746.52 - $2620.68)	$4.74<br>($2.36 - $8.53)	$54.57<br>($27.13 - $98.14)
Ecuador	$154 458<br>($75 766 - $266 919)	$2035.97<br>($1004.85 - $3489.27)	$8.78<br>($4.31 - $15.18)	$94.52<br>($46.36 - $163.34)
Peru	$296 935<br>($134 821 - $548 357)	$1651.24<br>($778.39 - $2964.66)	$8.73<br>($3.97 - $16.13)	$87.77<br>($39.85 - $162.09)
Caribbean	$866 754<br>($474 182 - $1 379 504)	$3254.17<br>($1783.29 - $5143.16)	$19.05<br>($10.42 - $30.31)	$166.55<br>($91.12 - $265.08)
Antigua and Barbuda	$1190<br>($556 - $2131)	$2531.50<br>($1177.63 - $4453.95)	$13.45<br>($6.28 - $24.09)	$127.47<br>($59.57 - $228.36)
The Bahamas	$6983<br>($3292 - $12 890)	$4045.68<br>($1937.09 - $7458.49)	$18.53<br>($8.73 - $34.20)	$205.12<br>($96.68 - $378.63)
Barbados	$5180<br>($2446 - $9426)	$1917.31<br>($890.06 - $3353.36)	$17.40<br>($8.21 - $31.66)	$106.47<br>($50.27 - $193.73)
Belize	$1306<br>($618 - $2368)	$1061.22<br>($498.30 - $1893.77)	$3.19<br>($1.51 - $5.77)	$46.65<br>($22.08 - $84.55)
Bermuda	$4560<br>($2206 - $8117)	$5590.48<br>($2676.37 - $9926.48)	$71.21<br>($34.46 - $126.77)	$356.47<br>($172.50 - $634.61)
Cuba	$517 444<br>($246 718 - $925 023)	$4805.64<br>($2331.68 - $8402.02)	$45.56<br>($21.72 - $81.44)	$280.35<br>($133.67 - $501.17)
Dominica	$903<br>($429 - $1603)	$1856.28<br>($885.26 - $3200.34)	$13.15<br>($6.25 - $23.34)	$101.86<br>($48.42 - $180.83)
Dominican Republic	$92 061<br>($46 001 - $169 718)	$2105.79<br>($1070.43 - $3770.07)	$8.46<br>($4.23 - $15.60)	$92.45<br>($46.19 - $170.43)
Grenada	$944<br>($452 - $1721)	$1924.38<br>($941.32 - $3469.81)	$9.14<br>($4.38 - $16.68)	$89.63<br>($42.98 - $163.53)
Guyana	$3874<br>($1936 - $7066)	$1609.94<br>($783.16 - $2850.69)	$5.03<br>($2.51 - $9.17)	$63.14<br>($31.56 - $115.17)
Haiti	$9509<br>($4904 - $17 295)	$397.42<br>($207.90 - $712.59)	$0.77<br>($0.40 - $1.39)	$11.57<br>($5.97 - $21.04)
Jamaica	$32 277<br>($15 174 - $58 164)	$1818.59<br>($857.46 - $3409.12)	$11.48<br>($5.40 - $20.69)	$109.11<br>($51.29 - $196.61)
Puerto Rico	$145 426<br>($69 542 - $259 697)	$3011.38<br>($1404.34 - $5295.89)	$41.30<br>($19.75 - $73.75)	$196.84<br>($94.13 - $351.50)
Saint Lucia	$1791<br>($858 - $3127)	$1651.87<br>($786.46 - $2883.95)	$10.25<br>($4.91 - $17.91)	$89.82<br>($43.05 - $156.86)
Saint Vincent and the Grenadines	$946<br>($474 - $1672)	$1467.09<br>($725.60 - $2546.20)	$8.36<br>($4.19 - $14.78)	$72.63<br>($36.39 - $128.39)
Suriname	$7253<br>($3576 - $12 540)	$2574.55<br>($1274.92 - $4407.39)	$12.60<br>($6.21 - $21.78)	$126.84<br>($62.54 - $219.29)
Trinidad and Tobago	$32 888<br>($14 906 - $58 920)	$3571.07<br>($1653.47 - $6307.95)	$23.70<br>($10.74 - $42.47)	$180.63<br>($81.87 - $323.60)

Virgin Islands, U.S.	$2217 ($962 - $4120)	$2444.88 ($1076.52 - $4581.37)	$21.32 ($9.25 - $39.63)	$111.04 ($48.19 - $206.40)
Central Latin America	$2 149 726 ($1 278 627 - $3 240 158)	$1782.71 ($1046.83 - $2646.58)	$8.60 ($5.11 - $12.96)	$88.84 ($52.84 - $133.90)
Colombia	$651 382 ($337 699 - $1 130 680)	$1985.13 ($1009.22 - $3436.40)	$13.63 ($7.07 - $23.67)	$120.66 ($62.55 - $209.44)
Costa Rica	$77 637 ($38 932 - $140 733)	$2577.63 ($1335.27 - $4574.32)	$16.46 ($8.25 - $29.84)	$153.07 ($76.76 - $277.47)
El Salvador	$50 589 ($24 593 - $89 647)	$1341.07 ($645.15 - $2316.42)	$8.09 ($3.93 - $14.33)	$78.33 ($38.08 - $138.81)
Guatemala	$56 777 ($28 618 - $99 753)	$1050.15 ($534.43 - $1856.88)	$3.19 ($1.61 - $5.61)	$42.77 ($21.56 - $75.15)
Honduras	$24 346 ($12 559 - $42 490)	$923.48 ($479.26 - $1620.67)	$2.48 ($1.28 - $4.33)	$33.97 ($17.53 - $59.29)
Mexico	$1 070 870 ($535 299 - $1 869 308)	$1994.71 ($1018.79 - $3446.24)	$8.57 ($4.28 - $14.96)	$91.05 ($45.51 - $158.94)
Nicaragua	$22 691 ($11 236 - $39 259)	$1114.73 ($547.82 - $1898.65)	$3.49 ($1.73 - $6.03)	$47.92 ($23.73 - $82.90)
Panama	$84 480 ($42 105 - $154 599)	$3397.44 ($1692.94 - $5986.18)	$20.31 ($10.12 - $37.16)	$192.61 ($95.99 - $352.47)
Venezuela	$110 950 ($55 544 - $199 263)	$752.87 ($371.39 - $1329.22)	$3.95 ($1.98 - $7.10)	$37.87 ($18.96 - $68.01)
Tropical Latin America	$3 611 774 ($1 831 691 - $6 251 797)	$2080.41 ($1085.94 - $3514.48)	$16.15 ($8.19 - $27.96)	$150.98 ($76.57 - $261.35)
Brazil	$3 535 066 ($1 750 559 - $6 150 563)	$2079.05 ($1072.24 - $3533.82)	$16.32 ($8.08 - $28.39)	$151.45 ($75.00 - $263.51)
Paraguay	$76 708 ($37 109 - $138 698)	$2145.14 ($1028.19 - $3897.75)	$11.07 ($5.35 - $20.01)	$132.15 ($63.93 - $238.95)
North Africa and Middle East	$5 791 698 ($3 499 556 - $8 501 562)	$2337.69 ($1439.70 - $3381.73)	$9.52 ($5.75 - $13.98)	$132.00 ($79.76 - $193.76)
Afghanistan	$38 072 ($19 162 - $67 746)	$697.13 ($355.33 - $1213.36)	$0.99 ($0.50 - $1.77)	$17.33 ($8.72 - $30.83)
Algeria	$373 551 ($181 232 - $649 216)	$1935.83 ($962.59 - $3334.70)	$8.93 ($4.33 - $15.51)	$107.01 ($51.92 - $185.98)
Bahrain	$14 414 ($7040 - $25 087)	$3842.29 ($1943.92 - $6599.49)	$9.99 ($4.88 - $17.39)	$224.21 ($109.50 - $390.22)
Egypt	$659 112 ($331 559 - $1 184 761)	$2232.16 ($1107.97 - $3926.02)	$6.65 ($3.35 - $11.96)	$99.09 ($49.84 - $178.11)
Iran	$1 007 239 ($500 251 - $1 764 585)	$2143.32 ($1062.34 - $3724.07)	$11.95 ($5.93 - $20.93)	$145.91 ($72.47 - $255.61)
Iraq	$132 351 ($67 294 - $230 165)	$1116.96 ($553.32 - $1970.40)	$3.14 ($1.60 - $5.46)	$51.63 ($26.25 - $89.79)
Jordan	$66 767 ($33 029 - $117 753)	$2090.20 ($1028.39 - $3636.89)	$5.74 ($2.84 - $10.12)	$97.36 ($48.16 - $171.70)
Kuwait	$64 683 ($31 271 - $112 361)	$4534.07 ($2198.25 - $7810.54)	$14.61 ($7.06 - $25.38)	$313.50 ($151.57 - $544.58)
Lebanon	$83 956 ($40 293 - $147 122)	$2103.44 ($1005.96 - $3629.78)	$16.22 ($7.78 - $28.42)	$146.18 ($70.16 - $256.17)
Libya	$97 800 ($47 864 - $176 583)	$3214.66 ($1592.09 - $5684.32)	$14.52 ($7.11 - $26.22)	$223.26 ($109.27 - $403.12)
Morocco	$194 693 ($98 344 - $337 824)	$1118.03 ($571.90 - $1922.35)	$5.42 ($2.74 - $9.40)	$66.46 ($33.57 - $115.31)
Oman	$18 544 ($9128 - $33 612)	$2752.85 ($1362.45 - $4919.58)	$4.05 ($1.99 - $7.33)	$107.43 ($52.88 - $194.71)
Palestine	$4421 ($2333 - $8000)	$367.81 ($189.96 - $652.83)	$0.89 ($0.47 - $1.61)	$15.54 ($8.20 - $28.13)
Qatar	$13 091 ($6484 - $22 730)	$4865.47 ($2427.00 - $8279.33)	$4.57 ($2.26 - $7.94)	$202.14 ($100.11 - $350.95)
Saudi Arabia	$359 190 ($176 095 - $658 446)	$5296.73 ($2647.40 - $9608.33)	$10.05 ($4.93 - $18.43)	$260.88 ($127.90 - $478.22)
Sudan	$102 170 ($49 703 - $179 392)	$1018.08 ($497.44 - $1769.58)	$2.50 ($1.22 - $4.40)	$41.24 ($20.06 - $72.41)
Syria	$316 809 ($159 718 - $551 113)	$4990.06 ($2455.80 - $8589.81)	$21.86 ($11.02 - $38.03)	$274.76 ($138.52 - $477.97)
Tunisia	$203 039 ($100 462 - $363 650)	$2402.61 ($1221.55 - $4189.58)	$17.55 ($8.68 - $31.43)	$167.14 ($82.70 - $299.36)
Turkey	$1 965 353 ($947 378 - $3 602 316)	$3063.04 ($1486.19 - $5543.93)	$24.16 ($11.64 - $44.28)	$233.82 ($112.71 - $428.57)

United Arab Emirates	$49 949<br>($23 858 - $88 870)	$6198.67<br>($2974.54 - $10968.41)	$5.40<br>($2.58 - $9.62)	$294.11<br>($140.48 - $523.28)
Yemen	$26 484<br>($13 864 - $46 560)	$409.80<br>($210.80 - $713.15)	$0.84<br>($0.44 - $1.48)	$14.29<br>($7.48 - $25.12)
South Asia	$3 340 044<br>($1 770 906 - $5 708 511)	$720.71<br>($392.57 - $1187.83)	$1.85<br>($0.98 - $3.16)	$22.87<br>($12.13 - $39.09)
Bangladesh	$222 302<br>($109 729 - $386 651)	$413.31<br>($201.98 - $722.64)	$1.40<br>($0.69 - $2.43)	$16.49<br>($8.14 - $28.68)
Bhutan	$1589<br>($748 - $2805)	$738.14<br>($351.08 - $1279.11)	$2.11<br>($0.99 - $3.72)	$26.94<br>($12.69 - $47.55)
India	$2 909 995<br>($1 464 438 - $5 183 340)	$788.21<br>($401.83 - $1357.70)	$2.09<br>($1.05 - $3.73)	$25.07<br>($12.62 - $44.66)
Nepal	$38 998<br>($19 983 - $68 719)	$497.90<br>($251.98 - $870.66)	$1.28<br>($0.66 - $2.26)	$16.09<br>($8.25 - $28.35)
Pakistan	$167 159<br>($82 368 - $297 020)	$516.68<br>($255.91 - $888.36)	$0.75<br>($0.37 - $1.33)	$11.94<br>($5.88 - $21.22)
East Asia	$32 582 377<br>($16 474 691 - $55 275 247)	$2407.57<br>($1235.94 - $4034.41)	$22.13<br>($11.19 - $37.55)	$162.75<br>($82.29 - $276.11)
China	$29 899 313<br>($14 409 590 - $51 984 458)	$2280.94<br>($1114.97 - $3878.93)	$21.02<br>($10.13 - $36.55)	$154.59<br>($74.50 - $268.77)
North Korea	$30 950<br>($14 966 - $57 135)	$179.68<br>($91.42 - $322.58)	$1.18<br>($0.57 - $2.18)	$10.38<br>($5.02 - $19.15)
Taiwan	$2 652 113<br>($1 220 781 - $4 741 790)	$10419.05<br>($4675.87 - $19020.47)	$112.28<br>($51.68 - $200.75)	$698.81<br>($321.67 - $1249.43)
Oceania	$15 879<br>($9439 - $23 470)	$714.88<br>($433.35 - $1055.04)	$1.26<br>($0.75 - $1.86)	$19.84<br>($11.80 - $29.33)
American Samoa	$344<br>($178 - $596)	$1604.06<br>($834.09 - $2781.40)	$6.21<br>($3.20 - $10.73)	$73.58<br>($37.92 - $127.28)
Federated States of Micronesia	$111<br>($57 - $193)	$485.43<br>($252.84 - $833.29)	$1.08<br>($0.55 - $1.89)	$17.88<br>($9.12 - $31.09)
Fiji	$4795<br>($2413 - $8518)	$1763.46<br>($911.67 - $3136.95)	$5.26<br>($2.65 - $9.35)	$67.69<br>($34.06 - $120.24)
Guam	$2940<br>($1397 - $5255)	$2712.56<br>($1288.92 - $4822.33)	$17.23<br>($8.19 - $30.80)	$151.49<br>($71.97 - $270.72)
Kiribati	$164<br>($82 - $290)	$803.09<br>($410.11 - $1410.80)	$1.38<br>($0.69 - $2.44)	$23.19<br>($11.65 - $40.96)
Marshall Islands	$101<br>($52 - $176)	$1055.53<br>($553.92 - $1733.05)	$1.78<br>($0.92 - $3.09)	$29.73<br>($15.36 - $51.67)
Northern Mariana Islands	$334<br>($167 - $585)	$1755.09<br>($885.72 - $3018.87)	$7.87<br>($3.93 - $13.77)	$84.60<br>($42.30 - $148.07)
Papua New Guinea	$5389<br>($2686 - $9726)	$363.34<br>($183.14 - $642.17)	$0.55<br>($0.27 - $0.99)	$8.96<br>($4.46 - $16.16)
Samoa	$744<br>($356 - $1385)	$1093.07<br>($543.00 - $2009.96)	$3.52<br>($1.69 - $6.55)	$51.41<br>($24.65 - $95.79)
Solomon Islands	$330<br>($159 - $596)	$366.00<br>($179.49 - $654.64)	$0.50<br>($0.24 - $0.91)	$8.48<br>($4.09 - $15.30)
Tonga	$413<br>($212 - $763)	$929.70<br>($474.53 - $1638.48)	$4.03<br>($2.07 - $7.45)	$46.13<br>($23.72 - $85.23)
Vanuatu	$214<br>($108 - $377)	$339.72<br>($175.24 - $602.41)	$0.73<br>($0.37 - $1.28)	$10.38<br>($5.24 - $18.30)
Southeast Asia	$5 371 772<br>($3 251 444 - $7 991 936)	$1928.62<br>($1166.97 - $2794.97)	$7.98<br>($4.83 - $11.88)	$94.69<br>($57.31 - $140.87)
Cambodia	$46 436<br>($23 892 - $83 964)	$899.21<br>($461.13 - $1577.73)	$2.80<br>($1.44 - $5.06)	$37.29<br>($19.19 - $67.43)
Indonesia	$1 429 136<br>($669 691 - $2 558 025)	$1710.39<br>($828.09 - $3070.21)	$5.51<br>($2.58 - $9.86)	$74.51<br>($34.92 - $133.37)
Laos	$14 513<br>($6827 - $27 061)	$838.47<br>($409.90 - $1464.60)	$2.03<br>($0.95 - $3.78)	$30.75<br>($14.46 - $57.33)
Malaysia	$469 995<br>($222 538 - $846 639)	$3663.01<br>($1737.56 - $6473.98)	$15.02<br>($7.11 - $27.05)	$175.25<br>($82.98 - $315.69)
Maldives	$4839<br>($2457 - $8751)	$3385.13<br>($1734.62 - $5870.51)	$9.71<br>($4.93 - $17.56)	$172.56<br>($87.63 - $312.06)
Mauritius	$33 412<br>($16 453 - $58 093)	$3543.03<br>($1749.76 - $6109.54)	$26.17<br>($12.89 - $45.50)	$203.86<br>($100.39 - $354.45)
Myanmar	$227 556<br>($112 115 - $419 311)	$1088.07<br>($540.34 - $2001.11)	$4.16<br>($2.05 - $7.67)	$50.07<br>($24.67 - $92.25)

Philippines	$509 669<br>($245 038 - $881 534)	$1563.22<br>($771.59 - $2670.76)	$4.54<br>($2.19 - $7.86)	$61.30<br>($29.47 - $106.03)
Sri Lanka	$307 737<br>($145 805 - $554 958)	$2344.82<br>($1161.78 - $4156.34)	$14.08<br>($6.67 - $25.39)	$120.62<br>($57.15 - $217.52)
Seychelles	$1714<br>($847 - $2896)	$3126.24<br>($1575.22 - $5265.24)	$16.78<br>($8.29 - $28.36)	$175.56<br>($86.77 - $296.74)
Thailand	$1 475 784<br>($685 379 - $2 618 591)	$2404.87<br>($1159.07 - $4196.40)	$21.05<br>($9.78 - $37.35)	$158.15<br>($73.45 - $280.61)
Timor-Leste	$2901<br>($1450 - $5178)	$848.25<br>($428.43 - $1466.67)	$2.17<br>($1.09 - $3.88)	$26.92<br>($13.46 - $48.06)
Vietnam	$848 079<br>($409 200 - $1 509 559)	$1854.06<br>($924.24 - $3314.57)	$8.80<br>($4.25 - $15.66)	$104.71<br>($50.52 - $186.37)
Central Sub-Saharan Africa	$72 076<br>($41 863 - $108 333)	$326.63<br>($191.15 - $489.13)	$0.55<br>($0.32 - $0.82)	$9.37<br>($5.44 - $14.08)
Angola	$24 492<br>($11 949 - $42 515)	$571.54<br>($286.52 - $978.72)	$0.81<br>($0.40 - $1.41)	$13.90<br>($6.78 - $24.13)
Central African Republic	$1522<br>($769 - $2620)	$192.44<br>($97.91 - $334.64)	$0.29<br>($0.15 - $0.49)	$4.94<br>($2.49 - $8.50)
Congo	$4420<br>($2173 - $8099)	$399.43<br>($193.47 - $719.25)	$0.84<br>($0.41 - $1.54)	$14.25<br>($7.01 - $26.11)
Democratic Republic of the Congo	$29 554<br>($14 799 - $52 089)	$195.02<br>($95.34 - $343.15)	$0.34<br>($0.17 - $0.59)	$5.76<br>($2.89 - $10.16)
Equatorial Guinea	$3938<br>($1809 - $7253)	$1793.76<br>($853.10 - $3444.55)	$2.77<br>($1.27 - $5.11)	$55.72<br>($25.61 - $102.65)
Gabon	$8148<br>($3881 - $14 714)	$1603.01<br>($753.28 - $2914.66)	$4.66<br>($2.22 - $8.41)	$70.45<br>($33.56 - $127.22)
Eastern Sub-Saharan Africa	$265 265<br>($162 788 - $381 733)	$428.94<br>($261.45 - $607.42)	$0.64<br>($0.40 - $0.93)	$10.67<br>($6.55 - $15.36)
Burundi	$4825<br>($2294 - $9149)	$307.40<br>($149.92 - $562.26)	$0.40<br>($0.19 - $0.77)	$6.51<br>($3.09 - $12.34)
Comoros	$1273<br>($611 - $2272)	$573.80<br>($271.93 - $1014.38)	$1.78<br>($0.86 - $3.18)	$23.38<br>($11.22 - $41.71)
Djibouti	$651<br>($330 - $1149)	$342.07<br>($176.83 - $586.86)	$0.54<br>($0.27 - $0.96)	$9.15<br>($4.65 - $16.17)
Eritrea	$2280<br>($1114 - $4033)	$268.50<br>($136.86 - $473.72)	$0.34<br>($0.17 - $0.60)	$6.48<br>($3.17 - $11.46)
Ethiopia	$51 062<br>($25 620 - $92 724)	$303.53<br>($154.83 - $537.41)	$0.47<br>($0.24 - $0.86)	$7.80<br>($3.91 - $14.17)
Kenya	$67 929<br>($32 059 - $126 108)	$838.15<br>($385.94 - $1524.72)	$1.35<br>($0.64 - $2.51)	$24.20<br>($11.42 - $44.93)
Madagascar	$12 325<br>($6227 - $21 538)	$356.91<br>($182.57 - $607.17)	$0.46<br>($0.23 - $0.81)	$8.30<br>($4.19 - $14.50)
Malawi	$10 912<br>($5465 - $19 034)	$375.95<br>($189.17 - $645.43)	$0.59<br>($0.30 - $1.03)	$10.22<br>($5.12 - $17.83)
Mozambique	$12 643<br>($6650 - $22 840)	$309.51<br>($161.95 - $543.46)	$0.43<br>($0.23 - $0.77)	$6.88<br>($3.62 - $12.43)
Rwanda	$12 643<br>($6276 - $22 547)	$548.28<br>($280.05 - $972.79)	$1.00<br>($0.49 - $1.78)	$16.61<br>($8.25 - $29.62)
Somalia	$1989<br>($999 - $3557)	$97.25<br>($49.75 - $167.19)	$0.10<br>($0.05 - $0.17)	$1.58<br>($0.80 - $2.83)
South Sudan	$3707<br>($1906 - $6609)	$258.92<br>($131.24 - $454.94)	$0.40<br>($0.21 - $0.71)	$6.26<br>($3.22 - $11.15)
Tanzania	$42 394<br>($20 763 - $72 712)	$425.49<br>($207.98 - $721.81)	$0.75<br>($0.37 - $1.28)	$11.26<br>($5.51 - $19.31)
Uganda	$25 952<br>($12 752 - $46 653)	$471.51<br>($232.10 - $841.46)	$0.63<br>($0.31 - $1.13)	$10.46<br>($5.14 - $18.80)
Zambia	$14 676<br>($7064 - $25 155)	$612.99<br>($302.09 - $1053.39)	$0.80<br>($0.39 - $1.38)	$14.17<br>($6.82 - $24.29)
Southern Sub-Saharan Africa	$604 242<br>($299 393 - $1 019 801)	$2358.10<br>($1177.38 - $3929.73)	$7.69<br>($3.81 - $12.98)	$101.03<br>($50.06 - $170.51)
Botswana	$13 094<br>($6486 - $23 772)	$2506.27<br>($1271.05 - $4549.57)	$5.60<br>($2.77 - $10.16)	$92.62<br>($45.87 - $168.14)
Lesotho	$4597<br>($2191 - $8373)	$950.64<br>($461.60 - $1767.22)	$2.20<br>($1.05 - $4.00)	$32.71<br>($15.59 - $59.59)
Namibia	$12 784<br>($6507 - $22 684)	$1953.91<br>($1030.01 - $3386.85)	$5.32<br>($2.71 - $9.44)	$75.21<br>($38.28 - $133.44)
South Africa	$556 075<br>($261 231 - $960 714)	$2630.65<br>($1264.14 - $4469.02)	$10.00<br>($4.70 - $17.28)	$123.14<br>($57.85 - $212.75)

Swaziland	$3093<br>($1423 - $5497)	$1422.01<br>($661.53 - $2482.84)	$2.71<br>($1.25 - $4.81)	$43.08<br>($19.82 - $76.54)
Zimbabwe	$14 598<br>($6951 - $26 303)	$559.56<br>($263.57 - $1004.66)	$0.97<br>($0.46 - $1.75)	$15.51<br>($7.38 - $27.94)
Western Sub-Saharan Africa	$352 126<br>($203 152 - $530 679)	$581.96<br>($339.20 - $877.25)	$0.77<br>($0.45 - $1.16)	$12.34<br>($7.12 - $18.60)
Benin	$5452<br>($2865 - $9205)	$333.80<br>($171.42 - $568.76)	$0.43<br>($0.23 - $0.73)	$6.60<br>($3.47 - $11.15)
Burkina Faso	$11 883<br>($6021 - $20 102)	$374.06<br>($188.93 - $641.55)	$0.52<br>($0.27 - $0.89)	$7.77<br>($3.94 - $13.15)
Cameroon	$20 613<br>($10 303 - $37 107)	$542.52<br>($273.02 - $949.52)	$0.71<br>($0.35 - $1.28)	$12.24<br>($6.12 - $22.04)
Cape Verde	$2178<br>($1092 - $3819)	$1000.18<br>($495.22 - $1749.37)	$3.87<br>($1.94 - $6.78)	$50.84<br>($25.49 - $89.13)
Chad	$4343<br>($2192 - $7577)	$233.96<br>($119.07 - $413.00)	$0.26<br>($0.13 - $0.46)	$3.80<br>($1.92 - $6.64)
Cote d'Ivoire	$17 383<br>($8949 - $30 192)	$549.60<br>($289.59 - $947.06)	$0.66<br>($0.34 - $1.15)	$11.36<br>($5.85 - $19.73)
The Gambia	$1618<br>($823 - $2842)	$455.01<br>($231.17 - $798.76)	$0.72<br>($0.37 - $1.27)	$11.45<br>($5.82 - $20.11)
Ghana	$42 063<br>($21 455 - $75 028)	$790.44<br>($401.33 - $1408.20)	$1.33<br>($0.68 - $2.38)	$21.29<br>($10.86 - $37.97)
Guinea	$8111<br>($4123 - $14 173)	$392.71<br>($199.71 - $661.89)	$0.64<br>($0.33 - $1.12)	$9.26<br>($4.70 - $16.17)
Guinea-Bissau	$1148<br>($563 - $1921)	$535.46<br>($263.32 - $912.16)	$0.60<br>($0.30 - $1.01)	$10.64<br>($5.22 - $17.82)
Liberia	$3008<br>($1467 - $5257)	$442.32<br>($222.19 - $773.39)	$0.63<br>($0.31 - $1.10)	$11.23<br>($5.48 - $19.63)
Mali	$8654<br>($4231 - $15 221)	$289.81<br>($140.45 - $506.97)	$0.39<br>($0.19 - $0.69)	$5.67<br>($2.77 - $9.97)
Mauritania	$6339<br>($3212 - $11 238)	$798.62<br>($407.27 - $1384.89)	$1.58<br>($0.80 - $2.80)	$23.65<br>($11.98 - $41.93)
Niger	$5242<br>($2588 - $9093)	$213.30<br>($103.63 - $370.46)	$0.23<br>($0.11 - $0.39)	$3.24<br>($1.60 - $5.62)
Nigeria	$186 725<br>($87 161 - $331 387)	$701.27<br>($338.62 - $1220.18)	$0.87<br>($0.41 - $1.54)	$14.39<br>($6.72 - $25.54)
Sao Tome and Principe	$259<br>($130 - $466)	$694.53<br>($354.84 - $1225.43)	$1.26<br>($0.63 - $2.27)	$21.78<br>($10.97 - $39.23)
Senegal	$14 269<br>($6888 - $25 790)	$518.84<br>($248.25 - $920.12)	$0.94<br>($0.46 - $1.70)	$14.14<br>($6.82 - $25.55)
Sierra Leone	$8405<br>($4254 - $15 144)	$670.43<br>($332.07 - $1179.65)	$1.01<br>($0.51 - $1.83)	$15.94<br>($8.07 - $28.72)
Togo	$4431<br>($2296 - $7642)	$394.31<br>($207.32 - $671.47)	$0.56<br>($0.29 - $0.96)	$9.35<br>($4.85 - $16.13)

Notes: All spending measured in 2019 PPP.

Table S10: Attributable dementia spending by GBD regions and countries in 2050 (2019 USD)

	Spending per person attributable to dementia 2050 – baseline	Spending as a percent of total health spending, 2050	Spending per person attributable to dementia 2050 – accelerated diagnosis and treatment rates	Spending per person attributable to dementia 2050 – accelerated nursing home based care rates	Spending per person attributable to dementia 2050 – accelerated unit costs
Central Asia	$39.65 ($18.27- $69.00)	16.5% (7.7% - 28.2%)	$47.34 ($23.73- $77.87)	$46.00 ($21.22- $79.62)	$49.65 ($23.47- $83.51)
Armenia	$291.71 ($59.14- $784.17)	54.6% (10.8% - 149.7%)	$322.20 ($86.04- $820.68)	$319.10 ($64.00- $873.93)	$293.81 ($61.59- $784.17)
Azerbaijan	$82.27 ($16.18- $228.50)	41.4% (8.2% - 120.2%)	$93.82 ($26.44- $251.95)	$88.69 ($17.60- $242.14)	$87.07 ($18.27- $228.63)
Georgia	$113.17 ($19.02- $312.50)	21.9% (3.9% - 60.9%)	$133.73 ($38.38- $327.38)	$146.40 ($23.37- $406.16)	$127.15 ($27.64- $320.73)
Kazakhstan	$38.00 ($6.61- $100.99)	8.9% (1.5% - 23.9%)	$49.51 ($14.73- $119.28)	$47.33 ($7.57- $123.99)	$65.93 ($13.54- $147.12)
Kyrgyzstan	$10.12 ($2.25- $26.98)	10.6% (2.4% - 28.0%)	$13.04 ($4.41- $29.06)	$13.91 ($3.10- $37.07)	$16.09 ($3.97- $37.22)
Mongolia	$29.80 ($5.68- $82.28)	11.5% (2.1% - 33.5%)	$36.78 ($10.90- $90.17)	$37.01 ($6.85- $102.66)	$36.38 ($7.91- $90.69)
Tajikistan	$12.86 ($2.58- $33.50)	19.9% (3.9% - 53.6%)	$15.05 ($4.87- $34.83)	$15.12 ($3.12- $39.04)	$13.51 ($2.81- $33.73)
Turkmenistan	$102.80 ($17.71- $281.65)	14.4% (2.6% - 40.2%)	$123.50 ($35.90- $294.21)	$115.47 ($19.73- $318.31)	$114.38 ($24.93- $290.22)
Uzbekistan	$14.67 ($2.85- $39.45)	11.9% (2.3% - 30.6%)	$17.80 ($5.37- $41.03)	$16.85 ($3.08- $47.36)	$20.72 ($4.77- $47.96)
Central Europe	$293.72 ($126.51- $570.41)	22.0% (9.5% - 42.0%)	$373.89 ($172.98- $672.66)	$358.02 ($149.32- $687.35)	$421.84 ($204.79- $719.26)
Albania	$85.08 ($15.51- $214.15)	25.1% (4.6% - 65.5%)	$101.21 ($31.73- $243.95)	$95.47 ($17.21- $243.30)	$157.77 ($35.66- $350.97)
Bosnia and Herzegovina	$191.31 ($38.05- $553.84)	21.2% (4.2% - 61.2%)	$235.41 ($73.48- $596.81)	$215.40 ($41.54- $616.71)	$260.17 ($63.43- $620.29)
Bulgaria	$265.52 ($51.75- $756.67)	28.1% (5.8% - 82.8%)	$322.51 ($111.16- $788.04)	$336.22 ($66.84- $908.64)	$345.87 ($84.16- $869.09)
Croatia	$206.81 ($34.15- $597.46)	16.7% (2.7% - 48.3%)	$275.77 ($78.59- $678.68)	$258.04 ($42.80- $754.61)	$413.07 ($84.87- $976.62)
Czechia	$388.33 ($78.80- $1021.46)	15.7% (3.1% - 43.9%)	$501.37 ($165.13- $1201.22)	$508.47 ($97.25- $1452.52)	$584.44 ($141.03- $1307.08)
Hungary	$198.32 ($36.44- $529.21)	15.2% (2.8% - 41.3%)	$267.71 ($80.87- $623.13)	$263.87 ($45.48- $712.33)	$366.23 ($86.70- $819.58)
Montenegro	$192.44 ($33.26- $555.99)	24.5% (4.2% - 70.7%)	$239.43 ($66.32- $600.14)	$226.58 ($37.60- $613.86)	$256.67 ($58.65- $579.49)
North Macedonia	$93.71 ($16.88- $247.59)	24.2% (4.4% - 64.2%)	$121.70 ($36.86- $265.77)	$117.23 ($20.27- $309.63)	$215.28 ($49.98- $482.44)
Poland	$350.69 ($71.97- $959.36)	24.3% (5.0% - 67.3%)	$448.00 ($131.16- $1038.61)	$411.55 ($81.53- $1128.16)	$496.41 ($128.17- $1084.11)
Romania	$321.39 ($62.41- $826.18)	32.5% (5.8% - 86.3%)	$395.40 ($117.53- $917.91)	$391.82 ($73.77- $1027.99)	$380.42 ($85.48- $861.21)
Serbia	$154.13 ($27.85- $413.48)	25.5% (4.6% - 68.9%)	$192.36 ($53.59- $428.22)	$178.53 ($30.82- $486.65)	$199.42 ($44.46- $469.45)
Slovakia	$334.63 ($57.62- $960.36)	19.1% (3.2% - 55.1%)	$427.29 ($131.32- $1044.35)	$415.93 ($70.86- $1172.12)	$465.56 ($96.08- $1057.46)
Slovenia	$406.56 ($71.29- $1111.22)	15.5% (2.8% - 42.2%)	$550.53 ($178.72- $1325.54)	$497.97 ($88.35- $1353.18)	$773.84 ($179.68- $1758.08)
Eastern Europe	$162.86 ($47.07- $397.97)	26.5% (7.8% - 64.4%)	$210.03 ($78.64- $459.72)	$179.52 ($51.18- $422.02)	$257.68 ($77.68- $528.39)
Belarus	$170.37 ($33.03- $476.80)	35.7% (6.5% - 98.8%)	$211.97 ($63.02- $500.23)	$184.46 ($37.01- $514.40)	$230.33 ($55.29- $535.47)
Estonia	$630.74 ($107.51- $1819.61)	29.1% (4.9% - 80.5%)	$795.09 ($224.50- $1981.68)	$638.68 ($108.50- $1819.61)	$814.74 ($176.87- $1946.35)

Latvia	$516.86 ($101.47- $1417.14)	38.1% (7.6% - 104.2%)	$653.41 ($190.21- $1639.11)	$533.17 ($102.77- $1481.01)	$695.75 ($150.46- $1546.69)
Lithuania	$488.73 ($89.12- $1360.79)	30.9% (5.8% - 86.6%)	$617.66 ($173.87- $1613.59)	$490.30 ($89.12- $1360.79)	$622.21 ($138.59- $1452.88)
Republic of Moldova	$155.93 ($26.62- $468.50)	66.6% (11.5% - 201.3%)	$183.74 ($50.93- $520.93)	$174.58 ($30.15- $526.56)	$208.87 ($47.20- $527.56)
Russian Federation	$166.83 ($24.53- $476.59)	24.4% (4.0% - 71.0%)	$216.47 ($63.49- $538.66)	$180.83 ($26.49- $503.42)	$273.57 ($54.20- $616.49)
Ukraine	$94.83 ($16.38- $280.18)	38.6% (6.7% - 115.3%)	$122.16 ($34.16- $323.66)	$124.11 ($21.50- $370.40)	$145.29 ($29.96- $347.06)
Australasia	$537.01 ($145.96- $1328.16)	7.1% (1.9% - 17.5%)	$676.32 ($254.69- $1543.71)	$791.89 ($213.34- $1871.52)	$672.16 ($216.39- $1405.37)
Australia	$551.72 ($115.12- $1460.29)	7.1% (1.5% - 18.4%)	$694.82 ($229.06- $1728.71)	$786.18 ($161.44- $2011.20)	$692.33 ($181.24- $1526.23)
New Zealand	$451.49 ($97.21- $1174.38)	7.6% (1.7% - 19.2%)	$569.23 ($202.44- $1330.29)	$827.27 ($177.98- $2141.67)	$554.66 ($149.32- $1224.90)
High-income Asia Pacific	$1801.66 ($550.04- $4033.88)	31.8% (9.9% - 71.6%)	$2175.93 ($845.01- $4554.14)	$2286.15 ($675.59- $5250.99)	$1988.29 ($643.88- $4078.77)
Brunei Darussalam	$39.33 ($7.43- $106.85)	6.5% (1.2% - 18.1%)	$54.57 ($17.59- $122.21)	$69.83 ($12.27- $191.67)	$87.92 ($21.13- $184.71)
Japan	$1631.10 ($326.03- $4506.50)	27.0% (5.3% - 73.0%)	$2021.15 ($637.76- $4913.90)	$2367.48 ($497.69- $6522.80)	$1912.23 ($471.76- $4567.34)
Republic of Korea	$2360.53 ($457.72- $5898.41)	46.9% (9.2% - 115.4%)	$2742.86 ($806.46- $6491.23)	$2381.81 ($466.71- $5938.21)	$2375.12 ($486.19- $5903.22)
Singapore	$617.35 ($126.04- $1566.01)	12.3% (2.5% - 30.1%)	$762.18 ($239.87- $1771.68)	$783.43 ($153.28- $1915.59)	$670.91 ($163.06- $1577.96)
High-income North America	$689.28 ($280.50- $1202.57)	4.3% (1.8% - 7.5%)	$1068.86 ($297.23- $2619.20)	$1115.45 ($425.64- $2090.78)	$2172.15 ($1001.24- $3426.03)
Canada	$363.85 ($91.25- $902.46)	5.1% (1.3% - 12.5%)	$469.80 ($172.92- $1045.51)	$599.39 ($144.94- $1522.62)	$664.98 ($188.51- $1358.37)
Greenland	$498.65 ($123.44- $1187.46)	6.8% (1.7% - 16.0%)	$665.85 ($255.71- $1352.86)	$643.13 ($151.43- $1538.62)	$884.36 ($261.88- $1792.08)
United States of America	$728.39 ($278.32- $1289.22)	4.3% (1.6% - 7.6%)	$1140.90 ($278.32- $2877.20)	$1177.49 ($421.40- $2268.71)	$2353.44 ($1060.86- $3772.90)
Southern Latin America	$65.59 ($23.11- $134.98)	4.2% (1.5% - 8.9%)	$86.46 ($38.47- $169.57)	$79.22 ($29.43- $167.30)	$111.31 ($45.72- $199.03)
Argentina	$40.28 ($9.38- $100.90)	3.2% (0.7% - 7.9%)	$55.91 ($20.55- $116.05)	$50.27 ($11.57- $121.31)	$80.50 ($22.67- $171.84)
Chile	$131.05 ($26.05- $338.76)	5.9% (1.2% - 15.5%)	$165.60 ($54.82- $381.13)	$151.64 ($31.15- $395.21)	$188.26 ($48.68- $418.40)
Uruguay	$75.79 ($14.43- $205.31)	3.3% (0.6% - 9.2%)	$98.49 ($33.24- $229.89)	$104.97 ($19.22- $272.27)	$139.86 ($33.45- $297.89)
Western Europe	$502.13 ($247.63- $850.80)	8.9% (4.3% - 15.1%)	$682.45 ($350.66- $1066.89)	$776.13 ($354.52- $1350.87)	$792.38 ($405.27- $1282.55)
Andorra	$501.09 ($86.89- $1428.39)	16.5% (2.8% - 48.5%)	$701.91 ($224.24- $1723.63)	$814.69 ($151.02- $2301.65)	$871.97 ($199.25- $1995.05)
Austria	$612.32 ($120.67- $1766.16)	9.3% (1.8% - 26.9%)	$814.57 ($270.34- $2017.11)	$1038.16 ($201.64- $3019.46)	$987.53 ($256.56- $2070.33)
Belgium	$498.99 ($105.21- $1263.26)	8.0% (1.7% - 20.8%)	$653.34 ($211.17- $1500.30)	$803.57 ($156.39- $2062.88)	$721.99 ($194.69- $1524.76)
Cyprus	$239.91 ($40.13- $739.36)	15.0% (2.5% - 44.7%)	$314.72 ($91.94- $801.25)	$357.47 ($57.74- $1010.34)	$387.79 ($87.84- $867.91)
Denmark	$568.21 ($102.14- $1546.82)	7.2% (1.3% - 19.4%)	$751.81 ($216.21- $1835.54)	$769.30 ($145.50- $2139.50)	$860.48 ($225.87- $1818.18)
Finland	$449.44 ($105.69- $1154.26)	8.3% (2.0% - 20.7%)	$602.94 ($207.26- $1404.22)	$781.54 ($163.48- $2084.94)	$662.47 ($181.98- $1387.61)
France	$439.86 ($93.25- $1076.85)	7.3% (1.5% - 18.1%)	$596.69 ($208.51- $1293.45)	$661.81 ($131.66- $1722.87)	$732.52 ($197.89- $1553.09)
Germany	$678.39 ($145.47- $1839.86)	9.3% (2.0% - 25.1%)	$911.66 ($292.57- $2102.78)	$1027.38 ($201.18- $2852.55)	$1039.15 ($280.78- $2362.04)
Greece	$208.21 ($39.17- $574.05)	13.2% (2.5% - 35.5%)	$284.25 ($84.89- $738.38)	$353.90 ($65.05- $973.56)	$387.02 ($100.94- $899.29)

Iceland	$502.85<br>($85.00- $1508.58)	6.7%<br>(1.1% - 19.4%)	$669.76<br>($204.49- $1789.91)	$785.02<br>($128.52- $2227.70)	$799.55<br>($177.86- $1880.64)
Ireland	$877.84<br>($193.78- $2304.84)	10.8%<br>(2.4% - 28.1%)	$1030.27<br>($300.77- $2571.75)	$1299.49<br>($285.90- $3310.66)	$1021.03<br>($277.64- $2384.08)
Israel	$130.90<br>($24.71- $319.93)	3.2%<br>(0.6% - 8.2%)	$175.90<br>($64.12- $378.64)	$176.69<br>($30.63- $468.18)	$250.06<br>($56.27- $511.11)
Italy	$469.95<br>($97.19- $1209.26)	15.5%<br>(3.2% - 41.0%)	$672.56<br>($256.45- $1394.59)	$672.68<br>($128.89- $1732.91)	$834.60<br>($203.84- $1850.73)
Luxembourg	$404.97<br>($76.50- $1234.31)	5.9%<br>(1.1% - 18.1%)	$554.34<br>($175.05- $1343.95)	$642.33<br>($118.78- $1915.31)	$720.46<br>($173.74- $1644.80)
Malta	$650.20<br>($130.34- $1705.44)	13.9%<br>(2.8% - 36.2%)	$787.10<br>($247.12- $1906.86)	$992.80<br>($197.55- $2723.39)	$724.61<br>($177.52- $1755.66)
Netherlands	$766.45<br>($174.56- $1894.56)	10.3%<br>(2.3% - 24.9%)	$1012.11<br>($373.36- $2271.42)	$1086.95<br>($220.55- $2815.60)	$1061.02<br>($275.34- $2190.61)
Norway	$625.26<br>($131.35- $1614.83)	7.0%<br>(1.4% - 18.3%)	$829.13<br>($245.41- $2002.70)	$861.17<br>($166.25- $2355.09)	$943.80<br>($256.85- $1910.94)
Portugal	$202.79<br>($42.86- $585.88)	8.8%<br>(1.9% - 25.8%)	$277.08<br>($98.95- $664.41)	$290.08<br>($57.31- $853.14)	$447.27<br>($113.63- $992.01)
Spain	$499.87<br>($106.18- $1325.62)	15.7%<br>(3.3% - 42.7%)	$659.80<br>($231.44- $1522.74)	$751.53<br>($158.77- $2017.40)	$703.29<br>($173.04- $1534.75)
Sweden	$578.38<br>($108.32- $1736.10)	7.5%<br>(1.5% - 22.3%)	$795.15<br>($256.40- $1985.04)	$763.79<br>($137.59- $2382.26)	$939.76<br>($243.94- $2324.12)
Switzerland	$433.33<br>($83.50- $1313.66)	3.2%<br>(0.6% - 9.6%)	$676.77<br>($236.77- $1498.01)	$591.49<br>($104.44- $1787.25)	$848.88<br>($183.03- $2175.93)
United Kingdom	$419.07<br>($86.19- $1111.79)	8.2%<br>(1.7% - 22.5%)	$586.94<br>($192.25- $1310.97)	$794.99<br>($150.11- $2207.04)	$668.09<br>($180.92- $1425.51)
Andean Latin America	$45.38<br>($15.45- $101.81)	9.9%<br>(3.4% - 22.6%)	$60.23<br>($25.14- $120.13)	$53.40<br>($17.83- $118.63)	$55.51<br>($20.76- $114.27)
Bolivia (Plurinational State of)	$15.37<br>($2.69- $43.66)	4.7%<br>(0.8% - 13.0%)	$20.46<br>($6.81- $48.37)	$18.68<br>($3.51- $50.69)	$19.00<br>($4.34- $49.03)
Ecuador	$67.74<br>($11.47- $195.30)	9.6%<br>(1.7% - 27.5%)	$86.68<br>($25.38- $213.68)	$79.11<br>($13.85- $225.28)	$74.53<br>($15.82- $195.37)
Peru	$47.17<br>($7.96- $131.92)	11.7%<br>(2.0% - 34.9%)	$63.98<br>($20.47- $153.83)	$55.55<br>($9.38- $152.01)	$61.26<br>($13.64- $150.80)
Caribbean	$109.38<br>($40.32- $241.35)	13.6%<br>(5.0% - 30.1%)	$139.71<br>($62.67- $269.32)	$134.84<br>($48.69- $301.14)	$127.03<br>($50.65- $262.60)
Antigua and Barbuda	$94.63<br>($14.92- $263.08)	7.0%<br>(1.2% - 19.0%)	$138.56<br>($42.91- $316.59)	$113.92<br>($17.97- $306.11)	$156.59<br>($34.66- $373.98)
Bahamas	$139.14<br>($22.90- $424.03)	4.4%<br>(0.7% - 13.4%)	$198.91<br>($62.12- $491.19)	$195.00<br>($32.34- $589.15)	$208.88<br>($46.61- $505.78)
Barbados	$80.20<br>($13.94- $232.79)	7.3%<br>(1.3% - 21.3%)	$117.91<br>($37.05- $283.03)	$113.70<br>($19.28- $328.13)	$153.04<br>($34.73- $354.75)
Belize	$20.69<br>($4.04- $58.97)	4.9%<br>(0.9% - 14.6%)	$29.05<br>($9.95- $71.87)	$24.47<br>($4.74- $69.61)	$31.30<br>($7.04- $76.12)
Bermuda	$981.60<br>($173.71- $2577.46)	10.1%<br>(1.7% - 26.2%)	$1338.20<br>($416.65- $2913.72)	$1224.26<br>($218.91- $3037.66)	$1300.05<br>($298.55- $2904.33)
Cuba	$317.78<br>($66.39- $885.00)	18.4%<br>(3.7% - 51.1%)	$389.29<br>($125.04- $959.90)	$400.11<br>($81.73- $1168.41)	$328.48<br>($69.79- $896.80)
Dominica	$38.64<br>($7.12- $103.71)	8.6%<br>(1.5% - 22.4%)	$54.58<br>($16.72- $121.26)	$48.84<br>($9.32- $130.85)	$71.87<br>($16.54- $168.18)
Dominican Republic	$53.17<br>($9.74- $152.27)	6.9%<br>(1.3% - 20.6%)	$68.91<br>($22.16- $159.96)	$57.25<br>($10.69- $158.20)	$61.00<br>($13.22- $160.75)
Grenada	$45.44<br>($7.91- $128.99)	7.6%<br>(1.3% - 21.3%)	$64.54<br>($20.18- $157.64)	$51.54<br>($9.46- $142.36)	$80.04<br>($17.58- $196.26)
Guyana	$36.47<br>($7.18- $106.53)	7.0%<br>(1.4% - 20.0%)	$47.35<br>($14.78- $117.31)	$42.23<br>($8.59- $118.25)	$41.80<br>($9.40- $109.05)
Haiti	$1.46<br>($0.29- $4.08)	2.5%<br>(0.5% - 6.9%)	$2.14<br>($0.74- $4.87)	$1.84<br>($0.36- $4.95)	$3.01<br>($0.67- $7.27)
Jamaica	$38.25<br>($6.81- $106.10)	7.7%<br>(1.4% - 21.0%)	$53.58<br>($16.73- $128.62)	$49.29<br>($8.57- $140.04)	$64.17<br>($13.52- $152.29)
Puerto Rico	$324.59<br>($62.38- $936.38)	20.6%<br>(3.8% - 61.6%)	$444.98<br>($141.71- $1159.24)	$399.26<br>($74.43- $1162.94)	$463.14<br>($112.30- $1081.67)
Saint Lucia	$44.79<br>($7.80- $133.99)	6.7%<br>(1.2% - 20.5%)	$65.61<br>($22.52- $160.88)	$53.87<br>($9.66- $164.75)	$84.81<br>($17.86- $202.06)
Saint Vincent and the Grenadines	$44.29<br>($7.86- $133.99)	10.3%<br>(1.9% - 30.3%)	$61.98<br>($20.26- $161.09)	$53.49<br>($9.15- $161.54)	$66.99<br>($14.55- $162.87)
Suriname	$46.57<br>($8.20- $133.49)	6.2%<br>(1.1% - 18.0%)	$63.22<br>($20.11- $155.74)	$55.17<br>($9.94- $157.76)	$58.02<br>($12.55- $145.30)

Trinidad and Tobago	$202.25<br>($39.70- $578.66)	14.7%<br>(2.8% - 41.9%)	$268.65<br>($81.70- $667.32)	$254.69<br>($49.81- $735.25)	$236.71<br>($52.93- $600.64)
United States Virgin Islands	$143.47<br>($23.85- $439.58)	13.0%<br>(2.1% - 39.1%)	$193.16<br>($53.85- $500.31)	$196.84<br>($32.30- $600.91)	$190.76<br>($43.63- $476.81)
Central Latin America	$31.96<br>($12.89- $66.79)	5.2%<br>(2.1% - 11.0%)	$44.29<br>($21.51- $80.40)	$37.68<br>($15.29- $78.23)	$46.68<br>($20.38- $86.90)
Colombia	$69.06<br>($14.51- $184.98)	8.2%<br>(1.7% - 22.6%)	$94.73<br>($30.56- $220.76)	$77.20<br>($15.55- $204.92)	$88.34<br>($21.43- $215.16)
Costa Rica	$115.03<br>($21.85- $317.12)	7.1%<br>(1.4% - 19.5%)	$156.30<br>($53.07- $354.12)	$141.38<br>($28.02- $395.70)	$142.82<br>($32.24- $354.95)
El Salvador	$20.78<br>($3.51- $60.31)	4.2%<br>(0.7% - 12.1%)	$29.84<br>($9.80- $72.00)	$22.97<br>($4.03- $63.41)	$39.49<br>($8.70- $95.10)
Guatemala	$15.64<br>($2.78- $49.43)	3.4%<br>(0.6% - 10.6%)	$22.66<br>($7.59- $59.94)	$16.33<br>($3.04- $51.06)	$22.99<br>($4.77- $58.32)
Honduras	$7.62<br>($1.39- $20.85)	2.8%<br>(0.5% - 8.2%)	$10.61<br>($3.43- $24.75)	$8.57<br>($1.59- $24.38)	$11.61<br>($2.62- $28.13)
Mexico	$25.44<br>($4.84- $71.63)	3.9%<br>(0.8% - 10.9%)	$35.62<br>($12.13- $83.41)	$31.59<br>($6.09- $86.33)	$41.21<br>($9.92- $100.02)
Nicaragua	$18.48<br>($3.30- $50.86)	7.5%<br>(1.3% - 20.3%)	$24.64<br>($7.98- $56.86)	$21.45<br>($4.04- $58.60)	$24.03<br>($4.89- $59.17)
Panama	$108.48<br>($20.72- $297.49)	6.5%<br>(1.2% - 18.0%)	$144.60<br>($47.89- $332.27)	$129.41<br>($23.99- $346.20)	$132.03<br>($31.22- $320.41)
Venezuela (Bolivarian Republic of)	$1.71<br>($0.30- $4.60)	2.5%<br>(0.4% - 6.7%)	$2.99<br>($1.00- $6.48)	$2.61<br>($0.45- $7.04)	$11.60<br>($2.63- $26.50)
Tropical Latin America	$76.20<br>($15.01- $205.74)	5.7%<br>(1.1% - 15.0%)	$107.96<br>($35.60- $245.40)	$90.36<br>($16.59- $243.30)	$118.72<br>($26.27- $267.65)
Brazil	$77.49<br>($14.30- $212.16)	5.7%<br>(1.0% - 15.2%)	$109.95<br>($35.10- $250.86)	$91.97<br>($15.90- $248.26)	$121.24<br>($26.00- $275.19)
Paraguay	$43.61<br>($7.49- $120.98)	5.9%<br>(1.0% - 15.9%)	$57.64<br>($17.95- $132.58)	$49.76<br>($8.76- $137.58)	$55.12<br>($11.31- $134.64)
North Africa and Middle East	$64.48<br>($30.37- $116.00)	13.6%<br>(6.3% - 24.7%)	$85.14<br>($42.39- $143.70)	$65.35<br>($30.51- $117.43)	$83.40<br>($40.27- $142.75)
Afghanistan	$2.35<br>($0.45- $6.59)	4.2%<br>(0.8% - 11.8%)	$3.00<br>($1.03- $6.85)	$2.36<br>($0.46- $6.59)	$2.66<br>($0.61- $6.77)
Algeria	$55.63<br>($10.87- $146.53)	16.8%<br>(3.3% - 46.0%)	$70.18<br>($23.12- $167.48)	$58.54<br>($11.93- $157.64)	$62.75<br>($15.00- $156.47)
Bahrain	$130.46<br>($24.89- $344.65)	14.1%<br>(2.8% - 39.0%)	$184.57<br>($63.63- $426.88)	$138.31<br>($26.69- $374.76)	$225.79<br>($57.29- $508.11)
Egypt	$18.98<br>($3.49- $55.70)	8.0%<br>(1.5% - 23.1%)	$25.09<br>($8.20- $61.67)	$19.14<br>($3.60- $55.71)	$27.54<br>($6.67- $64.82)
Iran (Islamic Republic of)	$144.47<br>($26.31- $420.70)	18.2%<br>(3.1% - 52.6%)	$184.97<br>($57.80- $455.66)	$145.96<br>($26.68- $425.44)	$162.78<br>($33.61- $425.44)
Iraq	$43.19<br>($8.15- $114.80)	12.5%<br>(2.1% - 34.5%)	$55.62<br>($18.16- $133.82)	$43.19<br>($8.15- $114.80)	$51.39<br>($11.15- $124.78)
Jordan	$20.69<br>($3.75- $58.68)	4.4%<br>(0.8% - 12.2%)	$30.23<br>($9.75- $70.95)	$21.65<br>($3.90- $59.70)	$47.54<br>($11.52- $111.48)
Kuwait	$262.93<br>($47.09- $791.73)	21.1%<br>(3.6% - 63.8%)	$364.99<br>($124.02- $931.04)	$276.56<br>($51.59- $836.26)	$410.43<br>($91.18- $941.98)
Lebanon	$36.96<br>($7.29- $100.30)	6.1%<br>(1.2% - 16.3%)	$54.13<br>($17.83- $123.19)	$37.17<br>($7.48- $101.44)	$81.47<br>($21.18- $189.89)
Libya	$32.72<br>($5.52- $90.32)	8.7%<br>(1.4% - 24.0%)	$50.87<br>($16.11- $122.32)	$33.52<br>($5.64- $90.65)	$81.12<br>($17.66- $185.39)
Morocco	$38.24<br>($7.92- $104.95)	13.3%<br>(2.7% - 37.5%)	$48.75<br>($16.89- $112.26)	$39.01<br>($8.18- $106.78)	$44.51<br>($10.81- $112.29)
Oman	$73.95<br>($14.18- $204.90)	10.3%<br>(2.0% - 28.9%)	$104.79<br>($34.34- $255.81)	$73.97<br>($14.18- $204.90)	$133.88<br>($33.82- $298.11)
Palestine	$25.23<br>($5.10- $67.85)	3.5%<br>(0.7% - 9.3%)	$34.75<br>($12.72- $75.86)	$25.25<br>($5.15- $67.85)	$37.20<br>($8.90- $85.21)
Qatar	$224.64<br>($43.48- $596.51)	7.4%<br>(1.3% - 21.4%)	$316.68<br>($99.60- $726.63)	$233.29<br>($45.07- $604.56)	$351.36<br>($83.37- $780.01)
Saudi Arabia	$243.82<br>($46.46- $694.45)	10.2%<br>(2.0% - 28.2%)	$332.68<br>($104.48- $803.55)	$243.83<br>($46.46- $694.45)	$316.53<br>($76.85- $779.67)
Sudan	$6.12<br>($1.21- $19.73)	10.3%<br>(2.1% - 33.2%)	$8.11<br>($2.71- $21.95)	$6.12<br>($1.21- $19.73)	$9.80<br>($2.28- $24.26)
Syrian Arab Republic	$6.53<br>($1.16- $17.36)	15.8%<br>(2.7% - 44.6%)	$9.44<br>($3.17- $22.13)	$6.68<br>($1.19- $17.36)	$17.45<br>($4.16- $39.24)
Tunisia	$81.98<br>($16.14- $230.07)	20.7%<br>(4.0% - 58.6%)	$106.10<br>($36.48- $266.26)	$85.01<br>($17.51- $233.90)	$93.33<br>($20.58- $236.93)

Turkey	$126.28 ($22.72- $370.98)	20.9% (3.7% - 60.0%)	$165.24 ($53.35- $387.94)	$126.53 ($22.72- $370.98)	$160.95 ($35.43- $389.82)
United Arab Emirates	$226.19 ($40.21- $615.42)	18.0% (3.0% - 53.7%)	$317.07 ($102.85- $758.65)	$246.76 ($46.10- $650.95)	$385.04 ($85.32- $913.12)
Yemen	$0.93 ($0.18- $2.58)	2.1% (0.4% - 5.9%)	$1.49 ($0.55- $3.36)	$0.94 ($0.18- $2.59)	$2.87 ($0.68- $6.72)
South Asia	$8.85 ($2.00- $23.25)	6.8% (1.5% - 17.1%)	$11.00 ($3.96- $25.70)	$9.10 ($2.08- $23.34)	$10.15 ($2.65- $24.46)
Bangladesh	$10.81 ($1.86- $32.38)	14.2% (2.4% - 41.0%)	$13.59 ($4.21- $34.71)	$11.71 ($2.07- $34.35)	$11.57 ($2.21- $32.38)
Bhutan	$5.74 ($0.94- $17.43)	3.7% (0.6% - 11.2%)	$7.58 ($2.36- $18.48)	$6.02 ($1.03- $18.20)	$9.91 ($2.05- $25.06)
India	$10.05 ($1.67- $28.72)	6.8% (1.1% - 19.1%)	$12.43 ($3.73- $31.50)	$10.23 ($1.71- $29.02)	$11.51 ($2.26- $29.86)
Nepal	$6.31 ($0.98- $19.46)	6.2% (1.0% - 18.7%)	$8.02 ($2.55- $19.85)	$6.67 ($1.02- $20.42)	$7.01 ($1.45- $19.76)
Pakistan	$1.87 ($0.31- $5.36)	2.4% (0.4% - 6.8%)	$2.51 ($0.78- $5.77)	$2.04 ($0.35- $5.83)	$2.71 ($0.58- $6.93)
East Asia	$401.67 ($83.29- $1022.27)	27.9% (5.5% - 72.4%)	$452.35 ($150.05- $1039.29)	$408.51 ($83.76- $1034.05)	$417.60 ($93.75- $1041.47)
China	$408.86 ($80.97- $1047.29)	28.1% (5.5% - 73.8%)	$459.13 ($148.43- $1062.06)	$415.22 ($81.75- $1057.05)	$419.28 ($85.78- $1057.05)
Democratic People's Republic of Korea	$7.13 ($1.43- $19.40)	10.1% (2.0% - 27.2%)	$10.56 ($3.81- $22.83)	$8.61 ($1.77- $21.94)	$18.18 ($4.42- $42.46)
Taiwan (Province of China)	$410.84 ($78.21- $1147.87)	18.2% (3.6% - 51.3%)	$540.22 ($157.59- $1279.30)	$452.69 ($86.41- $1279.29)	$765.07 ($183.37- $1683.68)
Oceania	$2.52 ($1.15- $4.71)	1.7% (0.8% - 3.1%)	$3.66 ($1.97- $6.19)	$3.38 ($1.49- $6.28)	$5.26 ($2.57- $9.26)
American Samoa	$12.28 ($2.28- $33.16)	1.8% (0.3% - 5.0%)	$19.55 ($7.32- $43.03)	$16.97 ($3.18- $45.73)	$35.01 ($8.96- $81.43)
Fiji	$9.49 ($1.98- $26.43)	3.0% (0.6% - 8.3%)	$13.32 ($4.88- $30.34)	$12.45 ($2.46- $33.84)	$18.78 ($4.47- $44.93)
Guam	$62.41 ($12.12- $175.59)	5.5% (1.1% - 14.9%)	$86.94 ($28.93- $203.10)	$90.75 ($15.92- $245.26)	$126.03 ($28.71- $290.64)
Kiribati	$2.39 ($0.47- $6.69)	1.1% (0.2% - 3.1%)	$3.47 ($1.18- $7.49)	$3.02 ($0.62- $7.98)	$6.70 ($1.64- $16.02)
Marshall Islands	$9.43 ($2.21- $26.27)	1.2% (0.3% - 3.2%)	$13.97 ($4.86- $29.97)	$11.41 ($2.70- $29.84)	$18.96 ($5.30- $43.73)
Micronesia (Federated States of)	$6.00 ($1.24- $15.78)	2.5% (0.5% - 6.4%)	$8.21 ($3.10- $18.36)	$7.65 ($1.62- $19.75)	$10.90 ($2.68- $25.15)
Northern Mariana Islands	$56.67 ($11.95- $149.40)	9.7% (2.0% - 26.1%)	$77.39 ($28.50- $166.06)	$86.41 ($18.22- $231.50)	$119.85 ($28.53- $271.50)
Papua New Guinea	$1.10 ($0.22- $3.14)	1.0% (0.2% - 2.8%)	$1.67 ($0.56- $3.83)	$1.39 ($0.29- $3.80)	$2.26 ($0.56- $5.66)
Samoa	$5.70 ($1.00- $15.90)	2.0% (0.4% - 5.7%)	$8.17 ($2.77- $19.15)	$7.62 ($1.33- $21.87)	$11.23 ($2.48- $29.02)
Solomon Islands	$1.56 ($0.26- $4.34)	1.1% (0.1% - 3.4%)	$2.42 ($0.83- $5.10)	$1.92 ($0.32- $5.24)	$4.21 ($0.95- $10.32)
Tonga	$7.44 ($1.61- $18.94)	2.4% (0.5% - 6.0%)	$9.97 ($3.66- $21.01)	$9.69 ($2.15- $25.40)	$16.54 ($4.52- $38.53)
Vanuatu	$1.45 ($0.30- $4.08)	1.0% (0.2% - 3.0%)	$2.18 ($0.81- $4.97)	$1.81 ($0.40- $4.84)	$3.99 ($1.02- $9.62)
Southeast Asia	$34.41 ($15.63- $64.67)	10.9% (5.0% - 20.6%)	$42.44 ($22.42- $74.97)	$39.89 ($18.31- $76.96)	$43.65 ($21.09- $76.52)
Cambodia	$9.98 ($2.13- $26.72)	9.3% (2.0% - 25.2%)	$12.65 ($4.34- $27.91)	$10.48 ($2.30- $27.33)	$14.51 ($3.55- $34.14)
Indonesia	$25.66 ($5.16- $71.92)	9.2% (1.8% - 25.4%)	$30.78 ($10.80- $74.16)	$29.07 ($5.60- $81.72)	$31.93 ($6.90- $77.54)
Lao People's Democratic Republic	$5.69 ($1.09- $15.76)	5.9% (1.1% - 16.6%)	$7.83 ($2.81- $18.04)	$5.87 ($1.11- $16.02)	$10.91 ($2.48- $26.01)
Malaysia	$59.02 ($11.35- $159.03)	6.2% (1.1% - 16.6%)	$73.65 ($25.51- $167.05)	$71.43 ($13.18- $193.50)	$74.81 ($17.72- $175.80)
Maldives	$87.99 ($19.70- $227.44)	5.1% (1.1% - 13.6%)	$116.63 ($41.67- $257.45)	$93.02 ($21.37- $240.03)	$135.59 ($36.65- $303.56)
Mauritius	$195.26 ($40.34- $489.24)	15.1% (3.2% - 39.2%)	$230.72 ($84.28- $513.23)	$229.97 ($47.63- $610.24)	$210.10 ($50.85- $507.72)

Myanmar	$9.55 ($4.19- $17.07)	8.7% (3.9% - 15.8%)	$11.93 ($5.84- $20.58)	$10.71 ($4.57- $19.59)	$13.31 ($5.77- $23.98)
Philippines	$12.03 ($2.57- $31.10)	4.8% (1.0% - 12.8%)	$15.01 ($5.20- $33.04)	$15.14 ($3.19- $39.76)	$15.24 ($3.96- $35.29)
Seychelles	$41.84 ($8.71- $104.58)	3.8% (0.8% - 9.5%)	$59.54 ($22.31- $124.60)	$55.09 ($10.92- $137.95)	$88.61 ($21.24- $187.40)
Sri Lanka	$39.02 ($7.47- $102.04)	16.9% (3.2% - 44.3%)	$51.13 ($17.20- $108.57)	$46.40 ($8.77- $120.81)	$62.45 ($14.10- $142.43)
Thailand	$104.21 ($21.49- $298.89)	18.1% (3.6% - 50.3%)	$134.50 ($47.03- $314.20)	$128.43 ($24.97- $363.18)	$146.96 ($35.68- $332.80)
Timor-Leste	$5.50 ($1.21- $14.31)	5.5% (1.1% - 14.5%)	$6.90 ($2.51- $14.93)	$5.62 ($1.21- $14.31)	$8.85 ($2.11- $20.40)
Viet Nam	$57.82 ($10.71- $151.30)	20.9% (3.9% - 54.4%)	$68.78 ($21.28- $153.75)	$61.66 ($11.17- $163.39)	$62.29 ($12.40- $153.53)
Central Sub-Saharan Africa	$2.07 ($0.76- $4.25)	3.3% (1.2% - 6.8%)	$2.71 ($1.20- $4.93)	$2.22 ($0.80- $4.55)	$3.19 ($1.41- $5.87)
Angola	$2.61 ($0.47- $7.07)	2.0% (0.3% - 5.5%)	$3.51 ($1.12- $8.06)	$2.63 ($0.48- $7.07)	$4.77 ($1.04- $10.97)
Central African Republic	$0.60 ($0.12- $1.82)	1.6% (0.3% - 5.0%)	$0.90 ($0.31- $2.18)	$0.88 ($0.17- $2.50)	$1.25 ($0.28- $3.17)
Congo	$4.01 ($0.67- $12.05)	5.4% (0.9% - 16.5%)	$5.16 ($1.56- $13.36)	$4.51 ($0.75- $12.90)	$7.87 ($1.70- $18.76)
Democratic Republic of the Congo	$1.48 ($0.31- $3.83)	5.7% (1.2% - 14.8%)	$1.88 ($0.64- $4.20)	$1.64 ($0.35- $4.27)	$1.75 ($0.39- $4.17)
Equatorial Guinea	$12.27 ($4.13- $26.43)	2.2% (0.7% - 4.7%)	$16.17 ($6.50- $32.37)	$12.90 ($4.38- $27.82)	$22.54 ($8.57- $44.33)
Gabon	$17.01 ($2.99- $48.52)	3.6% (0.6% - 9.9%)	$23.59 ($7.21- $58.39)	$17.66 ($3.09- $49.37)	$35.00 ($7.55- $79.02)
Eastern Sub-Saharan Africa	$2.12 ($0.92- $3.99)	3.7% (1.6% - 6.8%)	$2.72 ($1.37- $4.60)	$2.34 ($1.00- $4.21)	$2.84 ($1.28- $4.90)
Burundi	$0.42 ($0.08- $1.24)	1.3% (0.2% - 3.8%)	$0.60 ($0.20- $1.50)	$0.58 ($0.11- $1.64)	$0.69 ($0.16- $1.82)
Comoros	$2.84 ($0.51- $7.44)	3.3% (0.6% - 8.7%)	$4.05 ($1.35- $9.66)	$3.45 ($0.63- $9.03)	$6.34 ($1.42- $14.34)
Djibouti	$2.38 ($0.44- $6.82)	3.8% (0.7% - 10.9%)	$3.40 ($1.18- $8.13)	$2.62 ($0.49- $7.48)	$6.21 ($1.44- $14.14)
Eritrea	$0.30 ($0.06- $0.80)	1.7% (0.3% - 4.5%)	$0.42 ($0.14- $0.95)	$0.36 ($0.07- $1.00)	$1.14 ($0.27- $2.59)
Ethiopia	$2.25 ($0.38- $6.54)	5.8% (1.0% - 17.0%)	$2.78 ($0.88- $6.96)	$2.28 ($0.40- $6.54)	$2.75 ($0.55- $6.92)
Kenya	$4.74 ($0.76- $13.38)	2.8% (0.4% - 7.8%)	$6.33 ($2.02- $15.21)	$5.75 ($0.92- $16.77)	$6.57 ($1.36- $15.69)
Madagascar	$0.44 ($0.09- $1.22)	1.7% (0.4% - 4.7%)	$0.62 ($0.23- $1.32)	$0.57 ($0.12- $1.54)	$0.96 ($0.22- $2.23)
Malawi	$2.12 ($0.47- $6.20)	4.5% (0.9% - 13.1%)	$2.63 ($0.88- $6.48)	$2.31 ($0.50- $6.46)	$2.74 ($0.71- $7.02)
Mozambique	$1.56 ($0.30- $4.38)	3.1% (0.6% - 8.5%)	$2.05 ($0.68- $4.75)	$1.69 ($0.33- $4.72)	$1.87 ($0.40- $4.75)
Rwanda	$6.59 ($1.29- $18.46)	7.0% (1.3% - 19.2%)	$7.92 ($2.44- $19.35)	$6.99 ($1.42- $19.30)	$6.79 ($1.40- $19.23)
Somalia	$0.07 ($0.01- $0.19)	0.9% (0.2% - 2.6%)	$0.10 ($0.04- $0.24)	$0.10 ($0.02- $0.26)	$0.13 ($0.03- $0.34)
South Sudan	$0.32 ($0.06- $0.89)	1.3% (0.2% - 3.7%)	$0.44 ($0.15- $0.98)	$0.43 ($0.08- $1.15)	$0.96 ($0.22- $2.31)
Uganda	$1.67 ($0.27- $4.73)	3.0% (0.5% - 8.4%)	$2.22 ($0.74- $5.17)	$1.69 ($0.27- $4.83)	$2.54 ($0.59- $6.01)
United Republic of Tanzania	$2.34 ($0.45- $6.23)	4.3% (0.8% - 12.0%)	$2.93 ($1.02- $6.80)	$2.75 ($0.54- $7.34)	$2.93 ($0.66- $6.95)
Zambia	$1.97 ($0.36- $5.48)	2.2% (0.4% - 6.1%)	$2.68 ($0.86- $6.25)	$2.02 ($0.36- $5.56)	$3.73 ($0.83- $8.56)
Southern Sub-Saharan Africa	$19.90 ($5.08- $48.78)	2.9% (0.7% - 7.2%)	$26.96 ($10.20- $55.61)	$27.84 ($6.62- $68.12)	$30.37 ($8.55- $66.14)
Botswana	$32.41 ($6.98- $91.20)	2.5% (0.5% - 7.0%)	$41.90 ($13.31- $104.86)	$38.85 ($8.18- $107.87)	$41.61 ($10.12- $99.92)
Eswatini	$8.69 ($1.62- $23.60)	2.2% (0.4% - 6.1%)	$11.20 ($3.49- $26.61)	$10.85 ($1.96- $30.15)	$11.50 ($2.63- $26.79)
Lesotho	$8.05 ($1.60- $22.44)	2.8% (0.6% - 7.7%)	$10.02 ($2.96- $24.00)	$9.52 ($1.92- $25.81)	$8.67 ($1.77- $22.97)

Namibia	$15.31 ($2.81- $40.00)	1.7% (0.3% - 4.6%)	$21.32 ($7.31- $48.20)	$19.42 ($3.69- $48.77)	$25.88 ($5.96- $58.89)
South Africa	$27.27 ($5.35- $69.38)	3.1% (0.6% - 8.2%)	$37.01 ($12.34- $78.68)	$38.54 ($7.23- $97.08)	$41.76 ($9.41- $95.02)
Zimbabwe	$0.79 ($0.15- $2.22)	0.7% (0.1% - 2.2%)	$1.15 ($0.37- $2.65)	$1.36 ($0.25- $3.72)	$1.91 ($0.42- $4.60)
Western Sub-Saharan Africa	$2.56 ($0.89- $5.86)	3.0% (1.1% - 6.7%)	$3.37 ($1.42- $6.59)	$2.73 ($1.00- $6.06)	$3.48 ($1.41- $7.01)
Benin	$0.54 ($0.11- $1.45)	1.3% (0.3% - 3.5%)	$0.77 ($0.27- $1.70)	$0.70 ($0.15- $1.95)	$1.22 ($0.31- $2.81)
Burkina Faso	$1.43 ($0.29- $4.24)	2.1% (0.4% - 5.8%)	$1.79 ($0.61- $4.34)	$1.76 ($0.34- $4.94)	$1.59 ($0.38- $4.34)
Cabo Verde	$21.12 ($4.05- $56.01)	6.7% (1.3% - 17.7%)	$27.85 ($9.54- $64.95)	$22.24 ($4.20- $58.21)	$29.38 ($6.86- $67.32)
Cameroon	$1.47 ($0.29- $3.87)	2.0% (0.4% - 5.3%)	$1.99 ($0.71- $4.41)	$1.68 ($0.32- $4.39)	$3.31 ($0.77- $7.29)
Chad	$0.21 ($0.04- $0.60)	0.6% (0.1% - 1.8%)	$0.31 ($0.12- $0.71)	$0.26 ($0.05- $0.71)	$0.46 ($0.11- $1.11)
CÃ´te d'Ivoire	$1.49 ($0.29- $4.21)	1.4% (0.3% - 4.1%)	$2.10 ($0.73- $4.98)	$1.88 ($0.36- $5.51)	$2.89 ($0.67- $6.61)
Gambia	$1.97 ($0.40- $5.66)	4.1% (0.8% - 11.8%)	$2.55 ($0.90- $6.13)	$2.23 ($0.45- $6.52)	$2.41 ($0.54- $6.07)
Ghana	$6.30 ($1.26- $16.70)	5.2% (1.0% - 13.7%)	$7.95 ($2.66- $18.39)	$6.84 ($1.38- $17.99)	$7.92 ($1.87- $18.27)
Guinea	$1.17 ($0.24- $3.29)	1.4% (0.3% - 3.9%)	$1.48 ($0.53- $3.46)	$1.42 ($0.30- $3.87)	$1.69 ($0.39- $4.22)
Guinea-Bissau	$1.14 ($0.23- $3.05)	1.6% (0.3% - 4.3%)	$1.55 ($0.51- $3.43)	$1.40 ($0.27- $3.84)	$1.83 ($0.43- $4.30)
Liberia	$5.70 ($1.12- $15.16)	7.7% (1.5% - 20.2%)	$6.52 ($2.12- $15.86)	$5.83 ($1.14- $15.31)	$5.87 ($1.19- $15.45)
Mali	$0.36 ($0.06- $1.05)	0.9% (0.2% - 2.7%)	$0.53 ($0.18- $1.19)	$0.45 ($0.08- $1.28)	$0.87 ($0.20- $2.18)
Mauritania	$2.48 ($0.49- $6.38)	2.7% (0.5% - 7.3%)	$3.43 ($1.16- $7.45)	$2.85 ($0.58- $7.38)	$4.63 ($1.17- $10.45)
Niger	$0.26 ($0.05- $0.76)	0.7% (0.1% - 2.1%)	$0.37 ($0.12- $0.85)	$0.34 ($0.06- $0.90)	$0.45 ($0.11- $1.10)
Nigeria	$3.49 ($0.63- $9.81)	3.4% (0.6% - 9.7%)	$4.63 ($1.42- $10.46)	$3.59 ($0.65- $9.95)	$4.54 ($1.00- $10.81)
Sao Tome and Principe	$3.20 ($0.59- $9.39)	2.1% (0.3% - 6.5%)	$4.58 ($1.42- $11.07)	$3.36 ($0.61- $9.66)	$7.95 ($1.83- $17.64)
Senegal	$1.98 ($0.40- $5.10)	2.3% (0.4% - 6.1%)	$2.64 ($0.85- $5.86)	$2.44 ($0.52- $6.20)	$3.03 ($0.75- $7.34)
Sierra Leone	$2.08 ($0.36- $5.44)	2.0% (0.4% - 5.5%)	$2.81 ($0.91- $6.64)	$2.36 ($0.40- $6.23)	$2.81 ($0.65- $6.50)
Togo	$2.14 ($0.43- $6.06)	3.8% (0.7% - 11.0%)	$2.77 ($0.96- $6.51)	$2.70 ($0.53- $7.29)	$2.99 ($0.74- $7.08)

Notes: All spending measured in 2019 US dollars.

Table S11: Diagnosis, treatment, and institutionalization rates by GBD region and country in 2019 and 2050 baseline and increased projections

	Diagnosis and treatment rates 2019	Diagnosis and treatment rates 2050 (Baseline)	Diagnosis and treatment rates 2050 (Accelerated)	Nursing home based care rates 2019	Nursing home based care rates 2050 (Baseline)	Nursing home based care rates 2050 (Accelerated)
Central Asia	19.6% (16.5% - 23.1%)	34.7% (21.8% - 52.2%)	43.8% (33.9% - 57.1%)	27.1% (25.1% - 29.3%)	44.3% (35.9% - 54.4%)	54.7% (48.1% - 61.8%)
Armenia	22.9% (14.5% - 34.1%)	52.3% (14.7% - 90.0%)	57.6% (29.9% - 90.0%)	26.8% (23.5% - 30.2%)	52.1% (35.9% - 72.1%)	58.7% (50.2% - 72.1%)
Azerbaijan	21.3% (13.6% - 31.5%)	46.1% (12.5% - 90.0%)	52.5% (28.6% - 90.0%)	26.2% (22.9% - 29.5%)	52.2% (35.9% - 71.3%)	57.6% (49.1% - 71.3%)
Georgia	21.4% (13.9% - 31.8%)	41.5% (12.0% - 90.0%)	50.2% (28.7% - 90.0%)	27.9% (24.5% - 31.7%)	43.0% (30.4% - 59.8%)	60.4% (52.5% - 68.9%)
Kazakhstan	20.7% (13.2% - 31.9%)	31.3% (8.4% - 79.7%)	45.5% (27.4% - 79.7%)	30.1% (26.1% - 34.3%)	49.5% (33.6% - 70.1%)	65.2% (56.4% - 75.0%)
Kyrgyzstan	15.7% (10.0% - 24.0%)	25.4% (6.3% - 74.9%)	35.2% (21.4% - 74.9%)	19.4% (17.3% - 22.1%)	27.3% (19.7% - 37.5%)	42.0% (36.7% - 47.9%)
Mongolia	18.4% (11.8% - 27.3%)	32.4% (8.5% - 90.0%)	42.2% (25.2% - 90.0%)	20.0% (17.7% - 22.4%)	32.5% (22.3% - 45.5%)	43.2% (37.7% - 49.1%)
Tajikistan	14.1% (8.8% - 21.5%)	29.1% (7.9% - 76.2%)	34.2% (18.6% - 76.2%)	15.9% (14.0% - 17.9%)	27.4% (19.2% - 37.7%)	34.5% (30.0% - 39.3%)
Turkmenistan	24.6% (15.3% - 36.9%)	45.9% (12.6% - 90.0%)	56.2% (32.4% - 90.0%)	25.2% (22.2% - 28.5%)	46.8% (33.1% - 64.3%)	54.7% (47.6% - 64.4%)
Uzbekistan	15.9% (9.8% - 24.2%)	29.7% (8.2% - 83.1%)	37.3% (21.1% - 83.1%)	22.0% (19.5% - 25.1%)	39.9% (28.0% - 57.0%)	47.8% (41.9% - 57.0%)
Central Europe	27.6% (23.2% - 33.2%)	44.9% (28.4% - 65.7%)	60.7% (48.1% - 75.3%)	38.6% (35.4% - 42.4%)	62.8% (48.3% - 76.2%)	78.4% (74.4% - 79.9%)
Albania	22.8% (14.4% - 34.7%)	44.5% (11.8% - 90.0%)	53.5% (30.0% - 90.0%)	26.0% (22.9% - 29.5%)	48.5% (33.5% - 67.9%)	56.6% (49.4% - 68.2%)
Bosnia and Herzegovina	24.8% (15.4% - 36.7%)	42.2% (11.6% - 90.0%)	55.2% (32.5% - 90.0%)	29.5% (26.1% - 33.4%)	55.4% (39.6% - 76.8%)	64.3% (55.6% - 76.8%)
Bulgaria	26.6% (16.9% - 41.0%)	47.1% (13.2% - 90.0%)	59.4% (36.0% - 90.0%)	34.5% (30.1% - 39.2%)	54.5% (36.6% - 75.1%)	74.1% (64.8% - 80.0%)
Croatia	27.8% (17.5% - 40.7%)	38.8% (10.3% - 90.0%)	59.9% (36.9% - 90.0%)	38.5% (33.9% - 43.2%)	60.1% (42.4% - 80.0%)	79.1% (72.8% - 80.0%)
Czech Republic	32.0% (19.7% - 47.7%)	48.9% (13.3% - 90.0%)	68.1% (41.9% - 90.0%)	43.2% (38.4% - 48.9%)	57.0% (40.3% - 78.5%)	80.0% (80.0% - 80.0%)
Hungary	29.4% (18.4% - 45.5%)	41.5% (11.2% - 90.0%)	62.5% (38.8% - 90.0%)	38.2% (33.3% - 43.4%)	55.6% (39.1% - 77.6%)	78.9% (71.6% - 80.0%)
Macedonia	23.6% (15.1% - 35.6%)	36.8% (10.1% - 90.0%)	52.1% (31.5% - 90.0%)	32.4% (28.6% - 36.5%)	52.9% (37.4% - 72.6%)	69.9% (60.9% - 79.6%)
Montenegro	25.8% (16.3% - 39.0%)	43.8% (12.0% - 90.0%)	57.3% (34.0% - 90.0%)	38.2% (33.6% - 42.9%)	64.3% (45.5% - 80.0%)	78.9% (72.4% - 80.0%)
Poland	27.8% (18.0% - 41.7%)	45.2% (13.0% - 90.0%)	61.0% (37.5% - 90.0%)	39.6% (34.8% - 44.8%)	65.5% (45.1% - 80.0%)	79.5% (74.9% - 80.0%)
Romania	25.5% (16.3% - 39.0%)	45.5% (11.6% - 90.0%)	57.4% (33.9% - 90.0%)	34.3% (30.2% - 38.8%)	57.4% (39.9% - 79.1%)	73.8% (64.7% - 80.0%)
Serbia	23.7% (15.1% - 36.6%)	41.3% (11.1% - 90.0%)	53.4% (31.3% - 90.0%)	35.1% (31.0% - 39.6%)	63.0% (45.4% - 80.0%)	75.3% (67.0% - 80.0%)
Slovakia	30.1% (18.4% - 45.3%)	47.7% (12.3% - 90.0%)	64.7% (38.7% - 90.0%)	40.8% (35.8% - 46.0%)	60.6% (42.3% - 80.0%)	79.8% (77.1% - 80.0%)
Slovenia	33.6% (21.1% - 51.3%)	46.0% (12.3% - 90.0%)	70.0% (44.0% - 90.0%)	45.1% (39.6% - 51.4%)	62.4% (44.2% - 80.0%)	80.0% (80.0% - 80.0%)
Eastern Europe	23.6% (16.8% - 32.6%)	36.8% (15.1% - 74.5%)	52.0% (35.0% - 78.3%)	47.1% (41.6% - 52.9%)	71.3% (53.5% - 79.8%)	79.9% (79.1% - 80.0%)
Belarus	22.5% (14.7% - 33.5%)	38.9% (11.2% - 90.0%)	50.6% (30.6% - 90.0%)	38.8% (34.1% - 43.6%)	72.2% (53.2% - 80.0%)	79.2% (73.4% - 80.0%)
Estonia	30.9% (19.1% - 46.7%)	49.8% (13.7% - 90.0%)	66.4% (40.0% - 90.0%)	53.2% (46.6% - 60.4%)	78.7% (65.6% - 80.0%)	80.0% (80.0% - 80.0%)
Latvia	28.6% (18.3% - 43.6%)	46.9% (12.2% - 90.0%)	62.4% (37.6% - 90.0%)	50.5% (44.6% - 58.1%)	76.9% (60.8% - 80.0%)	80.0% (80.0% - 80.0%)

Lithuania	29.7% (18.6% - 45.3%)	48.6% (13.6% - 90.0%)	64.2% (38.8% - 90.0%)	54.5% (47.7% - 61.4%)	79.7% (75.2% - 80.0%)	80.0% (80.0% - 80.0%)
Moldova	20.0% (12.6% - 29.4%)	38.9% (10.0% - 90.0%)	47.1% (25.6% - 90.0%)	32.8% (28.9% - 37.1%)	61.6% (42.2% - 80.0%)	71.1% (62.2% - 80.0%)
Russian Federation	24.6% (15.2% - 37.6%)	37.5% (9.2% - 90.0%)	53.8% (31.5% - 90.0%)	48.0% (41.9% - 54.1%)	72.3% (53.2% - 80.0%)	80.0% (80.0% - 80.0%)
Ukraine	19.7% (12.3% - 29.4%)	30.7% (7.6% - 84.1%)	43.7% (25.4% - 84.1%)	37.6% (32.9% - 42.7%)	57.0% (39.8% - 80.0%)	78.4% (70.9% - 80.0%)
Australasia	45.0% (30.9% - 63.5%)	61.5% (23.8% - 90.0%)	84.1% (64.7% - 90.0%)	24.1% (21.2% - 26.9%)	33.9% (23.5% - 47.1%)	52.0% (45.9% - 58.6%)
Australia	45.3% (29.0% - 66.8%)	61.6% (20.2% - 90.0%)	84.4% (61.3% - 90.0%)	24.0% (21.1% - 26.9%)	34.2% (23.5% - 47.7%)	51.9% (45.5% - 58.6%)
New Zealand	43.3% (26.3% - 67.0%)	61.2% (18.9% - 90.0%)	82.4% (56.3% - 90.0%)	25.7% (22.4% - 28.9%)	27.1% (18.9% - 37.2%)	55.5% (48.7% - 62.6%)
High-income Asia Pacific	42.8% (29.0% - 61.8%)	63.0% (28.2% - 89.9%)	81.4% (62.0% - 90.0%)	41.7% (37.0% - 47.1%)	56.6% (42.6% - 73.9%)	79.9% (78.6% - 80.0%)
Brunei	27.8% (17.7% - 42.0%)	32.8% (8.5% - 90.0%)	58.9% (36.9% - 90.0%)	29.7% (26.2% - 33.6%)	34.1% (24.5% - 47.4%)	64.1% (56.4% - 73.3%)
Japan	43.6% (27.7% - 66.3%)	62.3% (19.0% - 90.0%)	83.0% (58.6% - 90.0%)	41.3% (36.5% - 46.8%)	52.8% (37.8% - 73.0%)	79.9% (78.5% - 80.0%)
Singapore	38.8% (23.3% - 57.8%)	60.6% (18.1% - 90.0%)	78.2% (48.7% - 90.0%)	22.3% (19.8% - 25.1%)	36.6% (26.2% - 50.7%)	48.2% (42.2% - 54.8%)
South Korea	37.4% (23.0% - 55.4%)	65.2% (20.0% - 90.0%)	77.5% (48.2% - 90.0%)	60.1% (52.9% - 67.8%)	79.1% (69.5% - 80.0%)	80.0% (80.0% - 80.0%)
High-income North America	47.4% (30.9% - 69.1%)	58.4% (21.0% - 90.0%)	85.1% (63.9% - 90.0%)	34.2% (30.5% - 38.6%)	43.5% (30.7% - 60.1%)	73.5% (65.3% - 79.7%)
Canada	40.8% (26.0% - 61.9%)	53.2% (15.2% - 90.0%)	80.2% (53.0% - 90.0%)	20.6% (18.0% - 23.5%)	24.9% (17.4% - 34.4%)	44.5% (38.8% - 50.8%)
Greenland	43.2% (26.9% - 64.3%)	53.9% (14.3% - 90.0%)	82.3% (56.3% - 90.0%)	22.6% (19.9% - 25.5%)	33.4% (23.3% - 46.0%)	48.8% (42.9% - 55.3%)
United States	48.2% (29.9% - 72.1%)	59.1% (17.3% - 90.0%)	85.7% (61.3% - 90.0%)	34.4% (30.6% - 38.8%)	43.8% (30.8% - 60.7%)	74.0% (65.8% - 80.0%)
Southern Latin America	30.2% (22.2% - 40.1%)	43.3% (19.8% - 77.1%)	64.6% (46.7% - 85.0%)	25.2% (22.5% - 27.9%)	40.5% (30.5% - 53.0%)	54.9% (49.3% - 61.1%)
Argentina	28.6% (17.9% - 42.1%)	38.2% (10.8% - 90.0%)	61.1% (36.8% - 90.0%)	25.0% (21.8% - 28.3%)	37.4% (26.5% - 52.2%)	53.9% (47.0% - 61.4%)
Chile	32.9% (20.7% - 49.7%)	51.5% (14.4% - 90.0%)	69.9% (43.3% - 90.0%)	26.4% (23.3% - 29.8%)	47.4% (33.5% - 64.7%)	57.2% (50.0% - 66.3%)
Uruguay	33.7% (21.7% - 49.9%)	49.1% (13.2% - 90.0%)	70.6% (44.4% - 90.0%)	22.3% (19.6% - 25.3%)	31.4% (21.9% - 43.8%)	48.2% (42.0% - 54.9%)
Western Europe	43.2% (36.9% - 50.6%)	57.0% (41.0% - 73.6%)	81.9% (73.6% - 88.0%)	32.2% (30.3% - 34.6%)	39.8% (33.2% - 48.7%)	68.3% (62.9% - 73.5%)
Andorra	43.9% (27.1% - 68.6%)	54.3% (14.4% - 90.0%)	82.7% (57.3% - 90.0%)	41.9% (37.2% - 47.2%)	45.6% (31.8% - 61.9%)	79.9% (79.2% - 80.0%)
Austria	45.7% (29.0% - 68.3%)	59.5% (18.0% - 90.0%)	84.6% (60.7% - 90.0%)	35.9% (31.6% - 40.9%)	41.7% (29.2% - 57.5%)	76.4% (68.4% - 80.0%)
Belgium	44.9% (28.3% - 68.1%)	61.3% (19.1% - 90.0%)	83.7% (59.6% - 90.0%)	36.2% (32.0% - 40.9%)	44.3% (30.1% - 61.3%)	76.9% (68.7% - 80.0%)
Cyprus	32.6% (20.3% - 49.6%)	46.3% (12.6% - 90.0%)	68.3% (42.4% - 90.0%)	35.0% (30.8% - 39.9%)	47.6% (33.5% - 65.6%)	75.0% (65.9% - 80.0%)
Denmark	47.1% (30.0% - 71.4%)	61.3% (19.0% - 90.0%)	85.5% (62.3% - 90.0%)	43.8% (38.4% - 49.3%)	56.4% (39.2% - 77.4%)	80.0% (80.0% - 80.0%)
Finland	52.7% (33.6% - 77.1%)	62.5% (19.6% - 90.0%)	88.2% (70.0% - 90.0%)	32.0% (28.4% - 35.9%)	36.3% (25.8% - 49.8%)	69.2% (61.0% - 77.9%)
France	44.4% (27.7% - 68.0%)	58.2% (17.5% - 90.0%)	83.5% (58.8% - 90.0%)	25.1% (22.0% - 28.4%)	31.1% (21.8% - 43.3%)	54.4% (47.3% - 62.0%)
Germany	45.4% (28.7% - 68.7%)	59.6% (18.0% - 90.0%)	84.2% (59.1% - 90.0%)	36.6% (32.6% - 41.3%)	48.7% (35.1% - 65.7%)	77.5% (69.8% - 80.0%)
Greece	34.6% (21.7% - 53.7%)	46.4% (13.7% - 90.0%)	71.7% (46.0% - 90.0%)	29.6% (26.0% - 33.6%)	34.4% (24.6% - 48.1%)	64.1% (56.4% - 73.1%)
Iceland	46.3% (28.5% - 69.4%)	59.5% (16.4% - 90.0%)	84.8% (60.6% - 90.0%)	38.7% (34.1% - 43.5%)	47.5% (33.4% - 65.4%)	79.1% (73.2% - 80.0%)
Ireland	46.1% (28.9% - 68.5%)	67.1% (19.6% - 90.0%)	84.8% (58.9% - 90.0%)	38.2% (33.5% - 43.3%)	52.0% (36.0% - 72.3%)	78.9% (72.5% - 80.0%)
Israel	40.3% (25.4% - 61.0%)	55.9% (15.9% - 90.0%)	79.7% (53.9% - 90.0%)	44.4% (39.2% - 50.5%)	46.1% (32.6% - 63.7%)	80.0% (80.0% - 80.0%)
Italy	39.8% (25.7% - 60.6%)	52.2% (14.5% - 90.0%)	79.0% (54.2% - 90.0%)	29.1% (25.6% - 32.9%)	36.3% (25.3% - 50.6%)	62.8% (55.0% - 71.5%)

Luxembourg	47.3% (30.3% - 71.7%)	57.8% (16.8% - 90.0%)	85.7% (63.8% - 90.0%)	42.0% (36.9% - 47.7%)	47.0% (33.2% - 65.1%)	79.9% (79.6% - 80.0%)
Malta	40.4% (25.1% - 61.3%)	64.4% (19.4% - 90.0%)	80.1% (53.4% - 90.0%)	30.4% (26.8% - 34.6%)	40.1% (29.1% - 56.0%)	65.7% (57.4% - 74.7%)
Netherlands	45.8% (28.6% - 68.7%)	62.1% (18.3% - 90.0%)	84.6% (59.5% - 90.0%)	40.5% (35.4% - 45.7%)	46.5% (32.3% - 64.5%)	79.7% (76.3% - 80.0%)
Norway	50.6% (31.6% - 77.2%)	63.9% (19.4% - 90.0%)	87.2% (66.0% - 90.0%)	41.2% (36.5% - 46.6%)	55.7% (39.5% - 77.4%)	79.9% (78.4% - 80.0%)
Portugal	35.8% (22.5% - 53.0%)	49.0% (12.7% - 90.0%)	73.8% (48.0% - 90.0%)	25.0% (21.9% - 28.1%)	33.1% (23.4% - 45.2%)	54.0% (47.1% - 61.4%)
Spain	38.9% (24.4% - 58.4%)	55.8% (14.8% - 90.0%)	78.3% (51.4% - 90.0%)	30.3% (26.7% - 34.2%)	38.9% (27.4% - 53.7%)	65.6% (57.4% - 74.4%)
Sweden	32.4% (21.2% - 47.9%)	47.2% (14.4% - 90.0%)	68.4% (43.9% - 90.0%)	43.4% (38.4% - 48.7%)	56.6% (39.6% - 77.3%)	80.0% (80.0% - 80.0%)
Switzerland	33.0% (21.4% - 49.6%)	42.1% (11.7% - 90.0%)	69.1% (45.1% - 90.0%)	47.5% (41.4% - 53.9%)	50.9% (35.8% - 71.5%)	80.0% (80.0% - 80.0%)
United Kingdom	50.7% (35.7% - 70.4%)	62.3% (20.7% - 90.0%)	88.4% (73.9% - 90.0%)	35.8% (31.7% - 40.4%)	35.4% (25.0% - 48.6%)	76.4% (68.1% - 80.0%)
Andean Latin America	20.1% (15.0% - 26.7%)	30.3% (13.6% - 63.8%)	44.3% (30.9% - 67.7%)	17.2% (15.6% - 18.9%)	30.7% (23.2% - 39.9%)	37.2% (33.3% - 42.0%)
Bolivia	18.1% (11.3% - 27.0%)	27.4% (6.7% - 79.2%)	40.0% (23.5% - 79.2%)	13.0% (11.5% - 14.7%)	22.1% (15.2% - 30.2%)	28.2% (24.9% - 32.1%)
Ecuador	21.9% (13.9% - 33.0%)	36.6% (10.1% - 90.0%)	49.1% (29.5% - 90.0%)	16.9% (14.7% - 19.2%)	30.3% (21.3% - 42.5%)	36.6% (31.8% - 42.7%)
Peru	19.9% (12.7% - 29.8%)	28.5% (7.8% - 78.2%)	43.3% (26.7% - 78.2%)	17.4% (15.5% - 19.5%)	31.1% (22.2% - 42.2%)	37.7% (33.2% - 43.1%)
Caribbean	24.3% (19.4% - 30.9%)	37.3% (20.2% - 58.0%)	52.0% (39.5% - 66.5%)	19.1% (17.3% - 21.0%)	30.6% (23.3% - 38.3%)	39.5% (34.1% - 44.8%)
Antigua and Barbuda	23.8% (15.0% - 35.4%)	27.4% (7.6% - 77.4%)	50.8% (31.3% - 82.7%)	23.3% (20.6% - 26.2%)	40.8% (28.8% - 56.4%)	50.4% (44.4% - 57.5%)
The Bahamas	30.3% (19.5% - 46.8%)	38.1% (9.5% - 90.0%)	63.8% (40.4% - 90.0%)	27.9% (24.6% - 31.3%)	40.8% (28.7% - 55.1%)	60.3% (52.8% - 68.3%)
Barbados	26.1% (16.3% - 39.1%)	30.4% (8.2% - 87.7%)	55.5% (34.5% - 90.0%)	23.2% (20.3% - 25.9%)	33.4% (23.7% - 45.5%)	50.1% (43.6% - 56.5%)
Belize	19.0% (12.0% - 29.2%)	25.0% (6.4% - 68.3%)	41.1% (24.9% - 69.0%)	14.3% (12.7% - 16.1%)	25.3% (18.1% - 34.3%)	31.0% (27.3% - 35.6%)
Bermuda	41.2% (26.0% - 61.0%)	55.2% (15.9% - 90.0%)	80.8% (53.9% - 90.0%)	29.5% (26.1% - 33.2%)	49.2% (34.2% - 67.7%)	63.9% (55.7% - 73.3%)
Cuba	27.4% (17.4% - 41.2%)	49.0% (13.1% - 90.0%)	61.2% (36.0% - 90.0%)	18.0% (15.8% - 20.3%)	28.9% (20.0% - 39.6%)	38.8% (33.8% - 44.3%)
Dominica	21.7% (13.8% - 32.7%)	27.9% (7.0% - 75.0%)	46.8% (28.5% - 77.0%)	22.1% (19.7% - 24.9%)	36.2% (25.8% - 50.4%)	47.7% (41.9% - 53.9%)
Dominican Republic	21.4% (13.6% - 31.7%)	34.1% (8.8% - 90.0%)	47.4% (29.1% - 90.0%)	13.8% (12.2% - 15.7%)	27.7% (19.5% - 38.8%)	30.5% (26.0% - 38.8%)
Grenada	22.1% (14.0% - 35.1%)	27.8% (7.4% - 76.2%)	47.3% (28.9% - 79.6%)	18.0% (15.9% - 20.5%)	33.6% (23.5% - 47.4%)	39.2% (34.2% - 47.6%)
Guyana	19.0% (12.2% - 29.1%)	30.2% (8.3% - 76.3%)	42.1% (25.9% - 76.3%)	15.1% (13.3% - 17.3%)	27.2% (19.0% - 38.4%)	32.8% (28.5% - 38.6%)
Haiti	12.3% (7.8% - 19.1%)	14.4% (3.9% - 41.5%)	26.5% (16.3% - 43.9%)	7.9% (7.0% - 9.0%)	12.7% (8.6% - 17.6%)	17.1% (15.0% - 19.6%)
Jamaica	20.1% (12.5% - 30.9%)	25.5% (6.3% - 66.0%)	43.0% (25.8% - 69.9%)	19.0% (16.7% - 21.4%)	30.4% (21.0% - 42.4%)	41.1% (35.8% - 46.9%)
Puerto Rico	27.7% (17.4% - 42.3%)	37.5% (9.6% - 90.0%)	59.0% (36.0% - 90.0%)	29.7% (26.1% - 33.3%)	51.1% (35.5% - 69.8%)	64.2% (56.0% - 73.2%)
Saint Lucia	22.4% (14.3% - 34.1%)	26.1% (6.7% - 66.6%)	48.0% (30.3% - 74.3%)	18.1% (15.9% - 20.2%)	31.4% (22.0% - 43.0%)	39.1% (34.2% - 44.9%)
Saint Vincent and the Grenadines	19.8% (12.6% - 30.0%)	25.9% (6.5% - 67.8%)	42.9% (25.8% - 70.2%)	15.6% (13.7% - 17.7%)	26.8% (18.8% - 36.8%)	33.7% (29.4% - 38.8%)
Suriname	21.1% (13.3% - 30.9%)	29.8% (7.3% - 75.1%)	46.0% (27.8% - 76.9%)	16.1% (14.2% - 18.2%)	28.3% (19.3% - 39.9%)	34.8% (30.5% - 40.5%)
Trinidad and Tobago	26.5% (16.3% - 39.5%)	40.8% (10.8% - 90.0%)	57.8% (34.6% - 90.0%)	24.3% (21.5% - 27.4%)	40.3% (27.4% - 55.8%)	52.6% (46.3% - 59.7%)
Virgin Islands, U.S.	29.3% (18.6% - 45.0%)	41.5% (11.6% - 90.0%)	62.5% (39.2% - 90.0%)	28.2% (24.9% - 31.9%)	42.2% (29.8% - 58.6%)	61.0% (53.6% - 69.1%)
Central Latin America	21.0% (16.3% - 26.7%)	27.8% (14.0% - 50.7%)	45.3% (33.6% - 61.1%)	12.9% (11.8% - 14.2%)	22.2% (17.0% - 28.4%)	27.9% (25.1% - 31.2%)
Colombia	21.6% (14.0% - 31.7%)	31.7% (8.5% - 86.1%)	47.2% (28.6% - 86.1%)	12.5% (11.0% - 14.0%)	23.6% (16.4% - 33.3%)	27.2% (23.6% - 33.3%)

Costa Rica	25.5%<br>(16.2% - 39.4%)	37.2%<br>(9.9% - 90.0%)	55.2%<br>(34.4% - 90.0%)	14.7%<br>(12.9% - 16.7%)	24.4%<br>(16.7% - 33.5%)	31.8%<br>(27.9% - 36.3%)
El Salvador	19.7%<br>(12.5% - 29.5%)	24.3%<br>(6.5% - 67.9%)	42.3%<br>(25.7% - 68.3%)	10.1%<br>(9.0% - 11.4%)	19.4%<br>(13.3% - 26.8%)	22.2%<br>(19.3% - 26.8%)
Guatemala	18.7%<br>(12.2% - 27.9%)	23.3%<br>(5.9% - 61.8%)	40.2%<br>(25.4% - 64.6%)	8.7%<br>(7.6% - 9.8%)	18.4%<br>(12.8% - 25.7%)	19.5%<br>(16.3% - 25.7%)
Honduras	17.3%<br>(11.0% - 26.4%)	24.0%<br>(6.4% - 65.4%)	37.6%<br>(22.7% - 66.3%)	7.8%<br>(6.9% - 8.8%)	14.4%<br>(10.2% - 19.9%)	17.0%<br>(14.8% - 19.9%)
Mexico	22.6%<br>(14.1% - 34.2%)	30.2%<br>(8.3% - 86.1%)	48.6%<br>(29.4% - 86.1%)	13.2%<br>(11.8% - 15.0%)	21.7%<br>(15.0% - 30.1%)	28.6%<br>(25.1% - 32.5%)
Nicaragua	16.8%<br>(10.5% - 25.3%)	25.5%<br>(6.5% - 71.3%)	37.2%<br>(21.9% - 71.3%)	8.4%<br>(7.4% - 9.4%)	14.7%<br>(10.2% - 20.1%)	18.1%<br>(15.9% - 20.8%)
Panama	26.8%<br>(17.2% - 40.9%)	41.3%<br>(11.7% - 90.0%)	58.4%<br>(35.6% - 90.0%)	15.0%<br>(13.3% - 17.1%)	25.8%<br>(18.6% - 36.8%)	32.5%<br>(28.6% - 37.9%)
Venezuela	14.4%<br>(9.0% - 21.9%)	9.2%<br>(2.1% - 25.8%)	30.5%<br>(18.8% - 46.5%)	11.3%<br>(10.0% - 12.8%)	15.0%<br>(10.7% - 20.5%)	24.5%<br>(21.5% - 27.9%)
Tropical Latin America	23.8%<br>(15.8% - 34.4%)	31.4%<br>(8.8% - 84.5%)	51.4%<br>(33.3% - 85.4%)	15.3%<br>(13.6% - 17.3%)	27.0%<br>(18.8% - 36.7%)	33.2%<br>(29.3% - 38.3%)
Brazil	23.9%<br>(15.7% - 34.7%)	31.4%<br>(8.4% - 85.3%)	51.5%<br>(33.2% - 86.1%)	15.3%<br>(13.6% - 17.3%)	27.0%<br>(18.8% - 36.7%)	33.2%<br>(29.3% - 38.3%)
Paraguay	20.4%<br>(12.4% - 30.5%)	31.3%<br>(7.7% - 88.2%)	45.0%<br>(26.2% - 88.6%)	15.3%<br>(13.4% - 17.2%)	28.1%<br>(19.6% - 38.7%)	33.2%<br>(29.0% - 38.9%)
North Africa and Middle East	20.7%<br>(17.3% - 25.0%)	32.0%<br>(20.5% - 46.7%)	46.6%<br>(37.2% - 57.8%)	13.1%<br>(12.0% - 14.4%)	34.3%<br>(27.6% - 43.2%)	34.6%<br>(28.5% - 43.3%)
Afghanistan	13.5%<br>(8.6% - 20.1%)	22.9%<br>(5.7% - 60.6%)	30.8%<br>(18.3% - 60.6%)	3.8%<br>(3.3% - 4.2%)	9.1%<br>(6.4% - 12.7%)	9.2%<br>(7.0% - 12.7%)
Algeria	20.5%<br>(12.9% - 30.9%)	34.9%<br>(9.8% - 90.0%)	46.2%<br>(27.0% - 90.0%)	10.9%<br>(9.6% - 12.3%)	22.6%<br>(15.9% - 30.8%)	24.3%<br>(20.6% - 30.8%)
Bahrain	29.3%<br>(18.7% - 44.0%)	38.3%<br>(10.3% - 90.0%)	62.2%<br>(39.2% - 90.0%)	15.3%<br>(13.4% - 17.2%)	31.4%<br>(22.0% - 42.8%)	34.0%<br>(28.7% - 42.8%)
Egypt	17.9%<br>(11.2% - 26.7%)	25.4%<br>(6.8% - 67.3%)	39.1%<br>(23.5% - 67.6%)	11.1%<br>(9.8% - 12.5%)	27.0%<br>(18.7% - 36.1%)	27.3%<br>(21.2% - 36.1%)
Iran	23.5%<br>(14.8% - 34.8%)	39.0%<br>(9.2% - 90.0%)	52.4%<br>(30.7% - 90.0%)	11.6%<br>(10.2% - 13.1%)	28.1%<br>(19.6% - 39.9%)	28.5%<br>(22.1% - 39.9%)
Iraq	19.2%<br>(11.6% - 29.0%)	31.0%<br>(7.4% - 85.2%)	43.1%<br>(24.9% - 85.2%)	11.6%<br>(10.2% - 13.2%)	38.0%<br>(26.9% - 51.7%)	38.0%<br>(26.9% - 51.7%)
Jordan	21.1%<br>(13.4% - 32.0%)	23.4%<br>(6.0% - 61.6%)	44.9%<br>(27.7% - 71.1%)	14.3%<br>(12.6% - 16.2%)	30.7%<br>(21.4% - 42.4%)	32.4%<br>(27.1% - 42.4%)
Kuwait	31.4%<br>(20.0% - 47.0%)	40.9%<br>(11.2% - 90.0%)	66.2%<br>(41.8% - 90.0%)	21.8%<br>(19.3% - 24.6%)	45.9%<br>(31.8% - 63.6%)	48.9%<br>(41.3% - 63.6%)
Lebanon	27.8%<br>(17.7% - 41.7%)	33.5%<br>(8.9% - 88.0%)	59.2%<br>(36.8% - 90.0%)	13.2%<br>(11.6% - 14.9%)	32.8%<br>(23.5% - 45.1%)	33.1%<br>(25.2% - 45.1%)
Libya	23.3%<br>(14.5% - 36.0%)	23.9%<br>(6.3% - 68.1%)	49.4%<br>(31.0% - 81.3%)	13.3%<br>(11.7% - 15.1%)	30.2%<br>(20.9% - 42.5%)	31.1%<br>(25.2% - 42.5%)
Morocco	18.7%<br>(11.9% - 27.5%)	31.4%<br>(8.2% - 81.4%)	42.0%<br>(25.0% - 81.4%)	7.6%<br>(6.7% - 8.6%)	17.1%<br>(12.2% - 23.8%)	17.7%<br>(14.5% - 23.8%)
Oman	24.9%<br>(16.0% - 36.9%)	29.9%<br>(8.6% - 84.5%)	53.2%<br>(34.1% - 89.8%)	17.1%<br>(15.2% - 19.2%)	52.2%<br>(36.6% - 71.0%)	52.2%<br>(36.6% - 71.0%)
Palestine	22.2%<br>(14.2% - 34.3%)	30.5%<br>(8.2% - 83.7%)	47.9%<br>(29.5% - 86.1%)	8.7%<br>(7.7% - 9.9%)	24.6%<br>(17.0% - 33.8%)	24.6%<br>(17.4% - 33.8%)
Qatar	33.3%<br>(21.9% - 49.5%)	42.1%<br>(11.7% - 90.0%)	69.7%<br>(46.3% - 90.0%)	20.2%<br>(17.6% - 23.0%)	44.2%<br>(30.4% - 61.6%)	46.3%<br>(37.9% - 61.6%)
Saudi Arabia	30.1%<br>(19.3% - 45.5%)	41.0%<br>(11.3% - 90.0%)	63.8%<br>(39.9% - 90.0%)	18.6%<br>(16.4% - 21.1%)	59.5%<br>(42.6% - 80.0%)	59.5%<br>(42.6% - 80.0%)
Sudan	13.8%<br>(8.9% - 20.9%)	20.1%<br>(5.1% - 55.9%)	30.4%<br>(18.3% - 55.9%)	6.8%<br>(6.0% - 7.8%)	22.1%<br>(15.2% - 31.3%)	22.1%<br>(15.2% - 31.3%)
Syria	14.1%<br>(8.8% - 20.8%)	15.4%<br>(3.8% - 41.2%)	30.0%<br>(18.1% - 47.1%)	9.7%<br>(8.5% - 11.0%)	22.1%<br>(15.3% - 30.9%)	22.8%<br>(18.4% - 30.9%)
Tunisia	20.1%<br>(12.5% - 30.7%)	32.6%<br>(8.1% - 90.0%)	44.7%<br>(26.1% - 90.0%)	11.7%<br>(10.3% - 13.3%)	25.3%<br>(17.7% - 34.4%)	26.5%<br>(22.3% - 34.4%)
Turkey	22.1%<br>(13.5% - 33.5%)	33.8%<br>(7.9% - 90.0%)	48.4%<br>(28.5% - 90.0%)	15.2%<br>(13.4% - 17.1%)	40.6%<br>(28.1% - 56.2%)	40.7%<br>(29.4% - 56.2%)
United Arab Emirates	32.1%<br>(20.7% - 49.4%)	40.0%<br>(10.4% - 90.0%)	67.4%<br>(43.2% - 90.0%)	24.1%<br>(21.2% - 27.2%)	47.7%<br>(33.8% - 66.5%)	53.0%<br>(45.3% - 66.5%)
Yemen	13.8%<br>(8.7% - 21.1%)	13.8%<br>(3.7% - 39.9%)	29.3%<br>(18.3% - 45.7%)	4.8%<br>(4.2% - 5.4%)	11.2%<br>(7.7% - 15.3%)	11.4%<br>(9.0% - 15.3%)
South Asia	11.0%<br>(7.8% - 15.4%)	18.3%<br>(6.7% - 42.7%)	24.8%<br>(16.2% - 44.0%)	12.5%<br>(11.0% - 14.1%)	28.7%<br>(20.6% - 39.3%)	29.4%<br>(23.6% - 39.5%)

Bangladesh	9.9% (6.4% - 14.6%)	17.0% (4.3% - 44.2%)	22.6% (13.2% - 44.2%)	9.5% (8.3% - 10.8%)	19.0% (13.2% - 27.2%)	21.0% (17.8% - 27.2%)
Bhutan	11.8% (7.4% - 18.5%)	17.0% (4.3% - 49.9%)	26.1% (15.5% - 49.9%)	8.6% (7.6% - 9.8%)	18.1% (12.7% - 25.0%)	19.3% (16.2% - 25.0%)
India	11.2% (7.3% - 16.6%)	19.1% (4.5% - 51.4%)	25.7% (15.3% - 51.4%)	12.6% (11.0% - 14.3%)	29.2% (20.6% - 40.4%)	29.9% (23.8% - 40.4%)
Nepal	10.4% (6.6% - 15.4%)	17.8% (4.1% - 49.8%)	23.8% (13.7% - 49.8%)	7.7% (6.8% - 8.7%)	16.0% (11.3% - 22.7%)	17.2% (14.5% - 22.7%)
Pakistan	9.6% (5.9% - 14.6%)	13.2% (3.4% - 36.3%)	20.9% (12.2% - 36.3%)	8.4% (7.5% - 9.5%)	16.4% (11.5% - 22.5%)	18.5% (16.1% - 22.5%)
East Asia	23.9% (15.4% - 35.5%)	51.8% (14.5% - 89.2%)	58.5% (32.1% - 89.4%)	16.7% (14.7% - 19.0%)	38.5% (26.2% - 54.1%)	39.5% (31.5% - 54.1%)
China	23.9% (15.0% - 36.0%)	52.2% (14.0% - 90.0%)	58.6% (31.5% - 90.0%)	16.7% (14.7% - 19.0%)	38.4% (26.2% - 54.1%)	39.5% (31.4% - 54.1%)
North Korea	15.3% (9.9% - 22.9%)	19.7% (5.4% - 56.5%)	33.0% (20.8% - 56.5%)	11.0% (9.6% - 12.4%)	16.5% (11.1% - 22.8%)	23.7% (20.6% - 27.0%)
Taiwan	31.2% (19.8% - 47.0%)	46.5% (12.9% - 90.0%)	66.4% (41.1% - 90.0%)	37.7% (33.1% - 42.6%)	69.5% (50.2% - 80.0%)	78.5% (71.2% - 80.0%)
Oceania	16.4% (12.7% - 21.0%)	21.4% (10.4% - 44.2%)	34.9% (25.8% - 50.2%)	7.0% (6.2% - 7.8%)	8.7% (6.1% - 11.9%)	15.0% (12.8% - 18.1%)
American Samoa	25.8% (16.3% - 38.3%)	28.6% (7.9% - 73.9%)	54.8% (34.6% - 84.8%)	18.9% (16.6% - 21.6%)	24.1% (16.5% - 34.1%)	40.8% (35.6% - 46.7%)
Federated States of Micronesia	18.0% (11.2% - 26.5%)	27.2% (7.1% - 71.5%)	39.7% (24.1% - 71.5%)	11.9% (10.5% - 13.5%)	15.9% (11.1% - 22.8%)	25.8% (22.6% - 29.4%)
Fiji	19.2% (12.7% - 29.2%)	27.4% (7.2% - 78.5%)	42.1% (26.6% - 78.5%)	16.0% (14.1% - 18.0%)	22.0% (15.3% - 30.4%)	34.7% (30.2% - 39.2%)
Guam	30.3% (19.2% - 45.1%)	41.7% (11.6% - 90.0%)	64.4% (40.7% - 90.0%)	26.7% (23.5% - 30.2%)	31.9% (22.6% - 43.3%)	57.7% (50.4% - 64.9%)
Kiribati	19.8% (13.0% - 30.9%)	27.4% (7.0% - 75.4%)	43.2% (27.3% - 75.4%)	10.0% (8.8% - 11.3%)	12.8% (8.9% - 17.9%)	21.5% (18.8% - 24.5%)
Marshall Islands	23.0% (14.5% - 34.1%)	30.5% (8.0% - 80.8%)	49.6% (30.3% - 83.3%)	10.6% (9.4% - 11.9%)	15.5% (11.0% - 21.5%)	22.9% (20.1% - 26.0%)
Northern Mariana Islands	26.5% (16.7% - 39.6%)	39.5% (10.2% - 90.0%)	57.6% (35.0% - 90.0%)	23.2% (20.5% - 26.1%)	24.0% (16.8% - 33.2%)	50.1% (44.1% - 56.9%)
Papua New Guinea	14.2% (8.9% - 21.1%)	18.4% (4.6% - 52.9%)	30.8% (18.8% - 53.1%)	6.3% (5.6% - 7.1%)	7.8% (5.5% - 10.9%)	13.6% (11.9% - 15.4%)
Samoa	19.8% (12.4% - 30.6%)	26.9% (6.7% - 72.4%)	42.8% (25.4% - 72.4%)	14.8% (13.2% - 16.7%)	18.9% (13.4% - 26.3%)	32.0% (28.1% - 36.2%)
Solomon Islands	16.7% (10.4% - 25.4%)	20.9% (5.0% - 55.5%)	35.9% (21.8% - 58.3%)	6.6% (5.8% - 7.4%)	8.7% (6.1% - 11.8%)	14.2% (12.4% - 16.1%)
Tonga	19.9% (12.6% - 29.6%)	31.0% (8.8% - 87.4%)	43.9% (26.2% - 87.4%)	14.5% (12.8% - 16.3%)	19.0% (13.1% - 26.7%)	31.3% (27.5% - 35.4%)
Vanuatu	16.1% (10.2% - 24.6%)	20.6% (5.2% - 58.7%)	34.7% (21.4% - 59.7%)	8.6% (7.6% - 9.7%)	11.7% (8.3% - 15.9%)	18.6% (16.2% - 21.0%)
Southeast Asia	18.1% (15.0% - 22.2%)	32.6% (19.0% - 53.0%)	41.8% (31.6% - 59.2%)	15.7% (14.6% - 17.0%)	26.7% (21.6% - 33.1%)	34.1% (31.0% - 37.9%)
Cambodia	15.9% (10.0% - 24.3%)	28.2% (7.3% - 86.6%)	36.8% (20.6% - 86.6%)	8.2% (7.2% - 9.3%)	16.1% (11.2% - 22.9%)	18.0% (15.4% - 22.9%)
Indonesia	17.3% (10.9% - 25.9%)	32.8% (8.5% - 90.0%)	40.5% (23.4% - 90.0%)	15.8% (14.0% - 17.9%)	27.8% (19.5% - 38.3%)	34.2% (30.0% - 39.8%)
Laos	14.3% (8.9% - 21.1%)	20.6% (5.3% - 56.0%)	31.4% (18.5% - 57.5%)	8.8% (7.7% - 9.9%)	18.8% (13.0% - 26.4%)	19.8% (16.6% - 26.4%)
Malaysia	23.1% (14.5% - 34.2%)	40.3% (10.5% - 90.0%)	52.1% (30.2% - 90.0%)	20.6% (18.1% - 23.4%)	32.2% (22.7% - 44.4%)	44.6% (39.0% - 50.7%)
Maldives	29.1% (18.7% - 44.8%)	44.8% (13.0% - 90.0%)	62.5% (39.5% - 90.0%)	11.2% (9.9% - 12.5%)	21.8% (15.3% - 28.6%)	24.5% (21.3% - 28.7%)
Mauritius	25.5% (16.1% - 37.9%)	49.0% (13.9% - 90.0%)	58.7% (33.9% - 90.0%)	18.4% (16.3% - 20.9%)	29.7% (20.5% - 41.6%)	39.8% (34.8% - 45.9%)
Myanmar	14.3% (9.0% - 22.2%)	22.4% (12.3% - 37.3%)	30.4% (18.9% - 47.0%)	9.8% (8.6% - 11.0%)	17.1% (14.4% - 20.2%)	21.1% (18.4% - 24.0%)
Philippines	17.6% (11.1% - 26.4%)	31.1% (8.2% - 87.2%)	40.0% (23.7% - 87.2%)	13.9% (12.3% - 15.7%)	19.5% (13.6% - 27.4%)	30.0% (26.3% - 34.3%)
Sri Lanka	17.9% (11.0% - 27.2%)	28.3% (7.2% - 80.4%)	39.8% (23.3% - 80.4%)	17.4% (15.3% - 19.5%)	28.7% (19.9% - 40.4%)	37.7% (32.7% - 43.0%)
Seychelles	26.9% (17.1% - 40.3%)	36.4% (9.1% - 90.0%)	57.5% (36.0% - 90.0%)	19.7% (17.2% - 22.1%)	26.7% (18.5% - 36.5%)	42.5% (37.4% - 48.1%)
Thailand	20.7% (12.9% - 30.7%)	33.8% (9.0% - 90.0%)	46.0% (26.9% - 90.0%)	17.3% (15.3% - 19.7%)	26.5% (18.4% - 36.9%)	37.4% (32.8% - 42.8%)

Timor-Leste	15.3% (9.8% - 22.9%)	27.6% (7.0% - 76.8%)	35.6% (20.1% - 76.8%)	9.5% (8.3% - 10.8%)	21.2% (14.3% - 29.3%)	22.1% (17.9% - 29.3%)
Vietnam	17.5% (11.2% - 26.6%)	35.3% (9.0% - 90.0%)	42.4% (23.2% - 90.0%)	13.6% (12.0% - 15.4%)	26.7% (18.6% - 37.3%)	30.0% (25.7% - 37.3%)
Central Sub-Saharan Africa	12.2% (9.1% - 16.2%)	18.8% (8.3% - 39.9%)	27.7% (19.5% - 43.4%)	7.0% (6.2% - 7.8%)	14.5% (10.3% - 19.7%)	16.2% (13.7% - 20.1%)
Angola	15.3% (9.7% - 22.7%)	20.2% (5.1% - 55.5%)	33.1% (20.2% - 55.7%)	9.0% (8.0% - 10.2%)	22.5% (15.7% - 31.5%)	22.7% (17.3% - 31.5%)
Central African Republic	11.1% (7.0% - 16.8%)	13.2% (3.1% - 35.3%)	24.0% (14.5% - 38.4%)	4.6% (4.0% - 5.2%)	5.5% (3.9% - 7.7%)	9.9% (8.7% - 11.2%)
Congo	14.0% (8.7% - 21.5%)	21.5% (4.9% - 61.7%)	31.3% (18.3% - 61.7%)	12.7% (11.1% - 14.4%)	23.9% (16.7% - 32.7%)	27.6% (23.9% - 33.0%)
Democratic Republic of the Congo	10.8% (6.8% - 16.5%)	17.9% (4.6% - 49.3%)	24.4% (14.2% - 49.3%)	6.7% (5.9% - 7.5%)	12.6% (8.8% - 17.5%)	14.6% (12.7% - 17.5%)
Equatorial Guinea	20.5% (13.1% - 32.4%)	25.0% (12.7% - 47.2%)	43.3% (27.1% - 69.1%)	19.1% (16.7% - 21.6%)	39.4% (32.3% - 48.0%)	41.7% (36.1% - 48.2%)
Gabon	20.9% (12.8% - 31.2%)	26.1% (6.2% - 72.2%)	44.9% (27.1% - 73.6%)	17.2% (15.1% - 19.5%)	37.7% (25.6% - 52.1%)	39.3% (32.3% - 52.1%)
Eastern Sub-Saharan Africa	13.0% (10.9% - 15.3%)	20.8% (12.7% - 36.1%)	29.2% (22.6% - 41.1%)	7.1% (6.5% - 7.7%)	14.1% (11.2% - 17.5%)	15.6% (12.8% - 18.8%)
Burundi	11.8% (7.3% - 17.7%)	16.1% (4.1% - 41.5%)	25.7% (15.3% - 42.4%)	4.8% (4.2% - 5.4%)	6.2% (4.5% - 8.5%)	10.3% (9.1% - 11.8%)
Comoros	15.1% (9.4% - 22.7%)	18.3% (4.6% - 53.4%)	32.5% (19.8% - 54.4%)	8.6% (7.6% - 9.8%)	14.4% (10.0% - 20.2%)	18.6% (16.3% - 21.4%)
Djibouti	13.8% (9.0% - 21.0%)	15.5% (4.1% - 40.9%)	29.6% (18.7% - 47.4%)	8.7% (7.6% - 9.8%)	16.9% (11.7% - 23.6%)	19.1% (16.5% - 23.6%)
Eritrea	11.8% (7.6% - 18.1%)	14.6% (3.9% - 43.5%)	25.5% (15.9% - 44.6%)	7.0% (6.2% - 7.9%)	11.6% (8.2% - 16.0%)	15.2% (13.3% - 17.3%)
Ethiopia	12.0% (7.4% - 18.8%)	21.1% (4.8% - 59.2%)	27.7% (15.5% - 59.2%)	5.8% (5.1% - 6.6%)	13.5% (9.2% - 18.8%)	13.9% (10.9% - 18.8%)
Kenya	16.1% (10.1% - 24.6%)	23.6% (5.6% - 63.1%)	35.4% (21.0% - 63.2%)	10.4% (9.1% - 11.8%)	17.5% (12.4% - 24.6%)	22.4% (19.6% - 25.8%)
Madagascar	11.5% (7.2% - 17.4%)	14.6% (3.8% - 37.9%)	24.8% (15.1% - 39.8%)	7.0% (6.2% - 7.9%)	10.8% (7.6% - 15.4%)	15.2% (13.3% - 17.2%)
Malawi	13.1% (8.1% - 19.5%)	23.2% (6.2% - 62.9%)	30.1% (17.0% - 62.9%)	6.8% (5.9% - 7.7%)	13.0% (8.9% - 17.9%)	14.8% (12.6% - 18.0%)
Mozambique	12.4% (8.0% - 19.2%)	19.9% (4.8% - 58.8%)	28.1% (17.0% - 58.8%)	5.1% (4.5% - 5.8%)	10.0% (6.9% - 14.1%)	11.3% (9.7% - 14.1%)
Rwanda	13.3% (8.6% - 20.4%)	25.8% (6.9% - 70.7%)	31.7% (17.6% - 70.7%)	7.8% (6.9% - 8.8%)	16.0% (11.4% - 21.9%)	17.4% (14.8% - 21.9%)
Somalia	8.4% (5.4% - 12.5%)	11.0% (2.6% - 30.6%)	18.3% (11.1% - 31.1%)	2.4% (2.1% - 2.7%)	2.3% (1.6% - 3.2%)	5.1% (4.5% - 5.8%)
South Sudan	12.0% (7.4% - 18.4%)	17.3% (4.2% - 48.8%)	26.4% (15.7% - 48.8%)	6.3% (5.6% - 7.0%)	8.9% (6.4% - 12.1%)	13.5% (11.9% - 15.3%)
Tanzania	13.3% (8.3% - 20.0%)	23.3% (6.1% - 64.9%)	30.6% (17.3% - 64.9%)	7.7% (6.8% - 8.7%)	13.5% (9.5% - 18.6%)	16.7% (14.6% - 19.3%)
Uganda	13.3% (8.1% - 20.1%)	18.7% (4.7% - 48.7%)	29.1% (17.2% - 49.5%)	7.2% (6.3% - 8.2%)	17.4% (11.8% - 23.9%)	17.6% (13.6% - 23.9%)
Zambia	14.5% (9.1% - 22.5%)	19.0% (4.6% - 51.6%)	31.3% (18.9% - 52.6%)	10.2% (9.0% - 11.7%)	23.3% (16.2% - 32.5%)	24.0% (19.3% - 32.5%)
Southern Sub-Saharan Africa	22.9% (15.2% - 33.1%)	32.8% (11.6% - 76.4%)	50.0% (32.0% - 79.3%)	18.4% (16.3% - 20.8%)	25.8% (18.4% - 35.9%)	39.7% (34.7% - 45.1%)
Botswana	23.6% (15.1% - 35.3%)	36.4% (10.6% - 90.0%)	51.7% (31.5% - 90.0%)	15.9% (14.0% - 18.1%)	27.5% (19.2% - 38.5%)	34.5% (29.9% - 39.8%)
Lesotho	17.2% (10.9% - 26.5%)	30.3% (8.0% - 90.0%)	39.2% (22.6% - 90.0%)	10.3% (9.1% - 11.8%)	17.8% (12.4% - 24.9%)	22.3% (19.4% - 26.0%)
Namibia	24.0% (15.4% - 36.2%)	32.6% (8.1% - 88.7%)	51.7% (32.7% - 90.0%)	14.8% (12.9% - 16.8%)	23.6% (16.1% - 32.9%)	32.0% (27.8% - 36.5%)
South Africa	24.1% (14.7% - 36.6%)	34.5% (8.9% - 90.0%)	52.3% (30.4% - 90.0%)	18.5% (16.3% - 21.0%)	26.0% (18.4% - 36.5%)	40.0% (35.0% - 45.6%)
Swaziland	20.9% (12.8% - 31.7%)	33.9% (8.8% - 90.0%)	46.6% (26.7% - 90.0%)	13.1% (11.5% - 14.9%)	21.2% (14.8% - 29.0%)	28.3% (24.8% - 32.4%)
Zimbabwe	14.5% (9.2% - 21.8%)	17.9% (4.5% - 49.8%)	31.4% (19.4% - 51.8%)	9.2% (8.1% - 10.4%)	9.5% (6.5% - 13.6%)	19.9% (17.5% - 22.7%)
Western Sub-Saharan Africa	14.3% (11.7% - 17.9%)	21.5% (11.6% - 41.4%)	31.9% (24.0% - 46.1%)	10.3% (9.1% - 11.6%)	22.8% (16.4% - 30.9%)	23.9% (19.7% - 31.1%)
Benin	12.5% (7.7% - 18.5%)	16.1% (4.0% - 43.6%)	27.0% (16.2% - 44.5%)	6.0% (5.3% - 6.8%)	9.0% (6.4% - 12.3%)	13.0% (11.3% - 14.7%)

Burkina Faso	13.0%<br>(8.4% - 19.7%)	23.9%<br>(6.3% - 66.8%)	30.5%<br>(17.4% - 66.8%)	4.3%<br>(3.8% - 4.9%)	6.5%<br>(4.6% - 8.9%)	9.4%<br>(8.2% - 10.7%)
Cameroon	14.6%<br>(9.3% - 21.9%)	20.4%<br>(5.0% - 56.0%)	32.0%<br>(19.5% - 56.0%)	9.7%<br>(8.5% - 11.0%)	17.8%<br>(12.6% - 24.8%)	21.1%<br>(18.2% - 24.8%)
Cape Verde	18.6%<br>(11.8% - 28.2%)	27.3%<br>(7.4% - 79.3%)	40.6%<br>(24.9% - 79.3%)	10.9%<br>(9.6% - 12.4%)	23.1%<br>(16.6% - 32.8%)	24.6%<br>(20.6% - 32.8%)
Chad	11.9%<br>(7.6% - 17.7%)	14.6%<br>(3.5% - 39.7%)	25.7%<br>(15.9% - 40.0%)	4.1%<br>(3.6% - 4.6%)	6.4%<br>(4.5% - 8.8%)	8.8%<br>(7.7% - 10.0%)
Cote d'Ivoire	15.1%<br>(9.3% - 22.6%)	19.5%<br>(4.8% - 52.8%)	32.5%<br>(19.6% - 53.9%)	7.3%<br>(6.4% - 8.3%)	11.4%<br>(8.0% - 15.9%)	15.8%<br>(13.8% - 18.0%)
The Gambia	13.0%<br>(8.2% - 19.8%)	20.9%<br>(5.3% - 55.6%)	29.2%<br>(17.2% - 55.6%)	7.1%<br>(6.2% - 8.0%)	12.9%<br>(9.2% - 18.0%)	15.4%<br>(13.4% - 18.0%)
Ghana	15.1%<br>(9.7% - 22.5%)	26.1%<br>(6.8% - 76.6%)	34.7%<br>(20.4% - 76.6%)	12.2%<br>(10.7% - 13.9%)	24.3%<br>(16.9% - 34.1%)	26.9%<br>(23.0% - 34.1%)
Guinea	13.5%<br>(8.5% - 20.3%)	23.2%<br>(6.2% - 62.1%)	30.9%<br>(18.0% - 62.1%)	5.5%<br>(4.8% - 6.2%)	8.7%<br>(6.1% - 11.9%)	11.8%<br>(10.5% - 13.6%)
Guinea-Bissau	14.3%<br>(8.6% - 21.6%)	20.9%<br>(5.1% - 57.9%)	31.5%<br>(18.5% - 57.9%)	6.1%<br>(5.4% - 6.9%)	9.8%<br>(6.9% - 13.6%)	13.2%<br>(11.6% - 15.2%)
Liberia	14.7%<br>(9.3% - 22.5%)	31.8%<br>(8.1% - 90.0%)	36.5%<br>(19.3% - 90.0%)	6.4%<br>(5.6% - 7.3%)	14.3%<br>(9.9% - 19.9%)	14.8%<br>(12.1% - 19.9%)
Mali	12.3%<br>(7.7% - 18.8%)	15.0%<br>(3.7% - 43.4%)	26.5%<br>(16.1% - 44.8%)	4.4%<br>(3.9% - 5.0%)	6.9%<br>(4.8% - 9.5%)	9.6%<br>(8.4% - 11.0%)
Mauritania	14.3%<br>(9.1% - 21.3%)	18.8%<br>(4.7% - 51.1%)	31.0%<br>(18.5% - 51.8%)	9.9%<br>(8.7% - 11.3%)	18.0%<br>(12.7% - 25.2%)	21.5%<br>(18.8% - 25.4%)
Niger	11.9%<br>(7.7% - 17.6%)	16.5%<br>(4.4% - 43.8%)	26.0%<br>(16.3% - 44.7%)	3.1%<br>(2.7% - 3.6%)	4.1%<br>(2.9% - 5.6%)	6.7%<br>(5.9% - 7.7%)
Nigeria	14.9%<br>(9.3% - 22.4%)	22.3%<br>(5.5% - 62.4%)	32.9%<br>(19.7% - 62.4%)	10.6%<br>(9.3% - 12.0%)	23.8%<br>(16.4% - 32.8%)	24.6%<br>(19.9% - 32.8%)
Sao Tome and Principe	16.3%<br>(10.4% - 24.3%)	18.8%<br>(5.0% - 50.3%)	34.9%<br>(22.2% - 54.3%)	10.1%<br>(8.8% - 11.4%)	21.4%<br>(14.7% - 29.6%)	22.7%<br>(19.1% - 29.6%)
Senegal	14.8%<br>(9.3% - 22.8%)	22.1%<br>(5.8% - 63.0%)	32.8%<br>(19.6% - 63.0%)	6.8%<br>(6.0% - 7.7%)	10.9%<br>(7.6% - 15.0%)	14.8%<br>(13.0% - 16.8%)
Sierra Leone	14.7%<br>(9.5% - 22.2%)	22.2%<br>(5.8% - 62.3%)	32.6%<br>(19.6% - 63.1%)	5.9%<br>(5.2% - 6.7%)	10.7%<br>(7.8% - 14.9%)	12.9%<br>(11.2% - 15.0%)
Togo	13.1%<br>(8.3% - 19.5%)	21.0%<br>(5.4% - 55.6%)	29.5%<br>(17.3% - 55.6%)	7.5%<br>(6.6% - 8.5%)	11.8%<br>(8.2% - 16.8%)	16.3%<br>(14.2% - 18.6%)

Table S12: facility and community unit cost attributable to dementia by GBD region and country in 2019 and 2050 baseline and increased projections (2019 USD)

	Unit cost in community based care settings 2019	Unit cost in community based care settings 2050 (Baseline)	Unit cost in community based care settings 2050 (Accelerated)	Unit cost in nursing home based care settings 2019	Unit cost in nursing home based care settings 2050 (Baseline)	Unit cost in nursing home based care settings 2050 (Accelerated)
Central Asia	$1188 ($697 - $1718)	$2269 ($1130 - $4032)	$2650 ($1437 - $4387)	$4711 ($2341 - $7378)	$12 889 ($5753 - $23 896)	$15 776 ($7780 - $26 409)
Armenia	$1591 ($832 - $2614)	$5728 ($1627 - $14 442)	$5769 ($1996 - $14 442)	$6190 ($2935 - $10 391)	$31 156 ($9702 - $72 633)	$31 384 ($10 764 - $72 633)
Azerbaijan	$991 ($528 - $1645)	$2551 ($810 - $6476)	$2659 ($1095 - $6476)	$3737 ($1741 - $6210)	$13 527 ($3789 - $32 093)	$14 335 ($5624 - $32 093)
Georgia	$1390 ($745 - $2192)	$3127 ($1001 - $7761)	$3402 ($1517 - $7761)	$5352 ($2493 - $8729)	$16 236 ($4913 - $36 647)	$18 402 ($7790 - $36 647)
Kazakhstan	$1307 ($679 - $2123)	$1775 ($558 - $4221)	$2691 ($1395 - $4671)	$4973 ($2418 - $8150)	$8717 ($2621 - $20 485)	$15 333 ($7274 - $26 093)
Kyrgyzstan	$728 ($410 - $1136)	$1042 ($312 - $2518)	$1506 ($804 - $2606)	$2706 ($1279 - $4588)	$5168 ($1593 - $12 443)	$8386 ($3868 - $14 327)
Mongolia	$1114 ($591 - $1812)	$2158 ($649 - $5312)	$2519 ($1174 - $5312)	$4294 ($2056 - $7241)	$11 485 ($3333 - $29 072)	$14 147 ($6226 - $29 072)
Tajikistan	$622 ($338 - $985)	$1626 ($500 - $3913)	$1692 ($728 - $3913)	$2306 ($1079 - $3764)	$8558 ($2536 - $20 535)	$9017 ($3474 - $20 535)
Turkmenistan	$1835 ($953 - $2972)	$4246 ($1318 - $10 326)	$4585 ($1971 - $10 326)	$7253 ($3399 - $11 768)	$22 630 ($6410 - $50 544)	$25 286 ($10 930 - $50 544)
Uzbekistan	$779 ($440 - $1238)	$1293 ($387 - $3268)	$1666 ($869 - $3273)	$2880 ($1345 - $4707)	$6259 ($1859 - $14 512)	$9012 ($4060 - $15 667)
Central Europe	$2324 ($1349 - $3420)	$3783 ($1799 - $6627)	$4996 ($2758 - $8047)	$9358 ($4664 - $14 318)	$20 022 ($8919 - $35 894)	$29 010 ($14 442 - $45 919)
Albania	$1319 ($716 - $2101)	$1680 ($517 - $4347)	$2702 ($1431 - $4685)	$4898 ($2284 - $7937)	$8043 ($2572 - $18 136)	$15 090 ($7066 - $25 014)
Bosnia and Herzegovina	$1757 ($949 - $2825)	$2983 ($893 - $7559)	$3779 ($1912 - $7565)	$6810 ($3161 - $11 415)	$15 710 ($4630 - $38 945)	$21 599 ($9880 - $39 854)
Bulgaria	$2062 ($1113 - $3295)	$3732 ($1090 - $9284)	$4536 ($2228 - $9284)	$8042 ($3749 - $13 220)	$19 476 ($5967 - $44 178)	$25 684 ($11 597 - $45 466)
Croatia	$2332 ($1195 - $3666)	$2678 ($791 - $6477)	$4732 ($2435 - $7581)	$9184 ($4314 - $15 199)	$13 911 ($3977 - $32 069)	$28 283 ($13 834 - $47 387)
Czech Republic	$3065 ($1623 - $4973)	$4663 ($1426 - $10 987)	$6406 ($3205 - $11 611)	$12 341 ($5851 - $20 634)	$25 373 ($7817 - $62 209)	$38 515 ($17 806 - $67 509)
Hungary	$2431 ($1262 - $3927)	$3015 ($871 - $7508)	$4970 ($2554 - $8363)	$9535 ($4382 - $16 145)	$15 694 ($4419 - $38 459)	$29 407 ($13 349 - $49 038)
Macedonia	$1554 ($809 - $2526)	$1576 ($481 - $3791)	$3138 ($1629 - $5132)	$5875 ($2770 - $9440)	$7718 ($2159 - $18 117)	$18 058 ($8875 - $29 650)
Montenegro	$1997 ($1095 - $3202)	$3481 ($1079 - $8580)	$4334 ($2155 - $8594)	$7848 ($3737 - $13 225)	$18 794 ($5690 - $45 033)	$25 076 ($11 527 - $46 334)
Poland	$2337 ($1262 - $3774)	$3817 ($1145 - $9283)	$4986 ($2541 - $9411)	$9221 ($4259 - $15 154)	$20 504 ($6385 - $47 662)	$29 059 ($13 069 - $50 336)
Romania	$2086 ($1097 - $3334)	$4349 ($1253 - $11 466)	$4888 ($2222 - $11 466)	$7965 ($3753 - $12 736)	$22 525 ($6710 - $55 803)	$26 632 ($11 391 - $55 803)
Serbia	$1729 ($921 - $2726)	$3166 ($945 - $7229)	$3790 ($1835 - $7229)	$6607 ($3032 - $10 954)	$16 272 ($4732 - $37 007)	$21 094 ($9334 - $38 009)
Slovakia	$2696 ($1461 - $4377)	$4556 ($1332 - $11 147)	$5772 ($3005 - $11 147)	$10 668 ($4798 - $17 384)	$24 096 ($6849 - $56 264)	$33 762 ($15 094 - $59 123)
Slovenia	$3342 ($1763 - $5449)	$3977 ($1212 - $9708)	$6799 ($3634 - $11 558)	$13 614 ($6391 - $23 639)	$21 845 ($6328 - $51 105)	$41 967 ($19 557 - $74 866)
Eastern Europe	$1683 ($949 - $2535)	$2452 ($980 - $5077)	$3447 ($1848 - $5652)	$6817 ($3341 - $10 696)	$13 380 ($5195 - $26 264)	$21 135 ($10 632 - $33 265)
Belarus	$1457 ($787 - $2360)	$2504 ($764 - $6257)	$3141 ($1574 - $6329)	$5663 ($2577 - $9054)	$13 267 ($3981 - $29 895)	$17 955 ($7998 - $31 480)
Estonia	$2908 ($1515 - $4781)	$5377 ($1463 - $12 896)	$6458 ($3051 - $12 896)	$11 894 ($5427 - $19 392)	$29 689 ($8444 - $69 710)	$38 296 ($17 490 - $69 710)
Latvia	$2474 ($1293 - $4121)	$4410 ($1371 - $11 223)	$5422 ($2634 - $11 223)	$10 032 ($4580 - $16 328)	$23 665 ($6911 - $55 593)	$31 819 ($14 435 - $56 286)
Lithuania	$2637 ($1349 - $4307)	$4775 ($1385 - $11 758)	$5748 ($2775 - $11 758)	$10 668 ($5156 - $18 339)	$27 208 ($8323 - $67 926)	$34 581 ($15 906 - $67 926)

Moldova	$1132 ($603 - $1846)	$1994 ($573 - $5080)	$2473 ($1204 - $5080)	$4332 ($1996 - $7260)	$10 136 ($2969 - $24 425)	$13 757 ($6186 - $24 925)
Russian Federation	$1788 ($941 - $2808)	$2546 ($733 - $6270)	$3698 ($1891 - $6472)	$7013 ($3328 - $11 592)	$13 092 ($4039 - $30 759)	$21 752 ($10 179 - $36 010)
Ukraine	$1250 ($645 - $2030)	$1929 ($590 - $5048)	$2626 ($1329 - $5048)	$4742 ($2192 - $8025)	$9586 ($2837 - $24 298)	$14 838 ($6759 - $25 719)
Australasia	$4803 ($2671 - $7531)	$6037 ($2217 - $13 541)	$9751 ($5246 - $15 513)	$29 069 ($14 041 - $47 445)	$80 059 ($26 415 - $176 127)	$96 786 ($42 495 - $179 685)
Australia	$4900 ($2609 - $7922)	$6159 ($1930 - $14 542)	$9988 ($5173 - $16 708)	$29 742 ($13 757 - $49 791)	$80 815 ($23 422 - $190 433)	$98 286 ($42 115 - $193 048)
New Zealand	$4289 ($2334 - $6956)	$5619 ($1678 - $13 834)	$8798 ($4747 - $15 202)	$25 741 ($11 838 - $41 531)	$74 201 ($23 908 - $176 883)	$86 832 ($36 245 - $176 883)
High-income Asia Pacific	$4355 ($2309 - $6823)	$6349 ($2314 - $12 811)	$8953 ($4730 - $14 315)	$25 666 ($12 530 - $41 738)	$88 410 ($33 633 - $189 019)	$96 109 ($41 517 - $190 171)
Brunei	$1685 ($924 - $2847)	$1163 ($343 - $2923)	$3398 ($1812 - $5738)	$9494 ($4568 - $15 638)	$13 568 ($4156 - $32 247)	$29 201 ($14 165 - $49 887)
Japan	$4458 ($2300 - $7105)	$6069 ($1743 - $14 504)	$9152 ($4620 - $15 167)	$26 885 ($12 761 - $44 279)	$79 932 ($23 911 - $191 623)	$92 157 ($40 172 - $191 623)
Singapore	$3613 ($1994 - $5713)	$5970 ($1746 - $14 350)	$7691 ($3987 - $14 386)	$21 407 ($9973 - $35 382)	$76 957 ($23 574 - $180 617)	$81 708 ($32 101 - $180 617)
South Korea	$3396 ($1793 - $5528)	$7951 ($2359 - $18 921)	$8525 ($3750 - $18 921)	$19 837 ($9783 - $32 830)	$102 613 ($31 982 - $242 278)	$103 135 ($34 120 - $242 278)
High-income North America	$7959 ($5142 - $11 778)	$7778 ($2235 - $19 277)	$15 412 ($10 101 - $22 272)	$82 723 ($49 776 - $125 268)	$89 017 ($20 467 - $242 213)	$249 993 ($153 246 - $369 370)
Canada	$2059 ($1078 - $3330)	$4049 ($1310 - $9714)	$4673 ($2199 - $9714)	$36 977 ($16 042 - $61 210)	$58 990 ($17 548 - $135 192)	$113 852 ($47 199 - $191 835)
Greenland	$6816 ($3715 - $11 325)	$14 123 ($4182 - $35 028)	$15 897 ($7368 - $35 028)	$41 535 ($19 112 - $68 949)	$64 607 ($19 850 - $153 055)	$127 955 ($59 422 - $218 012)
United States	$8689 ($5368 - $13 365)	$8954 ($2106 - $26 090)	$17 657 ($10 855 - $27 912)	$85 640 ($50 045 - $133 055)	$94 186 ($19 047 - $275 430)	$263 297 ($154 348 - $415 926)
Southern Latin America	$1770 ($966 - $2848)	$2603 ($1037 - $5578)	$3640 ($1901 - $6234)	$6337 ($3074 - $9892)	$11 700 ($4347 - $23 439)	$20 232 ($9901 - $33 454)
Argentina	$2018 ($1036 - $3422)	$2760 ($822 - $7098)	$4152 ($2058 - $7702)	$5700 ($2658 - $9079)	$8045 ($2657 - $18 800)	$17 530 ($8153 - $28 732)
Chile	$1316 ($703 - $2067)	$2543 ($768 - $6045)	$2964 ($1409 - $6045)	$7218 ($3317 - $11 919)	$15 330 ($4285 - $34 953)	$22 561 ($10 316 - $39 692)
Uruguay	$1581 ($825 - $2558)	$1782 ($530 - $4485)	$3204 ($1650 - $5377)	$7756 ($3586 - $12 686)	$12 857 ($3866 - $30 239)	$23 925 ($11 314 - $40 146)
Western Europe	$5960 ($3461 - $8721)	$7150 ($3738 - $12 522)	$12 269 ($6842 - $18 565)	$27 225 ($13 751 - $42 646)	$53 813 ($25 213 - $92 449)	$83 551 ($43 361 - $132 816)
Andorra	$4043 ($2148 - $6711)	$3545 ($1034 - $8351)	$8146 ($4347 - $13 312)	$20 844 ($9677 - $34 039)	$38 044 ($11 479 - $95 263)	$64 406 ($29 940 - $109 262)
Austria	$5288 ($2792 - $8631)	$4893 ($1453 - $12 303)	$10 665 ($5596 - $17 593)	$27 756 ($13 283 - $45 827)	$55 570 ($16 017 - $133 679)	$86 329 ($40 363 - $149 457)
Belgium	$5175 ($2841 - $8485)	$5537 ($1627 - $13 603)	$10 457 ($5705 - $17 259)	$26 567 ($13 023 - $43 526)	$59 653 ($18 821 - $139 234)	$83 448 ($39 882 - $150 525)
Cyprus	$2749 ($1402 - $4469)	$2543 ($744 - $5719)	$5541 ($2825 - $8888)	$13 581 ($6451 - $22 200)	$26 755 ($8541 - $62 873)	$42 194 ($19 605 - $71 713)
Denmark	$5698 ($3006 - $9043)	$5501 ($1582 - $13 046)	$11 492 ($6148 - $18 473)	$29 522 ($13 707 - $49 125)	$62 389 ($18 454 - $149 645)	$92 650 ($41 024 - $168 419)
Finland	$4964 ($2635 - $7863)	$5207 ($1710 - $12 363)	$10 049 ($5304 - $16 019)	$25 648 ($12 383 - $42 778)	$57 900 ($17 897 - $135 958)	$81 219 ($39 187 - $147 986)
France	$8337 ($4433 - $13 700)	$8075 ($2542 - $19 651)	$16 812 ($8788 - $27 633)	$26 463 ($12 644 - $44 345)	$53 120 ($14 842 - $118 971)	$82 069 ($39 388 - $141 840)

Germany	$4213 ($2216 - $6719)	$4593 ($1362 - $11 338)	$8525 ($4449 - $14 192)	$28 096 ($12 620 - $47 236)	$58 372 ($18 091 - $132 940)	$87 382 ($38 930 - $149 784)
Greece	$3020 ($1614 - $4867)	$2424 ($737 - $5794)	$6089 ($3255 - $9656)	$15 355 ($6968 - $25 246)	$26 865 ($7283 - $64 018)	$47 425 ($21 364 - $79 924)
Iceland	$5332 ($2896 - $8477)	$4973 ($1387 - $13 431)	$10 777 ($5780 - $17 730)	$28 211 ($12 942 - $47 440)	$57 007 ($15 628 - $144 842)	$88 009 ($39 684 - $154 720)
Ireland	$1953 ($1017 - $3205)	$3560 ($1038 - $9230)	$4321 ($2099 - $9230)	$28 715 ($13 606 - $47 251)	$83 867 ($26 285 - $194 190)	$97 137 ($43 266 - $194 190)
Israel	$6286 ($3301 - $10 160)	$8328 ($2471 - $19 910)	$12 876 ($6610 - $22 475)	$14 267 ($6779 - $23 556)	$21 374 ($6649 - $53 320)	$43 955 ($20 919 - $73 326)
Italy	$7310 ($3872 - $11 546)	$8511 ($2682 - $20 953)	$14 838 ($7850 - $24 449)	$20 858 ($9343 - $34 495)	$36 284 ($11 388 - $86 974)	$64 394 ($28 373 - $109 326)
Luxembourg	$5709 ($3053 - $9356)	$4804 ($1385 - $12 473)	$11 520 ($6142 - $19 363)	$29 889 ($13 577 - $49 308)	$53 211 ($15 647 - $126 399)	$92 417 ($43 148 - $156 730)
Malta	$4168 ($2137 - $6807)	$6223 ($1928 - $15 172)	$8703 ($4439 - $15 782)	$21 574 ($10 079 - $35 761)	$73 373 ($19 957 - $182 484)	$79 255 ($33 209 - $182 484)
Netherlands	$10 658 ($5877 - $16 918)	$17 468 ($5149 - $42 515)	$22 772 ($11 820 - $42 515)	$28 545 ($13 440 - $46 845)	$64 616 ($20 750 - $159 249)	$90 181 ($41 486 - $162 909)
Norway	$6139 ($3262 - $9766)	$6201 ($1871 - $15 273)	$12 414 ($6627 - $20 344)	$32 300 ($14 989 - $54 883)	$68 154 ($18 511 - $160 063)	$100 791 ($46 275 - $180 496)
Portugal	$3525 ($1820 - $5704)	$3264 ($992 - $8211)	$7116 ($3711 - $11 565)	$14 239 ($6752 - $24 516)	$19 799 ($6134 - $51 804)	$43 812 ($20 698 - $76 717)
Spain	$4784 ($2655 - $7913)	$6717 ($2044 - $16 331)	$9861 ($5286 - $17 560)	$20 149 ($9901 - $33 386)	$45 813 ($13 378 - $108 749)	$63 655 ($30 163 - $115 900)
Sweden	$7910 ($4254 - $12 843)	$13 369 ($4084 - $33 988)	$17 065 ($8461 - $33 988)	$44 748 ($20 763 - $76 989)	$82 705 ($25 766 - $187 602)	$138 266 ($62 800 - $239 873)
Switzerland	$9089 ($4950 - $14 307)	$12 464 ($3689 - $29 371)	$18 613 ($9807 - $31 363)	$31 561 ($14 914 - $51 082)	$47 136 ($15 110 - $106 039)	$97 037 ($46 021 - $159 583)
United Kingdom	$4538 ($2436 - $7402)	$6909 ($2078 - $17 510)	$9520 ($4918 - $18 300)	$37 486 ($17 643 - $63 001)	$70 644 ($20 146 - $163 124)	$115 857 ($53 958 - $194 808)
Andean Latin America	$1457 ($818 - $2195)	$2230 ($949 - $4511)	$3070 ($1682 - $5107)	$7513 ($3795 - $11 889)	$21 467 ($8146 - $43 410)	$25 519 ($12 076 - $45 708)
Bolivia	$1183 ($633 - $1935)	$1760 ($563 - $4362)	$2461 ($1285 - $4407)	$5948 ($2943 - $9763)	$16 190 ($5547 - $38 912)	$19 511 ($9232 - $38 912)
Ecuador	$1667 ($934 - $2642)	$3060 ($976 - $7412)	$3674 ($1901 - $7412)	$8534 ($4028 - $14 297)	$29 206 ($8369 - $69 114)	$31 643 ($12 839 - $69 114)
Peru	$1414 ($742 - $2301)	$1919 ($629 - $4488)	$2903 ($1467 - $4975)	$7264 ($3542 - $12 065)	$18 331 ($5347 - $43 491)	$23 356 ($10 782 - $43 743)
Caribbean	$2203 ($1273 - $3336)	$4007 ($1729 - $8155)	$4912 ($2522 - $8839)	$12 359 ($6377 - $19 377)	$39 368 ($16 567 - $77 985)	$44 906 ($20 769 - $82 569)
Antigua and Barbuda	$2291 ($1165 - $3753)	$2447 ($715 - $6219)	$4640 ($2348 - $7766)	$11 982 ($5432 - $19 494)	$22 788 ($6591 - $53 003)	$37 100 ($17 049 - $61 721)
The Bahamas	$3325 ($1757 - $5204)	$3850 ($1181 - $9620)	$6751 ($3498 - $11 178)	$18 125 ($8985 - $30 229)	$38 454 ($11 673 - $93 203)	$56 687 ($27 701 - $99 435)
Barbados	$2472 ($1274 - $4016)	$2280 ($731 - $5770)	$4986 ($2542 - $8163)	$12 865 ($6173 - $21 048)	$21 167 ($6468 - $48 087)	$39 744 ($18 803 - $67 405)
Belize	$1293 ($658 - $2070)	$1509 ($437 - $3689)	$2623 ($1316 - $4403)	$6521 ($2963 - $10 972)	$13 897 ($4309 - $33 187)	$20 446 ($9131 - $36 229)
Bermuda	$6087 ($3380 - $9724)	$8364 ($2671 - $21 239)	$12 545 ($6549 - $21 721)	$33 872 ($16 128 - $54 236)	$82 567 ($26 249 - $177 290)	$107 960 ($48 540 - $188 040)
Cuba	$2516 ($1357 - $4099)	$5700 ($1680 - $13 959)	$6219 ($2807 - $13 959)	$13 157 ($6471 - $21 486)	$55 874 ($17 022 - $120 679)	$57 075 ($22 340 - $120 679)
Dominica	$1573 ($838 - $2548)	$1527 ($474 - $3827)	$3177 ($1672 - $5186)	$7956 ($3892 - $13 166)	$13 500 ($4370 - $31 541)	$24 508 ($11 995 - $40 912)
Dominican Republic	$1602 ($862 - $2598)	$2648 ($827 - $6707)	$3439 ($1730 - $6791)	$8273 ($4113 - $13 972)	$25 715 ($8497 - $61 644)	$28 808 ($13 060 - $61 644)
Grenada	$1718 ($925 - $2759)	$1755 ($536 - $4398)	$3476 ($1851 - $5698)	$8780 ($4195 - $14 451)	$15 534 ($5014 - $36 624)	$27 099 ($12 872 - $44 453)

Guyana	$1369<br>($743 - $2175)	$2307<br>($671 - $5574)	$2946<br>($1497 - $5574)	$6961<br>($3349 - $11 412)	$21 708<br>($6960 - $51 520)	$24 369<br>($10 794 - $51 520)
Haiti	$553<br>($304 - $882)	$490<br>($151 - $1163)	$1115<br>($609 - $1804)	$2655<br>($1246 - $4271)	$4124<br>($1253 - $9379)	$8183<br>($3803 - $13 412)
Jamaica	$1460<br>($774 - $2345)	$1552<br>($484 - $3632)	$2953<br>($1570 - $4850)	$7419<br>($3520 - $12 151)	$13 999<br>($4101 - $31 658)	$23 030<br>($10 603 - $38 801)
Puerto Rico	$2582<br>($1316 - $4172)	$3151<br>($977 - $7341)	$5268<br>($2606 - $8994)	$13 659<br>($6565 - $23 265)	$30 824<br>($9137 - $78 486)	$43 355<br>($20 236 - $81 798)
Saint Lucia	$1740<br>($944 - $2972)	$1618<br>($510 - $4244)	$3511<br>($1922 - $5873)	$8928<br>($4195 - $14 832)	$14 816<br>($4445 - $36 114)	$27 578<br>($13 125 - $46 367)
Saint Vincent and the Grenadines	$1443<br>($808 - $2363)	$1668<br>($526 - $4162)	$2931<br>($1589 - $4992)	$7343<br>($3527 - $12 445)	$15 550<br>($4584 - $38 784)	$23 012<br>($10 764 - $41 401)
Suriname	$1517<br>($793 - $2474)	$2198<br>($627 - $5349)	$3148<br>($1612 - $5554)	$7858<br>($3531 - $13 071)	$21 296<br>($5735 - $50 911)	$25 837<br>($11 260 - $50 911)
Trinidad and Tobago	$2455<br>($1276 - $3921)	$3921<br>($1145 - $9163)	$5175<br>($2684 - $9275)	$13 061<br>($6199 - $22 350)	$38 611<br>($11 871 - $90 832)	$44 408<br>($20 064 - $91 159)
Virgin Islands, U.S.	$2308<br>($1217 - $3683)	$3132<br>($858 - $7424)	$4760<br>($2443 - $8411)	$12 020<br>($5367 - $20 170)	$29 282<br>($8789 - $69 563)	$38 475<br>($17 159 - $71 641)
Central Latin America	$1592<br>($896 - $2349)	$2040<br>($878 - $3872)	$3326<br>($1828 - $5183)	$8344<br>($3993 - $12 826)	$19 658<br>($7901 - $38 075)	$27 183<br>($13 143 - $44 897)
Colombia	$1605<br>($850 - $2514)	$2314<br>($667 - $5332)	$3328<br>($1665 - $5616)	$8230<br>($3838 - $13 373)	$21 834<br>($6258 - $55 497)	$26 920<br>($11 762 - $55 497)
Costa Rica	$2403<br>($1258 - $3831)	$3574<br>($1080 - $9102)	$5022<br>($2533 - $9277)	$12 743<br>($6129 - $20 714)	$34 994<br>($11 391 - $83 651)	$42 090<br>($18 887 - $83 651)
El Salvador	$1387<br>($740 - $2194)	$1273<br>($372 - $2966)	$2796<br>($1483 - $4426)	$7017<br>($3308 - $11 784)	$11 818<br>($3388 - $27 738)	$21 659<br>($9990 - $36 895)
Guatemala	$1351<br>($691 - $2207)	$1669<br>($512 - $4525)	$2759<br>($1408 - $4768)	$6847<br>($3176 - $11 313)	$15 226<br>($4354 - $40 975)	$21 616<br>($9866 - $40 975)
Honduras	$1082<br>($563 - $1795)	$1310<br>($379 - $3304)	$2205<br>($1121 - $3730)	$5336<br>($2571 - $9113)	$11 325<br>($3567 - $27 528)	$16 678<br>($7995 - $29 677)
Mexico	$1703<br>($902 - $2748)	$1893<br>($570 - $4636)	$3451<br>($1824 - $5643)	$8791<br>($4044 - $14 419)	$17 428<br>($5245 - $40 998)	$27 309<br>($12 422 - $45 808)
Nicaragua	$1023<br>($569 - $1663)	$1454<br>($457 - $3856)	$2126<br>($1137 - $3969)	$5106<br>($2319 - $8210)	$13 349<br>($4039 - $31 546)	$16 584<br>($7350 - $31 546)
Panama	$2457<br>($1302 - $3983)	$3834<br>($1146 - $9430)	$5175<br>($2649 - $9430)	$12 806<br>($6089 - $21 095)	$36 201<br>($10 607 - $91 059)	$42 890<br>($19 052 - $91 059)
Venezuela	$783<br>($435 - $1212)	$216<br>($65 - $523)	$1579<br>($872 - $2515)	$3774<br>($1751 - $6342)	$1730<br>($495 - $4095)	$11 599<br>($5441 - $19 189)
Tropical Latin America	$1880<br>($975 - $2982)	$2113<br>($600 - $5127)	$3796<br>($1990 - $6185)	$9849<br>($4627 - $16 065)	$20 480<br>($6237 - $48 533)	$30 693<br>($14 111 - $52 401)
Brazil	$1887<br>($974 - $3000)	$2111<br>($581 - $5200)	$3816<br>($1988 - $6247)	$9891<br>($4640 - $16 137)	$20 512<br>($6027 - $48 932)	$30 885<br>($14 202 - $52 881)
Paraguay	$1500<br>($809 - $2424)	$2167<br>($697 - $5281)	$3109<br>($1630 - $5485)	$7595<br>($3655 - $12 572)	$20 131<br>($5882 - $47 495)	$24 862<br>($11 296 - $47 495)
North Africa and Middle East	$1660<br>($987 - $2390)	$3071<br>($1617 - $5349)	$3775<br>($2160 - $6130)	$7948<br>($3929 - $12 222)	$21 787<br>($9502 - $38 986)	$28 365<br>($13 733 - $45 687)
Afghanistan	$688<br>($376 - $1096)	$1402<br>($432 - $3261)	$1582<br>($757 - $3261)	$2899<br>($1379 - $4839)	$8694<br>($2509 - $21 003)	$9939<br>($4289 - $21 003)
Algeria	$1353<br>($713 - $2141)	$2873<br>($886 - $7007)	$3205<br>($1479 - $7007)	$5967<br>($2789 - $9859)	$18 518<br>($5541 - $47 318)	$20 926<br>($8592 - $47 318)
Bahrain	$2704<br>($1436 - $4454)	$3447<br>($1036 - $9675)	$5567<br>($2882 - $9860)	$12 563<br>($5711 - $20 988)	$21 922<br>($6546 - $49 694)	$38 691<br>($17 626 - $64 536)
Egypt	$1190<br>($629 - $1942)	$1810<br>($530 - $4404)	$2493<br>($1243 - $4500)	$5207<br>($2430 - $8597)	$11 039<br>($3300 - $25 785)	$16 280<br>($7495 - $27 986)
Iran	$2229<br>($1195 - $3616)	$4689<br>($1447 - $12 301)	$5247<br>($2431 - $12 301)	$10 132<br>($4751 - $16 904)	$31 436<br>($9729 - $77 980)	$35 383<br>($15 064 - $77 980)
Iraq	$1220<br>($666 - $1958)	$2350<br>($712 - $5534)	$2736<br>($1333 - $5534)	$5306<br>($2462 - $8852)	$14 755<br>($4027 - $33 630)	$17 570<br>($7601 - $33 630)
Jordan	$1536<br>($817 - $2465)	$1453<br>($424 - $3829)	$3106<br>($1633 - $5084)	$6840<br>($3138 - $11 125)	$8994<br>($2760 - $21 637)	$21 047<br>($9841 - $35 097)
Kuwait	$3184<br>($1646 - $5140)	$4550<br>($1239 - $10 947)	$6577<br>($3268 - $11 470)	$14 623<br>($6902 - $24 283)	$28 791<br>($9171 - $70 677)	$45 459<br>($20 910 - $76 130)
Lebanon	$1744<br>($923 - $2813)	$1741<br>($549 - $4191)	$3516<br>($1859 - $5683)	$7729<br>($3692 - $12 854)	$10 589<br>($3271 - $25 488)	$23 787<br>($11 372 - $39 726)
Libya	$2409<br>($1210 - $3896)	$2192<br>($647 - $5211)	$4858<br>($2423 - $8030)	$10 887<br>($5138 - $18 070)	$13 307<br>($3878 - $30 094)	$33 481<br>($15 572 - $56 039)
Morocco	$1207<br>($643 - $1945)	$2423<br>($688 - $5743)	$2764<br>($1291 - $5743)	$5164<br>($2375 - $8502)	$14 569<br>($4741 - $32 518)	$17 223<br>($7635 - $32 518)

Oman	$2119 ($1112 - $3469)	$2531 ($791 - $6135)	$4299 ($2249 - $7063)	$9617 ($4534 - $15 997)	$16 181 ($5085 - $39 049)	$29 649 ($14 117 - $49 227)
Palestine	$1730 ($881 - $2902)	$2582 ($711 - $6454)	$3608 ($1798 - $6718)	$7711 ($3700 - $12 713)	$16 076 ($4951 - $36 797)	$24 044 ($11 194 - $41 767)
Qatar	$3412 ($1895 - $5416)	$4811 ($1549 - $11 781)	$7050 ($3777 - $12 008)	$16 111 ($7685 - $27 179)	$32 159 ($8847 - $79 714)	$50 182 ($23 938 - $87 947)
Saudi Arabia	$3052 ($1635 - $4917)	$5258 ($1595 - $13 365)	$6624 ($3268 - $13 365)	$14 167 ($6955 - $23 768)	$34 772 ($10 596 - $82 662)	$45 347 ($21 659 - $84 565)
Sudan	$648 ($332 - $1058)	$879 ($245 - $2140)	$1330 ($669 - $2286)	$2749 ($1283 - $4531)	$5197 ($1539 - $12 155)	$8515 ($3975 - $14 733)
Syria	$629 ($341 - $997)	$516 ($153 - $1243)	$1268 ($685 - $2058)	$2621 ($1170 - $4394)	$2970 ($787 - $7473)	$8059 ($3610 - $13 706)
Tunisia	$1475 ($783 - $2308)	$3075 ($891 - $7297)	$3441 ($1558 - $7297)	$6563 ($3048 - $10 987)	$19 792 ($6168 - $49 862)	$22 656 ($9508 - $49 862)
Turkey	$1672 ($897 - $2697)	$2984 ($912 - $7742)	$3660 ($1859 - $7742)	$7329 ($3464 - $11 988)	$18 236 ($5305 - $41 758)	$23 464 ($10 543 - $42 388)
United Arab Emirates	$3431 ($1857 - $5421)	$4425 ($1351 - $10 623)	$7025 ($3651 - $11 676)	$16 218 ($7329 - $26 726)	$29 105 ($8815 - $65 317)	$49 935 ($22 861 - $83 062)
Yemen	$563 ($298 - $902)	$404 ($111 - $1001)	$1134 ($605 - $1854)	$2324 ($1101 - $3749)	$2191 ($689 - $4924)	$7145 ($3357 - $11 720)
South Asia	$685 ($379 - $1096)	$1137 ($409 - $2422)	$1456 ($761 - $2643)	$3343 ($1570 - $5549)	$10 395 ($3638 - $24 392)	$11 719 ($4994 - $24 532)
Bangladesh	$580 ($315 - $919)	$1160 ($354 - $2956)	$1334 ($622 - $2956)	$2757 ($1340 - $4560)	$10 286 ($2924 - $23 869)	$10 805 ($4316 - $23 869)
Bhutan	$758 ($402 - $1203)	$770 ($239 - $1860)	$1532 ($814 - $2469)	$3669 ($1704 - $6141)	$6788 ($2047 - $14 649)	$11 350 ($5347 - $19 020)
India	$712 ($383 - $1164)	$1185 ($318 - $2895)	$1524 ($758 - $2938)	$3447 ($1577 - $5832)	$10 717 ($3436 - $27 099)	$12 087 ($4857 - $27 099)
Nepal	$628 ($332 - $1000)	$1128 ($325 - $2831)	$1377 ($656 - $2831)	$3038 ($1413 - $4945)	$10 390 ($3313 - $24 205)	$11 220 ($4638 - $24 205)
Pakistan	$551 ($277 - $886)	$697 ($209 - $1879)	$1129 ($558 - $1994)	$2579 ($1147 - $4226)	$5791 ($1623 - $13 278)	$8128 ($3569 - $13 965)
East Asia	$2766 ($1528 - $4516)	$10 159 ($3201 - $25 536)	$10 221 ($3454 - $25 546)	$8882 ($4405 - $14 523)	$33 534 ($11 320 - $78 700)	$35 444 ($14 133 - $79 606)
China	$2763 ($1527 - $4543)	$10 246 ($3194 - $26 031)	$10 288 ($3458 - $26 031)	$8354 ($4030 - $13 850)	$33 736 ($10 916 - $80 448)	$34 870 ($13 591 - $80 448)
North Korea	$960 ($504 - $1559)	$1059 ($304 - $2734)	$1947 ($1014 - $3210)	$3034 ($1398 - $4955)	$3033 ($954 - $6929)	$9327 ($4232 - $15 288)
Taiwan	$3776 ($1968 - $6279)	$6344 ($1872 - $16 215)	$8121 ($3973 - $16 215)	$18 607 ($8937 - $31 649)	$30 133 ($8576 - $73 664)	$57 322 ($27 287 - $98 739)
Oceania	$1360 ($795 - $2019)	$1667 ($736 - $3253)	$2763 ($1536 - $4352)	$6286 ($3074 - $9884)	$7792 ($3151 - $15 041)	$18 734 ($9089 - $30 999)
American Samoa	$2607 ($1411 - $4169)	$2564 ($676 - $6370)	$5263 ($2792 - $8568)	$9148 ($4209 - $15 216)	$8666 ($2515 - $21 175)	$28 132 ($12 856 - $47 061)
Federated States of Micronesia	$1400 ($754 - $2232)	$2031 ($592 - $4582)	$2891 ($1507 - $4936)	$4613 ($2233 - $7627)	$6548 ($2075 - $15 193)	$14 183 ($6915 - $23 657)
Fiji	$1587 ($845 - $2561)	$2170 ($678 - $5099)	$3267 ($1691 - $5688)	$5396 ($2584 - $9103)	$7436 ($2191 - $16 902)	$16 593 ($8132 - $28 197)
Guam	$3311 ($1720 - $5461)	$4605 ($1238 - $12 109)	$6855 ($3374 - $12 454)	$11 873 ($5390 - $19 789)	$16 260 ($4720 - $39 501)	$36 526 ($16 513 - $60 120)
Kiribati	$1595 ($838 - $2632)	$1469 ($437 - $3518)	$3219 ($1674 - $5335)	$5376 ($2538 - $8937)	$4683 ($1441 - $10 765)	$16 523 ($7905 - $27 608)
Marshall Islands	$2550 ($1343 - $4129)	$3337 ($955 - $7714)	$5225 ($2641 - $8848)	$8909 ($4172 - $14 851)	$11 539 ($3512 - $28 126)	$27 378 ($12 931 - $45 223)
Northern Mariana Islands	$2491 ($1378 - $4056)	$3203 ($970 - $8454)	$5101 ($2720 - $8962)	$8754 ($3980 - $14 985)	$11 005 ($3462 - $25 253)	$26 914 ($12 349 - $46 534)
Papua New Guinea	$992 ($534 - $1611)	$1185 ($364 - $2919)	$2014 ($1031 - $3354)	$3287 ($1551 - $5290)	$3900 ($1154 - $9221)	$10 102 ($4778 - $16 667)
Samoa	$1735 ($917 - $2758)	$2398 ($713 - $5873)	$3594 ($1834 - $5996)	$5804 ($2627 - $9928)	$7737 ($2182 - $19 422)	$17 854 ($8150 - $30 161)
Solomon Islands	$1178 ($647 - $1832)	$1090 ($337 - $2521)	$2382 ($1315 - $3724)	$3942 ($1753 - $6598)	$3538 ($988 - $8554)	$12 123 ($5360 - $20 961)
Tonga	$1743 ($975 - $2812)	$2102 ($665 - $5210)	$3543 ($1908 - $5995)	$5897 ($2855 - $9669)	$6955 ($2249 - $16 971)	$18 127 ($8935 - $29 754)
Vanuatu	$1156 ($603 - $1810)	$1101 ($344 - $2810)	$2334 ($1189 - $3738)	$3836 ($1766 - $6357)	$3498 ($1107 - $8132)	$11 792 ($5389 - $19 843)
Southeast Asia	$1541 ($898 - $2285)	$3510 ($1662 - $6281)	$3830 ($1986 - $6573)	$5435 ($2699 - $8328)	$12 744 ($5641 - $23 955)	$17 227 ($8447 - $28 281)

Cambodia	$1073 ($581 - $1721)	$1974 ($624 - $5070)	$2363 ($1200 - $5070)	$3477 ($1562 - $5623)	$6431 ($1924 - $14 847)	$10 772 ($4863 - $18 327)
Indonesia	$1283 ($658 - $2033)	$2970 ($856 - $7047)	$3189 ($1400 - $7047)	$4298 ($2018 - $7140)	$10 349 ($3162 - $24 041)	$13 702 ($6238 - $24 396)
Laos	$937 ($520 - $1581)	$1330 ($417 - $3250)	$1930 ($1044 - $3518)	$3032 ($1460 - $4990)	$4226 ($1269 - $9870)	$9327 ($4523 - $15 765)
Malaysia	$2356 ($1272 - $3912)	$5491 ($1606 - $14 228)	$5930 ($2576 - $14 228)	$8090 ($3877 - $13 436)	$19 024 ($5635 - $47 158)	$25 687 ($11 428 - $47 814)
Maldives	$3604 ($1904 - $5947)	$6240 ($1750 - $15 994)	$7830 ($3726 - $15 994)	$13 088 ($5945 - $22 186)	$23 008 ($6749 - $57 425)	$40 375 ($18 253 - $68 999)
Mauritius	$2785 ($1501 - $4514)	$8449 ($2385 - $20 912)	$8599 ($3216 - $20 912)	$9837 ($4612 - $16 397)	$31 492 ($9703 - $75 366)	$34 904 ($14 495 - $75 366)
Myanmar	$925 ($504 - $1461)	$1747 ($917 - $2963)	$1905 ($1023 - $3081)	$3000 ($1413 - $5257)	$5818 ($2579 - $10 368)	$9221 ($4404 - $16 150)
Philippines	$1433 ($750 - $2296)	$3165 ($929 - $7788)	$3464 ($1564 - $7788)	$4792 ($2187 - $7873)	$10 845 ($3146 - $26 291)	$15 130 ($7052 - $27 818)
Sri Lanka	$1425 ($747 - $2330)	$2488 ($719 - $6026)	$3081 ($1505 - $6026)	$4785 ($2332 - $7984)	$8414 ($2556 - $19 421)	$14 752 ($7301 - $24 943)
Seychelles	$2800 ($1510 - $4390)	$3609 ($1102 - $9202)	$5723 ($3077 - $9587)	$9899 ($4530 - $16 436)	$12 793 ($3759 - $30 031)	$30 441 ($13 891 - $51 020)
Thailand	$1956 ($1022 - $3180)	$3823 ($1124 - $8743)	$4415 ($2124 - $8874)	$6783 ($3279 - $11 490)	$13 666 ($4070 - $35 567)	$21 133 ($9902 - $39 050)
Timor-Leste	$1036 ($535 - $1724)	$1754 ($504 - $4396)	$2228 ($1061 - $4396)	$3434 ($1659 - $5730)	$5882 ($1848 - $13 906)	$10 586 ($5087 - $17 742)
Vietnam	$1478 ($804 - $2407)	$4478 ($1408 - $11 151)	$4555 ($1726 - $11 151)	$5003 ($2241 - $8117)	$16 205 ($4793 - $38 021)	$17 892 ($7417 - $38 021)
Central Sub-Saharan Africa	$489 ($272 - $746)	$614 ($256 - $1292)	$1007 ($554 - $1644)	$2689 ($1265 - $4162)	$5572 ($2175 - $10 724)	$8786 ($4052 - $14 707)
Angola	$614 ($324 - $993)	$586 ($167 - $1390)	$1240 ($654 - $2047)	$2944 ($1332 - $4806)	$5084 ($1449 - $11 616)	$9099 ($4142 - $14 929)
Central African Republic	$424 ($224 - $698)	$375 ($107 - $999)	$856 ($449 - $1408)	$1979 ($893 - $3318)	$3080 ($932 - $7055)	$6090 ($2784 - $10 234)
Congo	$545 ($290 - $884)	$491 ($139 - $1201)	$1099 ($578 - $1802)	$2580 ($1150 - $4291)	$4087 ($1181 - $9379)	$7939 ($3585 - $13 292)
Democratic Republic of the Congo	$378 ($206 - $613)	$617 ($190 - $1573)	$803 ($410 - $1573)	$1754 ($835 - $2798)	$5207 ($1559 - $11 801)	$5972 ($2539 - $11 801)
Equatorial Guinea	$1162 ($639 - $1936)	$1084 ($527 - $1955)	$2344 ($1280 - $3992)	$5824 ($2625 - $9607)	$9855 ($4197 - $17 621)	$17 899 ($8281 - $29 479)
Gabon	$1217 ($655 - $1962)	$1024 ($309 - $2378)	$2453 ($1337 - $4086)	$6143 ($2833 - $10 407)	$9283 ($2712 - $22 040)	$18 897 ($8827 - $31 907)
Eastern Sub-Saharan Africa	$529 ($320 - $777)	$743 ($384 - $1304)	$1098 ($634 - $1739)	$2697 ($1334 - $4255)	$6737 ($3081 - $12 304)	$8668 ($4159 - $14 501)
Burundi	$440 ($227 - $728)	$499 ($142 - $1207)	$894 ($454 - $1481)	$2071 ($946 - $3435)	$4202 ($1292 - $9826)	$6449 ($2916 - $11 401)
Comoros	$703 ($382 - $1131)	$568 ($163 - $1380)	$1417 ($754 - $2338)	$3395 ($1597 - $5656)	$4840 ($1339 - $11 771)	$10 445 ($4809 - $17 656)
Djibouti	$594 ($322 - $945)	$409 ($128 - $1065)	$1198 ($646 - $1919)	$2833 ($1300 - $4778)	$3403 ($1052 - $7843)	$8709 ($3886 - $14 893)
Eritrea	$300 ($164 - $478)	$143 ($44 - $335)	$605 ($335 - $984)	$1371 ($643 - $2297)	$1125 ($315 - $2672)	$4216 ($1952 - $7186)
Ethiopia	$427 ($234 - $691)	$652 ($193 - $1751)	$902 ($461 - $1757)	$2011 ($951 - $3341)	$5732 ($1674 - $14 315)	$6758 ($2908 - $14 315)
Kenya	$825 ($421 - $1323)	$1096 ($317 - $2744)	$1696 ($840 - $2981)	$3989 ($1886 - $6765)	$9502 ($2750 - $24 441)	$12 780 ($5794 - $24 655)
Madagascar	$395 ($214 - $628)	$331 ($104 - $809)	$796 ($420 - $1273)	$1826 ($863 - $3079)	$2687 ($868 - $6444)	$5621 ($2638 - $9729)
Malawi	$491 ($254 - $779)	$691 ($204 - $1539)	$1014 ($512 - $1743)	$2339 ($1112 - $4000)	$6140 ($1942 - $13 559)	$7578 ($3500 - $13 797)
Mozambique	$466 ($255 - $742)	$754 ($253 - $1929)	$992 ($517 - $1946)	$2190 ($1082 - $3618)	$6488 ($1999 - $15 044)	$7435 ($3386 - $15 044)
Rwanda	$601 ($334 - $953)	$1405 ($455 - $3307)	$1506 ($692 - $3307)	$2883 ($1326 - $4892)	$13 059 ($3749 - $33 057)	$13 286 ($4685 - $33 057)
Somalia	$232 ($123 - $360)	$227 ($71 - $565)	$470 ($245 - $754)	$1048 ($488 - $1709)	$1877 ($550 - $4484)	$3250 ($1522 - $5522)
South Sudan	$422 ($224 - $672)	$260 ($79 - $658)	$851 ($453 - $1377)	$1971 ($973 - $3237)	$2143 ($633 - $5111)	$6062 ($2984 - $10 152)
Tanzania	$520 ($285 - $824)	$781 ($240 - $1785)	$1083 ($576 - $1868)	$2464 ($1114 - $4160)	$6964 ($1991 - $17 118)	$8286 ($3413 - $17 118)

Uganda	$544 ($295 - $866)	$638 ($199 - $1616)	$1105 ($583 - $1804)	$2601 ($1206 - $4376)	$5511 ($1522 - $12 710)	$8135 ($3685 - $13 981)
Zambia	$600 ($321 - $998)	$565 ($173 - $1519)	$1211 ($630 - $2004)	$2863 ($1372 - $4837)	$4803 ($1462 - $11 257)	$8845 ($4294 - $15 264)
Southern Sub-Saharan Africa	$1566 ($840 - $2435)	$1841 ($649 - $4532)	$3167 ($1619 - $5159)	$8185 ($3830 - $13 355)	$17 353 ($5826 - $36 843)	$25 624 ($11 851 - $42 432)
Botswana	$1626 ($899 - $2619)	$2301 ($738 - $5773)	$3371 ($1807 - $6003)	$8308 ($3985 - $13 767)	$21 737 ($6327 - $52 504)	$27 175 ($12 183 - $52 733)
Lesotho	$853 ($451 - $1380)	$1681 ($510 - $4323)	$1945 ($928 - $4323)	$4220 ($1915 - $7041)	$15 743 ($4593 - $37 030)	$16 567 ($6314 - $37 030)
Namibia	$1512 ($839 - $2418)	$1567 ($475 - $3655)	$3051 ($1666 - $5000)	$7744 ($3772 - $12 638)	$14 616 ($4709 - $34 656)	$24 028 ($11 478 - $39 517)
South Africa	$1662 ($872 - $2634)	$1963 ($594 - $4980)	$3373 ($1750 - $5511)	$8445 ($3872 - $13 950)	$17 724 ($5384 - $40 101)	$26 329 ($11 806 - $44 578)
Swaziland	$1140 ($614 - $1865)	$1580 ($459 - $3938)	$2356 ($1222 - $4209)	$5607 ($2610 - $9261)	$14 201 ($4074 - $34 623)	$18 167 ($8142 - $34 623)
Zimbabwe	$659 ($352 - $1073)	$494 ($151 - $1241)	$1328 ($679 - $2200)	$3223 ($1478 - $5375)	$4290 ($1155 - $9890)	$9909 ($4651 - $16 730)
Western Sub-Saharan Africa	$650 ($376 - $966)	$862 ($395 - $1660)	$1350 ($767 - $2079)	$3272 ($1639 - $5095)	$8195 ($3315 - $15 902)	$10 753 ($5250 - $17 965)
Benin	$445 ($241 - $708)	$354 ($118 - $914)	$897 ($476 - $1443)	$2105 ($1025 - $3572)	$3016 ($887 - $7332)	$6473 ($3118 - $11 060)
Burkina Faso	$536 ($287 - $909)	$1048 ($303 - $2705)	$1221 ($576 - $2705)	$2537 ($1199 - $4180)	$9039 ($2608 - $20 545)	$9614 ($3873 - $20 545)
Cameroon	$561 ($292 - $916)	$451 ($132 - $1097)	$1132 ($590 - $1874)	$2679 ($1331 - $4388)	$3808 ($1224 - $9006)	$8243 ($4058 - $13 830)
Cape Verde	$1056 ($560 - $1652)	$1317 ($406 - $3289)	$2153 ($1139 - $3615)	$5286 ($2588 - $8794)	$12 353 ($3828 - $27 335)	$16 650 ($7752 - $29 980)
Chad	$442 ($239 - $710)	$372 ($118 - $867)	$892 ($485 - $1459)	$2074 ($984 - $3512)	$3038 ($852 - $7286)	$6382 ($3031 - $10 718)
Cote d'Ivoire	$687 ($373 - $1114)	$632 ($198 - $1624)	$1386 ($741 - $2255)	$3323 ($1590 - $5542)	$5542 ($1578 - $13 104)	$10 268 ($4817 - $17 496)
The Gambia	$513 ($278 - $822)	$790 ($254 - $1970)	$1081 ($567 - $1971)	$2408 ($1130 - $3940)	$6844 ($2093 - $15 498)	$8045 ($3470 - $15 811)
Ghana	$693 ($382 - $1092)	$1018 ($313 - $2680)	$1444 ($771 - $2715)	$3329 ($1611 - $5360)	$9003 ($2819 - $21 382)	$10 980 ($5093 - $21 382)
Guinea	$588 ($323 - $930)	$737 ($232 - $1814)	$1199 ($639 - $2003)	$2809 ($1327 - $4715)	$6664 ($1918 - $16 479)	$8948 ($4045 - $16 976)
Guinea-Bissau	$600 ($334 - $960)	$682 ($213 - $1673)	$1219 ($646 - $2045)	$2866 ($1261 - $4742)	$5886 ($1832 - $14 115)	$8931 ($3947 - $15 556)
Liberia	$626 ($323 - $972)	$1475 ($399 - $3617)	$1583 ($688 - $3617)	$3009 ($1473 - $5015)	$13 650 ($4187 - $31 964)	$13 829 ($5051 - $31 964)
Mali	$453 ($244 - $720)	$337 ($103 - $816)	$914 ($500 - $1479)	$2149 ($966 - $3679)	$2877 ($850 - $7144)	$6605 ($2991 - $11 643)
Mauritania	$657 ($350 - $1041)	$632 ($184 - $1650)	$1329 ($710 - $2181)	$3165 ($1483 - $5213)	$5399 ($1641 - $12 403)	$9762 ($4609 - $16 366)
Niger	$469 ($239 - $765)	$511 ($142 - $1277)	$951 ($474 - $1560)	$2162 ($989 - $3614)	$4105 ($1277 - $10 496)	$6730 ($3098 - $12 092)
Nigeria	$726 ($380 - $1206)	$1013 ($291 - $2541)	$1497 ($765 - $2648)	$3535 ($1715 - $5839)	$8969 ($2743 - $20 440)	$11 378 ($5305 - $20 866)
Sao Tome and Principe	$803 ($430 - $1319)	$579 ($186 - $1445)	$1619 ($868 - $2624)	$3896 ($1778 - $6490)	$4884 ($1361 - $11 650)	$11 980 ($5535 - $20 300)
Senegal	$661 ($347 - $1079)	$780 ($225 - $2010)	$1344 ($711 - $2232)	$3172 ($1469 - $5197)	$6804 ($2082 - $16 508)	$9922 ($4633 - $17 216)
Sierra Leone	$689 ($365 - $1107)	$943 ($289 - $2158)	$1415 ($734 - $2409)	$3268 ($1618 - $5429)	$8225 ($2459 - $20 275)	$10 578 ($5040 - $20 323)
Togo	$513 ($277 - $807)	$655 ($209 - $1555)	$1047 ($577 - $1695)	$2456 ($1148 - $4071)	$5919 ($1925 - $14 052)	$7845 ($3644 - $14 195)

Notes: All spending measured in 2019 US dollars.

Figure S11: Future health scenarios: Dementia prevalence and spending by World Bank income group in 2050

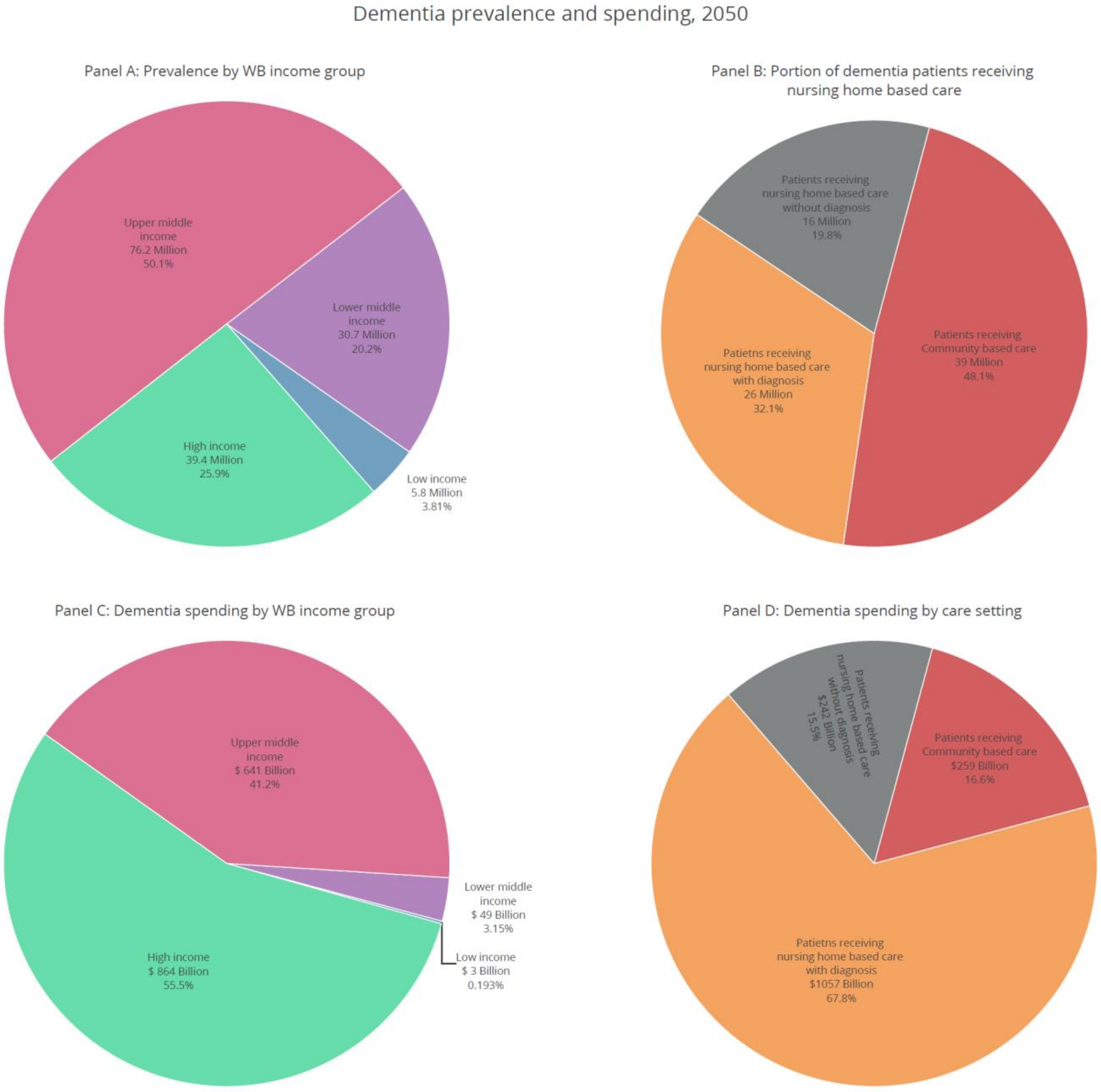

Sensitivity analyses

Severity sensitivity analysis

Literature on dementia spending indicates that unit costs increase as the condition progresses and it gets more severe. We decided to evaluate our results relative to including disease prevalence, diagnosis and treatment rates, nursing-home-based care rates, and unit costs disaggregated by severity. In our model we have three severity states; mild, moderate, and severe dementia. We looked at data extracted for the main analysis and selected data points where unit cost was reported by severity. In total, 42 data sources reported dementia unit costs disaggregated by severity. We cleaned and standardized 138 data points. The date range of the data spans from 1996 to 2016. We used prevalence by severity from GBD 2019. As in the main analysis, we removed outliers before applying ST-GPR to each key driver, based upon Cook's distance. We also removed data points from the same data source violated the assumption unit cost for treating a mild dementia case < unit cost for treating a moderate dementia case < unit cost for treating a severe dementia case. We also removed data points from the same data source where the average spending on all dementia severities was higher than spending on severe dementia. The table below contains all data points that were removed.

Country	Year	Severity	Care setting	Value	Currency
Canada	2003	Moderate	community-based care	9451	2019 USD
Turkey	2003	All	community-based care	3013	2019 USD
Switzerland	2007	Moderate	community-based care	12 762	2019 USD
Germany	2014	All	community-based care	2151	2019 USD
Germany	2014	Severe	community-based care	1914	2019 USD
Italy	2014	Moderate	community-based care	23 780	2019 USD

We ran a mixed effect model with fixed effects on severity and care setting and random intercepts on location to generate dementia spending estimates. We then used those estimates to generate the ratio of dementia spending by severity over total dementia spending. To strengthen our estimates and to generate a complete time series of unit costs we used ST-GPR. We multiplied the estimates from ST-GPR by the ratios from the mixed effects model to generate a final set of dementia estimates by severity. Figure 7 shows a comparison of the estimates modelled with (the estimates based on this sensitivity analysis) and without severity (our primary estimates).

Figure S12: Total dementia spending estimates modelled with (sensitivity analysis) and without severity in 2019 (main estimates) (2019 USD)

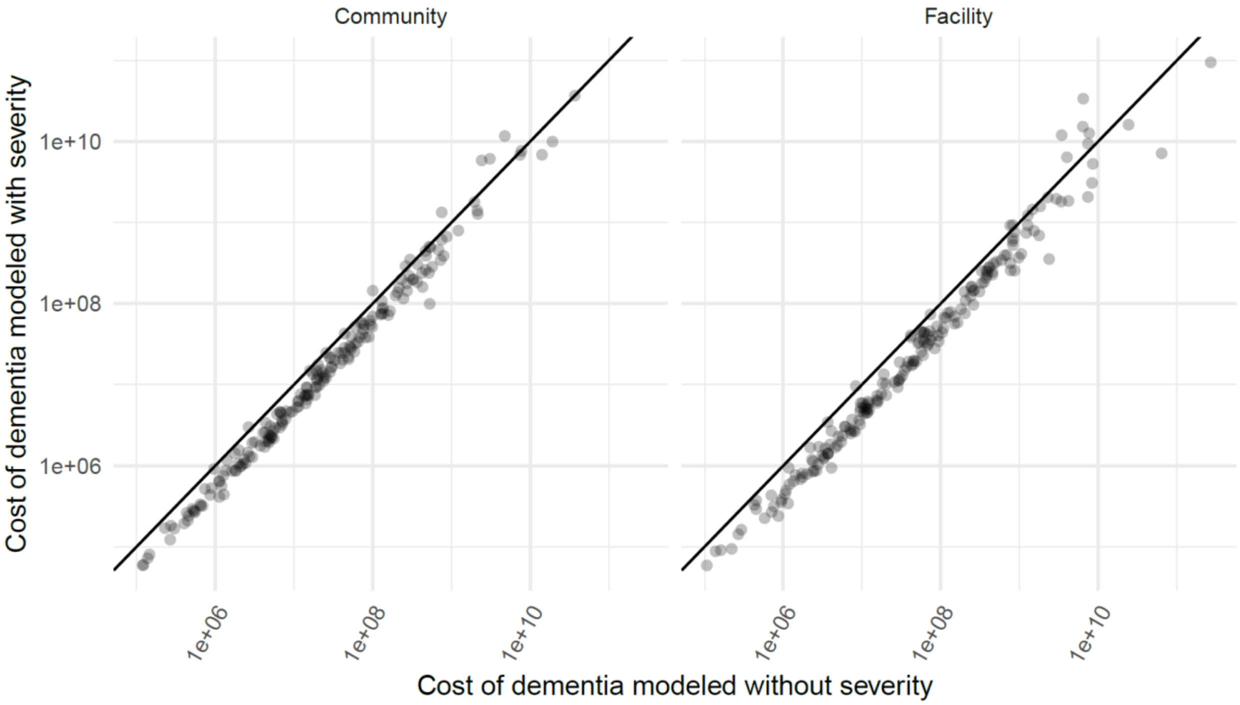

Costs attributable to dementia by diagnosis status

To our knowledge there is no literature on the healthcare cost that is attributable to dementia by severity. However these is strong evidence that the cost of dementia increases as the condition gets more severe. With this knowledge, for our main analysis we used half the healthcare cost attributable to dementia for those without a diagnosis. We assumed that patient with undiagnosed dementia suffer from less severe cases and require more care due to other conditions common among the elderly. This sensitivity analysis tests that assumption by generating estimates where the attributable cost to dementia is identical for those with and without a diagnosis. Figure S12 compares attributable cost to dementia from the main analysis to estimates from our sensitivity analysis.

Figure S13: Attributable dementia spending estimates modelled with attributable fraction

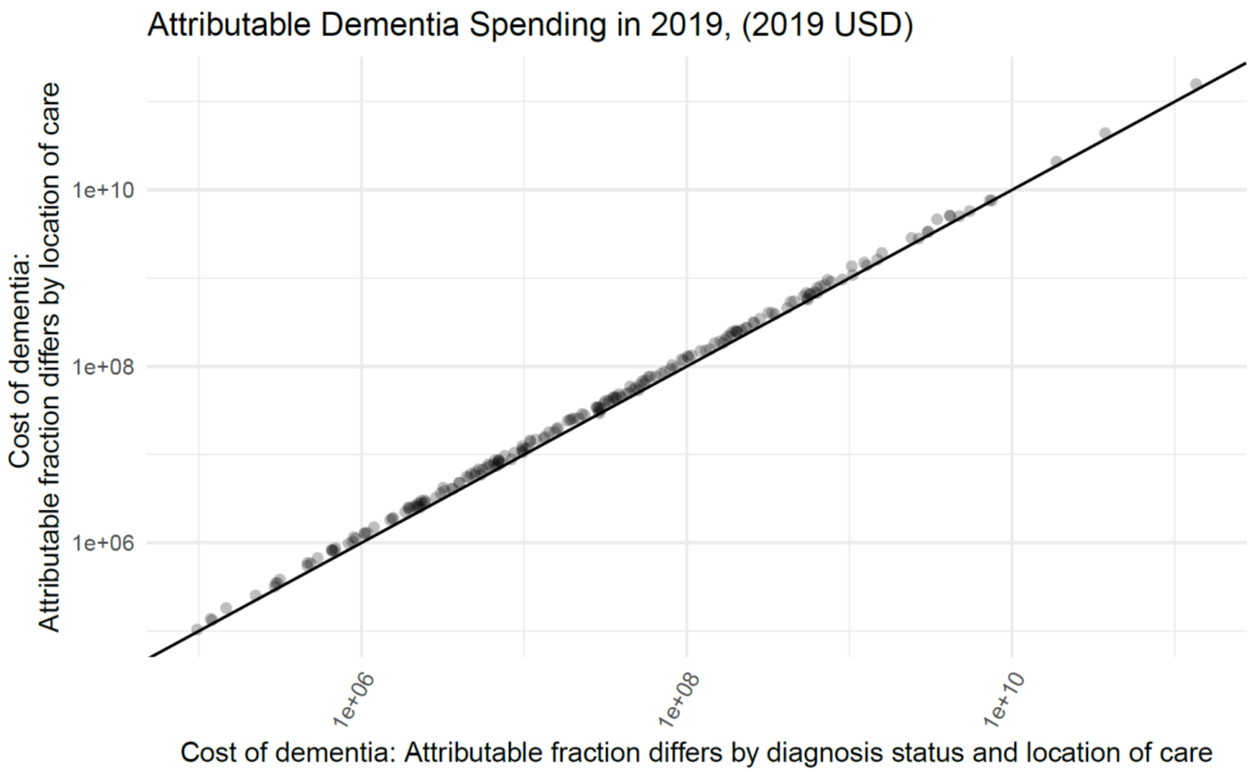

Mean absolute deviation analysis

For countries with data, we removed all input data and re-estimated dementia spending for 2019. We then assessed the statistical significance of the deviation of each country. Our analysis show that roughly two thirds of all countries would not have a statistically significant difference from the baseline even in the complete absence of data.

Table S13. Results from mean absolute deviation analysis

Omitted Country	Estimates were statistically significant in the absence of data
Argentina	TRUE
Australia	TRUE
Belgium	FALSE
Brazil	FALSE
Canada	FALSE
Switzerland	FALSE
Chile	TRUE
China	FALSE
Colombia	FALSE

Czech Republic	FALSE
Germany	FALSE
Denmark	FALSE
Dominican Republic	FALSE
Spain	FALSE
Finland	FALSE
France	FALSE
United Kingdom	FALSE
Hungary	FALSE
India	TRUE
Ireland	FALSE
Israel	TRUE
Italy	FALSE
Japan	TRUE
South Korea	TRUE
Mexico	FALSE
Netherlands	FALSE
Norway	FALSE
New Zealand	FALSE
Peru	TRUE
Portugal	FALSE
Russian Federation	TRUE
Singapore	TRUE
Sweden	FALSE
Thailand	FALSE
Turkey	TRUE
Taiwan (Province of China)	FALSE
USA	FALSE
Venezuela	TRUE
